# Herbal medicine as adjunctive therapy with antidepressants for post-stroke depression: a systematic review and network meta-analysis of randomized controlled trials

**DOI:** 10.3389/fphar.2023.1180071

**Published:** 2023-07-14

**Authors:** Jian Zhang, Shuping Ming, Xiaoming Chen, Teng Zhang, Hongyu Qian, Shixiong Peng, Yanbing Ding

**Affiliations:** ^1^ Department of Encephalopathy, Hubei Provincial Hospital of Traditional Chinese Medicine, Wuhan, China; ^2^ Department of Traditional Chinese Medicine Encephalopathy, Hubei Province Traditional Chinese Medicine Research Institute, Wuhan, China; ^3^ First Clinical College, Hubei University of Chinese Medicine, Wuhan, Hubei Province, China

**Keywords:** herbal medicine, adjunctive therapy, antidepressants, post-stroke depression, network meta analysis

## Abstract

**Background:** Herbal medicine can provide adjunctive therapy for adults with post-stroke depression. This study summarizes the latest evidence regarding the harms and benefits of herbal antidepressants.

**Methods:** The literature searched from the Cochrane Library (using the OVID platform), Embase, PubMed, the China National Knowledge Infrastructure (CNKI), the Wan Fang Data Knowledge Service Platform, and the China Scientific Journal Database (VIP) from their inception to 18 August 2021, for randomized controlled trials of herbal medicine in adults with post-stroke depression, were included in this systematic review and network meta-analysis. The search was updated on 1 December 2022. To summarize the evidence, the frequentist random-effect network meta-analyses were conducted. To categorize interventions, rate the certainty of the evidence, and present the findings, the Grading of Recommendations Assessment, Development, and Evaluation (GRADE) frameworks were carried out. The registration number of this study on PROSPERO website is CRD 42021273956.

**Findings:** Of 1132 citations identified from the search, 51 randomized clinical trials, totaling 4,507 participants, met the inclusion criteria for this study. For response rate, Shugan Jieyu capsule (SJC) plus selective serotonin reuptake inhibitors (SSRI), Jie-Yu Pills plus SSRI, and Wuling capsule plus SSRI were shown to be among the most effective with moderate certainty of evidence (RR: 1·45, 95%CI: 1·23 to 1·7; RR: 1·35, 95%CI: 1·09 to 1·68; RR: 1·32, 95%CI: 1·09 to 1·59). In terms of mean changes in Hamilton depression scale (HAMD) score after the completion of treatment, Wuling capsule plus Hypericum and Wuling capsule plus SSRI were found to be among the most effective in reducing symptoms of depression with moderate certainty of evidence (MD: 10·12, 95%CI: −17·25 to −2·99; MD: −3·81, 95%CI: −6·19 to −1·42). The network meta-analysis (NMA) showed that SJC may be a safer intervention than SSRI in terms of both total gastrointestinal and total nervous system events with moderate certainty of evidence (RR:0.34, 95%CI:0.18, 0.62 and RR: 0.11, 95%CI: 0.03, 0.35, respectively).

**Interpretation:** SJC plus SSRI**,** Jie-Yu Pills plus SSRI, and Wuling capsule plus SSRI were among the most effective in terms of HAMD score reduction response rates. Low to very low certainty of evidence revealed no increased risk of gastrointestinal and nervous system events.

**Systematic Review Registration:** https://www.crd.york.ac.uk/PROSPERO/display_record.php?RecordID=273956; Identifier: CRD42021273956.

## 1 Introduction

Post-stroke depression (PSD) is the most common psychiatric disorder associated with stroke and affects approximately one-third of stroke survivors (Ayerbe et al., 2013; Villa et al., 2018). The PSD can worsen the rehabilitation of neurological functions, reduce quality of life, increase the risk of cognitive impairment, and result in suicide or cardiovascular disease-related mortality (Robinson et al., 1986; Herrmann et al., 1998; House et al., 2001; Jaracz et al., 2002; Williams et al., 2004; Jørgensen et al., 2016).

Therefore, antidepressants are strongly recommended (class I recommendation) by the American Heart Association/American Stroke Association in their acute ischemic stroke guideline for 2019 (Powers et al., 2019). Because the majority of patients are elderly and cannot tolerate the side effects of tricyclic antidepressants, the selective serotonin reuptake inhibitors (SSRI) have been used as the first-line treatment for PSD among several antidepressants (Castilla-Guerra et al., 2020; Mortensen and Andersen, 2021). In addition, a systematic review and meta‐analysis proved SSRI can be an effective treatment for improving poststroke recovery (Kalbouneh et al., 2022). However, due to the significantly increased risks of adverse outcomes, such as seizures, falls, and delirium (Hackett et al., 2008; Mortensen and Andersen, 2021), and delayed onset of action, which can take two to three weeks or longer to become evident (National Collaborating Centre for Mental Health, 2004), SSRI are not always well tolerated.

In recent years, due to the concerns regarding the benefits and risks of these commonly accepted antidepressants, herbal medicines that contain traditional Chinese medicine (TCM) are gaining interests and recognitions (Jun et al., 2014; Peng et al., 2014; Zhao et al., 2020). The efficacy of herbs in depression patients has been investigated and proved by several trials (Zhao et al., 2018). Combined with other antidepressants, herbal medicine can significantly improve the symptoms of PSD, however its exact effect is largely unknown. Moreover, there is a lack of evidence regarding the comparative effectiveness of a number of choices for different types of herbal drugs that are commonly used. Therefore, to further clarify the effect of herbs alone or in combination with antidepressants in PSD patients, this network meta-analysis was performed.

## 2 Methods

Our present analysis was strictly performed according to the Preferred Reporting Items for Systematic Reviews and Meta-Analyses (PRISMA-2020) guidelines and the extension statement for network meta-analysis (PRISMA-NMA) (Hutton et al., 2015; Page et al., 2021) (Supplementary Appendix S1). The protocol of this review was registered on the website of PROSPERO (CRD 42021273956).

### 2.1 Search strategy and information sources

For the searching of literature, the Cochrane Library (using the OVID platform), Embase, PubMed, the China National Knowledge Infrastructure (CNKI), the Wanfang Data Knowledge Service Platform, and the China Science and Technology Journal Database from their inception to 18 August 2021 were used. By cross-checking the reference list of the key reviews (Supplementary Appendix S2), the search results were supplemented. The search was updated on 1 December 2022.

The strategy for the search was constructed using the free terms, such as “herbal medicine”, “phytotherapy”, “plant extracts”, “medicinal plants”, “Chinese herbal drugs”, “Chinese traditional medicine”, “Ginkgo biloba”, and “Eleutherococcus” (see details in Supplementary Appendix S2), and Medical Subject Headings (MeSH). To filter the titles, abstracts, and full-texts independently, two reviewers (T.Z. and J.Z.) were employed and EndNote X9 (Clarivate Analytics, United States) and NoteExpress 3.4 (Corporation for Aegean Yuezhi Technology, China) were applied after removing duplicates. To extract the information, such as trial characteristics (first author, publication year, study design and setting, diagnostic criteria, and total sample size) and baseline participant characteristics (mean age, sex ratio, mean Hamilton depression scale (HAMD) score), interventions (drugs and doses), and outcomes (adverse events, tolerability, and response rate), a pre-defined Microsoft Excel spreadsheets was applied. The data was extracted by two independent researchers (J.Z. and X. C). These two reviewers resolved discrepancies through discussions and consensus. If necessary, a third-reviewer was invited for the consensus adjudication.

### 2.2 Eligibility criteria of randomized controlled trials

The randomized controlled trials that satisfied the following criteria were included in the final assessment (Ayerbe et al., 2013). Studies that enrolled hemorrhagic or ischemic stroke subjects, who were diagnosed according to the corresponding clinical guideline criteria, magnetic resonance imaging (MRI) or computed tomography (CT) (Villa et al., 2018). The enrolled subjects were diagnosed with depression according to the Diagnosis and Statistical Manual of Mental Disorders, Fourth Edition (DSM-IV) or later versions (American Psychiatric Association, 1994), the International Classification of Diseases, 10th Edition (ICD-10) (Sheehan et al., 1998), the Chinese Classification of Mental Disorders, Third Edition (CCMD-Ⅲ) or later versions (Zou et al., 2008) (Herrmann et al., 1998). The enrolled subjects reported greater than 50% reduction in the total score or response rate in either 17 or 24-item Hamilton Depression Scale (HAMD) (Jaracz et al., 2002). The herbal intervention was approval by the National Drug Administration (Robinson et al., 1986). The eligible comparators in studies included: usual care, a placebo, herbal medicine, and any conventional antidepressant medications. The treatment duration and frequency were not included in the inclusion criteria for the meta-analysis.

### 2.3 Exclusion criteria of randomized controlled trials

The clinical trials were excluded from the meta-analysis, if the following conditions were met (Ayerbe et al., 2013). Chinese herbal decoctions or antidepressants combined with non-pharmacological treatments (e.g., electroconvulsive therapy, acupuncture, massage, nursing, and psychotherapy) were used in the trial (Villa et al., 2018). Studies enrolled patients with schizophrenia, eating disorders, or any serious medical illness that prohibited medicine use (Herrmann et al., 1998). Lack of primary outcome data.

### 2.4 Outcomes

The primary outcomes of the meta-analysis included (Ayerbe et al., 2013): response rate (calculated based on the patients whose 17- or 24-item HAMD total score decreased by 50% from baseline to endpoint) and (Villa et al., 2018) mean change in HAMD score from baseline to the end of follow-up.

The secondary outcomes included dropout due to any reason (Supplementary Appendix S3.1), mean change in patients, overall scores on the National Institute of Health stroke scale (NIHSS), which evaluated their neurological deficit, total gastrointestinal events, and total nervous system events (Supplementary Appendix S3.2).

### 2.5 Data analysis

We measured risk ratios (RRs) for binary outcomes such as response rate and mean differences (MDs) (change in HAMD and NIHSS score) for continuous outcomes. The missing standard deviation of change score from baseline was determined by a method from the Cochrane handbook (Supplementary Appendix S3.4).

The frequentist random-effect network meta-analysis with graph-theoretical approach using R package “netmeta” was used for the analysis.

(Rücker, 2012) (Supplementary Appendix S3.1). To estimate the variance for heterogeneity among studies, DerSimonian–Laird random-effects model was conducted. Each drug was represented by each network node in a particular drug class. The direct and indirect comparisons of network estimations for each outcome were revealed using league tables of the relative treatment effects and forest plots. The interventions were then ranked based on the P-score. To evaluate the local and global statistical heterogeneity, the generalized Cochran’s Q was applied. To detect the intransitivity of the network nodes, the following characteristics and settings of the studies were compared: mode of two items from the bias assessment risk (blinding of outcome assessment and incomplete outcome data), mode severity of dementia, mode outcome measure reported, mode study setting (e.g., nursing clinic or home), mode proportion of women (<50% or ≥50%), mean age of patient, and mean of study duration. The local inconsistency of the indirect and direct results for all comparison loops was evaluated by the node-splitting approach. The indirect results obtained from the network and direct results were calculated by the back-calculation method.

### 2.6 Quality assessment

To assess the risk of bias of individual studies for Randomized Trials, the Cochrane Risk-of-Bias Tool (RoB-2) was used by X.C. and J.Z. independently. Another researcher (S.M.) was also employed to resolve the disagreement.

### 2.7 GRADE certainty assessments

To rate the levels of evidence (defined as high, moderate, low, or very low certainty), the GRADE approach was utilized. Following the GRADE assessment for network meta-analysis (GRADE-NMA), we downgraded the evidence based on the seven domains, including imprecision, inconsistency, intransitivity, publication bias, heterogeneity, indirectness, and risk of bias. To draw the results from network meta-analysis, the minimally contextualized framework was conducted by categorizing the interventions into the most effective/harmful, intermediate and least effective/harmful interventions. This framework involved minimal contextualized judgement and adopted no effect as the GRADE assessment decision target. Meanwhile, drugs were categorized based on whether they were significantly better or worse than reference drug or other drugs. We also divided the drugs into two groups (Ayerbe et al., 2013): one group with high or moderate certainty and (Villa et al., 2018) second group with low or very low certainty.

## 3 Results

Of the 1132 citations identified, 264 potentially eligible articles were retrieved. Ultimately, 51 unique randomized control trials fulfilled all the inclusion and exclusion criteria, which included 4,506 adult subjects in total (Supplementary Appendix S4). All 51 studies were conducted in China. Only two articles were published in English (Supplementary Appendix S5).

### 3.1 Studies’ and participants’ characteristics

11 combined interventions and 9 monotherapies were included in these trials. SSRI were the most frequently used comparators across all studies. The median length of the follow-up was ranging from 4 weeks to 3 months. Of the recruited patients, 43.0% were inpatients, 4.0% were outpatients and 53.0% were mix of both inpatients and outpatients (Supplementary Appendix S5.1). Most of the trials (36 of 51, or 70.6%) adopted the CCMD diagnostic criteria.

The median age of the participants was 62.1 years old. Approximately, 46% of them were female. A complete description of the participants and studies are presented in Supplementary Appendix S5.

### 3.2 Assessment of the risk of bias

There were some general concerns or high concerns for the risk of bias for the randomization due to the lack of information regarding the allocation concealment. The risk of bias for the deviation was predominantly high, because of the lack of detailed methods to clearly indicate whether the participants were aware of their assigned intervention during the trail. Forty-nine trials presented with an unclear risk of bias and two trials with a low risk of bias for the measurement of the outcomes. Forty-five out of 51 publications presented with an unclear risk of bias for the selection of the reported results, because there were no advance announcement of the protocol (Supplementary Appendix S6).

### 3.3 Network meta-analysis

**FIGURE 1:**
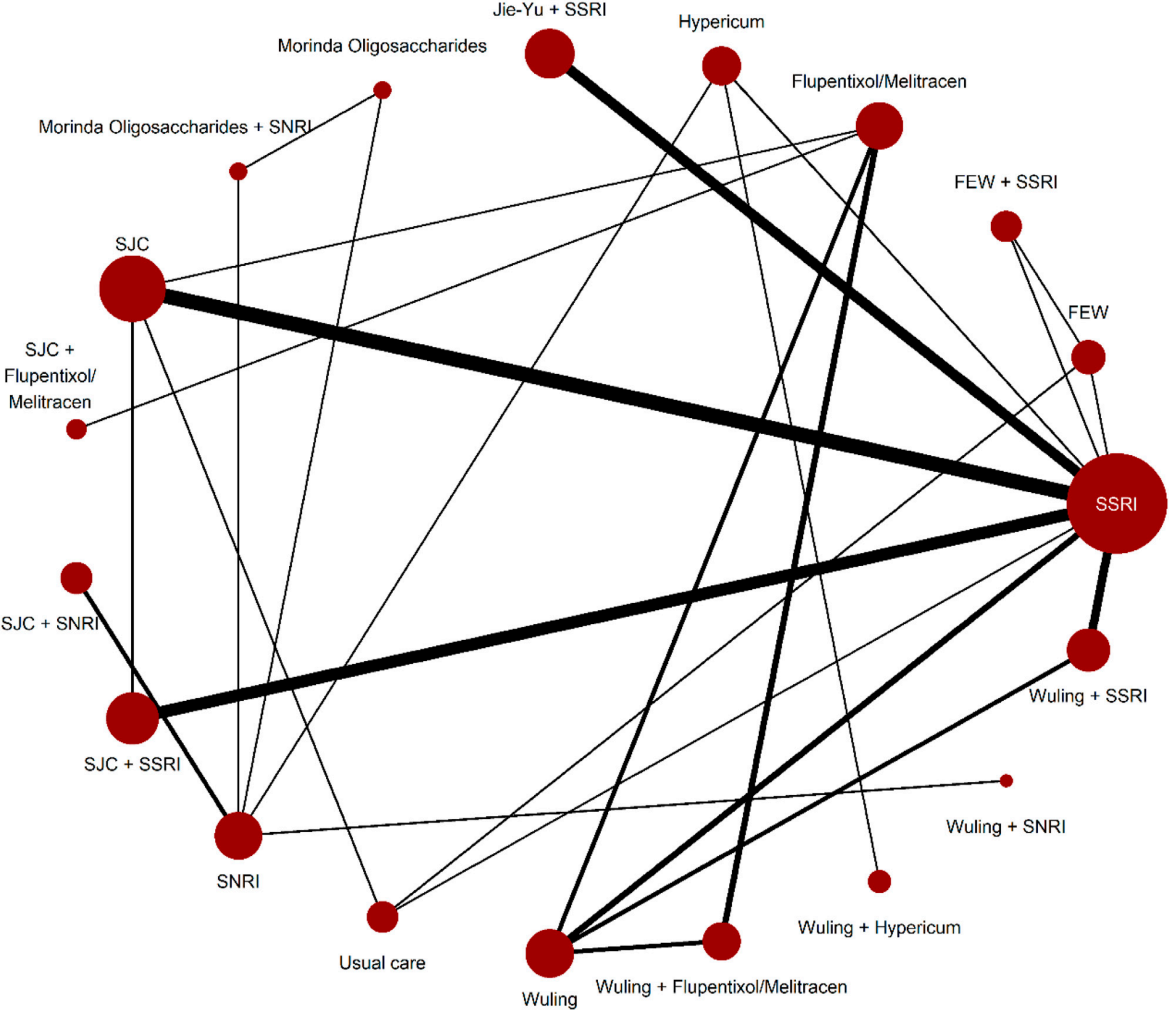
Network plots for response rate.Network plots for the included studies, by drug treatments. Network plots consist of the drug nodes with node size being proportional to the number of randomly assigned participants (i.e., sample size) and the comparison edges with line thickness being proportional to the number of trials comparing every pair of treatments. Abbreviations: FEW = Free and Easy Wanderer. Jie-Yu = Jie-Yu Pills. SNRI = serotonin and noradrenaline reuptake inhibitors. SSRI = selective serotonin reuptake inhibitors. SJC = Shugan Jieyu capsule. Wuling = Wuling capsule.

**FIGURE 2:**
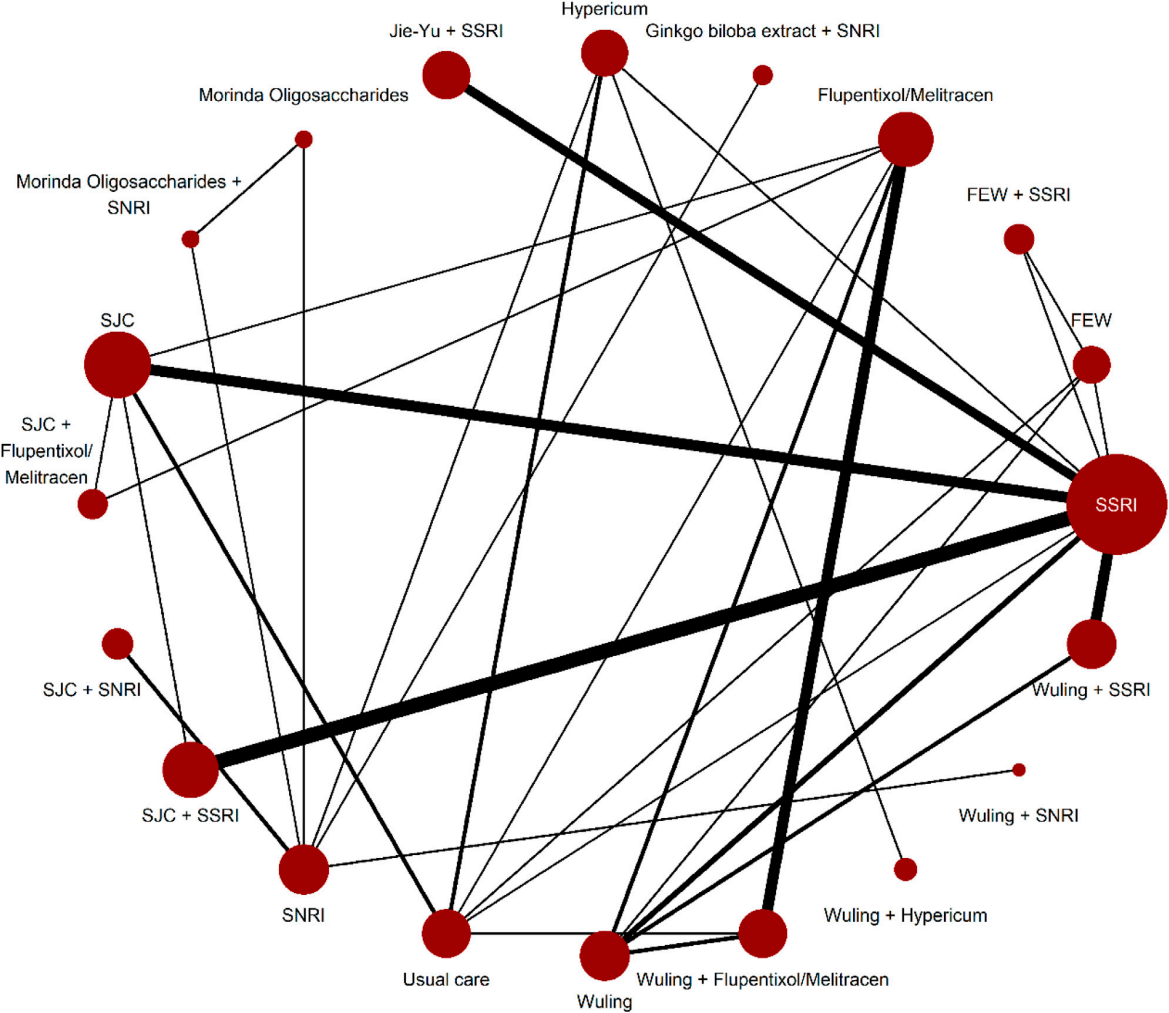
Network plots for mean changes in HAMD score from baseline. Network plots for the included studies, by drug treatments. Network plots consist of the drug nodes with node size being proportional to the number of randomly assigned participants (i.e., sample size) and the comparison edges with line thickness being proportional to the number of trials comparing every pair of treatments. Abbreviations: FEW = Free and Easy Wanderer. Jie-Yu = Jie-Yu Pills. SNRI = serotonin and noradrenaline reuptake inhibitors. SSRI = selective serotonin reuptake inhibitors. SJC = Shugan Jieyu capsule. Wuling = Wuling capsule.

Supplementary Appendix S7.5 shows the evaluation of heterogeneity and inconsistency. The evidence did not suggest global publication bias for any outcome (Supplementary Appendix S7.6), nor did the results suggest relevant global inconsistencies or incoherent outcomes except for the response rate and mean changes in the HAMD score from baseline (Supplementary Appendix S7.5). The network plots depicting the response rate and the change of mean in the HAMD total score are shown in Figure 1, Figure 2. Supplementary Appendix S7.1 presents all other network plots. The network estimates for all comparisons are shown in the league tables (Supplementary Appendix S7.3). Minimal framework context is provided in Supplementary Appendix S7.2. Based on the effect magnitude relative to SSRI alone and the certainty of evidence, Table 1 ranks interventions from best to worst when compared to SSRI for the two key benefits and two key harms. The study regressed the treatment effects of two primary outcomes on the subgroup factor—length of follow-up. We only show the coefficients for the analyses. As a result, we did not find any credible effect (Supplementary Appendix S9). In addition, we conducted subgroup analysis, based on the severity of depression, but we did not identify any credible subgroup effect (Supplementary Appendix S10). Finally, after excluding studies with follow-up of less than 8 weeks, we conducted the sensitivity analyses, which confirmed the robustness. All the results from the analyses of sensitivity were consistent with the primary results (Supplementary Appendix S11).

**TABLE 1:** Summary of the harm and benefit outcomes of the antidepressants.

Interventions	Benefit outcomes		Harm outcomes	
	Response rate	HAMD score	Any gastrointestinal event	Any nervous system event
	RR (95% CI)	MD (95% CI)	RR (95% CI)	RR (95% CI)
**Wuling + Hypericum**	1.65 (0.85, 3.22)	**−10.12 (-17.25, -2.99)**	0.36 (0.00, 42.76)	0.56 (0.01, 27.59)
**SJC + SSRI**	**1.45 (1.23, 1.7)**	**−4.08 (-6.17, -1.99)**	0.91 (0.56, 1.48)	1.16 (0.37, 3.62)
**Wuling + SSRI**	**1.32 (1.09, 1.59)**	**−3.81 (-6.19, -1.42)**	0.89 (0.46, 1.71)	0.47 (0.14, 1.53)
**Jie-Yu + SSRI**	**1.35 (1.09, 1.68)**	**−5.22 (-7.91, -2.53)**	0.83 (0.39, 1.77)	1.17 (0.34, 4.07)
**SJC + Flupentixol/Melitracen**	1.47 (0.87, 2.49)	−1.27 (−6.17, 3.64)	—	—
**SJC + SNRI**	1.48 (0.74, 2.97)	−7.91 (−16.2, 0.39)	1.61 (0.04, 70.71)	0.16 (0.00, 12.94)
**Wuling + SNRI**	1.53 (0.59, 3.97)	−4.68 (−14.03, 4.66)	0.36 (0.00, 42.76)	—
**Wuling + Flupentixol/Melitracen**	1.21 (0.92, 1.61)	−0.88 (−4.44, 2.68)	0.76 (0.03, 18.72)	—
**Morinda Oligosaccharides + SNRI**	1.22 (0.58, 2.57)	−7.16 (−16.58, 2.26)	0.40 (0.00, 50.36)	0.39 (0.00, 49.57)
**FEW + SSRI**	1.18 (0.86, 1.62)	−0.49 (−5.11, 4.14)	0.33 (0.06, 1.81)	0.11 (0.00, 2.95)
**SJC**	1.13 (0.97, 1.31)	−0.65 (−2.76, 1.47)	**0.34 (0.18, 0.62)**	**0.11 (0.03, 0.35)**
**Wuling**	1.04 (0.86, 1.27)	**3.42 (0.75, 6.09)**	0.36 (0.12, 1.02)	0.34 (0.04, 3.19)
**Morinda Oligosaccharides**	0.99 (0.46, 2.13)	5.1 (−4.32, 14.51)	0.36 (0.00, 42.85)	0.2 (0.00, 30.11)
**FEW**	1.02 (0.75, 1.38)	−1.92 (−5.68, 1.84)	0.33 (0.07, 1.59)	0.11 (0.01, 2.02)
**SNRI**	0.99 (0.53, 1.83)	−2.02 (−9.15, 5.1)	1.07 (0.03, 38.77)	0.2 (0.00, 13.43)
**Hypericum**	0.98 (0.62, 1.56)	−0.84 (−4.84, 3.15)	0.52 (0.03, 7.62)	0.57 (0.04, 9.16)
**Flupentixol/Melitracen**	0.96 (0.73, 1.27)	**3.49 (0.2, 6.79)**	—	—
**Usual care**	0.64 (0.3, 1.33)	**5.41 (2.47, 8.34)**	1.00 (0.27, 3.72)	1.10 (0.25, 4.86)
**Ginkgo biloba extract + SNRI**	—	−5.85 (−15.19, 3.48)	1.34 (0.03, 59.68)	0.47 (0.01, 33.79)

	High/Moderate Certainty of Evidence	Low/Very Low Certainty of Evidence	High/Moderate Certainty of Evidence	Low/Very Low Certainty of Evidence
**Among the best**	Better than SSRI	May be better than SSRI	Safer than SSRI	May be safer than SSRI
**Intermediate**	Same as SSRI	May be same as SSRI	Same as SSRI	May be same as SSRI
**Among the worst**	Worse than SSRI	May be worse than SSRI	More harmful than SSRI	May be more harmful than SSRI

The interventions were categorized into three groups, i.e., among the best/intermediate/among the worst, according to the minimally contextualized framework regarding whether the intervention was significantly better than the SSRI.

The bold text represents statistical significance.

Abbreviations: FEW = Free and Easy Wanderer. Jie-Yu = Jie-Yu Pills. SNRI = serotonin and noradrenaline reuptake inhibitors. SSRI = selective serotonin reuptake inhibitors. SJC = Shugan Jieyu capsule. Wuling = Wuling capsule.

The response rate was described by 38 RCTs (3,544 patients, 19 interventions/nodes). SJC plus SSRI, Jie-Yu Pills plus SSRI, and Wuling capsule plus SSRI proved to be among the most effective with moderate certainty of evidence (RR: 1.45, 95%CI: 1.23 to 1.7; RR: 1.35, 95%CI: 1.09 to 1.68; RR: 1.32, 95%CI: 1.09–1.59). Wuling capsule plus Hypericum and Free and Easy Wanderer (FEW) proved an intermediate effect with moderate certainty and showed the same effect as SSRI (RR: 1.65, 95%CI: 0.85 to 3.22; RR: 1.02, 95%CI: 0.75–1.38). Other drugs showed low or very low certainty of evidence (Table 1).

In the analyses of mean changes in HAMD score after treatment completion, 50 trials and 4,427 participants were included. Wuling capsule plus Hypericum and Wuling capsule plus SSRI were found to be among the most efficacious in reducing symptoms of depression with a moderate certainty of evidence (MD: -10.12, 95%CI: -17.25 to −2.99; MD: -3.81, 95%CI: -6.19 to −1.42). FEW proved intermediate effect with moderate certainty of evidence and showed the same effect as SSRI (MD: -1.92, 95%CI: -5.68 to 1.84). Other drugs showed low or very low certainty of evidence (Table 1).

Five trials reported drop out events due to any reason with 486 participants. No significant differences were found in the effect of these tested interventions, including Hypericum, SJC plus SSRI, Wuling capsule plus SSRI, usual care, SJC, Wuling capsule, and Free and Easy Wanderer, in comparison to SSRI with moderate to low certainty of evidence (Supplementary Appendix S7.4; Supplementary Appendix S8).

For the mean changes of the NIHSS scale score (12 RCTs, comprising 1,125 patients), Jie-Yu Pills plus SSRI (MD: -3.61, 95%CI: -4.69 to −2.52), SJC plus SSRI (MD: -2.66, 95%CI: -3.52 to −1.80), and Wuling capsule plus SSRI (MD: -1.59, 95%CI: -2.16 to −1.03) were superior to SSRI. Wuling capsule plus Flupentixol/Melitracen, SJC plus SNRI, Ginkgo biloba extract plus SNRI, SJC, Wuling capsule plus SNRI, usual care, and Flupentixol/Melitracen were comparable to placebo (Supplementary Appendix S7.4; Supplementary Appendix S8).

The NMA for the total gastrointestinal events consisted of 25 RCTs (2,178 patients, 17 interventions), and SJC were the only intervention that may be safer than SSRI with moderate certainty of evidence (RR: 0.34, 95%CI: 0.18, 0.62). FEW and standard care demonstrated intermediate safety with certainty of evidence and showed the same safety as SSRI (RR: 0.33, 95%CI: 0.07 to 1.59; RR: 1, 95%CI: 0.27–3.72). Other drugs had low or very low certainty of evidence (Table 1).

For the total evens of nervous system, 24 RCTs (2,104 patients, 16 interventions) were included. Additionally, SJC was the only intervention that may be safer than SSRI with moderate certainty of evidence (RR: 0.11, 95%CI: 0.03, 0.35). FEW and usual care still proved intermediate safe with moderate certainty of evidence and showed the same safe as SSRI (RR: 0.11, 95%CI: 0.01 to 2.02; RR: 1.11, 95%CI: 0.25–4.86). Other drugs showed low or very low certainty of evidence (Table 1).

## 4 Discussion

In the present study, the network meta-analysis was conducted for 51 randomized controlled trials that enrolled a total of 4,507 patients assigned to 20 different herbal medicines and first-line antidepressant drugs. We found that SJC plus SSRI, Jie-Yu Pills plus SSRI, and Wuling capsule plus SSRI are among the most effective agents for treating PSD patients. The three interventions mentioned above markedly reduced the HAMD score by more than 50% with a moderate (Wuling capsule plus SSRI) or a low (SJC plus SSRI and Jie-Yu Pills plus SSRI) certainty of evidence. Interestingly, Wuling capsule plus Hypericum and FEW was statistically not inferior to SSRI and was demonstrated to be among the most effective intervention to significantly improve the HAMD score. All the aforementioned treatments showed no additional risks of gastrointestinal and nervous system events with low to very low certainty of evidence. In particular, SJC had a significantly lower safety risk compared to SSRI.

The mechanism of action for these treatments are largely unknown. However, some links between the Chinese herbal medicines highlighted in this study and PSD can be discerned. Several evidence suggest that the underlying mechanisms of PSD include decreased neurotrophic factors such as Insulin-like growth factor 1 (IGF-1), Brain-derived neurotrophic factor (BDNF)) and monoamines (5-hydroxy tryptamine(5-HT), dopamine (DA) and norepinephrine (NE)), increased inflammatory factors, glutamate-mediated excitotoxicity, and disruption of the axis regulated by hypothalamic-pituitary-adrenal (HPA) (Guo et al., 2022). Wuling capsule can upregulate PI3K, p-Akt, and p-mTOR expression at protein level, and upregulate the NE, DA, and 5-HT content of hippocampus tissue, and increase neurotransmitter levels to improve the behavior of rats with PSD (Sun et al., 2021). Furthermore, improved response rate and cognitive deficits of Wuling capsule in PSD rat models, independent of BDNF, have been observed (Li et al., 2011). In addition, mitochondrial dysfunction is increasingly associated with depression by promoting the translocator protein (TSPO) mediated mitophagy signaling pathway. Wuling powder prevents the depression-like behavior in learned helplessness mice model through improving the TSPO mediated mitophagy (Li et al., 2016). Lastly, Wuling mycelia powder, one major component of Wuling capsule, has an antidepressant-like effect on the rats with chronic unpredictable mild stress (CUMS), and the signaling pathway mediated by l-arginine-nitric oxide (NO)-cyclic guanosine monophosphate (cGMP) is significantly involved in this antidepressant effect. After administration with Wuling mycelia (at 1 and 2 g/kg) for 6 weeks, the CUMS rats increased sucrose preference, crossing numbers in open-field test and food consumption. These antidepressant-like effects were demonstrated to be further enhanced by methylene blue and 7-nitroindazole pretreatment, and inhibited by sildenafil (a.k.a. Viagra) and l-arginine pretreatment (Tan et al., 2016).

SJC is a Chinese combined extract of Eleutherococcus senticosus (ES) and *Hypericum perforatum* (HP). SJC might improve the cognition of mild to moderate depression (MMD) patients, which may be realized partly through the regulation within two brain regions, ventral caudate (vCa) and orbitofrontal cortex (OFC) (Liu et al., 2020). SJC can significantly improve the depression-like symptoms and promote the repair and/or regeneration of nerve cell damage in the hippocampal CA3 area by reducing the caspase-3 protein expression and preventing neuronal apopotosis in a rat depression model (Fu et al., 2012; Fu et al., 2014). The antidepression-like effect of SJC was shown to be similar to a SSRI, fluoxetine (Fu et al., 2012; Fu et al., 2014).

The primary active antidepressant phytochemical is hyperforin for *Hypericum perforatum* (a.k.a., St. John’s wort or hypericum). (Ng et al., 2017). Hyperforin has been shown to improve synaptic plasticity *via* different pathways, including CaM-kinase IV (CaMKIV), PI3K/Akt, and Ras/MEK/ERK, enhancing the phosphorylation of cyclic adenosine monophosphate response element binding protein, associated with depression in primary hippocampal neurons and PC12 cells (Heiser et al., 2013).

Unlike the other herbal medicines above mentioned, Jie-Yu Pills is composed of Ganmaidazao (GMDZD) decoction and FEW (a.k.a., Xiao Yao San). It has been found that Jie-Yu Pills can reverse neuro-endocrine-immune dysfunction and exert antidepressant-like effect by reducing the excessive concentration of corticosterone (CORT), adrenocorticotropic hormone (ACTH), tumor necrosis factor-α (TNF-α) and interleukin-1β (IL-1β) in a rat model of unpredictable stress (Shi et al., 2007a; Shi et al., 2007b). The depression-like behavior can be significantly reduced by GMDZD through subchronic administration in rats, and this effect is contributed by the elevated NMDAR subunits (NR2A and NR2B) expression and reduced glutamate in the hippocampus and frontal cortex (Lou et al., 2010; El-Alfy et al., 2012). Furthermore, FEW, disassembled prescriptions of Jie-Yu Pills, can significantly attenuate the depression-like behavior by regulating the hippocampus synaptic plasticity and ameliorating the ultrastructrural damage to the CA1 region of hippocampus after immobilization stress in rats (Liang et al., 2013). In addition, FEW has the potential to activate cerebral 5-HT1A-receptor and suppress the activity of locus coeruleus NE neuron (Yin et al., 2012), which are targets of depressive symptoms. Similarly, FEW has been demonstrated to reduce the levels of NE, corticotropin-releasing factor (CRF) and tyrosine hydroxylase, were also discovered after the treatment of FEW in serum in locus coeruleus in a rat model of depression (Ding et al., 2014). Furthermore, by suppressing urocortin 2 and corticosterone (CORT), FEW also can markedly impair the hyperactivation of HPA. The reduction of serine/threonine protein phosphatase 2A (PP2A) regulatory subunit B and elecation of tyrosine receptor kinase B, mechanistic target of rapamycin (mTOR), BDNF, and β-arrestin 2 were also found after FEW treatment (Zhu et al., 2014).

To summarize, to our knowledge, this is the first study to systematically examine the efficacy of herbal medicine alone or in combination with classic antidepressants (SSRI, SNRI) to attenuate the depressive symptoms in PSD adults. Previous reviews on the efficacy of traditional Chinese medicine for PSD have mainly focused on Wuling capsule and have concluded that it may provide some benefits when combined with SSRI, such as sertraline, paroxetine and citalopram (Peng et al., 2014). We performed a systematic network meta-analysis to explore and to clarify the efficacy of single drugs and drug combinations. In order to do so, our study utilized the GRADE minimally contextualized framework to group several interventions that allowed us to facilitate the clinical interpretations and to draw conclusions.

It is worthy to note, however, that the current study does come with certain limitations. First, the conclusions drawn in the current study regarding the effectiveness of traditional Chinese medicines are based on a meta-analysis, which means that future clinical studies will need to be performed to confirm the efficacy of the treatments showing evidence of efficacy for PSD in the current study. Second, the main focus of our study was on the efficacy of the herbal medicines and the combination of these herbal medicines with classic antidepressants. Therefore, in-depths analyses of the standard non-herbal antidepressants and combinations of these non-herbal antidepressants are out of scope of this current study. Third, a few studies reported drop outs, the impact of which could not be fully evaluated on the final results of such studies. Future research should focus on long-term acceptability (treatment discontinuation measured by the proportion of patients who withdrew for any reason) of treatments which significantly connected to the highest remission rates and response in the PSD patients. Last but not least, it should be noted that majority of our findings indicated low to moderate certainty of evidence. Therefore, the conclusions that, for example, some of the herbal medicines can provide superior antidepression effect compared to the classic antidepressant alone should be taken with a caution.

## 5 Conclusion

Post-stroke depression is a serious condition that affects one-third of stroke patients every year, yet an effective treatment with minimal adverse effects is currently limited. To our knowledge, the current study is the first study to systematically examine the efficacy of traditional Chinese medicine alone or in combination with the primary treatment of PSD, such as SSRI and SSRI for treating the depression symptoms in PSD patients. We conclude that Wuling capsule, in particular, and other SSRI combinations, such as SSRI plus Wuling capsule, SJC, or Jie-Yu pills, hold a great promise for treating the depression symptoms in PSD patients. Future clinical research is needed to directly confirm these findings and to advance the current treatment strategy for PSD.

## Data Availability

The original contributions presented in the study are included in the article/Supplementary Material, further inquiries can be directed to the corresponding author.
