# Supplementary material for: Herbal medicine as adjunctive therapy with antidepressants for post-stroke depression: a systematic review and network meta-analysis of randomized controlled trials
Source: Front Pharmacol. 2023 Jul 14;14:1180071. doi: 10.3389/fphar.2023.1180071 (PMC10382276; doi:10.3389/fphar.2023.1180071)
Supplement: Supplementary file 1 [file DataSheet1.docx]

**Supplementary appendix**

**Supplement to:**

**Herbal medicine as adjunctive therapy with antidepressants for post-stroke depression: a systematic review and network meta-analysis of randomized controlled trials**

**Jian Zhang, Shuping Ming, Xiaoming Chen, Teng Zhang, Hongyu Qian, Shixiong Peng, Yanbing Ding**

**Table of contents**

[Appendix 1: PRISMA Checklist 4](#_Toc136038819)

[Appendix 2: Search strategy 8](#_Toc136038820)

[Appendix 3: Supplementary of methods 20](#_Toc136038821)

[3.1 Changes from protocol 20](#_Toc136038822)

[3.2 Definition of outcomes 22](#_Toc136038823)

[3.3 Methods for estimation of missing mean changes in HAMD score from baseline 23](#_Toc136038824)

[3.4 Methods for estimation of standard deviation of change score from baseline 24](#_Toc136038825)

[Appendix 4: PRISMA flow diagram 25](#_Toc136038826)

[Appendix 5: Characteristics of included studies 26](#_Toc136038827)

[5.1 Summary of characteristics of included studies 26](#_Toc136038828)

[5.2 List of included studies 27](#_Toc136038829)

[5.3 List of included patients 33](#_Toc136038830)

[Appendix 6: Risk of bias assessments 44](#_Toc136038831)

[6.1 Outcome: Response rate 44](#_Toc136038832)

[6.2 Outcome: Mean changes in HAMD score from baseline 45](#_Toc136038833)

[6.3 Outcome: All-cause drop out 46](#_Toc136038834)

[6.4 Outcome: Any gastrointestinal event 47](#_Toc136038835)

[6.5 Outcome: Any nervous system event 48](#_Toc136038836)

[Appendix 7: Other main results 49](#_Toc136038837)

[7.1 Network plots 49](#_Toc136038838)

[7.2 Minimally contextualized framework 51](#_Toc136038839)

[7.3 Network estimates (league tables) 58](#_Toc136038840)

[7.4 Forest plots 64](#_Toc136038841)

[7.5 Heterogeneity and inconsistency (incoherence) 70](#_Toc136038842)

[7.6 Comparison-adjusted funnel plots 79](#_Toc136038843)

[Appendix 8: GRADE assessments 84](#_Toc136038844)

[8.1 Certainty of evidence for direct, indirect and network estimates 84](#_Toc136038845)

[8.2 Absolute contribution matrices 124](#_Toc136038846)

[8.3 Percentage contribution matrices 129](#_Toc136038847)

[Appendix 9: Results of regression analyses 135](#_Toc136038848)

[Appendix 10: Results of subgroup analyses 137](#_Toc136038849)

[Appendix 11: Results of sensitivity analyses 139](#_Toc136038850)

[Appendix 12: Reference list of included studies 150](#_Toc136038851)

Section and Topic

# Appendix 1: PRISMA Checklist

| Section and Topic | Item  # | Checklist item | Location where item is reported |
| --- | --- | --- | --- |
| **TITLE** | | | |
| Title | 1 | Identify the report as a systematic review. | Page 1 |
| **ABSTRACT** | | | |
| Abstract | 2 | See the PRISMA 2020 for Abstracts checklist | Page 2 |
| **INTRODUCTION** | | | |
| Rationale | 3 | Describe the rationale for the review in the context of existing knowledge. | Page 5 |
| Objectives | 4 | Provide an explicit statement of the objective(s) or question(s) the review addresses. | Page 5 |
| **METHODS** | | | |
| Eligibility criteria | 5 | Specify the inclusion and exclusion criteria for the review and how studies were grouped for the syntheses. | Page 8 |
| Information sources | 6 | Specify all databases, registers, websites, organizations, reference lists and other sources searched or consulted to identify studies. Specify the date when each source was last searched or consulted. | Page 7 |
| Search strategy | 7 | Present the full search strategies for all databases, registers and websites, including any filters and limits used | Page 7; Appendix 2 |
| Selection process | 8 | Specify the methods used to decide whether a study met the inclusion criteria of the review, including how many reviewers screened each record and each report retrieved, whether they worked independently, and if applicable, details of automation tools used in the process. | Page 7 |
| Data collection process | 9 | Specify the methods used to collect data from reports, including how many reviewers collected data from each report, whether they worked independently, any processes for obtaining or confirming data from study investigators, and if applicable, details of automation tools used in the process. | Page 7 |
| Data items | 10a | List and define all outcomes for which data were sought. Specify whether all results that were compatible with each outcome domain in each study were sought (e.g., for all measures, time points, analyses), and if not, the methods used to decide which results to collect. | Page 9 |
|  | 10b | List and define all other variables for which data were sought (e.g., participant and intervention characteristics, funding sources). Describe any assumptions made about any missing or unclear information. | Page 9; Appendix 5 |
| Study risk of bias assessment | 11 | Specify the methods used to assess risk of bias in the included studies, including details of the tool(s) used, how many reviewers assessed each study and whether they worked independently, and if applicable, details of automation tools used in the process. | Page 11 |
| Effect measures | 12 | Specify for each outcome the effect measure(s) (e.g., risk ratio, mean difference) used in the synthesis or presentation of results. | Page 9-10 |
| Synthesis methods | 13a | Describe the processes used to decide which studies were eligible for each synthesis (e.g., tabulating the study intervention characteristics and comparing against the planned groups for each synthesis (item #5)) | Page 10 |
|  | 13b | Describe any methods required to prepare the data for presentation or synthesis, such as handling of missing summary statistics, or data conversions. | Page 10 |
|  | 13c | Describe any methods used to tabulate or visually display results of individual studies and syntheses. | Page 10 |
|  | 13d | Describe any methods used to synthesize results and provide a rationale for the choice(s). If meta-analysis was performed, describe the model(s), method(s) to identify the presence and extent of statistical heterogeneity, and software package(s) used. | Page 10 |
|  | 13e | Describe any methods used to explore possible causes of heterogeneity among study results (e.g. subgroup analysis, meta-regression). | Page 10 |
|  | 13f | Describe any sensitivity analyses conducted to assess robustness of the synthesized results. | Page 10 |
| Reporting bias assessment | 14 | Describe any methods used to assess risk of bias due to missing results in a synthesis (arising from reporting biases). | Page 10 |
| Certainty assessment | 15 | Describe any methods used to assess certainty (or confidence) in the body of evidence for an outcome. | Page 10 |
| **RESULTS** | | | |
| Study selection | 16a | Describe the results of the search and selection process, from the number of records identified in the search to the number of studies included in the review, ideally using a flow diagram. | Page 12;  Appendix 4 |
|  | 16b | Cite studies that might appear to meet the inclusion criteria, but which were excluded, and explain why they were excluded. | Appendix 4 |
| Study characteristics | 17 | Cite each included study and present its characteristics. | Appendix 5 |
| Risk of bias in studies | 18 | Present assessments of risk of bias for each included study. | Appendix 6 |
| Results of individual studies | 19 | For all outcomes, present, for each study: (a) summary statistics for each group (where appropriate) and (b) an effect estimates and its precision (e.g., confidence/credible interval), ideally using structured tables or plots. | Appendix 7 |
| Results of syntheses | 20a | For each synthesis, briefly summarize the characteristics and risk of bias among contributing studies. | Page 11-12 |
|  | 20b | Present results of all statistical syntheses conducted. If meta-analysis was done, present for each the summary estimate and its precision (e.g., confidence/credible interval) and measures of statistical heterogeneity. If comparing groups, describe the direction of the effect. | Appendix 7 |
|  | 20c | Present results of all investigations of possible causes of heterogeneity among study results. | Appendix 7.6 |
|  | 20d | Present results of all sensitivity analyses conducted to assess the robustness of the synthesized results. | Appendix 11 |
| Reporting biases | 21 | Present assessments of risk of bias due to missing results (arising from reporting biases) for each synthesis assessed. | Page 12 |
| Certainty of evidence | 22 | Present assessments of certainty (or confidence) in the body of evidence for each outcome assessed. | Appendix 8 |
| **DISCUSSION** | | | |
| Discussion | 23a | Provide a general interpretation of the results in the context of other evidence. | Page 22 |
|  | 23b | Discuss any limitations of the evidence included in the review. | Page 22 |
|  | 23c | Discuss implications of the results for practice, policy, and future research. | Page 22 |
| **OTHER INFORMATION** | | | |
| Registration and protocol | 24a | Provide registration information for the review, including register name and registration number, or state that the review was not registered. | Page 7 |
|  | 24b | Indicate where the review protocol can be accessed, or state that a protocol was not prepared. | Page 7 |
|  | 24c | Describe and explain any amendments to information provided at registration or in the protocol. | Appendix 3 |
| Support | 25 | Describe sources of financial or non-financial support for the review, and the role of the funders or sponsors in the review. | Page 4 |
| Competing interests | 26 | Declare any competing interests of review authors. | Page 28 |
| Availability of data, code and other materials | 27 | Report which of the following are publicly available and where they can be found: template data collection forms; data extracted from included studies; data used for all analyses; analytic code; any other materials used in the review. | Page 28 |

# Appendix 2: Search strategy

**PubMed (via OVID, Search date: August 17, 2021)**

| 1 | exp Herbal medicine/ | 2287 |
| --- | --- | --- |
| 2 | exp Phytotherapy/ | 40509 |
| 3 | exp Plant Extracts/ | 182799 |
| 4 | exp Plants, Medicinal/ | 60417 |
| 5 | "phytotherapy".ti,ab. | 1378 |
| 6 | "plant extrac*".ti,ab. | 11509 |
| 7 | exp Medicine, Chinese Traditional/ | 20916 |
| 8 | exp Drugs, Chinese Herbal/ | 46877 |
| 9 | "Chinese patent medicine".ti,ab. | 470 |
| 10 | "Chinese Drugs".ti,ab. | 411 |
| 11 | "Chinese materia medica".ti,ab. | 558 |
| 12 | "Chinese herbal medicine".ti,ab. | 4186 |
| 13 | exp Lavandula/ | 540 |
| 14 | "lavender".ti,ab. | 1322 |
| 15 | exp Passiflora/ | 542 |
| 16 | "passion flower".ti,ab. | 55 |
| 17 | exp Crocus/ | 802 |
| 18 | "Crocus sativus".ti,ab. | 855 |
| 19 | "Saffron".ti,ab. | 1477 |
| 20 | exp Borago/ | 67 |
| 21 | "Borage".ti,ab. | 318 |
| 22 | exp Panax/ | 6082 |
| 23 | "Korean ginseng".ti,ab. | 217 |
| 24 | exp Mimosa/ | 292 |
| 25 | "Albizia julibrissin".ti,ab. | 62 |
| 26 | exp Rhodiola/ | 600 |
| 27 | "Roseroot".ti,ab. | 25 |
| 28 | exp Hypericum/ | 2312 |
| 29 | "St John's wort".ti,ab. | 1791 |
| 30 | "perforatum".ti,ab. | 1498 |
| 31 | "St. John's Wort Extract".ti,ab. | 134 |
| 32 | "St. John's Wort Extract Tablets".ti,ab. | 1 |
| 33 | exp Ginkgo biloba/ | 2942 |
| 34 | "Ginkgo leaves".ti,ab. | 82 |
| 35 | exp Eleutherococcus/ | 519 |
| 36 | "Acanthopanax".ti,ab. | 477 |
| 37 | "Wuling Capsul*".ti,ab. | 14 |
| 38 | "Shugan Jieyu Capsul*".ti,ab. | 8 |
| 39 | "Jieyu pill*".ti,ab. | 5 |
| 40 | "Xiaoyao pill*".ti,ab. | 29 |
| 41 | exp Morinda/ | 503 |
| 42 | "Morinda officinalis oligosaccharides ".ti,ab. | 3 |
| 43 | "PSD".ti,ab. | 8303 |
| 44 | "Post-stroke depression".ti,ab. | 896 |
| 45 | "post-stroke depressive".ti,ab. | 50 |
| 46 | "depression after stroke".ti,ab. | 207 |
| 47 | "depression in stroke patients".ti,ab. | 55 |
| 48 | "Depression after cerebral infarction".ti,ab. | 1 |
| 49 | "depression after cerebral apoplexy".ti,ab. | 0 |
| 50 | "depression after cerebrovascular acciden".ti,ab. | 0 |
| 51 | "depression after cerebrovascular disease".ti,ab. | 0 |
| 52 | "Depression after cerebral hemorrhage".ti,ab. | 0 |
| 53 | 1 or 2 or 3 or 4 or 5 or 6 or 7 or 8 or 9 or 10 or 11 or 12 or 13 or 14 or 15 or 16 or 17 or 18 or 19 or 20 or 21 or 22 or 23 or 24 or 25 or 26 or 27 or 28 or 29 or 30 or 31 or 32 or 33 or 34 or 35 or 36 or 37 or 38 or 39 or 40 or 41 or 42 | 262655 |
| 54 | 43 or 44 or 45 or 46 or 47 or 48 or 49 or 50 or 51 or 52 | 8924 |
| 55 | 53 and 54 | 109 |

**Embase (Search date: August 18, 2021)**

| #1 | 'herbal medicine'/exp |
| --- | --- |
| #2 | 'phytotherapy'/exp |
| #3 | Phytotherapy |
| #4 | 'plant extract'/exp |
| #5 | "plant extrac*".ti,ab. |
| #6 | 'medicinal plant'/exp |
| #7 | 'Chinese medicine'/exp |
| #8 | 'Chinese drugs':ti,ab,kw |
| #9 | 'Chinese patent medicine':ti,ab,kw |
| #10 | 'Chinese materia medica':ti,ab,kw |
| #11 | 'Chinese herbal medicine':ti,ab,kw |
| #12 | #1 OR #2 OR #3 OR #4 OR #5 OR #6 OR #7 OR #8OR #9 OR #10 OR #11 |
| #13 | 'lavender'/exp |
| #14 | 'passiflora'/exp |
| #15 | "passion flower".ti,ab. |
| #16 | 'crocus'/exp |
| #17 | "Crocus sativus".ti,ab. |
| #18 | "Saffron".ti,ab. |
| #19 | 'borago'/exp |
| #20 | "Borage".ti,ab. |
| #21 | 'panax'/exp |
| #22 | "Korean ginseng".ti,ab. |
| #23 | 'mimosa'/exp |
| #24 | "Albizia julibrissin".ti,ab. |
| #25 | "Roseroot".ti,ab. |
| #26 | 'hypericum perforatum extract'/exp |
| #27 | "St John's wort".ti,ab. |
| #28 | "perforatum".ti,ab. |
| #29 | 'st. johns wort extract':ti,ab,kw |
| #30 | 'st. johns wort extract tablets':ti,ab,kw |
| #31 | 'ginkgo biloba'/exp |
| #32 | 'eleutherococcus'/exp |
| #33 | 'eleutherococcus extract'/exp |
| #34 | 'acanthopanax'/exp |
| #35 | 'wuling capsul*':ti,ab,kw |
| #36 | 'shugan jieyu capsul*':ti,ab,kw |
| #37 | 'jieyu pill*':ti,ab,kw |
| #38 | 'xiaoyao pill*':ti,ab,kw |
| #39 | 'morinda'/exp |
| #40 | 'xinnaoxin capsules':ti,ab,kw |
| #41 | #13 OR #14 OR #15OR #16 OR #17 OR #18 OR #19 OR #20 OR #21 OR #22 OR #23 OR #24 OR #25 OR #26 OR #27 OR #28OR #29 OR #30 OR #31OR #32 OR #33 OR #34 OR #35 OR #36 OR #37 OR #38OR #39 OR #40 |
| #42 | 'post-stroke depression'/exp |
| #43 | 'depression after stroke':ti,ab,kw |
| #44 | 'depression in stroke patients':ti,ab,kw |
| #45 | 'depression after cerebral infarction':ti,ab,kw |
| #46 | 'depression after cerebral apoplexy':ti,ab,kw |
| #47 | 'depression after cerebrovascular acciden':ti,ab,kw |
| #48 | 'depression after cerebrovascular disease':ti,ab,kw |
| #49 | 'depression after cerebral hemorrhage':ti,ab,kw |
| #50 | #42OR #43 OR #44 OR #45OR #46OR #47OR #48 OR #49OR #50 |
| #51 | (#12) AND (#41) AND (#50) |

**OVID-Cochrane Central Register of Controlled Trials** **(Search date: August 18, 2021)**

| #1 | exp Herbal medicine/ |
| --- | --- |
| #2 | exp Phytotherapy/ |
| #3 | exp Plant Extracts/ |
| #4 | exp Plants, Medicinal/ |
| #5 | "phytotherapy".ti,ab. |
| #6 | "plant extrac*".ti,ab. |
| #7 | exp Medicine, Chinese Traditional/ |
| #8 | exp Drugs, Chinese Herbal/ |
| #9 | "Chinese patent medicine".ti,ab. |
| #10 | "Chinese Drugs".ti,ab. |
| #11 | "Chinese materia medica".ti,ab. |
| #12 | "Chinese herbal medicine".ti,ab. |
| #13 | exp Lavandula/ |
| #14 | “lavender".ti,ab. |
| #15 | exp Passiflora/ |
| #16 | “passion flower”.ti,ab. |
| #17 | exp Crocus/ |
| #18 | "Crocus sativus".ti,ab. |
| #19 | "Saffron".ti,ab. |
| #20 | exp Borago/ |
| #21 | "Borage".ti,ab. |
| #22 | exp Panax/ |
| #23 | "Korean ginseng".ti,ab. |
| #24 | exp Mimosa/ |
| #25 | "Albizia julibrissin".ti,ab. |
| #26 | exp Rhodiola/ |
| #27 | "Roseroot".ti,ab. |
| #28 | exp Hypericum/ |
| #29 | "St John's wort".ti,ab. |
| #30 | "perforatum".ti,ab. |
| #31 | "St. John's Wort Extract".ti,ab |
| #32 | "St. John's Wort Extract Tablets".ti,ab |
| #33 | exp Ginkgo biloba/ |
| #34 | "Ginkgo leaves".ti,ab |
| #35 | exp Eleutherococcus/ |
| #36 | "Acanthopanax".ti,ab |
| #37 | "Wuling Capsul*".ti,ab |
| #38 | "Shugan Jieyu Capsul*".ti,ab. |
| #39 | "Jieyu pill*".ti,ab |
| #40 | "Xiaoyao pill*".ti,ab. |
| #41 | exp Morinda/ |
| #42 | "Morinda officinalis oligosaccharides ".ti,ab |
| #43 | OR/1-42 |
| #44 | "PSD".ti,ab. |
| #45 | "Post-stroke depression".ti,ab. |
| #46 | "post-stroke depressive".ti,ab. |
| #47 | "depression after stroke".ti,ab. |
| #48 | "depression in stroke patients".ti,ab. |
| #49 | "Depression after cerebral infarction".ti,ab. |
| #50 | "depression after cerebral apoplexy".ti,ab. |
| #51 | "depression after cerebrovascular acciden".ti,ab. |
| #52 | "depression after cerebrovascular disease".ti,ab. |
| #53 | "Depression after cerebral hemorrhage".ti,ab. |
| #54 | OR/44-54 |
| #55 | (#43) AND (#54) |

# Appendix 3: Supplementary of methods

## 3.1 Changes from protocol

**We initially planned a Bayesian analysis but during conduct of the study identified innovative methods for rating evidence quality that require a frequentist analysis; we therefore changed our analytic approach. The Bayesian analyses were used to duplicate all frequentist estimates. This change aimed to be in line with the GRADE assessments recommended by Nikolakopoulou et al (2020). We calculated the contribution matrices for all network comparisons derived from direct comparisons. The absolute contribution matrix was the projection matrix from frequentist network meta-analysis (Papakonstantinou et al., 2018). The proportion contribution matrix was derived from the absolute contribution matrix (König et al., 2013). This contribution matrix was used to assess GRADE certainty of evidence for indirect comparisons and network estimates.**

**We added all cause dropout which was not anticipated by the protocol as the secondary outcome.**

Reference: Nikolakopoulou, A., Higgins, J., Papakonstantinou, T., Chaimani, A., Del Giovane, C., Egger, M., & Salanti, G. (2020). CINeMA: An approach for assessing confidence in the results of a network meta-analysis. PLoS medicine, 17(4), e1003082. https://doi.org/10.1371/journal.pmed.1003082 König, J., Krahn, U., & Binder, H. (2013). Visualizing the flow of evidence in network meta-analysis and characterizing mixed treatment comparisons.

Statistics in medicine, 32(30), 5414–5429. https://doi.org/10.1002/sim.6001 Papakonstantinou, T., Nikolakopoulou, A., Rücker, G., Chaimani, A., Schwarzer, G., Egger, M., & Salanti, G. (2018). Estimating the contribution of studies in network meta-analysis: paths, flows and streams. F1000Research, 7, 610. https://doi.org/10.12688/f1000research.14770.3

## 3.2 Definition of outcomes

| **Outcomes** | **Definition/Description** | **Property** |
| --- | --- | --- |
| **Response rate** | Response rate measured by the total number of patients who had a decrease of ≥50% of 17 or 24-item HAMD total score from baseline to endpoint | Binary variable/ objective outcome |
| **Mean changes in HAMD score from baseline** | Mean changes in HAMD score of patients from baseline to end of follow-up | Continuous variable/ objective outcome |
| **All-cause drop out** | Number of patients who withdrew from the trial due to various events leading to drug discontinuation. | Binary variable/ objective outcome |
| **NIHSS** | The mean changes in the National Institute of Health stroke scale (NIHSS) total score of patients evaluating their neurological deficit | Continuous variable/ objective outcome |
| **Any gastrointestinal event** | Number of patients who suffered at least one severe gastrointestinal event or gastrointestinal event leading to discontinuation. | Binary variable/ objective outcome |
| **Any nervous system events** | Number of patients who suffered at least one severe nervous system event or nervous system event leading to discontinuation. | Binary variable/ objective outcome |

## 3.3 Methods for estimation of missing mean changes in HAMD score from baseline

We followed the method recommended by Cochrane handbook to estimate the missing mean changes in HAMD score from baseline (Higgins et al., 2021). The point estimate was obtained by subtracting the follow-up end HAMD score from the baseline HAMD score. The variance was estimated using the baseline and follow-up HAMD score, their standard deviations and the correlation between the baseline and follow-up end HAMD score. The correlation was obtained from the studies that reported the absolute HAMD score change and baseline and follow-up end HAMD score.

Reference:

Higgins JPT, Thomas J, Chandler J, Cumpston M, Li T, Page MJ, Welch VA (editors). Cochrane Handbook for Systematic Reviews of Interventions version 6.2 (updated February 2021). Cochrane, 2021. Available from [www.training.cochrane.org/handbook](http://www.training.cochrane.org/handbook)

## 3.4 Methods for estimation of standard deviation of change score from baseline

We followed the method recommended by Cochrane handbook 6.5.2.8 Imputing standard deviations for changes from baseline.

Reference:

https://training.cochrane.org/handbook/current/chapter-06#section-6-5

# Appendix 4: PRISMA flow diagram

**Identification**

Records identified from:

Pubmed (n = 109)

Embase (n =45)

Cochrane (n =15)

CNKI (n =354)

Wanfang data (n =349)

VIP (n =260)

Total (n =1,132)

Records removed before screening: Duplicate records removed (n = 533)

**Screening**

Records excluded for not beoing relevant based on title/abstract

(n = 335)

Records screened (n = 599)

Reports excluded:

Unclear random (195)

Not randomized trail (n = 5)

Irrelevant intervention (4)

Irrelevant diagnosis criteria (n =8)

N0 available outcome (1)

**Included**

Full-text articles assessed for eligibility (n =264)

Meta-analyses included in review (n =51)

# Appendix 5: Characteristics of included studies

## 5.1 Summary of characteristics of included studies

| Study characteristics | No. (%)/ Median | IQR | Range |
| --- | --- | --- | --- |
| Eligible studies: | | | |
| Total No of trials | 51 |  |  |
| No of participants | 4507 |  |  |
| Median treatment duration (weeks) | 8 | 6 to 8 | 2 to 12 |
| Median follow-up (weeks) | 8 | 6 to 8 | 2 to 12 |
| Participants: | | | |
| Mean age (years) | 62.1 | 59.48 to 65.5 | 42.68 to 73.7 |
| % of female | 46.6 | 42.85 to 46.3 | 31.4 to 61.9 |
| Baseline mean HAMD | 25.66 | 21.81 to 28.04 | 18.05 to 28.04 |
| % of cerebral infarction | 80 | 71.6 to 93.6 | 27.7 to 100 |

**Footnotes: IQR = interquartile range**

## 5.2 List of included studies

| Study | Participants  (N) | Intervention/control  (N) | Treatment  duration | Length of  follow-up | Setting | Depression  Diagnostic criteria |
| --- | --- | --- | --- | --- | --- | --- |
| An, L 2019 | 100 | SJC 50 | 6 weeks | 6-week | Inpatient | CCMD-Ⅲ |
|  |  | Usual care 50 |  |  |  |  |
| Chai, D 2015 | 72 | Wuling capsule + Flupentixol/Melitracen 36 | 8 weeks | 8-week | Inpatient | HAMD |
|  |  | Flupentixol/Melitracen 36 |  |  |  |  |
| Chen, D 2007 | 90 | Hypericum 45 | 4 weeks | 4-week | Inpatient | CCMD-Ⅱ |
|  |  | Usual care 45 |  |  |  |  |
| Chen, J 2015 | 90 | Wuling capsule 45 | 12 weeks | 12-week | Inpatient | CCMD-Ⅲ |
|  |  | Fluoxetine 45 |  |  |  |  |
| Chen, JB 2015 | 156 | SJC 78 | 6 weeks | 6-week | Mixed | CCMD-Ⅲ |
|  |  | Paroxetine 78 |  |  |  |  |
| Chen, L 2015 | 68 | Wuling capsule + Flupentixol/Melitracen 34 | 6 weeks | 6-week | Inpatient | CCMD-Ⅲ、HAMD |
|  |  | Flupentixol/Melitracen 34 |  |  |  |  |
| Deng, X 2016 | 55 | SJC 28 | 12 weeks | 12-week | Mixed | CCMD-Ⅲ |
|  |  | Sertraline 27 |  |  |  |  |
| Fang, J 2015 | 80 | SJC 40 | 6 weeks | 6-week | Mixed | DSM-Ⅳ |
|  |  | Usual care 40 |  |  |  |  |
| Fu, J 2008 | 114 | Wuling capsule 39 | 6 weeks | 6-week | Mixed | CCMD-Ⅲ |
|  |  | Flupentixol/Melitracen 37 |  |  |  |  |
|  |  | Wuling capsule + Flupentixol/Melitracen 38 |  |  |  |  |
| Gao, G 2016 | 73 | SJC + Flupentixol/Melitracen 37 | 6 weeks | 6-week | Mixed | HAMD |
|  |  | SJC 36 |  |  |  |  |
| Guo, H 2016 | 80 | SJC 40 | 6 weeks | 6-week | Inpatient | CCMD-Ⅲ |
|  |  | Paroxetine 40 |  |  |  |  |
| Hou, J 2015 | 72 | SJC + Paroxetine 36 | 8 weeks | 8-week | Outpatient | HAMD |
|  |  | Paroxetine 36 |  |  |  |  |
| Huang, R 2016 | 76 | Wuling capsule + Flupentixol/Melitracen 38 | 8 weeks | 8-week | Inpatient | CCMD-Ⅲ |
|  |  | Flupentixol/Melitracen 38 |  |  |  |  |
| Jia, K 2013 | 73 | Jie Yu Pill + Paroxetine 40 | 8 weeks | 8-week | Mixed | CCMD |
|  |  | Paroxetine 33 |  |  |  |  |
| Jia, K 2017 | 52 | SJC + Citalopram 26 | 2 months | 2-month | Inpatient | CCMD-Ⅲ |
|  |  | Citalopram 26 |  |  |  |  |
| Lei, X 2016 | 109 | SJC 36 | 8 weeks | 8-week | Mixed | CCMD-Ⅲ |
|  |  | Escitalopram 31 |  |  |  |  |
|  |  | SJC + Escitalopram 42 |  |  |  |  |
| Li, F 2019 | 93 | Wuling capsule + Hypericum 47 | 8 weeks | 8-week | Inpatient | CCMD-4 |
|  |  | Hypericum 46 |  |  |  |  |
| Li, W 2018 | 128 | SJC 64 | 8 weeks | 8-week | Inpatient | DSM-Ⅴ |
|  |  | Sertraline 64 |  |  |  |  |
| Li, X 2014 | 100 | Jie Yu Pill + Paroxetine 50 | 8 weeks | 8-week | Inpatient | HAMD |
|  |  | Paroxetine 50 |  |  |  |  |
| Li, Z 2010 | 60 | Danzhixiaoyaosan 30 | 60 days | 60-day | Mixed | CCMD-Ⅲ |
|  |  | Wuling capsule 30 |  |  |  |  |
| Li, L 2008 | 150 | FEW 60 | 8 weeks | 8-week | Mixed | HAMD |
|  |  | Fluoxetine 60 |  |  |  |  |
|  |  | Placebo 30 |  |  |  |  |
| Liang, Z 2019 | 80 | Ginkgobiloba extract plus venlafaxine 40 | 8 weeks | 8-week | Mixed | HAMD |
|  |  | Venlafaxine 40 |  |  |  |  |
| Liu, Y 2014 | 81 | Wuling capsule + Fluoxetine 41 | 6 weeks | 6-week | Mixed | DSM |
|  |  | Fluoxetine 41 |  |  |  |  |
| Luo, C 2014 | 67 | Jie Yu Pill + Fluoxetine 34 | 8 weeks | 8-week | Mixed | CCMD-Ⅲ |
|  |  | Fluoxetine 33 |  |  |  |  |
| Mao, S 2016 | 120 | SJC 60 | 8 weeks | 8-week | Outpatient | HAMD |
|  |  | Fluoxetine 60 |  |  |  |  |
| Mo, W 2004 | 65 | Hypericum 32 | 6 weeks | 6-week | Mixed | CCMD-Ⅱ |
|  |  | Fluoxetine 33 |  |  |  |  |
| Na, W 2012 | 80 | SJC + Sertraline 41 | 8 weeks | 8-week | Mixed | CCMD-Ⅲ |
|  |  | Sertraline 39 |  |  |  |  |
| Shen, G 2019 | 68 | Wuling capsule + Sertraline 34 | 4 weeks | 4-week | Inpatient | CCMD-Ⅲ |
|  |  | Sertraline 34 |  |  |  |  |
| Shi, Z 2017 | 102 | SJC + Fluoxetine 52 | 6 weeks | 6-week | Inpatient | CCMD-Ⅲ |
|  |  | Fluoxetine 50 |  |  |  |  |
| Su, W 2012 | 84 | SJC 42 | 6 weeks | 6-week | Mixed | DSM |
|  |  | Citalopram 42 |  |  |  |  |
| Sun, X 2010 | 98 | Hypericum 50 | 12 weeks | 12-week | Inpatient | CCMD-Ⅲ |
|  |  | SNRI 48 |  |  |  |  |
| Tao, Z 2020 | 66 | Wuling capsule + Venlafaxine 33 | 6 weeks | 6-week | Inpatient | DSM-V |
|  |  | Venlafaxine 33 |  |  |  |  |
| Tian, J 2020 | 98 | Jie Yu Pill + Escitalopram 51 | 8 weeks | 8-week | Mixed | HAMD |
|  |  | Escitalopram 47 |  |  |  |  |
| Tian, X 2021 | 90 | Wuling capsule + Flupentixol/Melitracen 29 | 2 weeks | 2-week | Inpatient | CCMD-Ⅴ |
|  |  | Flupentixol/Melitracen 31 |  |  |  |  |
|  |  | Usual care 30 |  |  |  |  |
| Wan, A 2006 | 70 | Wuling capsule + Paroxetine 35 | 6 weeks | 6-week | Inpatient | HAMD |
|  |  | Paroxetine 35 |  |  |  |  |
| Wang, T 2021 | 96 | SJC + Venlafaxine 48 | 6 weeks | 6-week | Mixed | CCMD-Ⅲ |
|  |  | Venlafaxine 48 |  |  |  |  |
| Wang, Z 2008 | 72 | FEW + Fluoxetine 36 | 3 months | 3-month | Mixed | CCMD-Ⅲ |
|  |  | Fluoxetine 36 |  |  |  |  |
| Wu, H 2015 | 80 | SJC + Flupentixol/Melitracen 40 | 4 weeks | 4-week | Mixed | CCMD-Ⅲ |
|  |  | Flupentixol/Melitracen 40 |  |  |  |  |
| Wu, W 2017 | 70 | SJC 35 | 6 weeks | 6-week | Mixed | CCMD-Ⅲ |
|  |  | Flupentixol/Melitracen 35 |  |  |  |  |
| Wu, Y 2016 | 108 | Morinda Oligosaccharides + Duloxetine 36 | 8 weeks | 8-week | Mixed | CCMD-Ⅲ |
|  |  | Morinda Oligosaccharides 36 |  |  |  |  |
|  |  | Duloxetine 36 |  |  |  |  |
| Xie, Y 2018 | 98 | Wuling capsule + Sertraline 49 | 8 weeks | 8-week | Mixed | CCMD-Ⅲ |
|  |  | Sertraline 49 |  |  |  |  |
| Xu, B 2007 | 108 | Wuling capsule + Fluoxetine 36 | 12 weeks | 12-week | Inpatient | CCMD-Ⅲ |
|  |  | Wuling capsule 36 |  |  |  |  |
|  |  | Fluoxetine 36 |  |  |  |  |
| Yang, H 2018 | 90 | Jie Yu Pill + Paroxetine 45 | 12 weeks | 12-week | Inpatient | CCMD-Ⅲ |
|  |  | Paroxetine 45 |  |  |  |  |
| Ye, Y 2021 | 74 | SJC + Fluoxetine 37 | 2 months | 2-month | Mixed | CCMD-Ⅲ |
|  |  | Fluoxetine 37 |  |  |  |  |
| Yu, W 2014 | 150 | Wuling capsule + Flupentixol/Melitracen 50 | 2 months | 2-month | Mixed | CCMD-Ⅲ |
|  |  | Wuling capsule 50 |  |  |  |  |
|  |  | Flupentixol/Melitracen 50 |  |  |  |  |
| Zeng, M 2018 | 86 | FEW + Fluoxetine 43 | 4 weeks | 4-week | Inpatient | CCMD-Ⅲ |
|  |  | Fluoxetine 43 |  |  |  |  |
| Zhang, M 2019 | 120 | Wuling capsule + Sertraline 40 | 6 weeks | 6-week | Inpatient | ICD-10、HAMD |
|  |  | Wuling capsule 40 |  |  |  |  |
|  |  | Sertraline 40 |  |  |  |  |
| Zhang, Y 2013 | 70 | SJC + Venlafaxine 35 | 6 weeks | 6-week | Mixed | CCMD-Ⅲ |
|  |  | Venlafaxine 35 |  |  |  |  |
| Zhao, B 2005 | 64 | Hypericum 33 | 60 days | 60-day | Inpatient | CCMD-Ⅱ |
|  |  | Usual care 31 |  |  |  |  |
| Zhao, G 2015 | 80 | SJC + Escitalopram 40 | 8 weeks | 8-week | Mixed | CCMD-Ⅲ |
|  |  | Escitalopram 40 |  |  |  |  |
| Zhao, Z 2013 | 80 | SJC + Paroxetine 40 | 6 weeks | 6-week | Mixed | CCMD-Ⅲ |
|  |  | Paroxetine 40 |  |  |  |  |

**Footnotes: If the study included all participants from outpatient, we categorized this as “outpatient”. Same for “inpatient”. If the study included both, we categorized it as “mixed”.**

## 5.3 List of included patients

| Study  (Author year) | Interventions/Controls | | Mean Age(sd) | Female  (%) | Dose | Baseline Severity Scale | Mean baseline  HAMD (SD) | Cerebral infarction   (%) | Mean duration of post-stroke depression(sd) |
| --- | --- | --- | --- | --- | --- | --- | --- | --- | --- |
|  | **Drugs** | **no.** |  |  |  |  |  |  |  |
| An, L 2019 | SJC | 50 | 65.34 (10.65) | 0.46 | 720mg | HAMD 17 | 23.58 (1.42) | — | 28.64 (16. 35) days |
|  | Usual care | 50 | 66.25 (10.35) | 0.44 | — |  | 23.65 (1.32) |  | 28.53 (16.87) days |
| Chai, D 2015 | Wuling capsule + Flupentixol/Melitracen | 36 | 65.2 (7.3) | 0.416 | 3 pills tid; 0.5mg bid | HAMD 17 | 23.1 (3.6) | — | — |
|  | Flupentixol/Melitracen | 36 |  |  | 0.5mg bid |  | 23.6 (3.2) |  |  |
| Chen, D2007 | Hypericum | 45 | 68(5) | 0.356 | 300mg tid | — | 21.77(2.19) | 0.644 | — |
|  | Usual care | 45 | 65(6) | 0.378 | — |  | 21.87(1.95) | 0.556 |  |
| Chen, J 2015 | Wuling capsule | 45 | 61.34 (8.75) | 0.444 | 1.2g tid; 20mg qd | HAMD 17 | 22.48 (4.15) | — | — |
|  | Fluoxetine | 45 | 61.30 (8.73) | 0.467 | 20mg qd |  | 22.50 (4.11) |  |  |
| Chen, JB 2015 | SJC | 78 | 62.04 (2.24) | 0.474 | 4 pills bid | HAMD 24 | 26.64 (6.3) | — | — |
|  | SSRI | 78 | 63.21 (2.30) | 0.487 | 20-40mg qd |  | 25.5 (4.79) |  |  |
| Chen, L 2015 | Wuling capsule + Flupentixol/Melitracen | 34 | 56.4 (2.3) | 0.426 | 1.2g tid; 0.25mg qd-1mg bid | HAMD 17 | 29.89 (8.24) | — | — |
|  | Flupentixol/Melitracen | 34 |  |  | 0.25mg qd-1mg bid |  | 29.67 (8.45) |  |  |
| Deng, X 2016 | SJC | 28 | 61.0 (11.3) | 0.429 | 720 mg bid | HAMD 24 | 23.2 (6.1) | 0.714 | — |
|  | Sertraline | 27 | 62.4 (10.6) | 0.37 | 50 mg qd |  | 22.1 (5.8) | 0.778 |  |
| Fang, J 2015 | SJC | 40 | 65.8 (15.8) | 0.525 | 0.36g bid | HAMD 24 | 23.22 (3.02) | 0.75 | — |
|  | Usual care | 40 | 64.5 (14.5) | 0.45 | — |  | 22.34 (2.35) | 0.775 |  |
| Fu, J 2008 | Wuling capsule + Flupentixol/Melitracen | 38 | 52.5 (5.3) | 0.541 | 1.2g tid; 0.5mg bid | HAMD 17 | 20.76 (4.87) | — | — |
|  | Wuling capsule | 39 |  |  | 1.2g tid |  | 20.23 (5.74) |  |  |
|  | Flupentixol/Melitracen | 37 |  |  | 0.5mg bid |  | 20.22 (4.99) |  |  |
| Gao, G 2016 | SJC + Flupentixol/Melitracen | 37 | 65.4 (7.2) | 0.411 | 2 pills bid; 0.5mg bid | HAMD 24 | 24.1 (6.3) | 0.534 | — |
|  | SJC | 36 |  |  | 2 pills bid |  | 23.9 (5.7) |  |  |
| Guo, H 2016 | SJC | 40 | 63.3 (5.1) | 0.45 | 2 pills bid | HAMD 24 | — | — | — |
|  | Paroxetine | 40 | 60.5 (5.8) | 0.55 | 20mg qd |  |  |  |  |
| Hou, J 2015 | SJC + Paroxetine | 36 | 68.9 (7.1) | 0.417 | 0.72g bid; 20mg qd | HAMD 24 | 27.9 (4.5) | 0.75 | 10.2(2.0)  months |
|  | Paroxetine | 36 | 69.3 (7.5) | 0.472 | 20mg qd |  | 28.1 (4.8) | 0.81 | 9.8(2.1)  months |
| Huang, R 2016 | Wuling capsule + Flupentixol/Melitracen | 38 | 52.11 (8.7) | 0.552 | 1.2g tid; 0.5mg bid | HAMD 24 | 28.32 (1.99) | — | — |
|  | Flupentixol/Melitracen | 38 | 51.71 (8.52) | 0.526 | 0.5mg bid |  | 27.97 (2.03) |  |  |
| Jia, K 2017 | SJC + Citalopram | 26 | 61.2 (4.4) | 0.423 | 360mg bid; 20-40mg qd | HAMD 24 | 18.23(1.51) | — | 4.1(0.9) years |
|  | Citalopram | 26 | 60.7 (5.2) | 0.461 | 20-40mg qd |  | 18.05(1.43) |  | 4.2(0,7) years |
| Jia, K 2013 | Jie Yu Pill + Paroxetine | 40 | 62.1 (6.5) | 0.55 | 4g tid | HAMD 24 | 27.4 (4.4) | 0.8 | — |
|  | Paroxetine | 33 | 62.5 (7.3) | 0.515 | 20-60mg qd |  | 26.8 (3.9) | 0.848 |  |
| Lei, X 2016 | SJC + Escitalopram | 42 | 52.75 (7.34) | 0.476 | 1440mg qd; 21.57±8.22mg qd | HAMD 17 | 27.36 (3.37) | — | — |
|  | SJC | 36 | 52.61 (9.42) | 0.472 | 1440mg qd |  | 27.57 (3.24) |  |  |
|  | Escitalopram | 31 | 53.14 (8.72) | 0.516 | 22.10±7.86mg qd |  | 26.94 (3.43) |  |  |
| Li, F 2019 | Wuling capsule + Hypericum | 47 | 59.62 (5.17) | 0.511 | 0.99g tid; 0.56g bid | HAMD 17 | 21.33 (1.1) | 1 | 2.27 (0.77) months |
|  | Hypericum | 46 | 60.80 (4.33) | 0.543 | 0.56g bid |  | 21.14 (1.28) | 1 | 2.20 (0.81) months |
| Li, W 2018 | SJC | 64 | 71.1 (12.3) | 0.531 | 0.72g bid | HAMD 24 | 28.89 (5.03) | — | 19.1 (15.8) months |
|  | Sertraline | 64 | 73.7 (11.3) | 0.578 | 25-100mg qd |  | 29.88 (5.21) |  | 19.7 (16.3) months |
| Li, X 2014 | Jie Yu Pill + Paroxetine | 50 | 62.4 (11.3) | 0.52 | 4g tid; 20-60mg | HAMD 24 | 27.8 (4.6) | 0.8 | 6.74 (2.73) months |
|  | Paroxetine | 50 | 62.1 (10.9) | 0.5 | 20-60mg |  | 26.5 (4.1) | 0.78 | 6.93 (2.88) months |
| Li, L 2008 | FEW | 60 | 68.5 (4.1) | 0.533 | 18g bid | HAMD 24 | 25.2 (3.8) | — | — |
|  | Fluoxetine | 60 | 69.2 (3.5) | 0.583 | 40mg bid |  | 25.5 (3.1) |  |  |
|  | Placebo | 30 | 67.8 (3.9) | 0.433 | 18g bid |  | 24.3 (2.9) |  |  |
| Liang, Z 2019 | Ginkgobiloba extract + venlafaxine | 40 | 60.86 (8.63) | 0.45 | 75mg qd; 40-225 mg qd | HAMD 17 | — | — | 11.31 (4.24) months |
|  | Venlafaxine | 40 | 60.52 (8.68) | 0.525 | 40-225 mg qd |  |  |  | 11.03 (4.77) months |
| Li, Z 2010 | FEW | 30 | — | — | — | HAMD 24 | 27.71 (4.18) | 1 | — |
|  | Wuling capsule | 30 |  |  | 0.99g tid |  | 27.10 (4.43) | 1 |  |
| Liu, Y 2014 | Wuling capsule + Fluoxetine | 41 | 51.3 | 0.366 | 0.99g tid; 20mg qd | HAMD 17 | 21.16 (2.03) | — | 26.4 days |
|  | Fluoxetine | 41 | 50.9 | 0.415 | 20mg qd |  | 22.13 (1.78) |  | 26.8 days |
| Luo, C 2014 | Jie Yu Pill + Fluoxetine | 34 | 65 (2.75) | 0.324 | 4g tid; 20mg qd | HAMD 24 | 20.58 (3.7) | — | — |
|  | Fluoxetine | 33 | 68 (3.2) | 0.394 | 4g tid; 20mg qd |  | 19.64 (3.6) |  |  |
| Mao, S 2016 | SJC | 60 | 62.5 (3.8) | 0.467 | 0.72g bid | HAMD 17 | 26.1(1.3) | 0.717 | 34 (8) days |
|  | Fluoxetine | 60 | 61.8 (2.1) | 0.517 | 20-40mg qd |  | 25.3(2.2) | 0.833 | 36 (9) days |
| Mo, W 2004 | Hypericum | 32 | 62.38(9.27) | 0.438 | 150mg tid | HAMD 17 | 26.8(4.9) | — | — |
|  | Fluoxetine | 33 | 63.12(8.83) | 0.485 | 20mg qd |  | 26,7(5.3) |  |  |
| Na, W 2012 | SJC + Sertraline | 41 | 71.12 (5.51) | 0.561 | 0.72g bid; 50-100mg qd | HAMD 17 | 25.87 (6.89) | — | 6.83 (4.08) months |
|  | Sertraline | 39 | 72.54 (7.16) | 0.564 | 50-100mg qd |  | 26.34 (6.97) |  | 6.31 (3.65) months |
| Shen, G 2019 | Wuling capsule + Sertraline | 34 | 62.46 (5.24) | 0.588 | 0.99g tid; 50-200mg | HAMD 24 | 28.34 (5.42) | 0.588 | 2.25 (0.63) months |
|  | Sertraline | 34 | 61.34 (5.53) | 0.618 | 50-200mg |  | 28.26 (5.24) | 0.529 | 2.14 (0.52) months |
| Shi, Z 2017 | SJC + Fluoxetine | 52 | 61.30 (8.7) | 0.519 | 0.72g bid; 20mg qd | — | 22.38 (4.25) | 0.808 | — |
|  | Fluoxetine | 50 | 60.20 (8.6) | 0.52 | 20mg qd |  | 21.01 (4.11) | 0.82 |  |
| Su, W 2012 | SJC | 42 | 68.12 (10.51) | 0.571 | 0.72g bid | HAMD 17 | 20.78 (2.06) | 1 | 5.12 (1.67) months |
|  | Citalopram | 42 | 71.44 (14.26) | 0.619 | 20mg qd |  | 20.83 (1.97) | 1 | 5.51 (1.05) months |
| Sun, X 2010 | Hypericum | 50 | 65.3(8.2) | 0.44 | 300-900mg tid | HAMD 17 | 43.01(4.32) | — | — |
|  | Venlafaxine | 48 | 63.2(7.8) | 0.458 | 150mg tid |  | 43.25(3.08) |  |  |
| Tao, Z 2020 | Wuling capsule + Venlafaxine | 33 | 51.5 (3.56) | 0.454 | 0.99g tid; 75-225mg qd | HAMD 24 | 20.11(5.32) | — | 5.88 (2.44) months |
|  | Venlafaxine | 33 | 52.8 (3.48) | 0.515 | 75-225mg qd |  | 19.87(5.25) |  | 5.90 (2.46) months |
| Tian, J 2020 | Jie Yu Pill + Escitalopram | 51 | 57.09 (7.13) | 0.451 | 4g tid; 10-20mg qd | HAMD 17 | 20.86 (3.07) | 0.765 | 1.3 (0.28) years |
|  | Escitalopram | 47 | 57.96 (6.81) | 0.447 | 10-20mg qd |  | 19.97 (2.98) | 0.787 | 1.26 (0.27) years |
| Tian, X 2021 | Wuling capsule + Flupentixol/Melitracen | 29 | 56.3(2.0) | 0.517 | 0.99g qn; 0.5mg bid | HAMD 24 | 20.72(2.31) | — | — |
|  | Flupentixol/Melitracen | 31 | 58.1(3.0) | 0.516 | 0.5mg bid |  | 20.00(1.84) |  |  |
|  | Usual care | 30 | 57.4(2.0) | 0.5 | — |  | 20.09(2.34) |  |  |
| Wan, A 2006 | Wuling capsule + Paroxetine | 35 | 59.23 (8.30) | 0.371 | 0.99g tid; 20mg qd | — | 21.71(5.97) | 0.771 | — |
|  | Paroxetine | 35 | 60.13(8.70) | 0.314 | 20mg qd |  | 22.03(6.12) | 0.8 |  |
| Wang, T 2021 | SJC + Venlafaxine | 48 | 52.46(4.78) | 0.458 | 0.72g bid; 75-150mg qd | HAMD 24 | 21.35(3.62) | 1 | 5.13 (2.40) years |
|  | Venlafaxine | 48 | 52.53(4.81) | 0.417 | 75-150mg qd |  | 21.47(3.70) | 1 | 5.25(2.56) years |
| Wang, Z 2008 | FEW + Fluoxetine | 36 | 68.3(7.3) | 0.472 | 9g qd; 20mg qd | HAMD 24 | 28.34(6.27) | 0.361 | — |
|  | FEW | 36 | 69.3(7.8) | 0.5 | 20mg qd |  | 29.52(7.31) | 0.277 |  |
| Wu, H 2015 | SJC + Flupentixol/Melitracen | 40 | — | 0.35 | 0.72g bid; 0.5mg bid | HAMD 17 | 27.90(4.17) | 1 | 2 weeks |
|  | Flupentixol/Melitracen | 40 |  | 0.425 | 0.5mg bid |  | 27.35(4.17) | 1 | 2 weeks |
| Wu, W 2017 | SJC | 35 | 61.32(3.24) | 0.428 | 0.72g bid | HAMD 24 | 27.13(6.86) | 1 | 5.28(0.33) months |
|  | Flupentixol/Melitracen | 35 |  |  | 0.5mg bid |  | 26.89(6.77) | 1 |  |
| Wu, Y 2016 | Morinda Oligosaccharides + Duloxetine | 36 | 65.4(11.2) | 0.389 | 150mg bid; 60mg qd | HAMD 17 | 30.13(6.67) | 0.861 | 38.4(6.7) months |
|  | Morinda Oligosaccharides | 36 | 65.8(13.2) | 0.444 | 150mg bid |  | 28.87(6.18) | 0.806 | 36.7(6.4) months |
|  | Duloxetine | 36 | 64.8(12.0) | 0.361 | 60mg qd |  | 29.66(6.34) | 0.833 | 34.8(8.7) months |
| Xie, Y 2018 | Wuling capsule + Sertraline | 49 | 61.38(5.47) | 0.571 | 0.99g tid; 50mg qd | HAMD 24 | 21.84(2.55) | 1 | 2.19(0.64) months |
|  | Sertraline | 49 | 60.49(5.69) | 0.551 | 50mg qd |  | 22.04(2.47) | 1 | 2.25(0.71) months |
| Xu, B 2007 | Wuling capsule + Fluoxetine | 36 | 61.1(10.2) | 0.472 | 0.99g tid; 20mg qd | HAMD 24 | 29.52(7.32) | 0.72 | 74.0(3.8) days |
|  | Wuling capsule | 36 | 65.2(14.2) | 0.5 | 0.99g tid |  | 27.32(5.68) | 0.806 | 72.0(6.8) days |
|  | Fluoxetine | 36 | 63.4(10.6) | 0.444 | 20mg qd |  | 28.24(6.27) | 0.667 | 72.0(7.8) days |
| Yang, H 2018 | Jie Yu Pill + Paroxetine | 45 | 68.59(8.13) | 0.467 | 4g tid; 20mg qd | — | 29.76(4.61) | — | 2.19(0.46) years |
|  | Paroxetine | 45 | 65.06(7.28) | 0.489 | 20mg qd |  | 28.49(4.83) |  | 2.38(0.61) years |
| Ye, Y 2021 | SJC + Fluoxetine | 37 | 59.34(5.93) | 0.378 | 0.72g bid; 20mg qd | — | — | 0.459 | — |
|  | Fluoxetine | 37 | 60.39(6.77) | 0.405 | 20mg qd |  |  | 0.405 |  |
| Yu, W 2014 | Wuling capsule + Flupentixol/Melitracen | 50 | 65.4(2.6) | 0.46 | 0.99g tid; 0.5mg bid | — | 29.41(6.59) | 0.3 | — |
|  | Wuling capsule | 50 | 65.3(2.4) | 0.48 | 0.99g tid |  | 29.48(6.75) | 0.36 |  |
|  | Flupentixol/Melitracen | 50 | 64.3(2.1) | 0.5 | 0.5mg bid |  | 29.37(6.67) | 0.3 |  |
| Zeng, M 2018 | FEW + Fluoxetine | 43 | 57.53 (4.70) | 0.465 | 8 pills; 20mg qd | HAMD 24 | 21.02 (2.84) | 0.744 | — |
|  | Fluoxetine | 43 | 59.10 (5.33) | 0.419 | 20mg qd |  | 20.75 (4.17) | 0.837 |  |
| Zhang, M 2019 | Wuling capsule + Sertraline | 40 | 42.68(8.97) | 0.425 | 0.99g tid; 50-100mg qd | HAMD 24 | 26.51(5.12) | — | 2.36(0.52) months |
|  | Wuling capsule | 40 | 42.79(8.38) | 0.425 | 0.99g tid |  | 26.06(6.09) |  | 2.31(0.65) months |
|  | Sertraline | 40 | 43.16(9.17) | 0.45 | 50-100mg qd |  | 25.81(5.56) |  | 2.19(0.54) months |
| Zhang, Y 2013 | SJC + Venlafaxine | 35 | 64.10(9.84) | 0.457 | 0.72g bid; 75-150mg qd | HAMD 17 | 35.4(5.88) | 0.857 | — |
|  | Venlafaxine | 35 | 65.30(6.18) | 0.429 | 75-150mg qd |  | 34.66(5.95) | 0.8 |  |
| Zhao, B 2005 | Hypericum | 33 | 62.35(11.44) | 0.455 | 300mg tid | HAMD 17 | 25.2(5.8) | 0.848 | — |
|  | Usual care | 31 | 63.1(10.36) | 0.452 | — |  | 26.7(6.1) | 0.871 |  |
| Zhao, G 2015 | SJC + Escitalopram | 40 | 60.23(9.34) | 0.45 | 0.72g bid; 10mg qd | HAMD 24 | 28.81(6.29) | — | 5.79(2.11) months |
|  | Escitalopram | 40 | 61.01(9.56) | 0.5 | 10mg qd |  | 28.44(6.57) |  | 6.09(9.45) months |
| Zhao, Z 2013 | SJC + Paroxetine | 40 | 61.2(11.2) | 0.475 | 0.72g bid; 20mg qd | HAMD 17 | 24.74(8.84) | — | 5.81(1.94) months |
|  | Paroxetine | 40 | 62.3(10.1) | 0.45 | 20mg qd |  | 25.2(7.26) |  | 6.13(1.89) months |

# Appendix 6: Risk of bias assessments

## 6.1 Outcome: Response rate


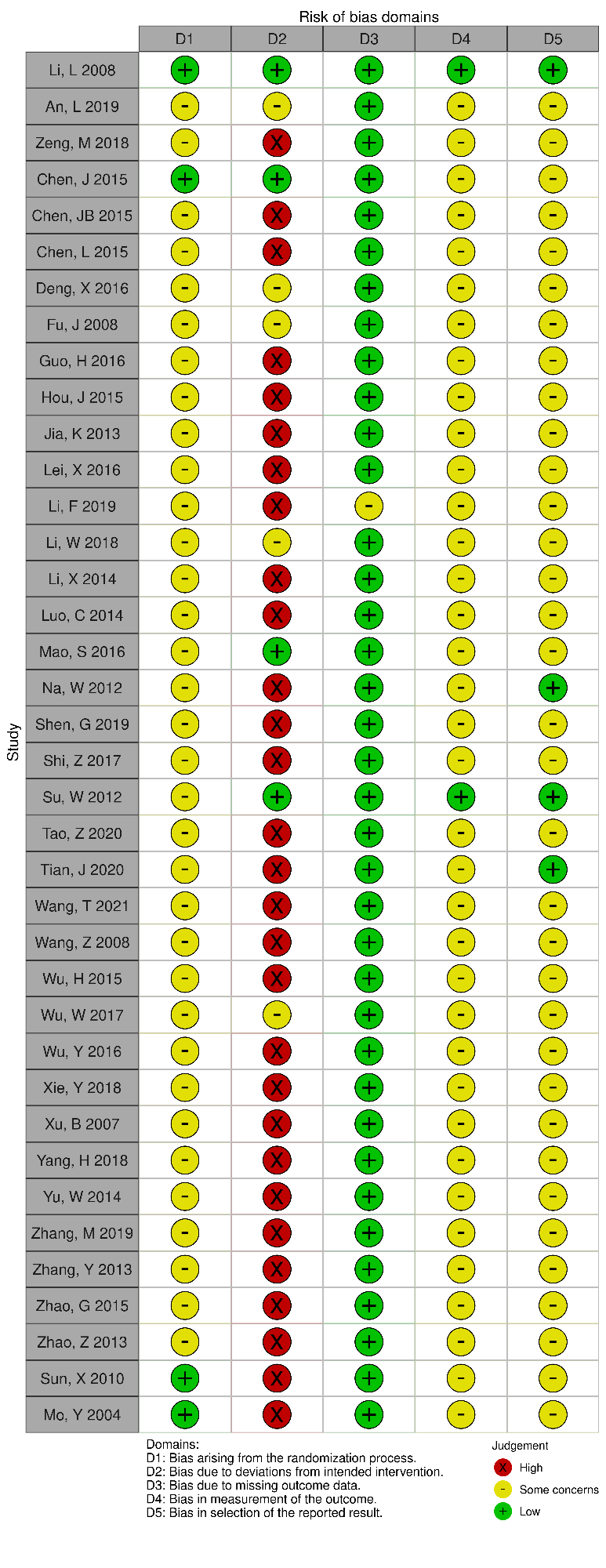


## 6.2 Outcome: Mean changes in HAMD score from baseline


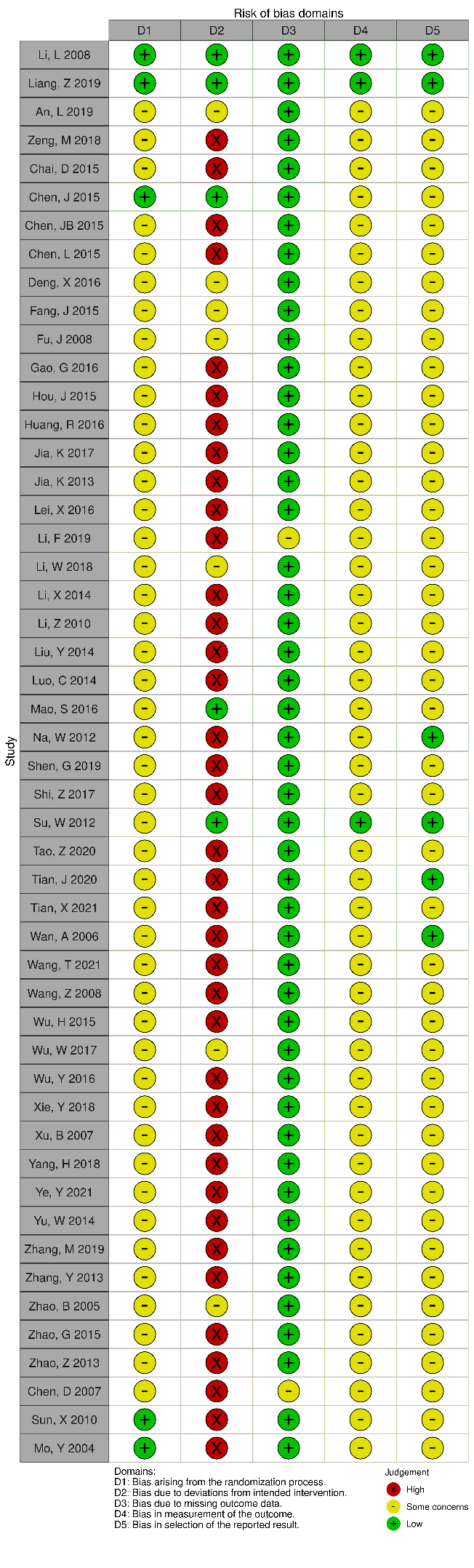


## 6.3 Outcome: All-cause drop out


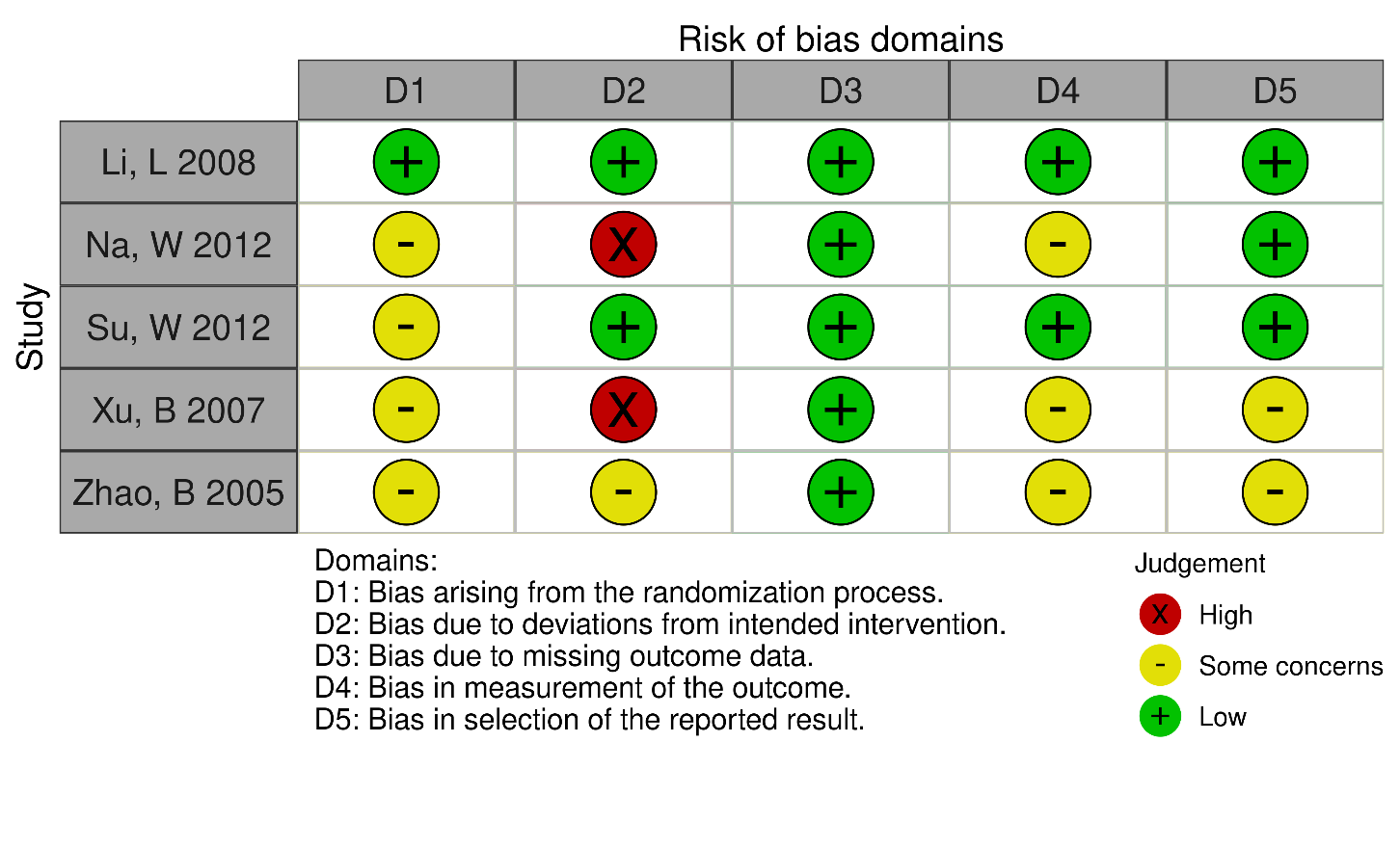


## 6.4 Outcome: Any gastrointestinal event


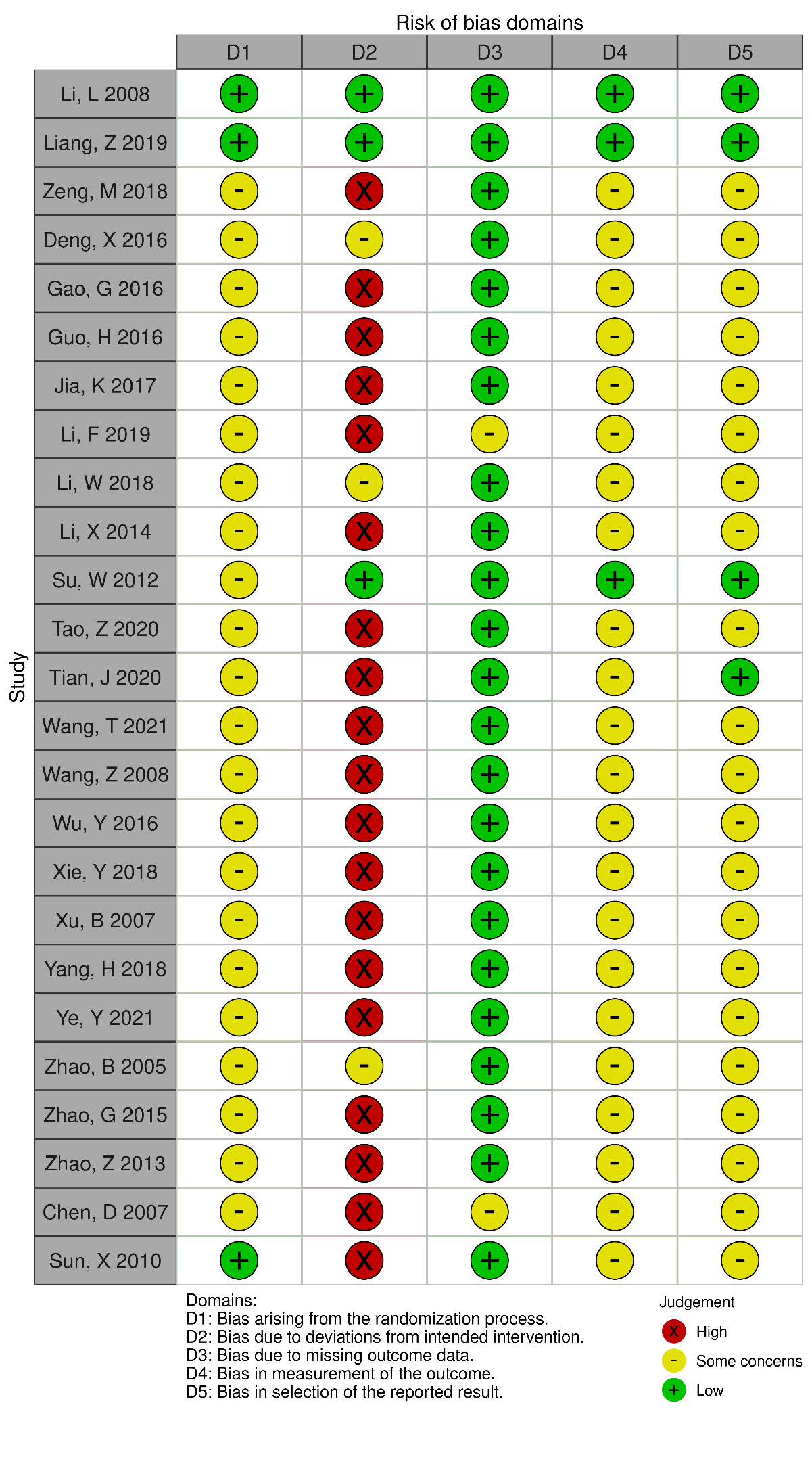


## 6.5 Outcome: Any nervous system event


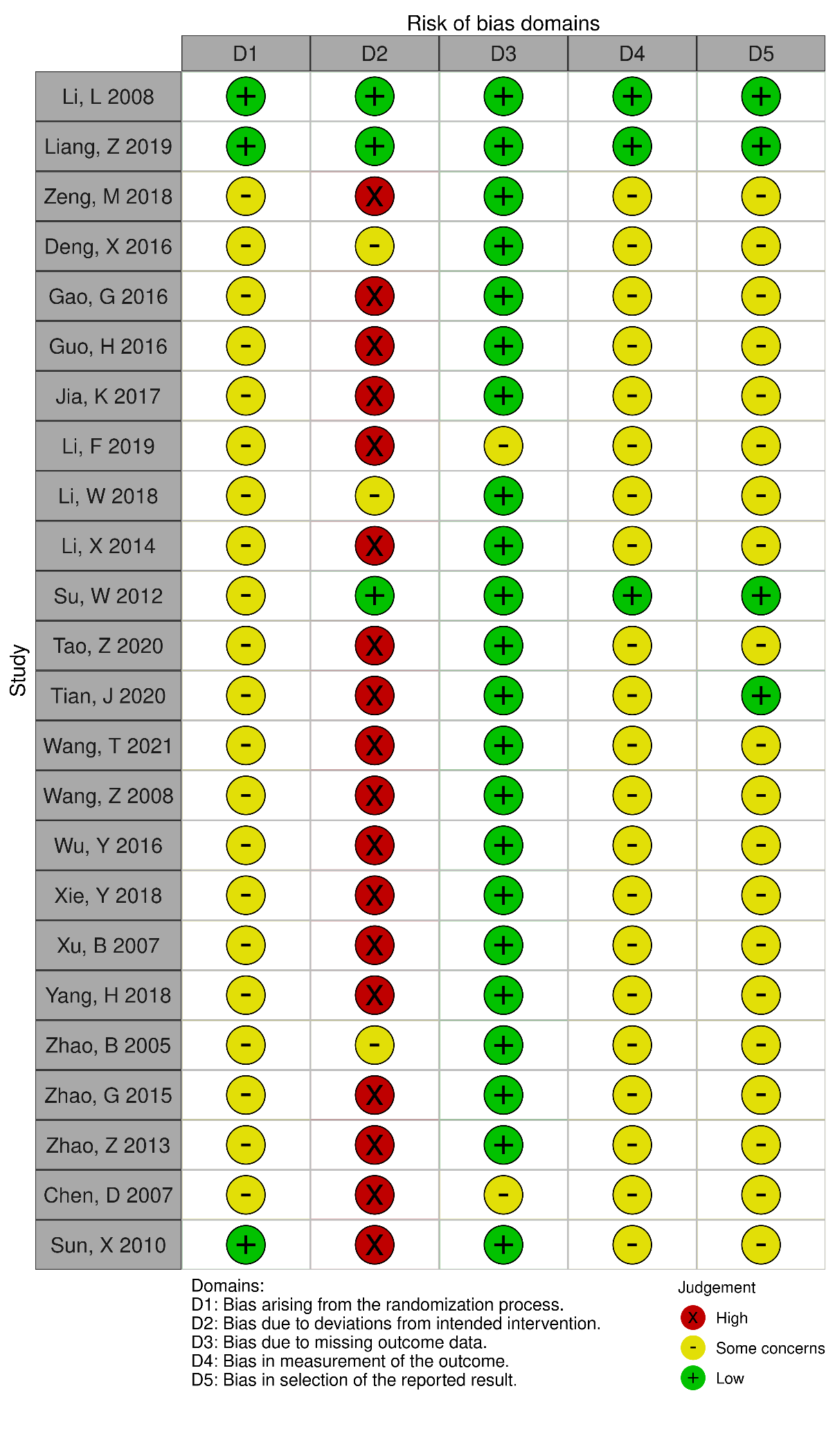


# Appendix 7: Other main results

## 7.1 Network plots

**Outcome: Outcome: Any gastrointestinal event**


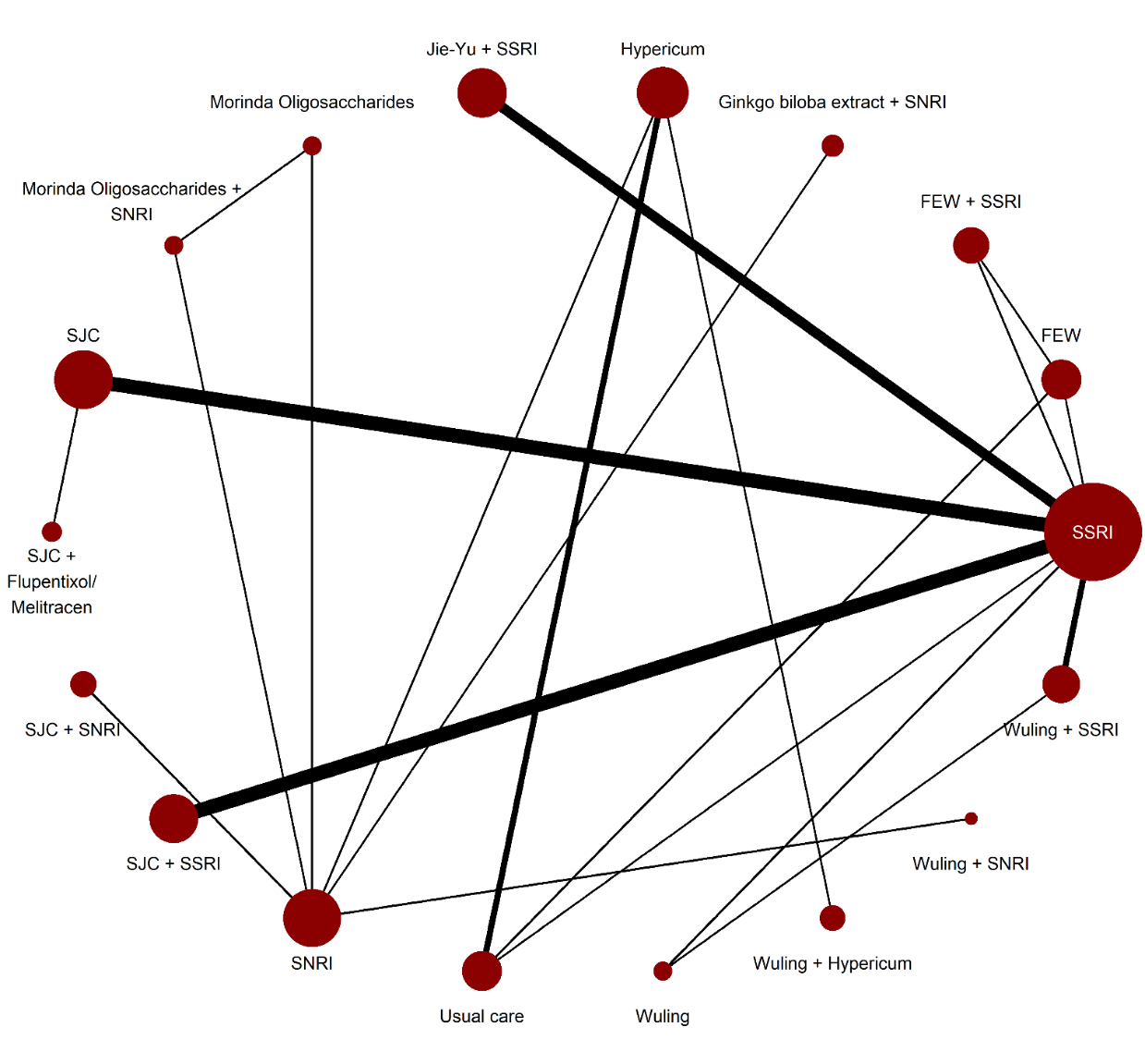


**Outcome: Any nervous system event**


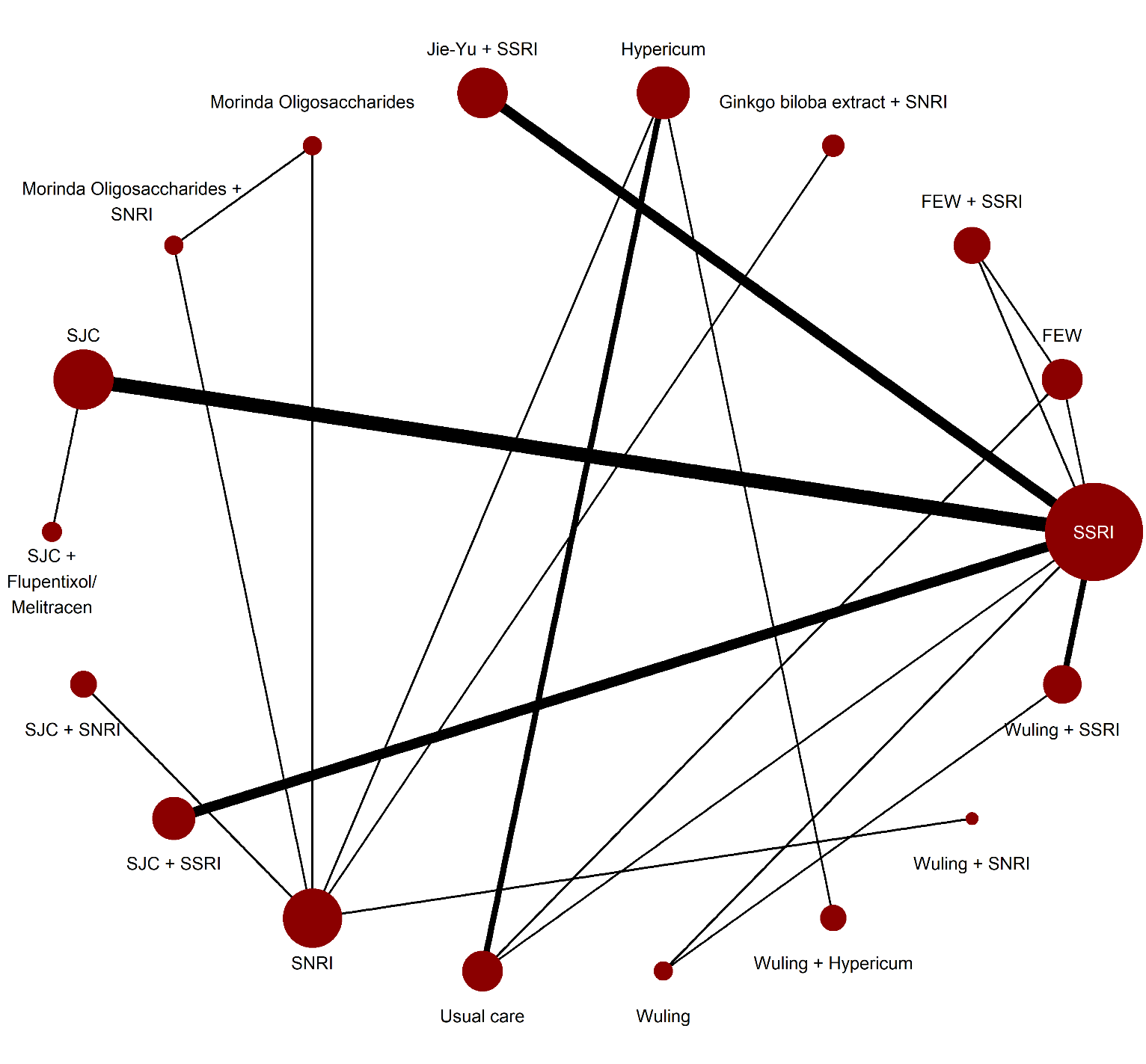


## 7.2 Minimally contextualized framework

| Outcomes, certainty of the evidence, and classification of intervention | Intervention | Intervention vs SSRI alone | Frequentist P-score |
| --- | --- | --- | --- |
| **Response Rate (relative risk; 95% confidence interval)** | | | |
| **High certainty** (moderate to high certainty evidence) | | | |
| Category 2: among the best | SJC + SSRI | 1.45 (1.23, 1.7) | 0.79 |
|  | Jie-Yu + SSRI | 1.35 (1.09, 1.68) | 0.71 |
|  | Wuling + SSRI | 1.32 (1.09, 1.59) | 0.68 |
| Category 1: intermediate | Wuling + Hypericum | 1.65 (0.85, 3.22) | 0.81 |
|  | FEW | 1.02 (0.75, 1.38) | 0.33 |
|  | SSRI | Reference | 0.27 |
| **Low certainty** (low to very low certainty evidence) | | | |
| Category 1: intermediate | SJC + Flupentixol/Melitracen | 1.47 (0.87, 2.49) | 0.74 |
|  | SJC + SNRI | 1.48 (0.74, 2.97) | 0.74 |
|  | Wuling + SNRI | 1.53 (0.59, 3.97) | 0.71 |
|  | Wuling + Flupentixol/Melitracen | 1.21 (0.92, 1.61) | 0.57 |
|  | Morinda Oligosaccharides + SNRI | 1.22 (0.58, 2.57) | 0.55 |
|  | FEW + SSRI | 1.18 (0.86, 1.62) | 0.53 |
|  | SJC | 1.13 (0.97, 1.31) | 0.46 |
|  | Wuling | 1.04 (0.86, 1.27) | 0.34 |
|  | Morinda Oligosaccharides | 0.99 (0.46, 2.13) | 0.34 |
|  | SNRI | 0.99 (0.53, 1.83) | 0.31 |
|  | Hypericum | 0.98 (0.62, 1.56) | 0.29 |
|  | Flupentixol/Melitracen | 0.96 (0.73, 1.27) | 0.24 |
|  | Usual care | 0.64 (0.3, 1.33) | 0.09 |
|  | | | |
| **Mean changes in HAMD score from baseline** **(mean difference; 95% confidence interval)** | | | |
| **High certainty** (moderate to high certainty evidence) | | | |
| Category 2: among the best | Wuling + Hypericum | -10.12 (-17.25, -2.99) | 0.93 |
|  | Wuling + SSRI | -3.81 (-6.19, -1.42) | 0.68 |
| Category 1: intermediate | FEW | -1.92 (-5.68, 1.84) | 0.52 |
|  | SSRI | Reference | 0.33 |
| Category 0: among the worst | Usual care | 5.41 (2.47, 8.34) | 0.04 |
| **Low certainty** (low to very low certainty evidence) | | | |
| Category 2: among the best | Jie-Yu + SSRI | -5.22 (-7.91, -2.53) | 0.78 |
|  | SJC + SSRI | -4.08 (-6.17, -1.99) | 0.70 |
| Category 1: intermediate | SJC + SNRI | -7.91 (-16.2, 0.39) | 0.86 |
|  | Morinda Oligosaccharides + SNRI | -7.16 (-16.58, 2.26) | 0.81 |
|  | Ginkgo biloba extract + SNRI | -5.85 (-15.19, 3.48) | 0.74 |
|  | Wuling + SNRI | -4.68 (-14.03, 4.66) | 0.67 |
|  | SNRI | -2.02 (-9.15, 5.1) | 0.49 |
|  | SJC + Flupentixol/Melitracen | -1.27 (-6.17, 3.64) | 0.46 |
|  | Wuling + Flupentixol/Melitracen | -0.88 (-4.44, 2.68) | 0.42 |
|  | Hypericum | -0.84 (-4.84, 3.15) | 0.41 |
|  | SJC | -0.65 (-2.76, 1.47) | 0.40 |
|  | FEW + SSRI | -0.49 (-5.11, 4.14) | 0.39 |
|  | Morinda Oligosaccharides | 5.1 (-4.32, 14.51) | 0.12 |
| Category 0: among the worst | Wuling | 3.42 (0.75, 6.09) | 0.12 |
|  | Flupentixol/Melitracen | 3.49 (0.2, 6.79) | 0.12 |
|  | | | |
| **Any gastrointestinal event (relative risk; 95% confidence interval)** | | | |
| **High certainty** (moderate to high certainty evidence) | | | |
| Category 2: among the best | SJC | 0.34 (0.18, 0.62) | 0.26 |
| Category 1: intermediate | SSRI | Reference | 0.66 |
|  | Usual care | 1.00 (0.27, 3.72) | 0.63 |
|  | FEW | 0.33 (0.07, 1.59) | 0.29 |
| **Low certainty** (low to very low certainty evidence) | | | |
| Category 1: intermediate | Morinda Oligosaccharides + SNRI | 0.40 (0.00, 50.36) | 0.70 |
|  | SJC + SNRI | -7.91 (-16.2, 0.39) | 0.69 |
|  | Ginkgo biloba extract + SNRI | 1.34 (0.03, 59.68) | 0.64 |
|  | SJC + SSRI | 0.91 (0.56, 1.48) | 0.61 |
|  | Wuling + SSRI | 0.89 (0.46, 1.71) | 0.60 |
|  | SNRI | 1.07 (0.03, 38.77) | 0.58 |
|  | Jie-Yu + SSRI | 0.83 (0.39, 1.77) | 0.57 |
|  | Wuling + Hypericum | 0.36 (0.00, 42.76) | 0.52 |
|  | Hypericum | 0.52 (0.03, 7.62) | 0.41 |
|  | Wuling + SNRI | 0.36 (0, 42.76) | 0.37 |
|  | Morinda Oligosaccharides | 0.36 (0.00, 42.85) | 0.37 |
|  | FEW + SSRI | 0.33 (0.06, 1.81) | 0.30 |
|  | Wuling | 0.36 (0.12, 1.02) | 0.29 |
|  | | | |
| **Any nervous system event (relative risk; 95% confidence interval)** | | | |
| High certainty (moderate to high certainty evidence) | | | |
| Category 2: among the best | SJC | 0.11 (0.03,0.35) | 0.23 |
| Category 1: intermediate | SSRI | Reference | 0.71 |
|  | Usual care | 1.10 (0.25, 4.86) | 0.73 |
|  | FEW | 0.11 (0.01, 2.02) | 0.27 |
| Low certainty (low to very low certainty evidence) | | | |
| Category 1: intermediate | Jie-Yu + SSRI | 1.17 (0.34, 4.07) | 0.74 |
|  | SJC + SSRI | 1.16 (0.37, 3.62) | 0.74 |
|  | Ginkgo biloba extract + SNRI | 0.47 (0.01, 33.79) | 0.58 |
|  | Hypericum | 0.57 (0.04, 9.16) | 0.58 |
|  | Wuling + Hypericum | 0.56 (0.01, 27.59) | 0.57 |
|  | Morinda Oligosaccharides + SNRI | 0.39 (0.00, 49.57) | 0.53 |
|  | Wuling + SSRI | 0.47 (0.14, 1.53) | 0.51 |
|  | Wuling | 0.34 (0.04, 3.19) | 0.46 |
|  | Morinda Oligosaccharides | 0.20 (0.00, 30.11) | 0.39 |
|  | SNRI | 0.20 (0.00, 13.43) | 0.36 |
|  | SJC + SNRI | 0.16 (0.00, 12.94) | 0.32 |
|  | FEW + SSRI | 0.11 (0.00, 2.95) | 0.28 |

## 7.3 Network estimates (league tables)

All network estimates were shown in league table. The lower left corner represents the network results. The results of comparisons should read from left to right with the column-treatment compared with the row-treatment.

**Outcome: Response rate**

| FEW |  |  |  |  |  |  |  |  |  |  |  |  |  |  |  |  |  |  |
| --- | --- | --- | --- | --- | --- | --- | --- | --- | --- | --- | --- | --- | --- | --- | --- | --- | --- | --- |
| 0.86 (0.64,1.17) | FEW + SSRI |  |  |  |  |  |  |  |  |  |  |  |  |  |  |  |  |  |
| 1.06 (0.70,1.59) | 1.22 (0.80,1.86) | Flupentixol/ Melitracen |  |  |  |  |  |  |  |  |  |  |  |  |  |  |  |  |
| 1.04 (0.60,1.82) | 1.21 (0.69,2.12) | 0.98 (0.57,1.69) | Hypericum |  |  |  |  |  |  |  |  |  |  |  |  |  |  |  |
| 0.75 (0.52,1.09) | 0.87 (0.59,1.28) | 0.71 (0.50,1.01) | 0.72 (0.43,1.21) | Jie-Yu + SSRI |  |  |  |  |  |  |  |  |  |  |  |  |  |  |
| 1.03 (0.45,2.36) | 1.20 (0.52,2.75) | 0.98 (0.43,2.21) | 0.99 (0.54,1.83) | 1.37 (0.62,3.05) | Morinda Oligosaccharides |  |  |  |  |  |  |  |  |  |  |  |  |  |
| 0.84 (0.37,1.87) | 0.97 (0.43,2.17) | 0.79 (0.36,1.75) | 0.80 (0.45,1.43) | 1.11 (0.51,2.41) | 0.81 (0.53,1.23) | Morinda Oligosaccharides + SNRI |  |  |  |  |  |  |  |  |  |  |  |  |
| 0.91 (0.65,1.27) | 1.05 (0.74,1.49) | 0.86 (0.64,1.14) | 0.87 (0.53,1.42) | 1.20 (0.92,1.56) | 0.88 (0.40,1.91) | 1.08 (0.51,2.31) | SJC |  |  |  |  |  |  |  |  |  |  |  |
| 0.69 (0.38,1.27) | 0.80 (0.43,1.48) | 0.66 (0.42,1.03) | 0.67 (0.33,1.34) | 0.92 (0.52,1.62) | 0.67 (0.26,1.70) | 0.83 (0.33,2.07) | 0.77 (0.45,1.31) | SJC + Flupentixol/ Melitracen |  |  |  |  |  |  |  |  |  |  |
| 0.69 (0.32,1.47) | 0.80 (0.37,1.71) | 0.65 (0.31,1.37) | 0.66 (0.39,1.11) | 0.91 (0.44,1.89) | 0.67 (0.38,1.17) | 0.82 (0.48,1.40) | 0.76 (0.37,1.55) | 0.99 (0.41,2.37) | SJC + SNRI |  |  |  |  |  |  |  |  |  |
| 0.71 (0.50,1.00) | 0.82 (0.57,1.17) | 0.67 (0.49,0.91) | 0.68 (0.41,1.11) | 0.94 (0.71,1.23) | 0.68 (0.31,1.49) | 0.84 (0.39,1.81) | 0.78 (0.64,0.96) | 1.02 (0.59,1.76) | 1.02 (0.50,2.09) | SJC + SSRI |  |  |  |  |  |  |  |  |
| 1.03 (0.52,2.05) | 1.20 (0.60,2.39) | 0.98 (0.50,1.92) | 0.99 (0.66,1.49) | 1.37 (0.71,2.63) | 1.00 (0.63,1.58) | 1.24 (0.81,1.88) | 1.14 (0.61,2.15) | 1.49 (0.66,3.35) | 1.50 (1.09,2.08) | 1.47 (0.78,2.77) | SNRI |  |  |  |  |  |  |  |
| 1.02 (0.75,1.38) | 1.18 (0.86,1.62) | 0.96 (0.73,1.27) | 0.98 (0.62,1.56) | 1.35 (1.09,1.68) | 0.99 (0.46,2.13) | 1.22 (0.58,2.57) | 1.13 (0.97,1.31) | 1.47 (0.87,2.49) | 1.48 (0.74,2.97) | 1.45 (1.23,1.70) | 0.99 (0.53,1.83) | SSRI |  |  |  |  |  |  |
| 1.60 (0.73,3.55) | 1.86 (0.83,4.13) | 1.52 (0.70,3.30) | 1.54 (0.64,3.68) | 2.13 (0.99,4.59) | 1.55 (0.53,4.50) | 1.92 (0.67,5.47) | 1.77 (0.86,3.66) | 2.31 (0.94,5.68) | 2.33 (0.85,6.42) | 2.27 (1.07,4.82) | 1.55 (0.59,4.05) | 1.57 (0.75,3.29) | Usual care |  |  |  |  |  |
| 0.98 (0.68,1.40) | 1.13 (0.78,1.65) | 0.92 (0.73,1.16) | 0.94 (0.57,1.56) | 1.30 (0.97,1.74) | 0.94 (0.43,2.09) | 1.17 (0.54,2.53) | 1.08 (0.85,1.37) | 1.41 (0.85,2.33) | 1.42 (0.69,2.93) | 1.39 (1.07,1.79) | 0.94 (0.49,1.80) | 0.96 (0.78,1.17) | 0.61 (0.28,1.30) | Wuling |  |  |  |  |
| 0.84 (0.56,1.27) | 0.97 (0.64,1.49) | 0.79 (0.66,0.96) | 0.81 (0.47,1.39) | 1.12 (0.78,1.59) | 0.81 (0.36,1.84) | 1.01 (0.45,2.23) | 0.93 (0.69,1.25) | 1.21 (0.75,1.97) | 1.22 (0.58,2.59) | 1.19 (0.86,1.65) | 0.81 (0.41,1.60) | 0.82 (0.62,1.09) | 0.52 (0.24,1.15) | 0.86 (0.69,1.08) | Wuling + Flupentixol/ Melitracen |  |  |  |
| 0.62 (0.30,1.29) | 0.72 (0.34,1.50) | 0.58 (0.28,1.20) | 0.59 (0.37,0.96) | 0.82 (0.41,1.65) | 0.60 (0.27,1.30) | 0.74 (0.35,1.57) | 0.68 (0.34,1.35) | 0.89 (0.38,2.09) | 0.90 (0.44,1.82) | 0.88 (0.44,1.74) | 0.60 (0.32,1.12) | 0.61 (0.31,1.18) | 0.39 (0.14,1.04) | 0.63 (0.31,1.27) | 0.73 (0.36,1.52) | Wuling + Hypericum |  |  |
| 0.67 (0.25,1.81) | 0.77 (0.28,2.10) | 0.63 (0.23,1.69) | 0.64 (0.28,1.46) | 0.88 (0.33,2.34) | 0.64 (0.27,1.52) | 0.80 (0.34,1.84) | 0.73 (0.28,1.92) | 0.96 (0.32,2.84) | 0.97 (0.44,2.14) | 0.94 (0.36,2.47) | 0.64 (0.31,1.33) | 0.65 (0.25,1.69) | 0.41 (0.12,1.38) | 0.68 (0.26,1.80) | 0.79 (0.29,2.13) | 1.08 (0.41,2.81) | Wuling + SNRI |  |
| 0.77 (0.54,1.11) | 0.90 (0.62,1.30) | 0.73 (0.54,0.99) | 0.74 (0.45,1.23) | 1.03 (0.77,1.37) | 0.75 (0.34,1.65) | 0.93 (0.43,2.00) | 0.86 (0.68,1.08) | 1.12 (0.65,1.91) | 1.12 (0.55,2.31) | 1.10 (0.86,1.41) | 0.75 (0.39,1.42) | 0.76 (0.63,0.92) | 0.48 (0.23,1.03) | 0.79 (0.64,0.99) | 0.92 (0.68,1.25) | 1.25 (0.63,2.51) | 1.16 (0.44,3.07) | Wuling + SSRI |

**Outcome: Mean changes in HAMD score from baseline**

| FEW |  |  |  |  |  |  |  |  |  |  |  |  |  |  |  |  |  |  |  |
| --- | --- | --- | --- | --- | --- | --- | --- | --- | --- | --- | --- | --- | --- | --- | --- | --- | --- | --- | --- |
| -1.44 ( -6.13, 3.26) | FEW + SSRI |  |  |  |  |  |  |  |  |  |  |  |  |  |  |  |  |  |  |
| -5.41 ( -9.94,-0.89) | -3.98 ( -9.46, 1.51) | Flupentixol  /Melitracen |  |  |  |  |  |  |  |  |  |  |  |  |  |  |  |  |  |
| 3.93 ( -5.95,13.81) | 5.37 ( -4.97,15.70) | 9.34 ( -0.30,18.99) | Ginkgo biloba extract + SNRI |  |  |  |  |  |  |  |  |  |  |  |  |  |  |  |  |
| -1.08 ( -6.22, 4.06) | 0.36 ( -5.61, 6.32) | 4.33 ( -0.35, 9.02) | -5.01 (-13.45, 3.43) | Hypericum |  |  |  |  |  |  |  |  |  |  |  |  |  |  |  |
| 3.30 ( -1.33, 7.92) | 4.73 ( -0.62,10.09) | 8.71 (4.46,12.97) | -0.63 (-10.35, 9.08) | 4.38 ( -0.44, 9.20) | Jie-Yu + SSRI |  |  |  |  |  |  |  |  |  |  |  |  |  |  |
| -7.02 (-16.98, 2.94) | -5.58 (-15.99, 4.82) | -1.61 (-11.33, 8.12) | -10.95 (-19.57, -2.33) | -5.94 (-14.47, 2.59) | -10.32 (-20.11, -0.52) | Morinda Oligosaccharides |  |  |  |  |  |  |  |  |  |  |  |  |  |
| 5.24 ( -4.72,15.20) | 6.68 ( -3.73,17.09) | 10.65 (0.92,20.39) | 1.31 ( -7.32, 9.94) | 6.32 ( -2.21,14.85) | 1.94 ( -7.86,11.74) | 12.26 (6.11,18.41) | Morinda Oligosaccharides + SNRI |  |  |  |  |  |  |  |  |  |  |  |  |
| -1.27 ( -5.37, 2.82) | 0.16 ( -4.84, 5.16) | 4.14 (0.87, 7.41) | -5.21 (-14.61, 4.20) | -0.20 ( -4.35, 3.96) | -4.57 ( -8.00, -1.15) | 5.74 ( -3.74,15.23) | -6.52 (-16.00, 2.97) | SJC |  |  |  |  |  |  |  |  |  |  |  |
| -0.66 ( -6.58, 5.27) | 0.78 ( -5.85, 7.41) | 4.76 (0.09, 9.43) | -4.59 (-14.94, 5.77) | 0.42 ( -5.58, 6.43) | -3.95 ( -9.55, 1.65) | 6.36 ( -4.06,16.79) | -5.90 (-16.33, 4.54) | 0.62 ( -4.04, 5.28) | SJC + Flupentixol/Melitracen |  |  |  |  |  |  |  |  |  |  |
| 5.98 ( -2.92,14.88) | 7.42 ( -1.98,16.82) | 11.40 (2.76,20.04) | 2.05 ( -5.32, 9.43) | 7.06 ( -0.20,14.33) | 2.69 ( -6.03,11.40) | 13.00 (5.53,20.48) | 0.74 ( -6.74, 8.22) | 7.26 ( -1.11,15.63) | 6.64 ( -2.78,16.06) | SJC + SNRI |  |  |  |  |  |  |  |  |  |
| 2.16 ( -2.12, 6.44) | 3.60 ( -1.47, 8.66) | 7.57 (3.72,11.42) | -1.77 (-11.32, 7.78) | 3.24 ( -1.24, 7.72) | -1.14 ( -4.55, 2.27) | 9.18 ( -0.45,18.81) | -3.08 (-12.72, 6.55) | 3.43 (0.59, 6.28) | 2.81 ( -2.47, 8.09) | -3.82 (-12.36, 4.71) | SJC + SSRI |  |  |  |  |  |  |  |  |
| 0.10 ( -7.73, 7.93) | 1.54 ( -6.85, 9.93) | 5.51 ( -2.02,13.05) | -3.83 ( -9.86, 2.20) | 1.18 ( -4.72, 7.08) | -3.20 (-10.81, 4.42) | 7.12 (0.96,13.28) | -5.14 (-11.30, 1.02) | 1.38 ( -5.84, 8.59) | 0.76 ( -7.66, 9.17) | -5.88 (-10.12, -1.64) | -2.06 ( -9.46, 5.35) | SNRI |  |  |  |  |  |  |  |
| -1.92 ( -5.68, 1.84) | -0.49 ( -5.11, 4.14) | 3.49 (0.20, 6.79) | -5.85 (-15.19, 3.48) | -0.84 ( -4.84, 3.15) | -5.22 ( -7.91, -2.53) | 5.10 ( -4.32,14.51) | -7.16 (-16.58, 2.26) | -0.65 ( -2.76, 1.47) | -1.27 ( -6.17, 3.64) | -7.91 (-16.20, 0.39) | -4.08 ( -6.17, -1.99) | -2.02 ( -9.15, 5.10) | SSRI |  |  |  |  |  |  |
| -7.33 (-11.49, -3.16) | -5.89 (-11.13, -0.65) | -1.91 ( -5.41, 1.58) | -11.26 (-20.43, -2.09) | -6.25 ( -9.85, -2.65) | -10.63 (-14.61, -6.64) | -0.31 ( -9.56, 8.94) | -12.57 (-21.83, -3.31) | -6.05 ( -8.95, -3.16) | -6.67 (-11.84, -1.50) | -13.31 (-21.42, -5.21) | -9.49 (-13.04, -5.94) | -7.43 (-14.34, -0.52) | -5.41 ( -8.34, -2.47) | Usual care |  |  |  |  |  |
| -5.34 ( -9.28, -1.41) | -3.91 ( -8.98, 1.17) | 0.07 ( -3.08, 3.23) | -9.27 (-18.86, 0.31) | -4.26 ( -8.82, 0.29) | -8.64 (-12.44, -4.85) | 1.68 ( -7.99,11.34) | -10.58 (-20.25, -0.91) | -4.07 ( -7.19, -0.95) | -4.69 ( -9.82, 0.44) | -11.33 (-19.90, -2.76) | -7.50 (-10.87, -4.14) | -5.44 (-12.89, 2.01) | -3.42 ( -6.09, -0.75) | 1.98 ( -1.51, 5.48) | Wuling |  |  |  |  |
| -1.04 ( -5.73, 3.64) | 0.40 ( -5.24, 6.03) | 4.37 (1.95, 6.79) | -4.97 (-14.70, 4.76) | 0.04 ( -4.81, 4.89) | -4.34 ( -8.81, 0.13) | 5.98 ( -3.83,15.79) | -6.28 (-16.10, 3.53) | 0.23 ( -3.38, 3.84) | -0.39 ( -5.48, 4.71) | -7.03 (-15.76, 1.71) | -3.20 ( -7.29, 0.89) | -1.14 ( -8.78, 6.50) | 0.88 ( -2.68, 4.44) | 6.29 (2.58, 9.99) | 4.30 (0.98, 7.63) | Wuling + Flupentixol/Melitracen |  |  |  |
| 8.20 (0.37,16.03) | 9.64 (1.24,18.03) | 13.61 (6.08,21.15) | 4.27 ( -6.03,14.57) | 9.28 (3.38,15.18) | 4.90 ( -2.72,12.52) | 15.22 (4.85,25.59) | 2.96 ( -7.42,13.34) | 9.48 (2.26,16.69) | 8.86 (0.44,17.27) | 2.22 ( -7.14,11.58) | 6.04 ( -1.37,13.45) | 8.10 ( -0.25,16.45) | 10.12 (2.99,17.25) | 15.53 (8.61,22.44) | 13.54 (6.09,21.00) | 9.24 (1.60,16.88) | Wuling + Hypericum |  |  |
| 2.76 ( -7.13,12.65) | 4.20 ( -6.15,14.54) | 8.17 ( -1.48,17.83) | -1.17 ( -9.71, 7.37) | 3.84 ( -4.61,12.29) | -0.54 (-10.26, 9.19) | 9.78 (1.15,18.41) | -2.48 (-11.12, 6.16) | 4.04 ( -5.38,13.45) | 3.42 ( -6.95,13.78) | -3.22 (-10.61, 4.16) | 0.60 ( -8.96,10.16) | 2.66 ( -3.39, 8.71) | 4.68 ( -4.66,14.03) | 10.09 (0.91,19.27) | 8.10 ( -1.49,17.70) | 3.80 ( -5.94,13.54) | -5.44 (-15.75, 4.87) | Wuling + SNRI | I |
| 1.88 ( -2.44, 6.21) | 3.32 ( -1.83, 8.47) | 7.30 (3.42,11.17) | -2.05 (-11.66, 7.57) | 2.96 ( -1.64, 7.57) | -1.41 ( -5.01, 2.19) | 8.90 ( -0.79,18.59) | -3.36 (-13.05, 6.34) | 3.16 (0.03, 6.29) | 2.54 ( -2.83, 7.91) | -4.10 (-12.70, 4.50) | -0.28 ( -3.44, 2.89) | 1.78 ( -5.70, 9.27) | 3.81 (1.42, 6.19) | 9.21 (5.52,12.90) | 7.23 (4.05,10.40) | 2.93 ( -1.16, 7.01) | -6.32 (-13.81, 1.17) | -0.88 (-10.50, 8.74) | Wuling + SSRI |

**Outcome: All-cause drop out**

| FEW |  |  |  |  |  |  |  |
| --- | --- | --- | --- | --- | --- | --- | --- |
| 0.04  (0.00, 2.81) | Hypericum |  |  |  |  |  |  |
| 0.40  (0.01, 18.43) | 11.19  (0.15, 822.89) | SJC |  |  |  |  |  |
| 0.11  (0.00, 4.84) | 2.94  (0.04, 216.04) | 0.26  (0.01, 7.41) | SJC + SSRI |  |  |  |  |
| 0.20  (0.01, 4.08) | 5.60  (0.15, 202.83) | 0.50  (0.05, 5.31) | 1.90  (0.18, 20.15) | SSRI |  |  |  |
| 0.10  (0.00, 2.04) | 2.82  (0.12, 66.72) | 0.25  (0.01, 4.62) | 0.96  (0.05, 17.56) | 0.50  (0.09, 2.75) | Usual care |  |  |
| 1.00  (0.01, 70.43) | 27.98  (0.26, 3014.39) | 2.50  (0.05, 113.95) | 9.51  (0.21, 433.04) | 5.00  (0.25, 100.58) | 9.92  (0.32, 311.98) | Wuling |  |
| 0.20  (0.01, 6.35) | 5.60  (0.11, 296.21) | 0.50  (0.03, 9.14) | 1.90  (0.10, 34.71) | 1.00  (0.18, 5.43) | 1.98  (0.18, 21.80) | 0.20  (0.01, 4.02) | Wuling + SSRI |

**Outcome: Any gastrointestinal event**

| FEW |  |  |  |  |  |  |  |  |  |  |  |  |  |  |  |  |
| --- | --- | --- | --- | --- | --- | --- | --- | --- | --- | --- | --- | --- | --- | --- | --- | --- |
| 1.00 (0.52, 1.92) | FEW + SSRI |  |  |  |  |  |  |  |  |  |  |  |  |  |  |  |
| 0.25 (0.00, 13.01) | 0.25 (0.00, 13.73) | Ginkgo biloba extract + SNRI |  |  |  |  |  |  |  |  |  |  |  |  |  |  |
| 0.65 (0.03, 12.00) | 0.65 (0.03, 12.90) | 2.60 (0.18, 37.70) | Hypericum |  |  |  |  |  |  |  |  |  |  |  |  |  |
| 0.40 (0.07, 2.27) | 0.40 (0.06, 2.56) | 1.62 (0.03, 77.43) | 0.62 (0.04, 10.18) | Jie-Yu + SSRI |  |  |  |  |  |  |  |  |  |  |  |  |
| 0.93 (0.01,127.11) | 0.93 (0.01,132.73) | 3.75 (0.12,112.53) | 1.44 (0.03, 75.13) | 2.32 (0.02,294.51) | Morinda Oligosaccharides |  |  |  |  |  |  |  |  |  |  |  |
| 0.19 (0.00, 13.00) | 0.19 (0.00, 13.66) | 0.75 (0.07, 7.70) | 0.29 (0.01, 6.27) | 0.46 (0.01, 29.77) | 0.20 (0.01, 4.02) | Morinda Oligosaccharides + SNRI |  |  |  |  |  |  |  |  |  |  |
| 0.99 (0.19, 5.28) | 0.99 (0.16, 5.97) | 3.99 (0.09,185.95) | 1.53 (0.10, 24.21) | 2.47 (0.94, 6.50) | 1.06 (0.01,132.20) | 5.32 (0.08,332.87) | SJC |  |  |  |  |  |  |  |  |  |
| 0.21 (0.00, 10.71) | 0.21 (0.00, 11.31) | 0.83 (0.15, 4.68) | 0.32 (0.02, 4.55) | 0.52 (0.01, 24.38) | 0.22 (0.01, 6.57) | 1.11 (0.11, 11.17) | 0.21 (0.00, 9.62) | SJC + SNRI |  |  |  |  |  |  |  |  |
| 0.37 (0.07, 1.87) | 0.37 (0.06, 2.12) | 1.47 (0.03, 67.51) | 0.57 (0.04, 8.73) | 0.91 (0.37, 2.23) | 0.39 (0.00, 48.17) | 1.96 (0.03,121.00) | 0.37 (0.17, 0.80) | 1.77 (0.04, 80.00) | SJC + SSRI |  |  |  |  |  |  |  |
| 0.31 (0.01, 13.33) | 0.31 (0.01, 14.10) | 1.25 (0.36, 4.32) | 0.48 (0.04, 5.12) | 0.77 (0.02, 30.19) | 0.33 (0.01, 7.92) | 1.67 (0.23, 11.97) | 0.31 (0.01, 11.90) | 1.50 (0.45, 4.98) | 0.85 (0.02, 31.63) | SNRI |  |  |  |  |  |  |
| 0.33 (0.07, 1.59) | 0.33 (0.06, 1.81) | 1.34 (0.03, 59.68) | 0.52 (0.03, 7.62) | 0.83 (0.39, 1.77) | 0.36 (0.00, 42.85) | 1.79 (0.03,107.20) | 0.34 (0.18, 0.62) | 1.61 (0.04, 70.71) | 0.91 (0.56, 1.48) | 1.07 (0.03, 38.77) | SSRI |  |  |  |  |  |
| 0.33 (0.06, 1.89) | 0.33 (0.05, 2.13) | 1.34 (0.04, 47.18) | 0.52 (0.05, 5.41) | 0.83 (0.18, 3.78) | 0.36 (0.00, 35.64) | 1.79 (0.04, 86.29) | 0.34 (0.08, 1.43) | 1.61 (0.05, 55.85) | 0.91 (0.22, 3.70) | 1.07 (0.04, 30.20) | 1.00 (0.27, 3.72) | Usual care |  |  |  |  |
| 0.93 (0.14, 6.12) | 0.93 (0.13, 6.84) | 3.76 (0.07,192.76) | 1.44 (0.08, 26.01) | 2.33 (0.64, 8.49) | 1.00 (0.01,134.47) | 5.01 (0.07,342.78) | 0.94 (0.28, 3.17) | 4.51 (0.09,228.50) | 2.55 (0.80, 8.12) | 3.01 (0.07,126.23) | 2.80 (0.98, 8.01) | 2.80 (0.52, 15.07) | Wuling |  |  |  |
| 0.44 (0.01, 13.22) | 0.44 (0.01, 14.06) | 1.77 (0.07, 43.10) | 0.68 (0.12, 3.89) | 1.10 (0.04, 29.62) | 0.47 (0.01, 35.62) | 2.37 (0.07, 81.48) | 0.44 (0.02, 11.64) | 2.13 (0.09, 50.94) | 1.20 (0.05, 30.87) | 1.42 (0.08, 26.83) | 1.32 (0.05, 32.66) | 1.32 (0.07, 24.64) | 0.47 (0.02, 13.80) | Wuling + Hypericum |  |  |
| 0.93 (0.01,126.87) | 0.93 (0.01,132.47) | 3.75 (0.13,112.22) | 1.44 (0.03, 74.95) | 2.32 (0.02,293.93) | 1.00 (0.01, 88.01) | 5.00 (0.12,208.08) | 0.94 (0.01,116.66) | 4.50 (0.15,132.77) | 2.55 (0.02,311.50) | 3.00 (0.13, 71.04) | 2.79 (0.02,333.53) | 2.79 (0.03,277.40) | 1.00 (0.01,133.53) | 2.11 (0.03,158.83) | Wuling + SNRI |  |
| 0.37 (0.07, 2.03) | 0.37 (0.06, 2.30) | 1.51 (0.03, 70.94) | 0.58 (0.04, 9.26) | 0.93 (0.34, 2.54) | 0.40 (0.00, 50.36) | 2.01 (0.03,126.92) | 0.38 (0.16, 0.92) | 1.81 (0.04, 84.08) | 1.02 (0.45, 2.31) | 1.21 (0.03, 46.24) | 1.12 (0.58, 2.16) | 1.12 (0.26, 4.88) | 0.40 (0.14, 1.13) | 0.85 (0.03, 22.47) | 0.40 (0.00, 50.26) | Wuling + SSRI |

**Outcome: Any nervous system event**

| FEW |  |  |  |  |  |  |  |  |  |  |  |  |  |  |  |
| --- | --- | --- | --- | --- | --- | --- | --- | --- | --- | --- | --- | --- | --- | --- | --- |
| 1.00 (0.22, 4.63) | FEW + SSRI |  |  |  |  |  |  |  |  |  |  |  |  |  |  |
| 0.24 (0.00, 35.68) | 0.24 (0.00, 44.86) | Ginkgo biloba extract + SNRI |  |  |  |  |  |  |  |  |  |  |  |  |  |
| 0.20 (0.00, 8.87) | 0.20 (0.00, 11.93) | 0.82 (0.03, 21.31) | Hypericum |  |  |  |  |  |  |  |  |  |  |  |  |
| 0.09 (0.00, 2.23) | 0.09 (0.00, 3.17) | 0.40 (0.00, 34.51) | 0.49 (0.02, 10.22) | Jie-Yu + SSRI |  |  |  |  |  |  |  |  |  |  |  |
| 0.56 (0.00, 162.90) | 0.56 (0.00, 199.66) | 2.37 (0.14, 39.90) | 2.88 (0.04, 190.27) | 5.93 (0.03,1054.90) | Morinda Oligosaccharides |  |  |  |  |  |  |  |  |  |  |
| 0.28 (0.00, 68.57) | 0.28 (0.00, 84.57) | 1.19 (0.10, 13.86) | 1.44 (0.03, 75.15) | 2.97 (0.02, 436.70) | 0.50 (0.05, 5.27) | Morinda Oligosaccharides + SNRI |  |  |  |  |  |  |  |  |  |
| 1.03 (0.05, 23.48) | 1.03 (0.03, 33.49) | 4.34 (0.05, 366.21) | 5.26 (0.26, 107.45) | 10.83 (1.95, 60.00) | 1.83 (0.01, 319.02) | 3.65 (0.03, 527.91) | SJC |  |  |  |  |  |  |  |  |
| 0.68 (0.00, 109.62) | 0.68 (0.00, 137.38) | 2.85 (0.76, 10.65) | 3.46 (0.12, 100.24) | 7.12 (0.08, 667.55) | 1.20 (0.06, 22.99) | 2.40 (0.18, 32.54) | 0.66 (0.01, 60.42) | SJC + SNRI |  |  |  |  |  |  |  |
| 0.10 (0.00, 2.17) | 0.10 (0.00, 3.10) | 0.41 (0.00, 33.98) | 0.49 (0.02, 9.94) | 1.01 (0.19, 5.50) | 0.17 (0.00, 29.64) | 0.34 (0.00, 49.03) | 0.09 (0.02, 0.48) | 0.14 (0.00, 12.98) | SJC + SSRI |  |  |  |  |  |  |
| 0.56 (0.00, 80.68) | 0.56 (0.00, 101.66) | 2.37 (1.18, 4.78) | 2.88 (0.12, 69.02) | 5.93 (0.07, 483.80) | 1.00 (0.07, 15.38) | 2.00 (0.19, 21.09) | 0.55 (0.01, 43.76) | 0.83 (0.27, 2.55) | 5.86 (0.07, 464.33) | SNRI |  |  |  |  |  |
| 0.11 (0.01, 2.02) | 0.11 (0.00, 2.95) | 0.47 (0.01, 33.79) | 0.57 (0.04, 9.16) | 1.17 (0.34, 4.07) | 0.20 (0.00, 30.11) | 0.39 (0.00, 49.57) | 0.11 (0.03, 0.35) | 0.16 (0.00, 12.94) | 1.16 (0.37, 3.62) | 0.20 (0.00, 13.43) | SSRI |  |  |  |  |
| 0.10 (0.00, 2.04) | 0.10 (0.00, 2.94) | 0.43 (0.01, 23.51) | 0.52 (0.05, 5.41) | 1.06 (0.15, 7.37) | 0.18 (0.00, 21.85) | 0.36 (0.00, 35.62) | 0.10 (0.01, 0.65) | 0.15 (0.00, 9.06) | 1.05 (0.16, 6.82) | 0.18 (0.00, 9.31) | 0.91 (0.21, 4.00) | Usual care |  |  |  |
| 0.33 (0.01, 12.68) | 0.33 (0.01, 17.24) | 1.37 (0.01, 171.45) | 1.67 (0.05, 58.98) | 3.43 (0.27, 44.35) | 0.58 (0.00, 141.87) | 1.16 (0.01, 237.62) | 0.32 (0.03, 3.95) | 0.48 (0.00, 65.03) | 3.39 (0.28, 41.69) | 0.58 (0.00, 68.59) | 2.93 (0.31, 27.40) | 3.23 (0.22, 47.24) | Wuling |  |  |
| 0.20 (0.00, 21.93) | 0.20 (0.00, 27.97) | 0.84 (0.01, 59.28) | 1.02 (0.07, 15.85) | 2.10 (0.03, 126.79) | 0.35 (0.00, 53.03) | 0.71 (0.01, 87.22) | 0.19 (0.00, 11.45) | 0.30 (0.00, 22.72) | 2.08 (0.04, 121.43) | 0.35 (0.01, 23.55) | 1.80 (0.04, 89.22) | 1.98 (0.05, 73.37) | 0.61 (0.01, 55.14) | Wuling + Hypericum |  |
| 0.24 (0.01, 5.48) | 0.24 (0.01, 7.82) | 1.01 (0.01, 85.38) | 1.22 (0.06, 25.10) | 2.51 (0.45, 14.08) | 0.42 (0.00, 74.33) | 0.85 (0.01, 123.02) | 0.23 (0.04, 1.23) | 0.35 (0.00, 32.60) | 2.48 (0.48, 12.91) | 0.42 (0.01, 34.01) | 2.15 (0.65, 7.05) | 2.37 (0.35, 15.85) | 0.73 (0.08, 6.85) | 1.20 (0.02, 70.78) | Wuling + SSRI |

## 7.4 Forest plots

**Footnotes: Wuling = Wuling Capture; SJC= Shugan jieyu Capture; SSRI=** **selective serotonin reuptake inhibitors; SNRI =** **serotonin and noradrenaline reuptake inhibitors; Jie-Yu=Jie-Yu Pills FEW=Free and Easy Wander;RR= relative risk;** **MD = mean difference**

**Outcome: Response rate**


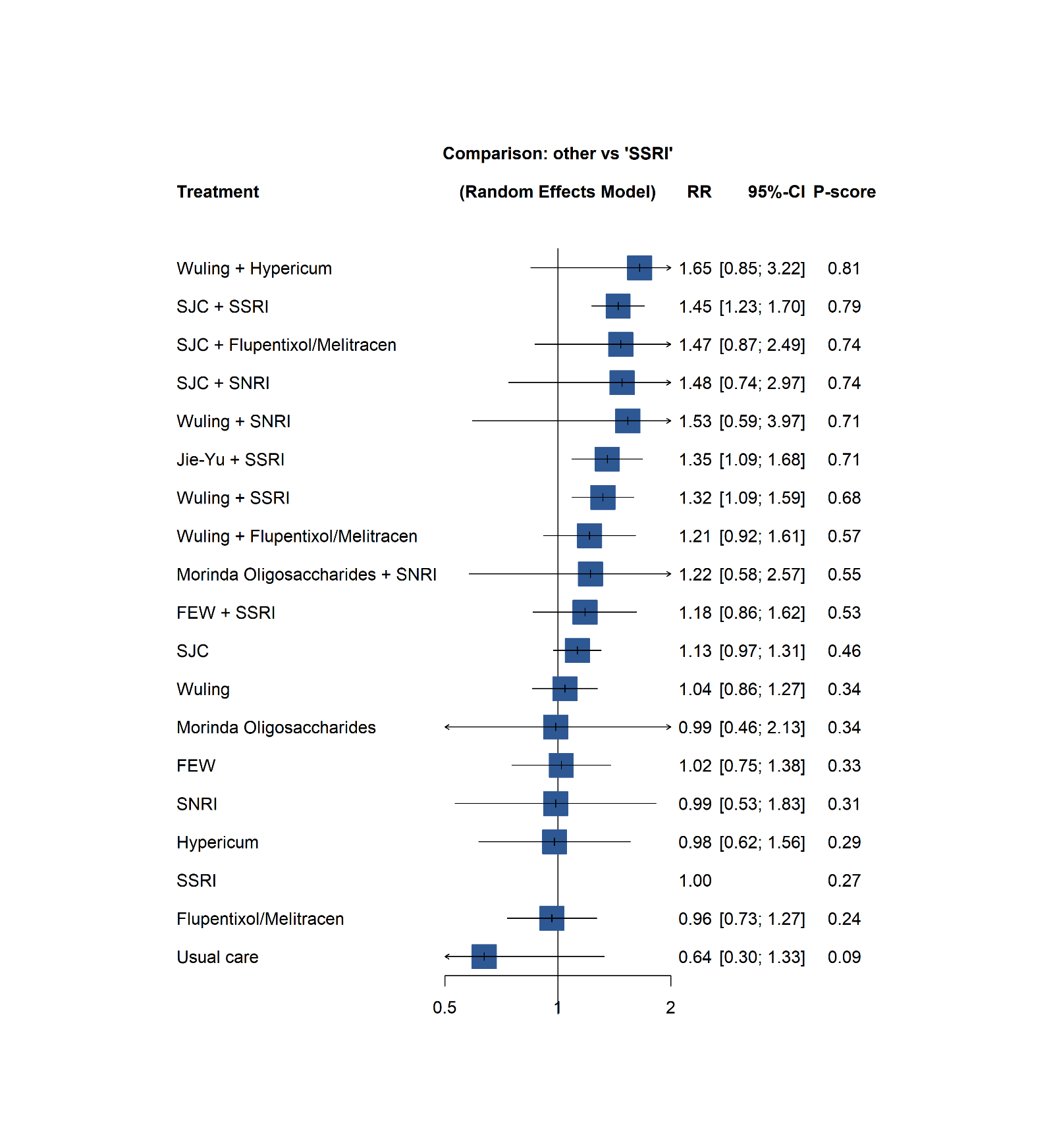


**Outcome: Mean changes in HAMD score from baseline**


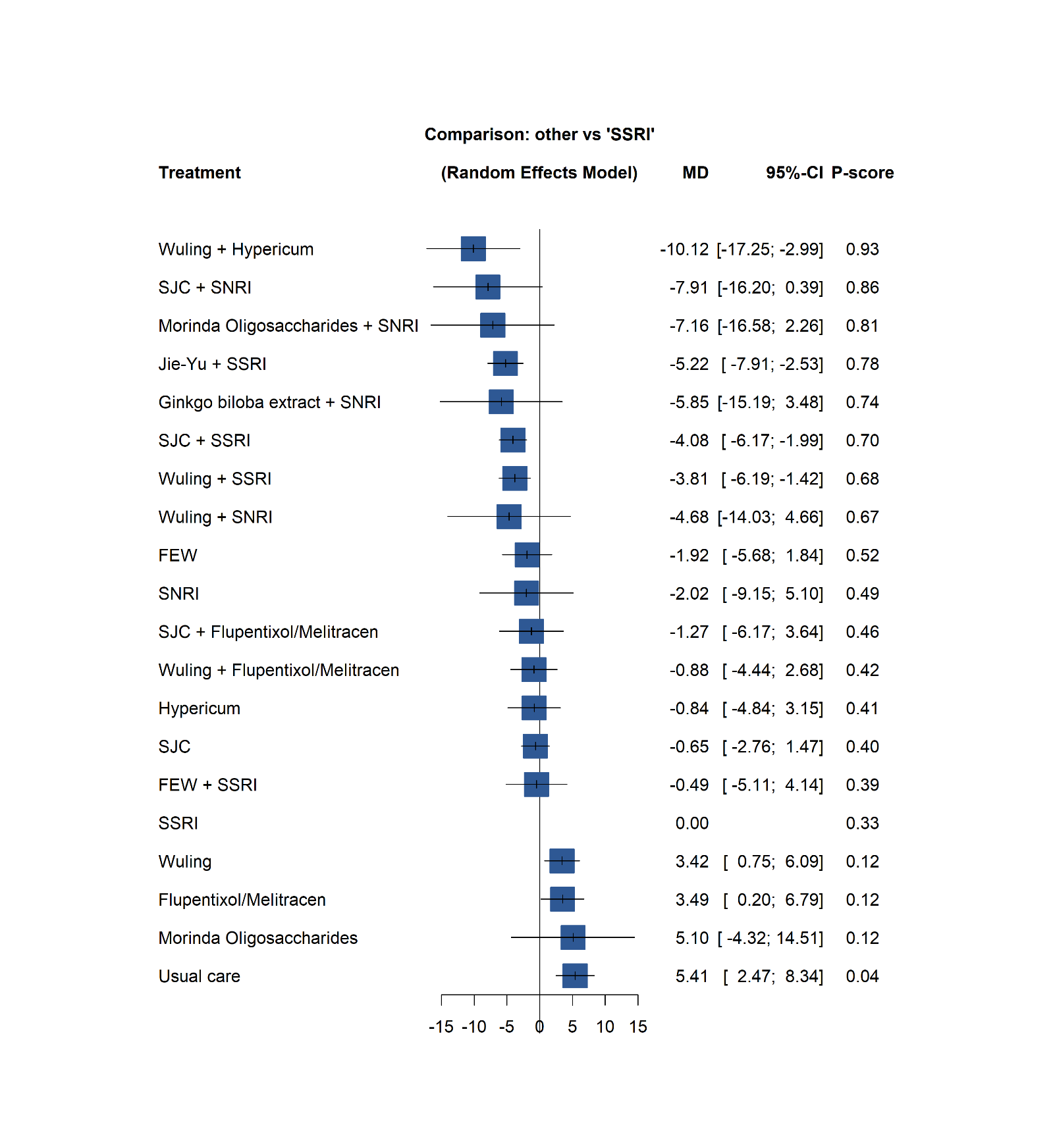


**Outcome: All-cause drop out**
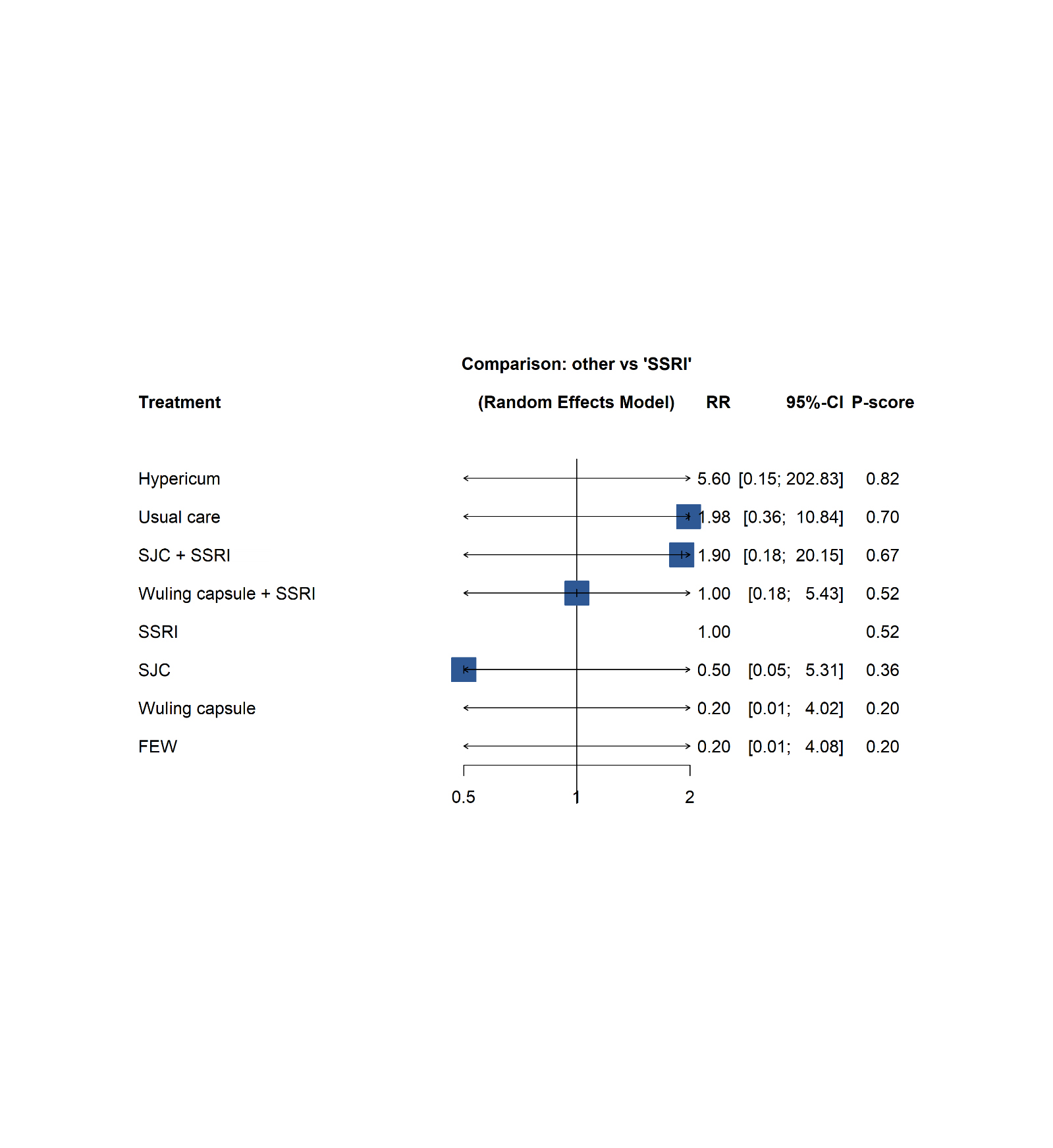


**Outcome: Mean changes in NIHSS score from baseline**


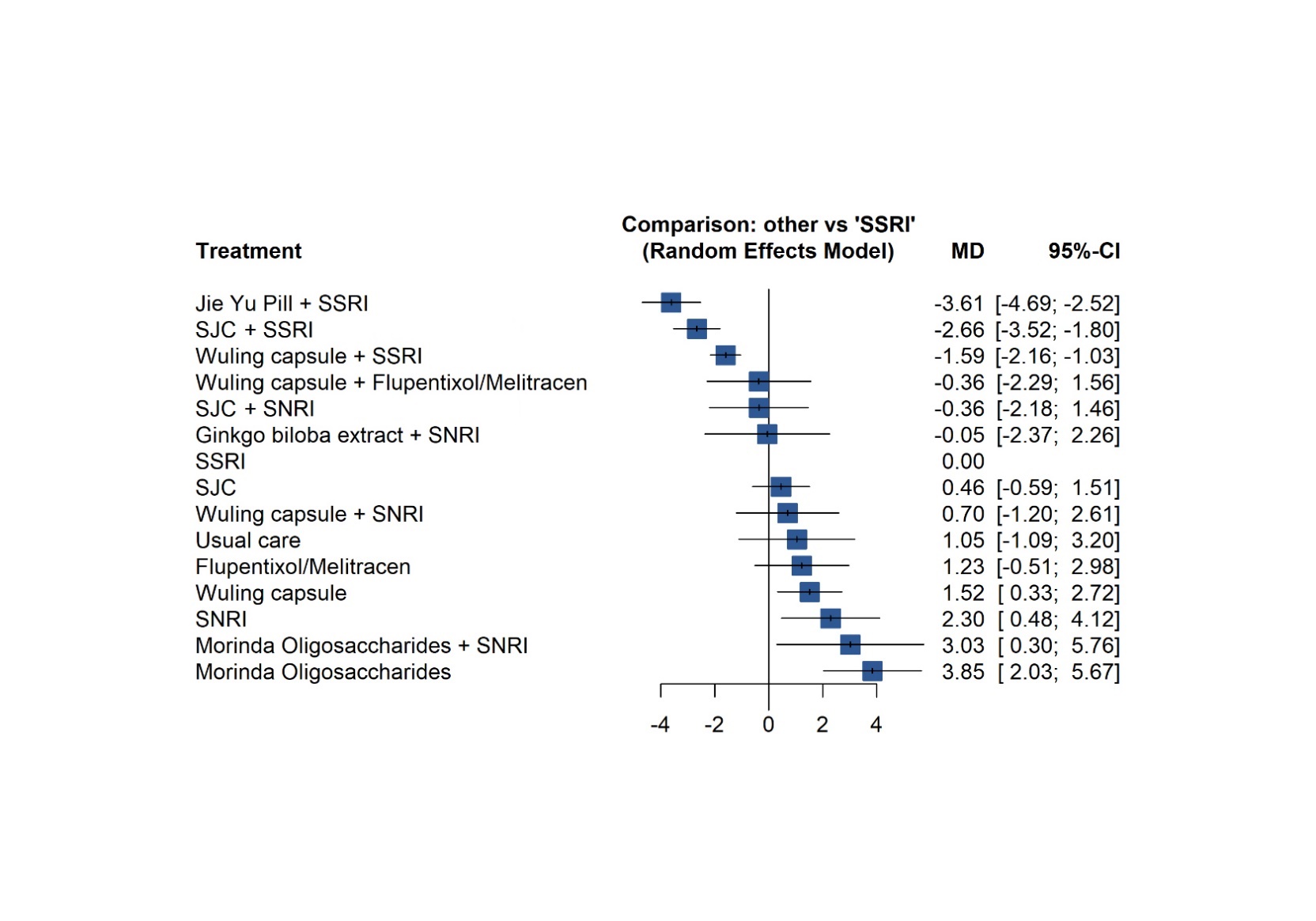


**Outcome: Any gastrointestinal event**

**
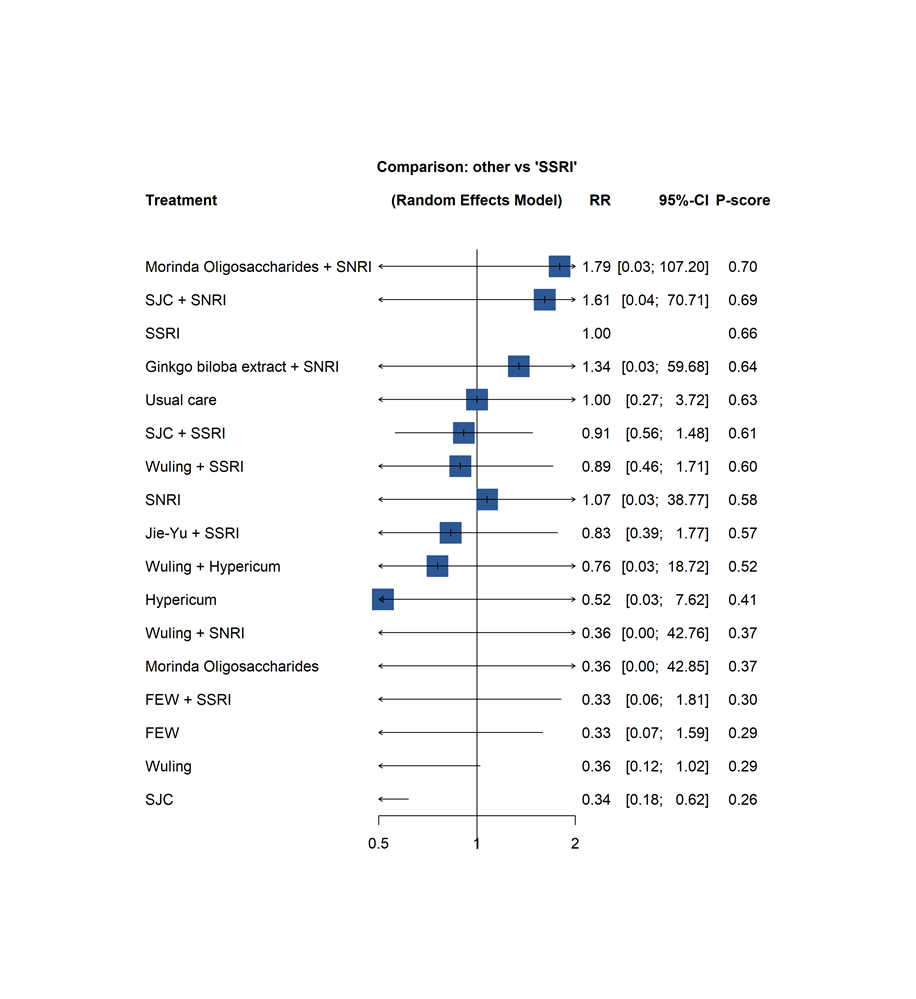
**

**Outcome: Any nervous system event**


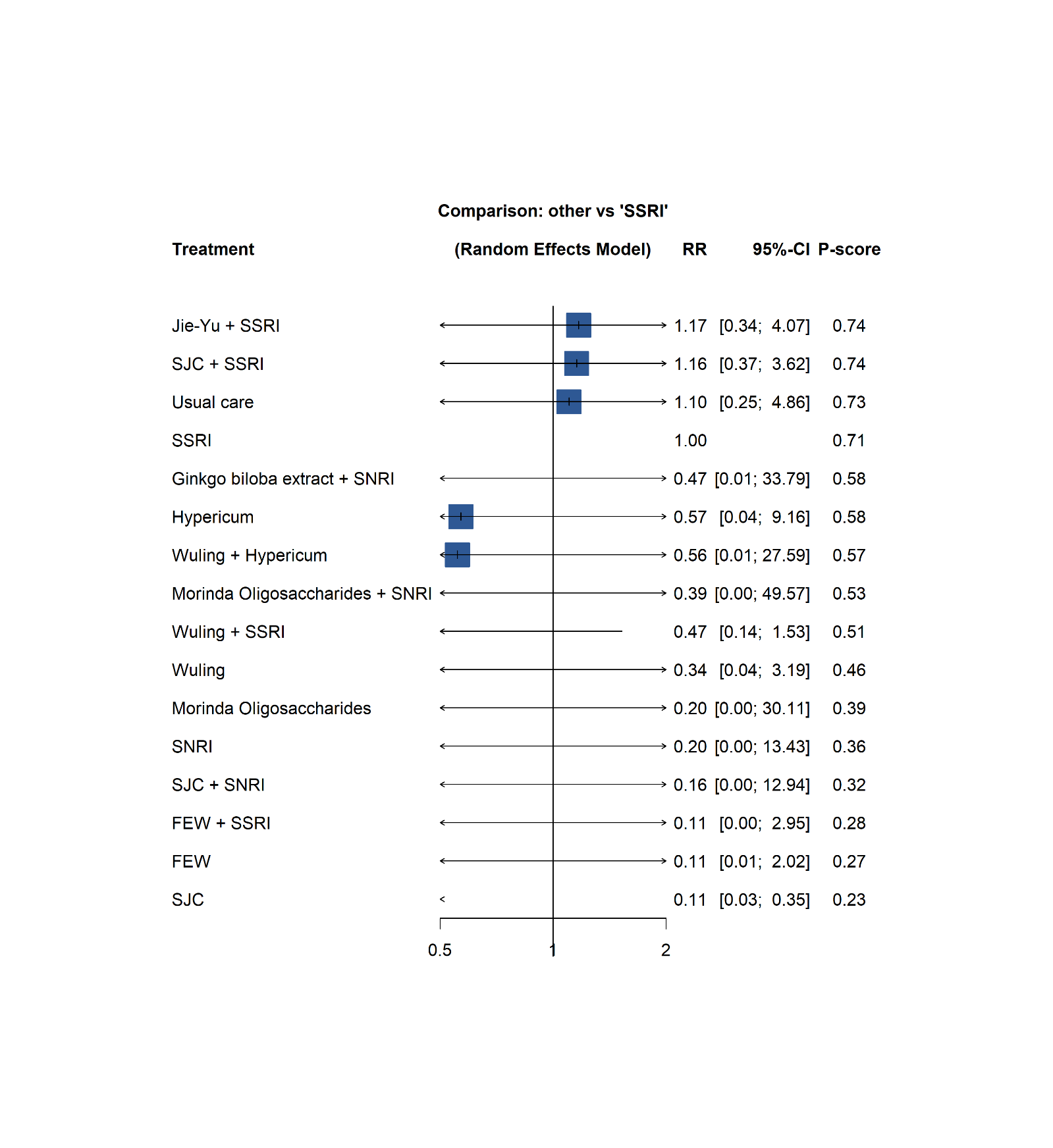


## 7.5 Heterogeneity and inconsistency (incoherence)

**Heterogeneity assessments**

Notes: We also considered the heterogeneity in the network meta-analysis. The analysis performed a design-based decomposition of generalised Cochran’s Q statistic for assessing the heterogeneity in the whole network, the heterogeneity within designs, and the heterogeneity/inconsistency between designs.

| Outcomes | Design-based Q statistic, comparisons, and overall statement | Q statistic | Degree of freedom | P value |
| --- | --- | --- | --- | --- |
| **Response rate** | **Design-specific decomposition of within-designs Q statistic** | | | |
|  | SJC + SNRI vs SNRI | 0.06 | 1 | 0.8007 |
|  | SSRI vs Jie-Yu + SSRI | 2.97 | 4 | 0.5623 |
|  | SSRI vs SJC | 6.01 | 5 | 0.3049 |
|  | SSRI vs SJC + SSRI | 2.01 | 4 | 0.7340 |
|  | SSRI vs Wuling + SSRI | 1.43 | 1 | 0.2311 |
|  | Flupentixol/Melitracen vs Wuling vs Wuling + Flupentixol/Melitracen | 1.92 | 2 | 0.3826 |
|  | SSRI vs Wuling vs Wuling + SSRI | 0.41 | 2 | 0.8164 |
|  | **Between-designs Q statistic after detaching of single designs** | | | |
|  | FEW vs FEW + SSRI | 26.80 | 7 | 0.0004 |
|  | Flupentixol/Melitracen vs SJC | 23.05 | 7 | 0.0017 |
|  | Flupentixol/Melitracen vs Wuling + Flupentixol/Melitracen | 26.79 | 7 | 0.0004 |
|  | SJC vs Usual care | 22.08 | 7 | 0.0025 |
|  | SSRI vs FEW + SSRI | 26.80 | 7 | 0.0004 |
|  | SSRI vs SJC | 27.69 | 7 | 0.0003 |
|  | SSRI vs SJC + SSRI | 27.72 | 7 | 0.0002 |
|  | SSRI vs Wuling | 20.87 | 7 | 0.0040 |
|  | SSRI vs Wuling + SSRI | 18.50 | 7 | 0.0099 |
|  | Flupentixol/Melitracen vs Wuling vs Wuling + Flupentixol/Melitracen | 22.65 | 6 | 0.0009 |
|  | SSRI vs FEW vs Usual care | 21.04 | 6 | 0.0018 |
|  | SSRI vs SJC vs SJC + SSRI | 26.61 | 6 | 0.0002 |
|  | SSRI vs Wuling vs Wuling + SSRI | 17.00 | 6 | 0.0093 |
|  | Q statistic to assess consistency under the assumption of a full design-by-treatment interaction random effects model: between designs Q statistic, 27.73; degree of freedom, 8; p value, 0.0005; tau. Within, 0.1234; tau^2^. Within, 0.0152. | | | |
| **Mean changes in HAMD score from baseline** | **Design-specific decomposition of within-designs Q statistic** | | | |
|  | Flupentixol/Melitracen vs Wuling + Flupentixol/Melitracen | 29.99 | 2 | < 0.0001 |
|  | Hypericum vs Usual care | 22.76 | 1 | < 0.0001 |
|  | SJC + SNRI vs SNRI | 1.66 | 1 | 0.1980 |
|  | SJC vs Usual care | 232.71 | 1 | < 0.0001 |
|  | SSRI vs Jie-Yu + SSRI | 13.44 | 4 | 0.0093 |
|  | SSRI vs SJC | 31.46 | 4 | < 0.0001 |
|  | SSRI vs SJC + SSRI | 408.30 | 6 | < 0.0001 |
|  | SSRI vs Wuling + SSRI | 4.63 | 3 | 0.2012 |
|  | Flupentixol/Melitracen vs Wuling vs Wuling + Flupentixol/Melitracen | 11.26 | 2 | 0.0036 |
|  | SSRI vs Wuling vs Wuling + SSRI | 22.09 | 2 | < 0.0001 |
|  | **Between-designs Q statistic after detaching of single designs** | | | |
|  | FEW vs FEW + SSRI | 347.93 | 12 | < 0.0001 |
|  | FEW vs Wuling | 384.03 | 12 | < 0.0001 |
|  | Flupentixol/Melitracen vs SJC | 389.13 | 12 | < 0.0001 |
|  | Flupentixol/Melitracen vs SJC + Flupentixol/Melitracen | 390.28 | 12 | < 0.0001 |
|  | Flupentixol/Melitracen vs Wuling + Flupentixol/Melitracen | 390.68 | 12 | < 0.0001 |
|  | Hypericum vs Usual care | 386.06 | 12 | < 0.0001 |
|  | SJC vs SJC + Flupentixol/Melitracen | 390.28 | 12 | < 0.0001 |
|  | SJC vs Usual care | 389.76 | 12 | < 0.0001 |
|  | SSRI vs FEW + SSRI | 347.93 | 12 | < 0.0001 |
|  | SSRI vs Hypericum | 386.06 | 12 | < 0.0001 |
|  | SSRI vs SJC | 391.80 | 12 | < 0.0001 |
|  | SSRI vs SJC + SSRI | 392.03 | 12 | < 0.0001 |
|  | SSRI vs Wuling | 164.64 | 12 | < 0.0001 |
|  | SSRI vs Wuling + SSRI | 340.57 | 12 | < 0.0001 |
|  | Flupentixol/Melitracen vs Usual care vs Wuling + Flupentixol/Melitracen | 372.80 | 11 | < 0.0001 |
|  | Flupentixol/Melitracen vs Wuling vs Wuling + Flupentixol/Melitracen | 339.59 | 11 | < 0.0001 |
|  | SSRI vs FEW vs Usual care | 343.93 | 11 | < 0.0001 |
|  | SSRI vs SJC vs SJC + SSRI | 387.29 | 11 | < 0.0001 |
|  | SSRI vs Wuling vs Wuling + SSRI | 252.09 | 11 | < 0.0001 |
|  | Q statistic to assess consistency under the assumption of a full design-by-treatment interaction random effects model: between designs Q statistic, 392.06; degree of freedom, 13; p value, < 0.0001; tau. Within, 2.9604; tau^2^. Within, 8.7641. | | | |
| **Any gastrointestinal event** | **Design-specific decomposition of within-designs Q statistic** | | | |
|  | SSRI vs Jie-Yu + SSRI | 1.18 | 2 | 0.5534 |
|  | SSRI vs SJC | 2.07 | 3 | 0.5580 |
|  | SSRI vs SJC + SSRI | 1.86 | 3 | 0.6020 |
|  | **Between-designs Q statistic after detaching of single designs** | | | |
|  | SSRI vs Wuling + SSRI | 0.00 | 0 | -- |
|  | Q statistic to assess consistency under the assumption of a full design-by-treatment interaction random effects model: between designs Q statistic, 1.87; degree of freedom, 1; p value, 0.1712; tau. Within, 0; tau^2^. Within, 0. | | | |
| **Any nervous system event** | **Design-specific decomposition of within-designs Q statistic** | | | |
|  | SSRI vs Jie-Yu + SSRI | 0.33 | 2 | 0.8464 |
|  | SSRI vs SJC | 0.12 | 3 | 0.9894 |
|  | SSRI vs SJC + SSRI | 0.82 | 2 | 0.6622 |
|  | **Between-designs Q statistic after detaching of single designs** | | | |
|  | SSRI vs Wuling + SSRI | 0.00 | 0 | -- |
|  | Q statistic to assess consistency under the assumption of a full design-by-treatment interaction random effects model: between designs Q statistic, 1.01; degree of freedom, 1; p value, 0.3137; tau. Within, 0; tau^2^. Within, 0. | | | |

**Inconsistency (incoherence) assessments**

| Comparisons | Direct estimates | Indirect estimates | Network estimates | Inconsistency P-value |
| --- | --- | --- | --- | --- |
| Response rate | | | | |
| FEW vs FEW + SSRI | 0.93 (0.65, 1.33) | 0.73 (0.43, 1.27) | 0.86 (0.64, 1.17) | 0.482602111 |
| FEW vs SSRI | 0.92 (0.64, 1.33) | 1.27 (0.74, 2.18) | 1.02 (0.75, 1.38) | 0.341120645 |
| FEW vs Usual care | 36.8 (2.31, 585.65) | 1.21 (0.53, 2.77) | 1.6 (0.73, 3.55) | 0.020561182 |
| FEW + SSRI vs SSRI | 1.29 (0.86, 1.94) | 1.02 (0.61, 1.71) | 1.18 (0.86, 1.62) | 0.482602111 |
| Flupentixol/Melitracen vs SJC | 0.59 (0.37, 0.95) | 1.06 (0.74, 1.53) | 0.86 (0.64, 1.14) | 0.053481524 |
| Flupentixol/Melitracen vs Wuling | 1.01 (0.77, 1.31) | 0.74 (0.48, 1.14) | 0.92 (0.73, 1.16) | 0.234255024 |
| Flupentixol/Melitracen vs Wuling + Flupentixol/Melitracen | 0.83 (0.68, 1) | 0.31 (0.12, 0.79) | 0.79 (0.66, 0.96) | 0.044125166 |
| SJC vs SJC + SSRI | 0.71 (0.49, 1.04) | 0.81 (0.63, 1.03) | 0.78 (0.64, 0.96) | 0.580958209 |
| SJC vs SSRI | 1.09 (0.94, 1.28) | 1.43 (0.92, 2.23) | 1.13 (0.97, 1.31) | 0.258539391 |
| SJC vs Usual care | 1.4 (0.66, 2.97) | 43.6 (2.74, 694.36) | 1.77 (0.86, 3.66) | 0.018777618 |
| SJC + SSRI vs SSRI | 1.42 (1.2, 1.68) | 1.77 (1, 3.13) | 1.45 (1.23, 1.7) | 0.468617753 |
| Usual care vs SSRI | 0.03 (0, 0.4) | 0.81 (0.38, 1.75) | 0.64 (0.3, 1.33) | 0.017471938 |
| Wuling + SSRI vs SSRI | 1.3 (1.07, 1.58) | 1.56 (0.76, 3.22) | 1.32 (1.09, 1.59) | 0.635277534 |
| Wuling vs Wuling + Flupentixol/Melitracen | 0.8 (0.63, 1.02) | 1.38 (0.76, 2.51) | 0.86 (0.69, 1.08) | 0.096912647 |
| Wuling vs Wuling + SSRI | 0.84 (0.66, 1.07) | 0.64 (0.4, 1.03) | 0.79 (0.64, 0.99) | 0.32665711 |
| Mean changes in HAMD score from baseline | | | | |
| FEW vs FEW + SSRI | -4.98 (-11.15, 1.19) | 3.43 (-3.8, 10.66) | -1.44 (-6.13, 3.26) | 0.082875838 |
| FEW vs SSRI | 0.4 (-5.46, 6.26) | -3.55 (-8.45, 1.35) | -1.92 (-5.68, 1.84) | 0.311307207 |
| FEW vs Usual care | -5 (-10.85, 0.85) | -9.72 (-15.64, -3.79) | -7.33 (-11.49, -3.16) | 0.267262701 |
| FEW vs Wuling | -5.24 (-11.26, 0.78) | -5.42 (-10.62, -0.22) | -5.34 (-9.28, -1.41) | 0.964235328 |
| FEW vs FEW + SSRI | -4.98 (-11.15, 1.19) | 3.43 (-3.8, 10.66) | -1.44 (-6.13, 3.26) | 0.082875838 |
| FEW vs SSRI | 0.4 (-5.46, 6.26) | -3.55 (-8.45, 1.35) | -1.92 (-5.68, 1.84) | 0.311307207 |
| FEW vs Usual care | -5 (-10.85, 0.85) | -9.72 (-15.64, -3.79) | -7.33 (-11.49, -3.16) | 0.267262701 |
| FEW vs Wuling | -5.24 (-11.26, 0.78) | -5.42 (-10.62, -0.22) | -5.34 (-9.28, -1.41) | 0.964235328 |
| FEW + SSRI vs SSRI | -3.72 (-9.61, 2.17) | 4.69 (-2.77, 12.15) | -0.49 (-5.11, 4.14) | 0.082875838 |
| Flupentixol/Melitracen vs SJC | 4 (-2.2, 10.2) | 4.19 (0.35, 8.04) | 4.14 (0.87, 7.41) | 0.958555557 |
| Flupentixol/Melitracen vs SJC + Flupentixol/Melitracen | 4.78 (-1.44, 11) | 4.73 (-2.36, 11.82) | 4.76 (0.09, 9.43) | 0.992247368 |
| Flupentixol/Melitracen vs Usual care | -3.32 (-9.17, 2.53) | -1.13 (-5.49, 3.22) | -1.91 (-5.41, 1.58) | 0.556743049 |
| Flupentixol/Melitracen vs Wuling | 1.31 (-2.96, 5.58) | -1.41 (-6.1, 3.27) | 0.07 (-3.08, 3.23) | 0.400145201 |
| Flupentixol/Melitracen vs Wuling + Flupentixol/Melitracen | 4.38 (1.92, 6.85) | 4.08 (-8.99, 17.15) | 4.37 (1.95, 6.79) | 0.964290063 |
| Hypericum vs SSRI | -1 (-7.09, 5.09) | -0.72 (-6.02, 4.57) | -0.84 (-4.84, 3.15) | 0.94653089 |
| Hypericum vs Usual care | -6.17 (-10.39, -1.95) | -6.45 (-13.33, 0.43) | -6.25 (-9.85, -2.65) | 0.94653089 |
| SJC vs SJC + Flupentixol/Melitracen | 0.6 (-5.55, 6.75) | 0.65 (-6.5, 7.79) | 0.62 (-4.04, 5.28) | 0.992247368 |
| SJC vs SJC + SSRI | 2.64 (-3.28, 8.56) | 3.67 (0.43, 6.92) | 3.43 (0.59, 6.28) | 0.764602878 |
| SJC vs SSRI | -0.33 (-2.77, 2.1) | -1.62 (-5.91, 2.66) | -0.65 (-2.76, 1.47) | 0.607523218 |
| SJC vs Usual care | -6.89 (-11.02, -2.76) | -5.24 (-9.3, -1.18) | -6.05 (-8.95, -3.16) | 0.576334926 |
| SJC + SSRI vs SSRI | -4.15 (-6.27, -2.03) | -2.13 (-13.34, 9.08) | -4.08 (-6.17, -1.99) | 0.727905028 |
| Usual care vs SSRI | 5.4 (-0.44, 11.24) | 5.41 (2.01, 8.81) | 5.41 (2.47, 8.34) | 0.99826838 |
| Wuling vs SSRI | 2.44 (-1.04, 5.92) | 4.84 (0.66, 9.02) | 3.42 (0.75, 6.09) | 0.386887656 |
| Wuling + SSRI vs SSRI | -3.01 (-5.46, -0.56) | -18.64 (-29.21, -8.06) | -3.81 (-6.19, -1.42) | 0.004789603 |
| Usual care vs Wuling + Flupentixol/Melitracen | 4.89 (-0.97, 10.75) | 7.22 (2.43, 12) | 6.29 (2.58, 9.99) | 0.54663187 |
| Wuling vs Wuling + Flupentixol/Melitracen | 5.44 (1.16, 9.72) | 2.56 (-2.73, 7.85) | 4.3 (0.98, 7.63) | 0.406602288 |
| Wuling vs Wuling + SSRI | 11.35 (7.03, 15.67) | 2.39 (-2.29, 7.07) | 7.23 (4.05, 10.4) | 0.005829779 |
| Any gastrointestinal event | | | | |
| Wuling vs SSRI | 0.4 (0.14, 1.16) | 0 (0, 3.02) | 0.36 (0.12, 1.02) | 0.171174519 |
| Wuling vs Wuling + SSRI | 0.36 (0.13, 1.04) | 80.23 (0.04, 169894.61) | 0.4 (0.14, 1.13) | 0.171174519 |
| Any nervous system event | | | | |
| Wuling vs SSRI | 0.5 (0.05, 5.27) | 0.01 (0, 12.79) | 0.34 (0.04, 3.19) | 0.313749201 |
| Wuling vs Wuling + SSRI | 0.5 (0.05, 5.27) | 23.08 (0.02, 27238.89) | 0.73 (0.08, 6.85) | 0.313749201 |

## 7.6 Comparison-adjusted funnel plots

**Outcome: Response rate**


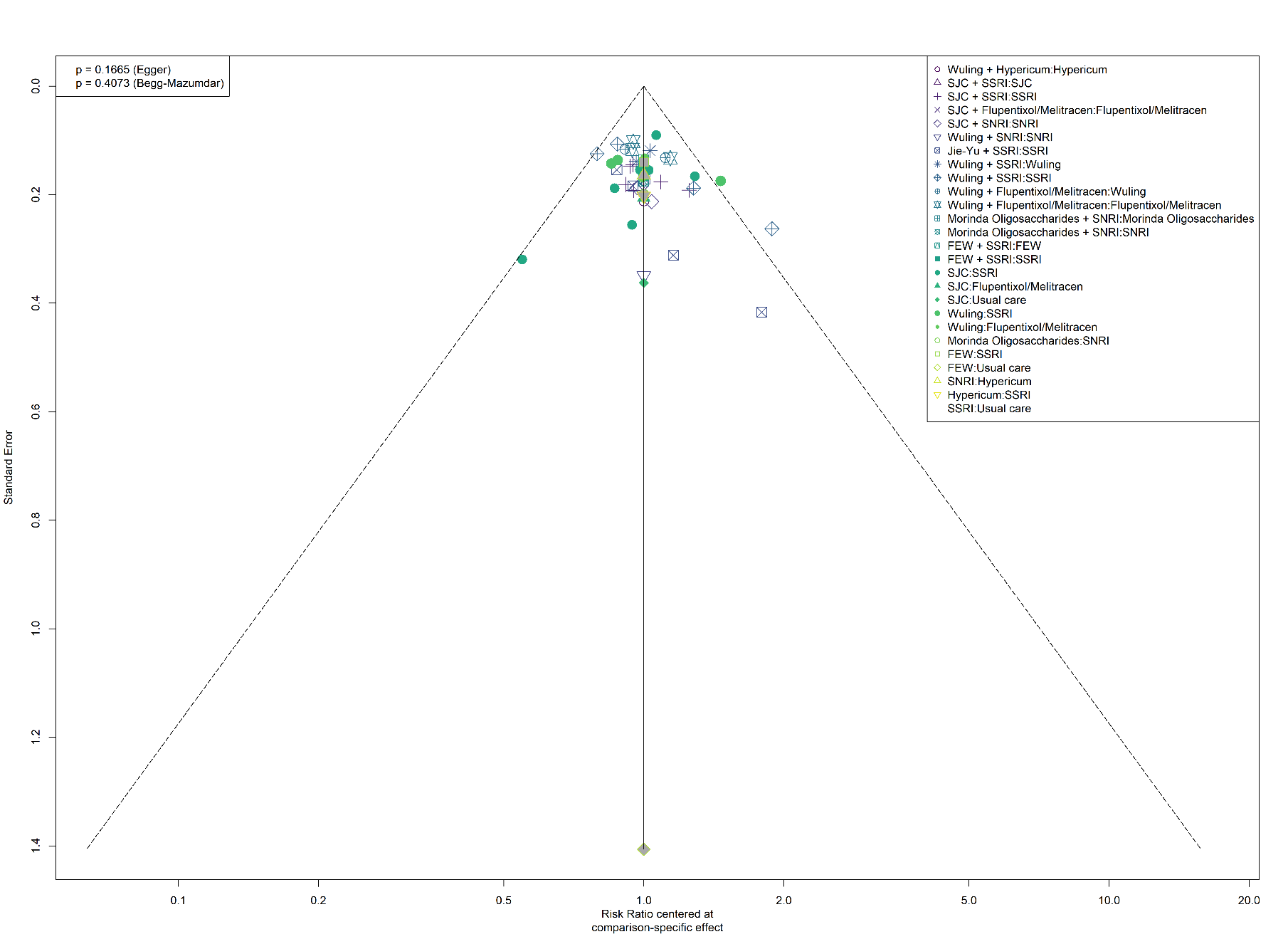


**Outcome: Mean changes in HAMD score from baseline**


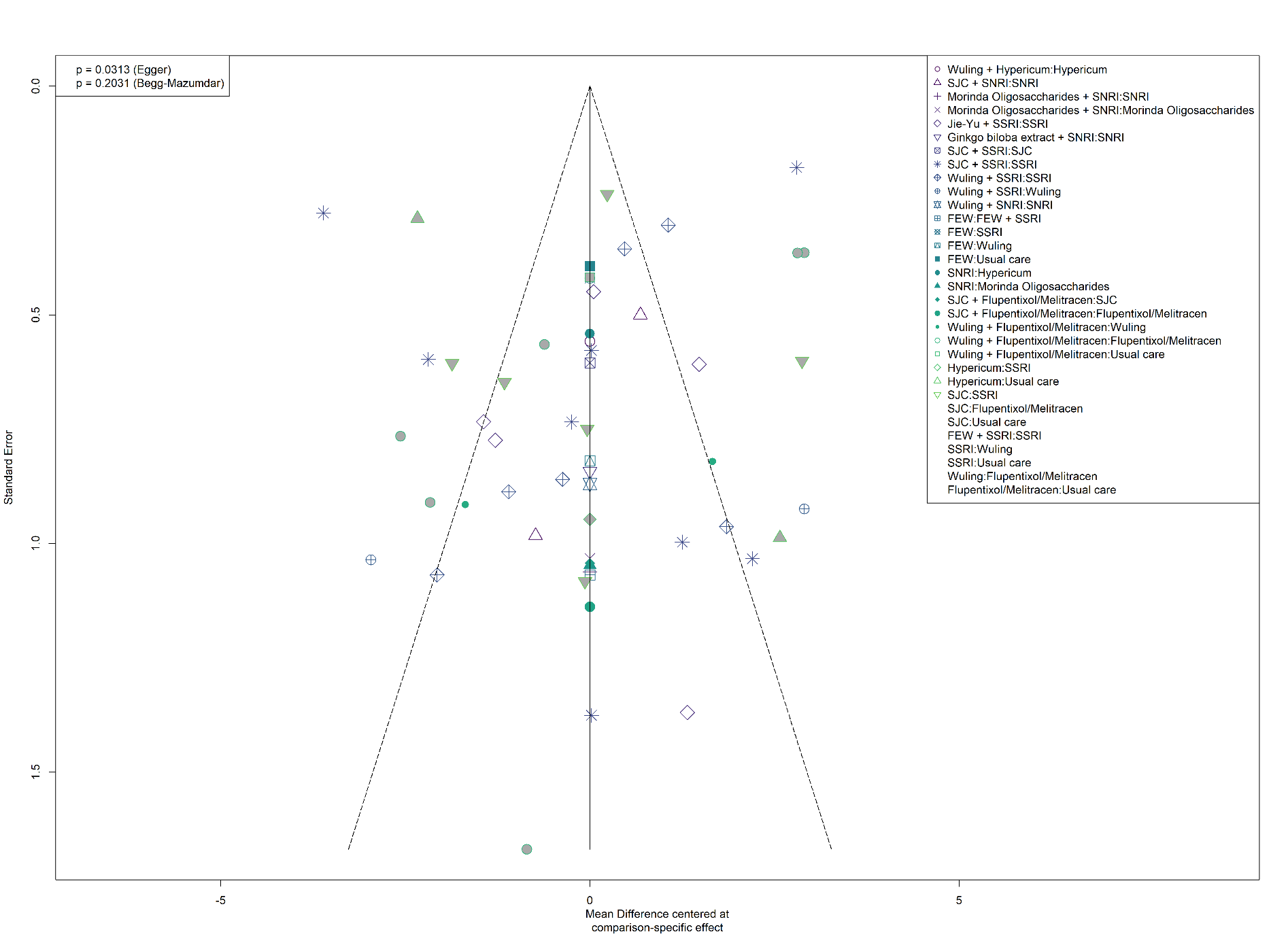


**Outcome: All-cause drop out**


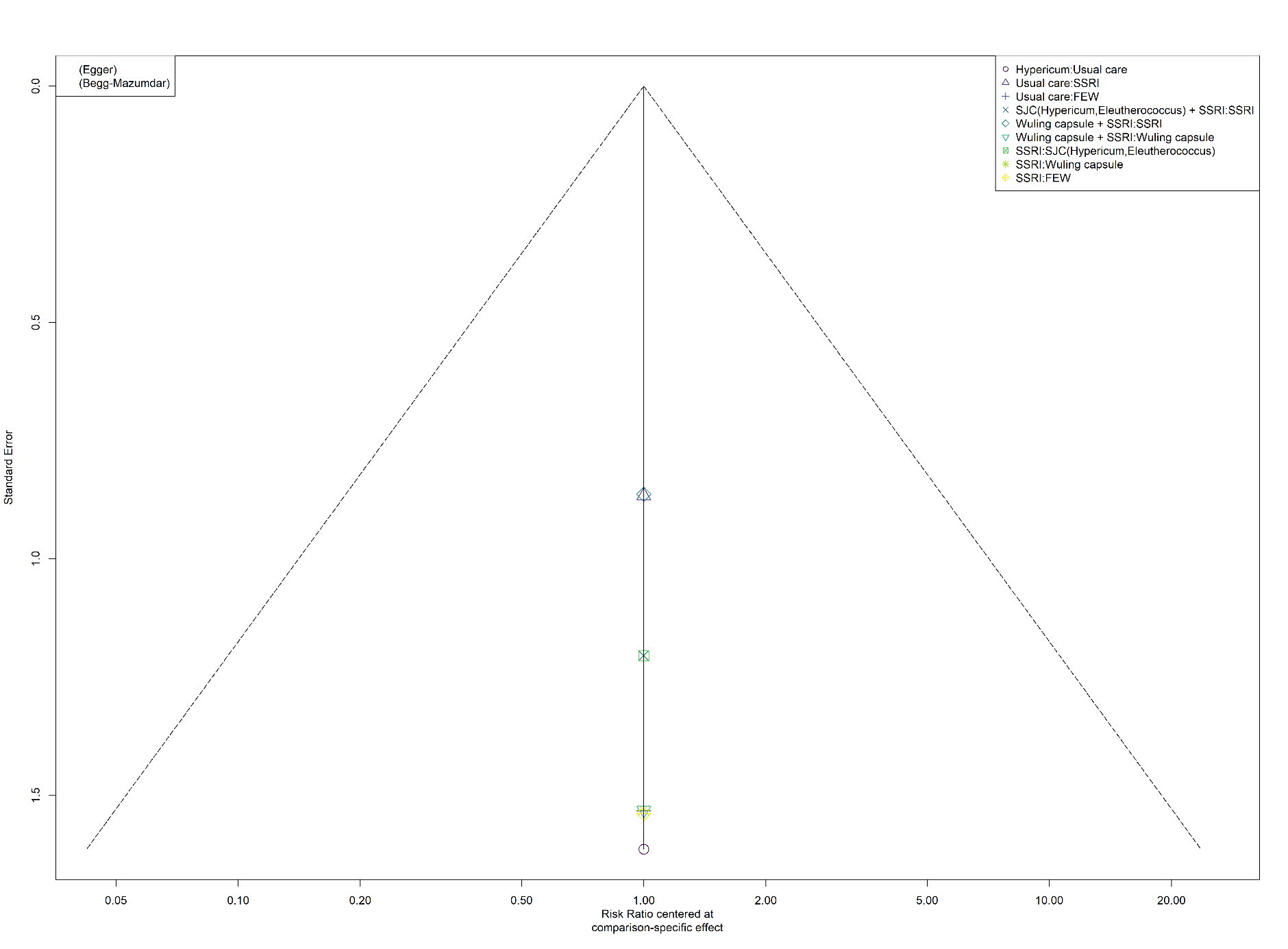


**Outcome: Any gastrointestinal event**


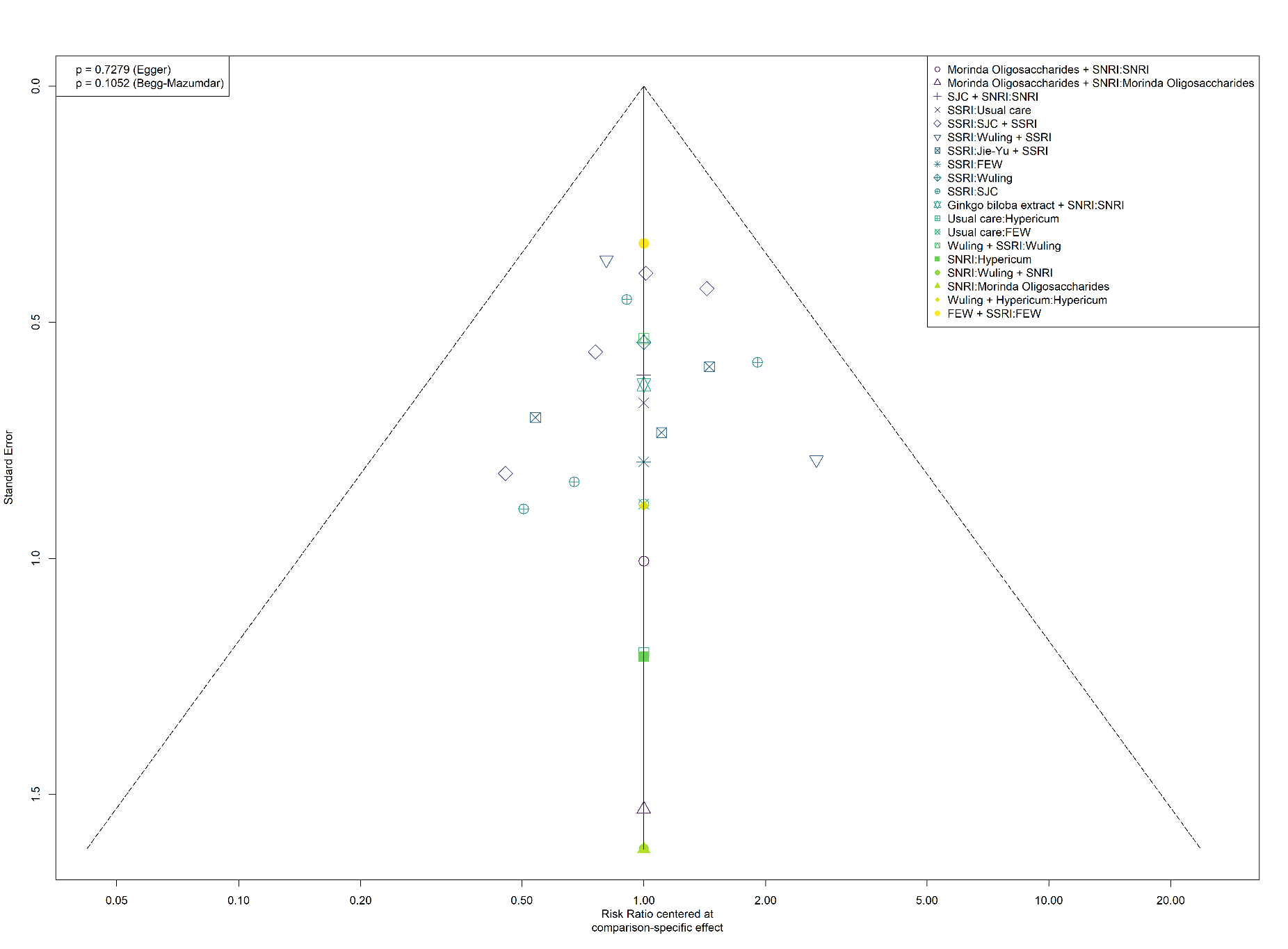


**Outcome: Any nervous system event**


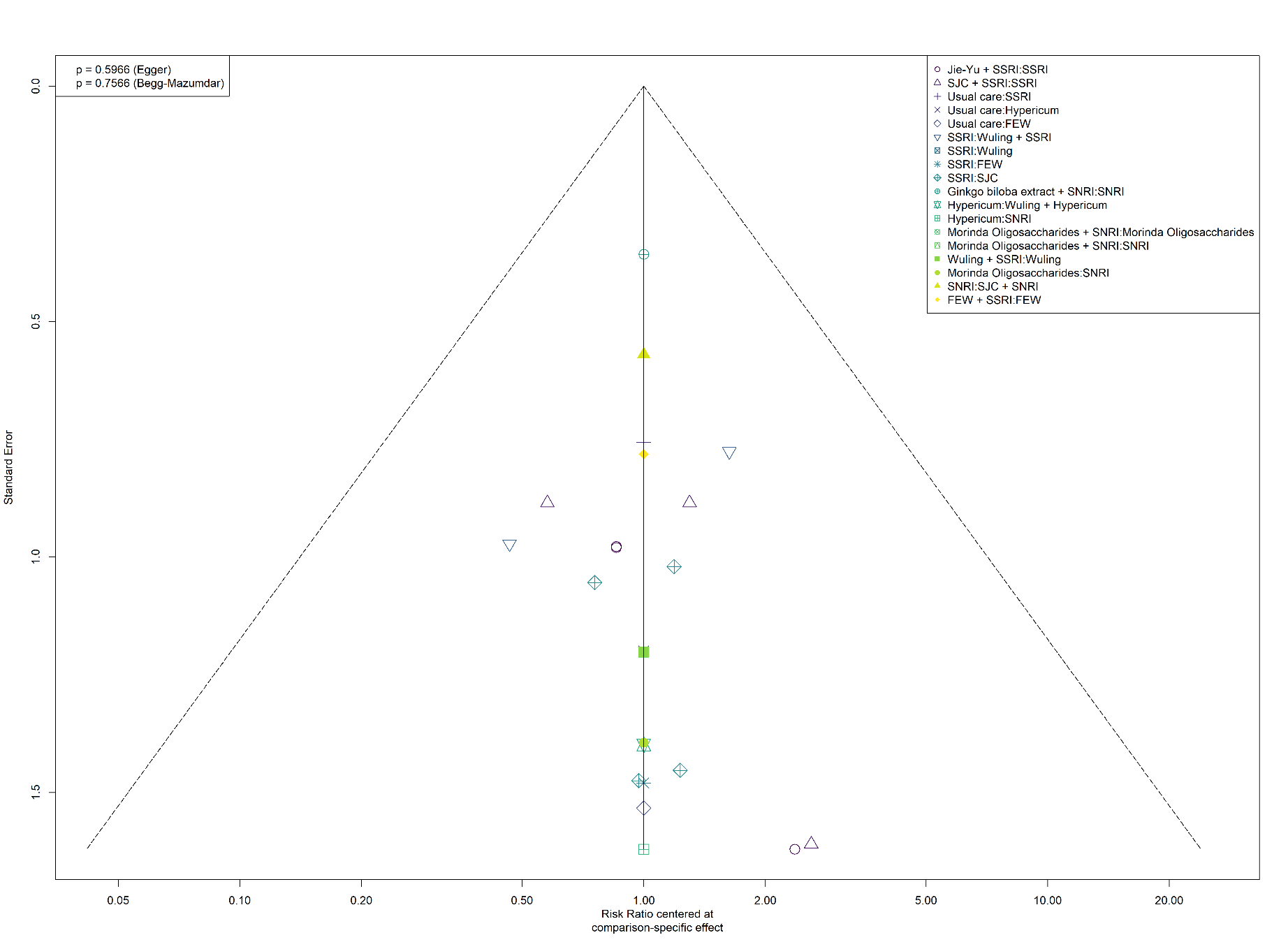


# Appendix 8: GRADE assessments

## 8.1 Certainty of evidence for direct, indirect and network estimates

Notes: a, Risk of bias ; b, Severe risk of bias ; c, Contributing direct evidence of high quality ; d, Contributing direct evidence of high or moderate quality ; e, Contributing direct evidence of high or low quality ; f, Contributing direct evidence of high or very low quality ; g, Contributing direct evidence of moderate quality ; h, Contributing direct evidence of moderate or low quality ; i, Contributing direct evidence of moderate or very low quality ; j, Contributing direct evidence of low quality ; k, Contributing direct evidence of low quality or very low quality ; l, Contributing direct evidence of very low quality ; m, Inconsistency ; n, Imprecision ; o, Severe imprecision .

**Outcome: Response rate**

| Comparison | Direct Estimate | Certainty | Indirect Estimate | Certainty | Network Estimate | Certainty |
| --- | --- | --- | --- | --- | --- | --- |
| FEW vs FEW + SSRI | 0.93 (0.65, 1.33) | Moderate ^a^ | 0.73 (0.43, 1.27) | Moderate ^d^ | 0.86 (0.64, 1.17) | Low ^n^ |
| FEW vs Flupentixol/Melitracen | — | — | 1.06 (0.7, 1.59) | Low ^h^ | 1.06 (0.7, 1.59) | Very low ^n^ |
| FEW vs Hypericum | — | — | 1.04 (0.6, 1.82) | Moderate ^d^ | 1.04 (0.6, 1.82) | Low ^n^ |
| FEW vs Jie-Yu + SSRI | — | — | 0.75 (0.52, 1.09) | Moderate ^e^ | 0.75 (0.52, 1.09) | Low ^n^ |
| FEW vs Morinda Oligosaccharides | — | — | 1.03 (0.45, 2.36) | Moderate ^g^ | 1.03 (0.45, 2.36) | Low ^n^ |
| FEW vs Morinda Oligosaccharides + SNRI | — | — | 0.84 (0.37, 1.87) | Moderate ^g^ | 0.84 (0.37, 1.87) | Low ^n^ |
| FEW vs SJC | — | — | 0.91 (0.65, 1.27) | Low ^h^ | 0.91 (0.65, 1.27) | Very low ^n^ |
| FEW vs SJC + Flupentixol/Melitracen | — | — | 0.69 (0.38, 1.27) | Moderate ^g^ | 0.69 (0.38, 1.27) | Low ^n^ |
| FEW vs SJC + SNRI | — | — | 0.69 (0.32, 1.47) | Moderate ^g^ | 0.69 (0.32, 1.47) | Low ^n^ |
| FEW vs SJC + SSRI | — | — | 0.71 (0.5, 1) | Moderate ^e^ | 0.71 (0.5, 1) | Low |
| FEW vs SNRI | — | — | 1.03 (0.52, 2.05) | Moderate ^g^ | 1.03 (0.52, 2.05) | Low ^n^ |
| FEW vs SSRI | 0.92 (0.64, 1.33) | High | 1.27 (0.74, 2.18) | Moderate ^g^ | 1.02 (0.75, 1.38) | Moderate ^n^ |
| FEW vs Usual care | 36.8 (2.31, 585.65) | High | 1.21 (0.53, 2.77) | Moderate ^d^ | 1.6 (0.73, 3.55) | Low ^m, n^ |
| FEW vs Wuling | — | — | 0.98 (0.68, 1.4) | Low ^h^ | 0.98 (0.68, 1.4) | Very low ^n^ |
| FEW vs Wuling + Flupentixol/Melitracen | — | — | 0.84 (0.56, 1.27) | Moderate ^d^ | 0.84 (0.56, 1.27) | Low ^n^ |
| FEW vs Wuling + Hypericum | — | — | 0.62 (0.3, 1.29) | Moderate ^g^ | 0.62 (0.3, 1.29) | Low ^n^ |
| FEW vs Wuling + SNRI | — | — | 0.67 (0.25, 1.81) | Moderate ^g^ | 0.67 (0.25, 1.81) | Low ^n^ |
| FEW vs Wuling + SSRI | — | — | 0.77 (0.54, 1.11) | Low ^h^ | 0.77 (0.54, 1.11) | Very low ^n^ |
| FEW + SSRI vs Flupentixol/Melitracen | — | — | 1.22 (0.8, 1.86) | Moderate ^g^ | 1.22 (0.8, 1.86) | Low ^n^ |
| FEW + SSRI vs Hypericum | — | — | 1.21 (0.69, 2.12) | Moderate ^g^ | 1.21 (0.69, 2.12) | Low ^n^ |
| FEW + SSRI vs Jie-Yu + SSRI | — | — | 0.87 (0.59, 1.28) | Low ^h^ | 0.87 (0.59, 1.28) | Very low ^n^ |
| FEW + SSRI vs Morinda Oligosaccharides | — | — | 1.2 (0.52, 2.75) | Moderate ^g^ | 1.2 (0.52, 2.75) | Low ^n^ |
| FEW + SSRI vs Morinda Oligosaccharides + SNRI | — | — | 0.97 (0.43, 2.17) | Moderate ^g^ | 0.97 (0.43, 2.17) | Low ^n^ |
| FEW + SSRI vs SJC | — | — | 1.05 (0.74, 1.49) | Moderate ^g^ | 1.05 (0.74, 1.49) | Low ^n^ |
| FEW + SSRI vs SJC + Flupentixol/Melitracen | — | — | 0.8 (0.43, 1.48) | Moderate ^g^ | 0.8 (0.43, 1.48) | Low ^n^ |
| FEW + SSRI vs SJC + SNRI | — | — | 0.8 (0.37, 1.71) | Moderate ^g^ | 0.8 (0.37, 1.71) | Low ^n^ |
| FEW + SSRI vs SJC + SSRI | — | — | 0.82 (0.57, 1.17) | Low ^h^ | 0.82 (0.57, 1.17) | Very low ^n^ |
| FEW + SSRI vs SNRI | — | — | 1.2 (0.6, 2.39) | Moderate ^g^ | 1.2 (0.6, 2.39) | Low ^n^ |
| FEW + SSRI vs SSRI | 1.29 (0.86, 1.94) | Moderate ^a^ | 1.02 (0.61, 1.71) | Moderate ^d^ | 1.18 (0.86, 1.62) | Low ^n^ |
| FEW + SSRI vs Usual care | — | — | 1.86 (0.83, 4.13) | Moderate ^d^ | 1.86 (0.83, 4.13) | Low ^n^ |
| FEW + SSRI vs Wuling | — | — | 1.13 (0.78, 1.65) | Low ^h^ | 1.13 (0.78, 1.65) | Very low ^n^ |
| FEW + SSRI vs Wuling + Flupentixol/Melitracen | — | — | 0.97 (0.64, 1.49) | Moderate ^g^ | 0.97 (0.64, 1.49) | Low ^n^ |
| FEW + SSRI vs Wuling + Hypericum | — | — | 0.72 (0.34, 1.5) | Moderate ^g^ | 0.72 (0.34, 1.5) | Low ^n^ |
| FEW + SSRI vs Wuling + SNRI | — | — | 0.77 (0.28, 2.1) | Moderate ^g^ | 0.77 (0.28, 2.1) | Low ^n^ |
| FEW + SSRI vs Wuling + SSRI | — | — | 0.9 (0.62, 1.3) | Low ^h^ | 0.9 (0.62, 1.3) | Very low ^n^ |
| Flupentixol/Melitracen vs Hypericum | — | — | 0.98 (0.57, 1.69) | Moderate ^g^ | 0.98 (0.57, 1.69) | Low ^n^ |
| Flupentixol/Melitracen vs Jie-Yu + SSRI | — | — | 0.71 (0.5, 1.01) | Low ^h^ | 0.71 (0.5, 1.01) | Very low ^n^ |
| Flupentixol/Melitracen vs Morinda Oligosaccharides | — | — | 0.98 (0.43, 2.21) | Moderate ^g^ | 0.98 (0.43, 2.21) | Low ^n^ |
| Flupentixol/Melitracen vs Morinda Oligosaccharides + SNRI | — | — | 0.79 (0.36, 1.75) | Moderate ^g^ | 0.79 (0.36, 1.75) | Low ^n^ |
| Flupentixol/Melitracen vs SJC | 0.59 (0.37, 0.95) | High | 1.06 (0.74, 1.53) | Moderate ^g^ | 0.86 (0.64, 1.14) | Moderate ^n^ |
| Flupentixol/Melitracen vs SJC + Flupentixol/Melitracen | 0.66 (0.42, 1.03) | Moderate ^a^ | — | — | 0.66 (0.42, 1.03) | Low ^n^ |
| Flupentixol/Melitracen vs SJC + SNRI | — | — | 0.65 (0.31, 1.37) | Moderate ^g^ | 0.65 (0.31, 1.37) | Low ^n^ |
| Flupentixol/Melitracen vs SJC + SSRI | — | — | 0.67 (0.49, 0.91) | Low ^h^ | 0.67 (0.49, 0.91) | Very low ^n^ |
| Flupentixol/Melitracen vs SNRI | — | — | 0.98 (0.5, 1.92) | Moderate ^g^ | 0.98 (0.5, 1.92) | Low ^n^ |
| Flupentixol/Melitracen vs SSRI | — | — | 0.96 (0.73, 1.27) | Low ^h^ | 0.96 (0.73, 1.27) | Very low ^n^ |
| Flupentixol/Melitracen vs Usual care | — | — | 1.52 (0.7, 3.3) | Moderate ^d^ | 1.52 (0.7, 3.3) | Low ^n^ |
| Flupentixol/Melitracen vs Wuling | 1.01 (0.77, 1.31) | Moderate ^a^ | 0.74 (0.48, 1.14) | Moderate ^e^ | 0.92 (0.73, 1.16) | Low ^n^ |
| Flupentixol/Melitracen vs Wuling + Flupentixol/Melitracen | 0.83 (0.68, 1) | Moderate ^a^ | 0.31 (0.12, 0.79) | Moderate ^e^ | 0.79 (0.66, 0.96) | Low ^n^ |
| Flupentixol/Melitracen vs Wuling + Hypericum | — | — | 0.58 (0.28, 1.2) | Moderate ^g^ | 0.58 (0.28, 1.2) | Low ^n^ |
| Flupentixol/Melitracen vs Wuling + SNRI | — | — | 0.63 (0.23, 1.69) | Moderate ^g^ | 0.63 (0.23, 1.69) | Low ^n^ |
| Flupentixol/Melitracen vs Wuling + SSRI | — | — | 0.73 (0.54, 0.99) | Low ^h^ | 0.73 (0.54, 0.99) | Very low ^n^ |
| Hypericum vs Jie-Yu + SSRI | — | — | 0.72 (0.43, 1.21) | Low ^h^ | 0.72 (0.43, 1.21) | Very low ^n^ |
| Hypericum vs Morinda Oligosaccharides | — | — | 0.99 (0.54, 1.83) | Moderate ^g^ | 0.99 (0.54, 1.83) | Low ^n^ |
| Hypericum vs Morinda Oligosaccharides + SNRI | — | — | 0.8 (0.45, 1.43) | Moderate ^g^ | 0.8 (0.45, 1.43) | Low ^n^ |
| Hypericum vs SJC | — | — | 0.87 (0.53, 1.42) | Moderate ^g^ | 0.87 (0.53, 1.42) | Low ^n^ |
| Hypericum vs SJC + Flupentixol/Melitracen | — | — | 0.67 (0.33, 1.34) | Moderate ^g^ | 0.67 (0.33, 1.34) | Low ^n^ |
| Hypericum vs SJC + SNRI | — | — | 0.66 (0.39, 1.11) | Low ^h^ | 0.66 (0.39, 1.11) | Very low ^n^ |
| Hypericum vs SJC + SSRI | — | — | 0.68 (0.41, 1.11) | Low ^h^ | 0.68 (0.41, 1.11) | Very low ^n^ |
| Hypericum vs SNRI | 0.99 (0.66, 1.49) | Moderate ^a^ | — | — | 0.99 (0.66, 1.49) | Low ^n^ |
| Hypericum vs SSRI | 0.98 (0.62, 1.56) | Moderate ^a^ | — | — | 0.98 (0.62, 1.56) | Low ^n^ |
| Hypericum vs Usual care | — | — | 1.54 (0.64, 3.68) | Moderate ^d^ | 1.54 (0.64, 3.68) | Low ^n^ |
| Hypericum vs Wuling | — | — | 0.94 (0.57, 1.56) | Low ^h^ | 0.94 (0.57, 1.56) | Very low ^n^ |
| Hypericum vs Wuling + Flupentixol/Melitracen | — | — | 0.81 (0.47, 1.39) | Moderate ^g^ | 0.81 (0.47, 1.39) | Low ^n^ |
| Hypericum vs Wuling + Hypericum | 0.59 (0.37, 0.96) | Moderate ^a^ | — | — | 0.59 (0.37, 0.96) | Low ^n^ |
| Hypericum vs Wuling + SNRI | — | — | 0.64 (0.28, 1.46) | Moderate ^g^ | 0.64 (0.28, 1.46) | Low ^n^ |
| Hypericum vs Wuling + SSRI | — | — | 0.74 (0.45, 1.23) | Low ^h^ | 0.74 (0.45, 1.23) | Very low ^n^ |
| Jie-Yu + SSRI vs Morinda Oligosaccharides | — | — | 1.37 (0.62, 3.05) | Low ^h^ | 1.37 (0.62, 3.05) | Very low ^n^ |
| Jie-Yu + SSRI vs Morinda Oligosaccharides + SNRI | — | — | 1.11 (0.51, 2.41) | Low ^h^ | 1.11 (0.51, 2.41) | Very low ^n^ |
| Jie-Yu + SSRI vs SJC | — | — | 1.2 (0.92, 1.56) | Low ^h^ | 1.2 (0.92, 1.56) | Very low ^n^ |
| Jie-Yu + SSRI vs SJC + Flupentixol/Melitracen | — | — | 0.92 (0.52, 1.62) | Low ^h^ | 0.92 (0.52, 1.62) | Very low ^n^ |
| Jie-Yu + SSRI vs SJC + SNRI | — | — | 0.91 (0.44, 1.89) | Low ^h^ | 0.91 (0.44, 1.89) | Very low ^n^ |
| Jie-Yu + SSRI vs SJC + SSRI | — | — | 0.94 (0.71, 1.23) | Low ^j^ | 0.94 (0.71, 1.23) | Very low ^n^ |
| Jie-Yu + SSRI vs SNRI | — | — | 1.37 (0.71, 2.63) | Low ^h^ | 1.37 (0.71, 2.63) | Very low ^n^ |
| Jie-Yu + SSRI vs SSRI | 1.35 (1.09, 1.68) | Moderate ^b^ | — | — | 1.35 (1.09, 1.68) | Moderate |
| Jie-Yu + SSRI vs Usual care | — | — | 2.13 (0.99, 4.59) | Moderate ^e^ | 2.13 (0.99, 4.59) | Low ^n^ |
| Jie-Yu + SSRI vs Wuling | — | — | 1.3 (0.97, 1.74) | Low ^h^ | 1.3 (0.97, 1.74) | Very low ^n^ |
| Jie-Yu + SSRI vs Wuling + Flupentixol/Melitracen | — | — | 1.12 (0.78, 1.59) | Low ^h^ | 1.12 (0.78, 1.59) | Very low ^n^ |
| Jie-Yu + SSRI vs Wuling + Hypericum | — | — | 0.82 (0.41, 1.65) | Low ^h^ | 0.82 (0.41, 1.65) | Very low ^n^ |
| Jie-Yu + SSRI vs Wuling + SNRI | — | — | 0.88 (0.33, 2.34) | Low ^h^ | 0.88 (0.33, 2.34) | Very low ^n^ |
| Jie-Yu + SSRI vs Wuling + SSRI | — | — | 1.03 (0.77, 1.37) | Low ^h^ | 1.03 (0.77, 1.37) | Very low ^n^ |
| Morinda Oligosaccharides vs Morinda Oligosaccharides + SNRI | 0.81 (0.53, 1.23) | Moderate ^a^ | — | — | 0.81 (0.53, 1.23) | Low ^n^ |
| Morinda Oligosaccharides vs SJC | — | — | 0.88 (0.4, 1.91) | Moderate ^g^ | 0.88 (0.4, 1.91) | Low ^n^ |
| Morinda Oligosaccharides vs SJC + Flupentixol/Melitracen | — | — | 0.67 (0.26, 1.7) | Moderate ^g^ | 0.67 (0.26, 1.7) | Low ^n^ |
| Morinda Oligosaccharides vs SJC + SNRI | — | — | 0.67 (0.38, 1.17) | Low ^h^ | 0.67 (0.38, 1.17) | Very low ^n^ |
| Morinda Oligosaccharides vs SJC + SSRI | — | — | 0.68 (0.31, 1.49) | Moderate ^g^ | 0.68 (0.31, 1.49) | Low ^n^ |
| Morinda Oligosaccharides vs SNRI | 1 (0.63, 1.58) | Moderate ^a^ | — | — | 1 (0.63, 1.58) | Low ^n^ |
| Morinda Oligosaccharides vs SSRI | — | — | 0.99 (0.46, 2.13) | Moderate ^g^ | 0.99 (0.46, 2.13) | Low ^n^ |
| Morinda Oligosaccharides vs Usual care | — | — | 1.55 (0.53, 4.5) | Moderate ^g^ | 1.55 (0.53, 4.5) | Low ^n^ |
| Morinda Oligosaccharides vs Wuling | — | — | 0.94 (0.43, 2.09) | Moderate ^g^ | 0.94 (0.43, 2.09) | Low ^n^ |
| Morinda Oligosaccharides vs Wuling + Flupentixol/Melitracen | — | — | 0.81 (0.36, 1.84) | Moderate ^g^ | 0.81 (0.36, 1.84) | Low ^n^ |
| Morinda Oligosaccharides vs Wuling + Hypericum | — | — | 0.6 (0.27, 1.3) | Moderate ^g^ | 0.6 (0.27, 1.3) | Low ^n^ |
| Morinda Oligosaccharides vs Wuling + SNRI | — | — | 0.64 (0.27, 1.52) | Moderate ^g^ | 0.64 (0.27, 1.52) | Low ^n^ |
| Morinda Oligosaccharides vs Wuling + SSRI | — | — | 0.75 (0.34, 1.65) | Moderate ^g^ | 0.75 (0.34, 1.65) | Low ^n^ |
| Morinda Oligosaccharides + SNRI vs SJC | — | — | 1.08 (0.51, 2.31) | Moderate ^g^ | 1.08 (0.51, 2.31) | Low ^n^ |
| Morinda Oligosaccharides + SNRI vs SJC + Flupentixol/Melitracen | — | — | 0.83 (0.33, 2.07) | Moderate ^g^ | 0.83 (0.33, 2.07) | Low ^n^ |
| Morinda Oligosaccharides + SNRI vs SJC + SNRI | — | — | 0.82 (0.48, 1.4) | Low ^h^ | 0.82 (0.48, 1.4) | Very low ^n^ |
| Morinda Oligosaccharides + SNRI vs SJC + SSRI | — | — | 0.84 (0.39, 1.81) | Moderate ^g^ | 0.84 (0.39, 1.81) | Low ^n^ |
| Morinda Oligosaccharides + SNRI vs SNRI | 1.24 (0.81, 1.88) | Moderate ^a^ | — | — | 1.24 (0.81, 1.88) | Low ^n^ |
| Morinda Oligosaccharides + SNRI vs SSRI | — | — | 1.22 (0.58, 2.57) | Moderate ^g^ | 1.22 (0.58, 2.57) | Low ^n^ |
| Morinda Oligosaccharides + SNRI vs Usual care | — | — | 1.92 (0.67, 5.47) | Moderate ^g^ | 1.92 (0.67, 5.47) | Low ^n^ |
| Morinda Oligosaccharides + SNRI vs Wuling | — | — | 1.17 (0.54, 2.53) | Moderate ^g^ | 1.17 (0.54, 2.53) | Low ^n^ |
| Morinda Oligosaccharides + SNRI vs Wuling + Flupentixol/Melitracen | — | — | 1.01 (0.45, 2.23) | Moderate ^g^ | 1.01 (0.45, 2.23) | Low ^n^ |
| Morinda Oligosaccharides + SNRI vs Wuling + Hypericum | — | — | 0.74 (0.35, 1.57) | Moderate ^g^ | 0.74 (0.35, 1.57) | Low ^n^ |
| Morinda Oligosaccharides + SNRI vs Wuling + SNRI | — | — | 0.8 (0.34, 1.84) | Moderate ^g^ | 0.8 (0.34, 1.84) | Low ^n^ |
| Morinda Oligosaccharides + SNRI vs Wuling + SSRI | — | — | 0.93 (0.43, 2) | Moderate ^g^ | 0.93 (0.43, 2) | Low ^n^ |
| SJC vs SJC + Flupentixol/Melitracen | — | — | 0.77 (0.45, 1.31) | Moderate ^g^ | 0.77 (0.45, 1.31) | Low ^n^ |
| SJC vs SJC + SNRI | — | — | 0.76 (0.37, 1.55) | Low ^h^ | 0.76 (0.37, 1.55) | Very low ^n^ |
| SJC vs SJC + SSRI | 0.71 (0.49, 1.04) | Moderate ^b^ | 0.81 (0.63, 1.03) | Low ^h^ | 0.78 (0.64, 0.96) | Low ^n^ |
| SJC vs SNRI | — | — | 1.14 (0.61, 2.15) | Moderate ^g^ | 1.14 (0.61, 2.15) | Low ^n^ |
| SJC vs SSRI | 1.09 (0.94, 1.28) | Moderate ^a^ | 1.43 (0.92, 2.23) | Low ^h^ | 1.13 (0.97, 1.31) | Low ^n^ |
| SJC vs Usual care | 1.4 (0.66, 2.97) | High | 43.6 (2.74, 694.36) | Moderate ^d^ | 1.77 (0.86, 3.66) | Low ^m, n^ |
| SJC vs Wuling | — | — | 1.08 (0.85, 1.37) | Low ^h^ | 1.08 (0.85, 1.37) | Very low ^n^ |
| SJC vs Wuling + Flupentixol/Melitracen | — | — | 0.93 (0.69, 1.25) | Moderate ^g^ | 0.93 (0.69, 1.25) | Low ^n^ |
| SJC vs Wuling + Hypericum | — | — | 0.68 (0.34, 1.35) | Moderate ^g^ | 0.68 (0.34, 1.35) | Low ^n^ |
| SJC vs Wuling + SNRI | — | — | 0.73 (0.28, 1.92) | Moderate ^g^ | 0.73 (0.28, 1.92) | Low ^n^ |
| SJC vs Wuling + SSRI | — | — | 0.86 (0.68, 1.08) | Low ^h^ | 0.86 (0.68, 1.08) | Very low ^n^ |
| SJC + Flupentixol/Melitracen vs SJC + SNRI | — | — | 0.99 (0.41, 2.37) | Low ^h^ | 0.99 (0.41, 2.37) | Very low ^n^ |
| SJC + Flupentixol/Melitracen vs SJC + SSRI | — | — | 1.02 (0.59, 1.76) | Low ^h^ | 1.02 (0.59, 1.76) | Very low ^n^ |
| SJC + Flupentixol/Melitracen vs SNRI | — | — | 1.49 (0.66, 3.35) | Moderate ^g^ | 1.49 (0.66, 3.35) | Low ^n^ |
| SJC + Flupentixol/Melitracen vs SSRI | — | — | 1.47 (0.87, 2.49) | Moderate ^g^ | 1.47 (0.87, 2.49) | Low ^n^ |
| SJC + Flupentixol/Melitracen vs Usual care | — | — | 2.31 (0.94, 5.68) | Moderate ^d^ | 2.31 (0.94, 5.68) | Low ^n^ |
| SJC + Flupentixol/Melitracen vs Wuling | — | — | 1.41 (0.85, 2.33) | Moderate ^g^ | 1.41 (0.85, 2.33) | Low ^n^ |
| SJC + Flupentixol/Melitracen vs Wuling + Flupentixol/Melitracen | — | — | 1.21 (0.75, 1.97) | Moderate ^g^ | 1.21 (0.75, 1.97) | Low ^n^ |
| SJC + Flupentixol/Melitracen vs Wuling + Hypericum | — | — | 0.89 (0.38, 2.09) | Moderate ^g^ | 0.89 (0.38, 2.09) | Low ^n^ |
| SJC + Flupentixol/Melitracen vs Wuling + SNRI | — | — | 0.96 (0.32, 2.84) | Moderate ^g^ | 0.96 (0.32, 2.84) | Low ^n^ |
| SJC + Flupentixol/Melitracen vs Wuling + SSRI | — | — | 1.12 (0.65, 1.91) | Low ^h^ | 1.12 (0.65, 1.91) | Very low ^n^ |
| SJC + SNRI vs SJC + SSRI | — | — | 1.02 (0.5, 2.09) | Low ^h^ | 1.02 (0.5, 2.09) | Very low ^n^ |
| SJC + SNRI vs SNRI | 1.5 (1.09, 2.08) | Moderate ^b^ | — | — | 1.5 (1.09, 2.08) | Moderate |
| SJC + SNRI vs SSRI | — | — | 1.48 (0.74, 2.97) | Low ^h^ | 1.48 (0.74, 2.97) | Very low ^n^ |
| SJC + SNRI vs Usual care | — | — | 2.33 (0.85, 6.42) | Low ^h^ | 2.33 (0.85, 6.42) | Very low ^n^ |
| SJC + SNRI vs Wuling | — | — | 1.42 (0.69, 2.93) | Low ^h^ | 1.42 (0.69, 2.93) | Very low ^n^ |
| SJC + SNRI vs Wuling + Flupentixol/Melitracen | — | — | 1.22 (0.58, 2.59) | Low ^h^ | 1.22 (0.58, 2.59) | Very low ^n^ |
| SJC + SNRI vs Wuling + Hypericum | — | — | 0.9 (0.44, 1.82) | Low ^h^ | 0.9 (0.44, 1.82) | Very low ^n^ |
| SJC + SNRI vs Wuling + SNRI | — | — | 0.97 (0.44, 2.14) | Low ^h^ | 0.97 (0.44, 2.14) | Very low ^n^ |
| SJC + SNRI vs Wuling + SSRI | — | — | 1.12 (0.55, 2.31) | Low ^h^ | 1.12 (0.55, 2.31) | Very low ^n^ |
| SJC + SSRI vs SNRI | — | — | 1.47 (0.78, 2.77) | Moderate ^g^ | 1.47 (0.78, 2.77) | Low ^n^ |
| SJC + SSRI vs SSRI | 1.42 (1.2, 1.68) | Moderate ^b^ | 1.77 (1, 3.13) | Low ^h^ | 1.45 (1.23, 1.7) | Moderate |
| SJC + SSRI vs Usual care | — | — | 2.27 (1.07, 4.82) | Moderate ^e^ | 2.27 (1.07, 4.82) | Moderate |
| SJC + SSRI vs Wuling | — | — | 1.39 (1.07, 1.79) | Low ^h^ | 1.39 (1.07, 1.79) | Low |
| SJC + SSRI vs Wuling + Flupentixol/Melitracen | — | — | 1.19 (0.86, 1.65) | Low ^h^ | 1.19 (0.86, 1.65) | Very low ^n^ |
| SJC + SSRI vs Wuling + Hypericum | — | — | 0.88 (0.44, 1.74) | Moderate ^g^ | 0.88 (0.44, 1.74) | Low ^n^ |
| SJC + SSRI vs Wuling + SNRI | — | — | 0.94 (0.36, 2.47) | Moderate ^g^ | 0.94 (0.36, 2.47) | Low ^n^ |
| SJC + SSRI vs Wuling + SSRI | — | — | 1.1 (0.86, 1.41) | Low ^h^ | 1.1 (0.86, 1.41) | Very low ^n^ |
| SNRI vs SSRI | — | — | 0.99 (0.53, 1.83) | Moderate ^g^ | 0.99 (0.53, 1.83) | Low ^n^ |
| SNRI vs Usual care | — | — | 1.55 (0.59, 4.05) | Moderate ^g^ | 1.55 (0.59, 4.05) | Low ^n^ |
| SNRI vs Wuling | — | — | 0.94 (0.49, 1.8) | Moderate ^g^ | 0.94 (0.49, 1.8) | Low ^n^ |
| SNRI vs Wuling + Flupentixol/Melitracen | — | — | 0.81 (0.41, 1.6) | Moderate ^g^ | 0.81 (0.41, 1.6) | Low ^n^ |
| SNRI vs Wuling + Hypericum | — | — | 0.6 (0.32, 1.12) | Moderate ^g^ | 0.6 (0.32, 1.12) | Low ^n^ |
| SNRI vs Wuling + SNRI | 0.64 (0.31, 1.33) | Moderate ^a^ | — | — | 0.64 (0.31, 1.33) | Low ^n^ |
| SNRI vs Wuling + SSRI | — | — | 0.75 (0.39, 1.42) | Moderate ^g^ | 0.75 (0.39, 1.42) | Low ^n^ |
| Usual care vs SSRI | 0.03 (0, 0.4) | High | 0.81 (0.38, 1.75) | Moderate ^d^ | 0.64 (0.3, 1.33) | Low ^m, n^ |
| Wuling vs SSRI | 1.06 (0.85, 1.32) | Moderate ^a^ | 0.99 (0.62, 1.58) | Low ^h^ | 1.04 (0.86, 1.27) | Low ^n^ |
| Wuling + Flupentixol/Melitracen vs SSRI | — | — | 1.21 (0.92, 1.61) | Low ^h^ | 1.21 (0.92, 1.61) | Very low ^n^ |
| Wuling + Hypericum vs SSRI | — | — | 1.65 (0.85, 3.22) | Moderate ^g^ | 1.65 (0.85, 3.22) | Low ^n^ |
| Wuling + SNRI vs SSRI | — | — | 1.53 (0.59, 3.97) | Moderate ^g^ | 1.53 (0.59, 3.97) | Low ^n^ |
| Wuling + SSRI vs SSRI | 1.3 (1.07, 1.58) | Moderate ^b^ | 1.56 (0.76, 3.22) | Low ^h^ | 1.32 (1.09, 1.59) | Moderate |
| Usual care vs Wuling | — | — | 0.61 (0.28, 1.3) | Moderate ^d^ | 0.61 (0.28, 1.3) | Low ^n^ |
| Usual care vs Wuling + Flupentixol/Melitracen | — | — | 0.52 (0.24, 1.15) | Moderate ^d^ | 0.52 (0.24, 1.15) | Low ^n^ |
| Usual care vs Wuling + Hypericum | — | — | 0.39 (0.14, 1.04) | Moderate ^g^ | 0.39 (0.14, 1.04) | Low ^n^ |
| Usual care vs Wuling + SNRI | — | — | 0.41 (0.12, 1.38) | Moderate ^g^ | 0.41 (0.12, 1.38) | Low ^n^ |
| Usual care vs Wuling + SSRI | — | — | 0.48 (0.23, 1.03) | Low ^h^ | 0.48 (0.23, 1.03) | Very low ^n^ |
| Wuling vs Wuling + Flupentixol/Melitracen | 0.8 (0.63, 1.02) | Moderate ^a^ | 1.38 (0.76, 2.51) | Moderate ^e^ | 0.86 (0.69, 1.08) | Low ^n^ |
| Wuling vs Wuling + Hypericum | — | — | 0.63 (0.31, 1.27) | Moderate ^g^ | 0.63 (0.31, 1.27) | Low ^n^ |
| Wuling vs Wuling + SNRI | — | — | 0.68 (0.26, 1.8) | Moderate ^g^ | 0.68 (0.26, 1.8) | Low ^n^ |
| Wuling vs Wuling + SSRI | 0.84 (0.66, 1.07) | Moderate ^b^ | 0.64 (0.4, 1.03) | Low ^h^ | 0.79 (0.64, 0.99) | Moderate |
| Wuling + Flupentixol/Melitracen vs Wuling + Hypericum | — | — | 0.73 (0.36, 1.52) | Moderate ^g^ | 0.73 (0.36, 1.52) | Low ^n^ |
| Wuling + Flupentixol/Melitracen vs Wuling + SNRI | — | — | 0.79 (0.29, 2.13) | Moderate ^g^ | 0.79 (0.29, 2.13) | Low ^n^ |
| Wuling + Flupentixol/Melitracen vs Wuling + SSRI | — | — | 0.92 (0.68, 1.25) | Low ^h^ | 0.92 (0.68, 1.25) | Very low ^n^ |
| Wuling + Hypericum vs Wuling + SNRI | — | — | 1.08 (0.41, 2.81) | Moderate ^g^ | 1.08 (0.41, 2.81) | Low ^n^ |
| Wuling + Hypericum vs Wuling + SSRI | — | — | 1.25 (0.63, 2.51) | Moderate ^g^ | 1.25 (0.63, 2.51) | Low ^n^ |
| Wuling + SNRI vs Wuling + SSRI | — | — | 1.16 (0.44, 3.07) | Moderate ^g^ | 1.16 (0.44, 3.07) | Low ^n^ |

**Outcome:** **Mean changes in HAMD score from baseline**

| Comparison | Direct Estimate | Certainty | Indirect Estimate | Certainty | Network Estimate | Certainty |
| --- | --- | --- | --- | --- | --- | --- |
| FEW vs FEW + SSRI | -4.98 (-11.15, 1.19) | Moderate ^a^ | 3.43 (-3.8, 10.66) | Moderate ^g^ | -1.44 (-6.13, 3.26) | Low ^n^ |
| FEW vs Flupentixol/Melitracen | — | — | -5.41 (-9.94, -0.89) | Moderate ^e^ | -5.41 (-9.94, -0.89) | Moderate |
| FEW vs Ginkgo biloba extract + SNRI | — | — | 3.93 (-5.95, 13.81) | Moderate ^d^ | 3.93 (-5.95, 13.81) | Low ^n^ |
| FEW vs Hypericum | — | — | -1.08 (-6.22, 4.06) | Moderate ^e^ | -1.08 (-6.22, 4.06) | Low ^n^ |
| FEW vs Jie-Yu + SSRI | — | — | 3.3 (-1.33, 7.92) | Very low ^f^ | 3.3 (-1.33, 7.92) | Very low ^n^ |
| FEW vs Morinda Oligosaccharides | — | — | -7.02 (-16.98, 2.94) | Moderate ^g^ | -7.02 (-16.98, 2.94) | Low ^n^ |
| FEW vs Morinda Oligosaccharides + SNRI | — | — | 5.24 (-4.72, 15.2) | Moderate ^g^ | 5.24 (-4.72, 15.2) | Low ^n^ |
| FEW vs SJC | — | — | -1.27 (-5.37, 2.82) | Moderate ^e^ | -1.27 (-5.37, 2.82) | Very low ^n^ |
| FEW vs SJC + Flupentixol/Melitracen | — | — | -0.66 (-6.58, 5.27) | Moderate ^d^ | -0.66 (-6.58, 5.27) | Low ^n^ |
| FEW vs SJC + SNRI | — | — | 5.98 (-2.92, 14.88) | Low ^h^ | 5.98 (-2.92, 14.88) | Very low ^n^ |
| FEW vs SJC + SSRI | — | — | 2.16 (-2.12, 6.44) | Very low ^f^ | 2.16 (-2.12, 6.44) | Very low ^n^ |
| FEW vs SNRI | — | — | 0.1 (-7.73, 7.93) | Low ^h^ | 0.1 (-7.73, 7.93) | Very low ^n^ |
| FEW vs SSRI | 0.4 (-5.46, 6.26) | High | -3.55 (-8.45, 1.35) | Moderate ^d^ | -1.92 (-5.68, 1.84) | Moderate ^n^ |
| FEW vs Usual care | -5 (-10.85, 0.85) | High | -9.72 (-15.64, -3.79) | Moderate ^g^ | -7.33 (-11.49, -3.16) | Moderate ^m^ |
| FEW vs Wuling | -5.24 (-11.26, 0.78) | Moderate ^a^ | -5.42 (-10.62, -0.22) | Very low ^k^ | -5.34 (-9.28, -1.41) | Low ^m^ |
| FEW vs Wuling + Flupentixol/Melitracen | — | — | -1.04 (-5.73, 3.64) | Moderate ^e^ | -1.04 (-5.73, 3.64) | Low ^n^ |
| FEW vs Wuling + Hypericum | — | — | 8.2 (0.37, 16.03) | Low ^h^ | 8.2 (0.37, 16.03) | Low |
| FEW vs Wuling + SNRI | — | — | 2.76 (-7.13, 12.65) | Moderate ^g^ | 2.76 (-7.13, 12.65) | Low ^n^ |
| FEW vs Wuling + SSRI | — | — | 1.88 (-2.44, 6.21) | Moderate ^e^ | 1.88 (-2.44, 6.21) | Low ^n^ |
| FEW + SSRI vs Flupentixol/Melitracen | — | — | -3.98 (-9.46, 1.51) | Low ^h^ | -3.98 (-9.46, 1.51) | Very low ^n^ |
| FEW + SSRI vs Ginkgo biloba extract + SNRI | — | — | 5.37 (-4.97, 15.7) | Moderate ^d^ | 5.37 (-4.97, 15.7) | Low ^n^ |
| FEW + SSRI vs Hypericum | — | — | 0.36 (-5.61, 6.32) | Low ^h^ | 0.36 (-5.61, 6.32) | Very low ^n^ |
| FEW + SSRI vs Jie-Yu + SSRI | — | — | 4.73 (-0.62, 10.09) | Very low ^i^ | 4.73 (-0.62, 10.09) | Very low ^n^ |
| FEW + SSRI vs Morinda Oligosaccharides | — | — | -5.58 (-15.99, 4.82) | Moderate ^g^ | -5.58 (-15.99, 4.82) | Low ^n^ |
| FEW + SSRI vs Morinda Oligosaccharides + SNRI | — | — | 6.68 (-3.73, 17.09) | Moderate ^g^ | 6.68 (-3.73, 17.09) | Low ^n^ |
| FEW + SSRI vs SJC | — | — | 0.16 (-4.84, 5.16) | Low ^h^ | 0.16 (-4.84, 5.16) | Very low ^n^ |
| FEW + SSRI vs SJC + Flupentixol/Melitracen | — | — | 0.78 (-5.85, 7.41) | Moderate ^g^ | 0.78 (-5.85, 7.41) | Low ^n^ |
| FEW + SSRI vs SJC + SNRI | — | — | 7.42 (-1.98, 16.82) | Low ^h^ | 7.42 (-1.98, 16.82) | Very low ^n^ |
| FEW + SSRI vs SJC + SSRI | — | — | 3.6 (-1.47, 8.66) | Very low ^i^ | 3.6 (-1.47, 8.66) | Very low ^n^ |
| FEW + SSRI vs SNRI | — | — | 1.54 (-6.85, 9.93) | Moderate ^g^ | 1.54 (-6.85, 9.93) | Low ^n^ |
| FEW + SSRI vs SSRI | -3.72 (-9.61, 2.17) | Moderate ^a^ | 4.69 (-2.77, 12.15) | Moderate ^d^ | -0.49 (-5.11, 4.14) | Low ^n^ |
| FEW + SSRI vs Usual care | — | — | -5.89 (-11.13, -0.65) | Moderate ^g^ | -5.89 (-11.13, -0.65) | Moderate |
| FEW + SSRI vs Wuling | — | — | -3.91 (-8.98, 1.17) | Moderate ^g^ | -3.91 (-8.98, 1.17) | Low ^n^ |
| FEW + SSRI vs Wuling + Flupentixol/Melitracen | — | — | 0.4 (-5.24, 6.03) | Low ^h^ | 0.4 (-5.24, 6.03) | Very low ^n^ |
| FEW + SSRI vs Wuling + Hypericum | — | — | 9.64 (1.24, 18.03) | Moderate ^g^ | 9.64 (1.24, 18.03) | Moderate |
| FEW + SSRI vs Wuling + SNRI | — | — | 4.2 (-6.15, 14.54) | Moderate ^g^ | 4.2 (-6.15, 14.54) | Low ^n^ |
| FEW + SSRI vs Wuling + SSRI | — | — | 3.32 (-1.83, 8.47) | Low ^h^ | 3.32 (-1.83, 8.47) | Very low ^n^ |
| Flupentixol/Melitracen vs Ginkgo biloba extract + SNRI | — | — | 9.34 (-0.3, 18.99) | Moderate ^d^ | 9.34 (-0.3, 18.99) | Low ^n^ |
| Flupentixol/Melitracen vs Hypericum | — | — | 4.33 (-0.35, 9.02) | Low ^h^ | 4.33 (-0.35, 9.02) | Very low ^n^ |
| Flupentixol/Melitracen vs Jie-Yu + SSRI | — | — | 8.71 (4.46, 12.97) | Very low ^k^ | 8.71 (4.46, 12.97) | Very Low |
| Flupentixol/Melitracen vs Morinda Oligosaccharides | — | — | -1.61 (-11.33, 8.12) | Moderate ^g^ | -1.61 (-11.33, 8.12) | Low ^n^ |
| Flupentixol/Melitracen vs Morinda Oligosaccharides + SNRI | — | — | 10.65 (0.92, 20.39) | Moderate ^g^ | 10.65 (0.92, 20.39) | Moderate |
| Flupentixol/Melitracen vs SJC | 4 (-2.2, 10.2) | High | 4.19 (0.35, 8.04) | Low ^h^ | 4.14 (0.87, 7.41) | Moderate ^m^ |
| Flupentixol/Melitracen vs SJC + Flupentixol/Melitracen | 4.78 (-1.44, 11) | Moderate ^a^ | 4.73 (-2.36, 11.82) | Low ^h^ | 4.76 (0.09, 9.43) | Moderate |
| Flupentixol/Melitracen vs SJC + SNRI | — | — | 11.4 (2.76, 20.04) | Low ^h^ | 11.4 (2.76, 20.04) | Low |
| Flupentixol/Melitracen vs SJC + SSRI | — | — | 7.57 (3.72, 11.42) | Very low ^k^ | 7.57 (3.72, 11.42) | Very Low |
| Flupentixol/Melitracen vs SNRI | — | — | 5.51 (-2.02, 13.05) | Low ^h^ | 5.51 (-2.02, 13.05) | Very low ^n^ |
| Flupentixol/Melitracen vs SSRI | — | — | 3.49 (0.2, 6.79) | Low ^j^ | 3.49 (0.2, 6.79) | Low |
| Flupentixol/Melitracen vs Usual care | -3.32 (-9.17, 2.53) | Moderate ^a^ | -1.13 (-5.49, 3.22) | Low ^h^ | -1.91 (-5.41, 1.58) | Low ^n^ |
| Flupentixol/Melitracen vs Wuling | 1.31 (-2.96, 5.58) | Low ^a, p^ | -1.41 (-6.1, 3.27) | Very low ^k^ | 0.07 (-3.08, 3.23) | Very low ^n^ |
| Flupentixol/Melitracen vs Wuling + Flupentixol/Melitracen | 4.38 (1.92, 6.85) | Very low ^b, p^ | 4.08 (-8.99, 17.15) | Low ^h^ | 4.37 (1.95, 6.79) | Very low ^m^ |
| Flupentixol/Melitracen vs Wuling + Hypericum | — | — | 13.61 (6.08, 21.15) | Low ^h^ | 13.61 (6.08, 21.15) | Low |
| Flupentixol/Melitracen vs Wuling + SNRI | — | — | 8.17 (-1.48, 17.83) | Moderate ^g^ | 8.17 (-1.48, 17.83) | Low ^n^ |
| Flupentixol/Melitracen vs Wuling + SSRI | — | — | 7.3 (3.42, 11.17) | Low ^j^ | 7.3 (3.42, 11.17) | Low |
| Ginkgo biloba extract + SNRI vs Hypericum | — | — | -5.01 (-13.45, 3.43) | Moderate ^d^ | -5.01 (-13.45, 3.43) | Low ^n^ |
| Ginkgo biloba extract + SNRI vs Jie-Yu + SSRI | — | — | -0.63 (-10.35, 9.08) | Very low ^i^ | -0.63 (-10.35, 9.08) | Very low ^n^ |
| Ginkgo biloba extract + SNRI vs Morinda Oligosaccharides | — | — | -10.95 (-19.57, -2.33) | Moderate ^d^ | -10.95 (-19.57, -2.33) | Moderate |
| Ginkgo biloba extract + SNRI vs Morinda Oligosaccharides + SNRI | — | — | 1.31 (-7.32, 9.94) | Moderate ^d^ | 1.31 (-7.32, 9.94) | Low ^n^ |
| Ginkgo biloba extract + SNRI vs SJC | — | — | -5.21 (-14.61, 4.2) | Moderate ^d^ | -5.21 (-14.61, 4.2) | Low ^n^ |
| Ginkgo biloba extract + SNRI vs SJC + Flupentixol/Melitracen | — | — | -4.59 (-14.94, 5.77) | Moderate ^d^ | -4.59 (-14.94, 5.77) | Low ^n^ |
| Ginkgo biloba extract + SNRI vs SJC + SNRI | — | — | 2.05 (-5.32, 9.43) | Moderate ^e^ | 2.05 (-5.32, 9.43) | Low ^n^ |
| Ginkgo biloba extract + SNRI vs SJC + SSRI | — | — | -1.77 (-11.32, 7.78) | Moderate ^d^ | -1.77 (-11.32, 7.78) | Low ^n^ |
| Ginkgo biloba extract + SNRI vs SNRI | -3.83 (-9.86, 2.2) | High | — | — | -3.83 (-9.86, 2.2) | Moderate ^n^ |
| Ginkgo biloba extract + SNRI vs SSRI | — | — | -5.85 (-15.19, 3.48) | Moderate ^d^ | -5.85 (-15.19, 3.48) | Low ^n^ |
| Ginkgo biloba extract + SNRI vs Usual care | — | — | -11.26 (-20.43, -2.09) | Moderate ^d^ | -11.26 (-20.43, -2.09) | Moderate |
| Ginkgo biloba extract + SNRI vs Wuling | — | — | -9.27 (-18.86, 0.31) | Moderate ^d^ | -9.27 (-18.86, 0.31) | Low ^n^ |
| Ginkgo biloba extract + SNRI vs Wuling + Flupentixol/Melitracen | — | — | -4.97 (-14.7, 4.76) | Moderate ^d^ | -4.97 (-14.7, 4.76) | Low ^n^ |
| Ginkgo biloba extract + SNRI vs Wuling + Hypericum | — | — | 4.27 (-6.03, 14.57) | Moderate ^e^ | 4.27 (-6.03, 14.57) | Low ^n^ |
| Ginkgo biloba extract + SNRI vs Wuling + SNRI | — | — | -1.17 (-9.71, 7.37) | Moderate ^d^ | -1.17 (-9.71, 7.37) | Low ^n^ |
| Ginkgo biloba extract + SNRI vs Wuling + SSRI | — | — | -2.05 (-11.66, 7.57) | Moderate ^d^ | -2.05 (-11.66, 7.57) | Low ^n^ |
| Hypericum vs Jie-Yu + SSRI | — | — | 4.38 (-0.44, 9.2) | Very low ^k^ | 4.38 (-0.44, 9.2) | Very low ^n^ |
| Hypericum vs Morinda Oligosaccharides | — | — | -5.94 (-14.47, 2.59) | Moderate ^g^ | -5.94 (-14.47, 2.59) | Low ^n^ |
| Hypericum vs Morinda Oligosaccharides + SNRI | — | — | 6.32 (-2.21, 14.85) | Moderate ^g^ | 6.32 (-2.21, 14.85) | Low ^n^ |
| Hypericum vs SJC | — | — | -0.2 (-4.35, 3.96) | Low ^j^ | -0.2 (-4.35, 3.96) | Very low ^n^ |
| Hypericum vs SJC + Flupentixol/Melitracen | — | — | 0.42 (-5.58, 6.43) | Low ^h^ | 0.42 (-5.58, 6.43) | Very low ^n^ |
| Hypericum vs SJC + SNRI | — | — | 7.06 (-0.2, 14.33) | Low ^h^ | 7.06 (-0.2, 14.33) | Very low ^n^ |
| Hypericum vs SJC + SSRI | — | — | 3.24 (-1.24, 7.72) | Very low ^k^ | 3.24 (-1.24, 7.72) | Very low ^n^ |
| Hypericum vs SNRI | 1.18 (-4.72, 7.08) | Moderate ^a^ | — | — | 1.18 (-4.72, 7.08) | Low ^n^ |
| Hypericum vs SSRI | -1 (-7.09, 5.09) | Moderate ^a^ | -0.72 (-6.02, 4.57) | Low ^h^ | -0.84 (-4.84, 3.15) | Low ^n^ |
| Hypericum vs Usual care | -6.17 (-10.39, -1.95) | Low ^a, p^ | -6.45 (-13.33, 0.43) | Low ^h^ | -6.25 (-9.85, -2.65) | Very low ^m^ |
| Hypericum vs Wuling | — | — | -4.26 (-8.82, 0.29) | Low ^h^ | -4.26 (-8.82, 0.29) | Very low ^n^ |
| Hypericum vs Wuling + Flupentixol/Melitracen | — | — | 0.04 (-4.81, 4.89) | Low ^h^ | 0.04 (-4.81, 4.89) | Very low ^n^ |
| Hypericum vs Wuling + Hypericum | 9.28 (3.38, 15.18) | Moderate ^a^ | — | — | 9.28 (3.38, 15.18) | Moderate |
| Hypericum vs Wuling + SNRI | — | — | 3.84 (-4.61, 12.29) | Moderate ^g^ | 3.84 (-4.61, 12.29) | Low ^n^ |
| Hypericum vs Wuling + SSRI | — | — | 2.96 (-1.64, 7.57) | Low ^j^ | 2.96 (-1.64, 7.57) | Very low ^n^ |
| Jie-Yu + SSRI vs Morinda Oligosaccharides | — | — | -10.32 (-20.11, -0.52) | Very low ^i^ | -10.32 (-20.11, -0.52) | Very Low |
| Jie-Yu + SSRI vs Morinda Oligosaccharides + SNRI | — | — | 1.94 (-7.86, 11.74) | Very low ^i^ | 1.94 (-7.86, 11.74) | Very low ^n^ |
| Jie-Yu + SSRI vs SJC | — | — | -4.57 (-8, -1.15) | Very low ^k^ | -4.57 (-8, -1.15) | Very Low |
| Jie-Yu + SSRI vs SJC + Flupentixol/Melitracen | — | — | -3.95 (-9.55, 1.65) | Very low ^i^ | -3.95 (-9.55, 1.65) | Very low ^n^ |
| Jie-Yu + SSRI vs SJC + SNRI | — | — | 2.69 (-6.03, 11.4) | Very low ^k^ | 2.69 (-6.03, 11.4) | Very low ^n^ |
| Jie-Yu + SSRI vs SJC + SSRI | — | — | -1.14 (-4.55, 2.27) | Very low ^l^ | -1.14 (-4.55, 2.27) | Very low ^n^ |
| Jie-Yu + SSRI vs SNRI | — | — | -3.2 (-10.81, 4.42) | Very low ^i^ | -3.2 (-10.81, 4.42) | Very low ^n^ |
| Jie-Yu + SSRI vs SSRI | -5.22 (-7.91, -2.53) | Very low ^b, p^ | — | — | -5.22 (-7.91, -2.53) | Very Low |
| Jie-Yu + SSRI vs Usual care | — | — | -10.63 (-14.61, -6.64) | Very low ^i^ | -10.63 (-14.61, -6.64) | Very Low |
| Jie-Yu + SSRI vs Wuling | — | — | -8.64 (-12.44, -4.85) | Very low ^k^ | -8.64 (-12.44, -4.85) | Very Low |
| Jie-Yu + SSRI vs Wuling + Flupentixol/Melitracen | — | — | -4.34 (-8.81, 0.13) | Very low ^k^ | -4.34 (-8.81, 0.13) | Very low ^n^ |
| Jie-Yu + SSRI vs Wuling + Hypericum | — | — | 4.9 (-2.72, 12.52) | Very low ^i^ | 4.9 (-2.72, 12.52) | Very low ^n^ |
| Jie-Yu + SSRI vs Wuling + SNRI | — | — | -0.54 (-10.26, 9.19) | Very low ^i^ | -0.54 (-10.26, 9.19) | Very low ^n^ |
| Jie-Yu + SSRI vs Wuling + SSRI | — | — | -1.41 (-5.01, 2.19) | Very low ^k^ | -1.41 (-5.01, 2.19) | Very low ^n^ |
| Morinda Oligosaccharides vs Morinda Oligosaccharides + SNRI | 12.26 (6.11, 18.41) | Moderate ^a^ | — | — | 12.26 (6.11, 18.41) | Moderate |
| Morinda Oligosaccharides vs SJC | — | — | 5.74 (-3.74, 15.23) | Moderate ^g^ | 5.74 (-3.74, 15.23) | Low ^n^ |
| Morinda Oligosaccharides vs SJC + Flupentixol/Melitracen | — | — | 6.36 (-4.06, 16.79) | Moderate ^g^ | 6.36 (-4.06, 16.79) | Low ^n^ |
| Morinda Oligosaccharides vs SJC + SNRI | — | — | 13 (5.53, 20.48) | Low ^h^ | 13 (5.53, 20.48) | Low |
| Morinda Oligosaccharides vs SJC + SSRI | — | — | 9.18 (-0.45, 18.81) | Moderate ^g^ | 9.18 (-0.45, 18.81) | Low ^n^ |
| Morinda Oligosaccharides vs SNRI | 7.12 (0.96, 13.28) | Moderate ^a^ | — | — | 7.12 (0.96, 13.28) | Moderate |
| Morinda Oligosaccharides vs SSRI | — | — | 5.1 (-4.32, 14.51) | Moderate ^g^ | 5.1 (-4.32, 14.51) | Low ^n^ |
| Morinda Oligosaccharides vs Usual care | — | — | -0.31 (-9.56, 8.94) | Moderate ^g^ | -0.31 (-9.56, 8.94) | Low ^n^ |
| Morinda Oligosaccharides vs Wuling | — | — | 1.68 (-7.99, 11.34) | Moderate ^g^ | 1.68 (-7.99, 11.34) | Low ^n^ |
| Morinda Oligosaccharides vs Wuling + Flupentixol/Melitracen | — | — | 5.98 (-3.83, 15.79) | Moderate ^g^ | 5.98 (-3.83, 15.79) | Low ^n^ |
| Morinda Oligosaccharides vs Wuling + Hypericum | — | — | 15.22 (4.85, 25.59) | Moderate ^g^ | 15.22 (4.85, 25.59) | Moderate |
| Morinda Oligosaccharides vs Wuling + SNRI | — | — | 9.78 (1.15, 18.41) | Moderate ^g^ | 9.78 (1.15, 18.41) | Moderate |
| Morinda Oligosaccharides vs Wuling + SSRI | — | — | 8.9 (-0.79, 18.59) | Moderate ^g^ | 8.9 (-0.79, 18.59) | Low ^n^ |
| Morinda Oligosaccharides + SNRI vs SJC | — | — | -6.52 (-16, 2.97) | Moderate ^g^ | -6.52 (-16, 2.97) | Low ^n^ |
| Morinda Oligosaccharides + SNRI vs SJC + Flupentixol/Melitracen | — | — | -5.9 (-16.33, 4.54) | Moderate ^g^ | -5.9 (-16.33, 4.54) | Low ^n^ |
| Morinda Oligosaccharides + SNRI vs SJC + SNRI | — | — | 0.74 (-6.74, 8.22) | Low ^h^ | 0.74 (-6.74, 8.22) | Very low ^n^ |
| Morinda Oligosaccharides + SNRI vs SJC + SSRI | — | — | -3.08 (-12.72, 6.55) | Moderate ^g^ | -3.08 (-12.72, 6.55) | Low ^n^ |
| Morinda Oligosaccharides + SNRI vs SNRI | -5.14 (-11.3, 1.02) | Moderate ^a^ | — | — | -5.14 (-11.3, 1.02) | Low ^n^ |
| Morinda Oligosaccharides + SNRI vs SSRI | — | — | -7.16 (-16.58, 2.26) | Moderate ^g^ | -7.16 (-16.58, 2.26) | Low ^n^ |
| Morinda Oligosaccharides + SNRI vs Usual care | — | — | -12.57 (-21.83, -3.31) | Moderate ^g^ | -12.57 (-21.83, -3.31) | Moderate |
| Morinda Oligosaccharides + SNRI vs Wuling | — | — | -10.58 (-20.25, -0.91) | Moderate ^g^ | -10.58 (-20.25, -0.91) | Moderate |
| Morinda Oligosaccharides + SNRI vs Wuling + Flupentixol/Melitracen | — | — | -6.28 (-16.1, 3.53) | Moderate ^g^ | -6.28 (-16.1, 3.53) | Low ^n^ |
| Morinda Oligosaccharides + SNRI vs Wuling + Hypericum | — | — | 2.96 (-7.42, 13.34) | Moderate ^g^ | 2.96 (-7.42, 13.34) | Low ^n^ |
| Morinda Oligosaccharides + SNRI vs Wuling + SNRI | — | — | -2.48 (-11.12, 6.16) | Moderate ^g^ | -2.48 (-11.12, 6.16) | Low ^n^ |
| Morinda Oligosaccharides + SNRI vs Wuling + SSRI | — | — | -3.36 (-13.05, 6.34) | Moderate ^g^ | -3.36 (-13.05, 6.34) | Low ^n^ |
| SJC vs SJC + Flupentixol/Melitracen | 0.6 (-5.55, 6.75) | Moderate ^a^ | 0.65 (-6.5, 7.79) | Moderate ^g^ | 0.62 (-4.04, 5.28) | Low ^n^ |
| SJC vs SJC + SNRI | — | — | 7.26 (-1.11, 15.63) | Low ^h^ | 7.26 (-1.11, 15.63) | Very low ^n^ |
| SJC vs SJC + SSRI | 2.64 (-3.28, 8.56) | Very low ^b, p^ | 3.67 (0.43, 6.92) | Low ^h^ | 3.43 (0.59, 6.28) | Very low ^m^ |
| SJC vs SNRI | — | — | 1.38 (-5.84, 8.59) | Low ^h^ | 1.38 (-5.84, 8.59) | Very low ^n^ |
| SJC vs SSRI | -0.33 (-2.77, 2.1) | Low ^a, p^ | -1.62 (-5.91, 2.66) | Very low ^i^ | -0.65 (-2.76, 1.47) | Very low ^n^ |
| SJC vs Usual care | -6.89 (-11.02, -2.76) | Moderate ^p^ | -5.24 (-9.3, -1.18) | Moderate ^e^ | -6.05 (-8.95, -3.16) | Moderate |
| SJC vs Wuling | — | — | -4.07 (-7.19, -0.95) | Low ^h^ | -4.07 (-7.19, -0.95) | Low |
| SJC vs Wuling + Flupentixol/Melitracen | — | — | 0.23 (-3.38, 3.84) | Low ^j^ | 0.23 (-3.38, 3.84) | Very low ^n^ |
| SJC vs Wuling + Hypericum | — | — | 9.48 (2.26, 16.69) | Low ^h^ | 9.48 (2.26, 16.69) | Low |
| SJC vs Wuling + SNRI | — | — | 4.04 (-5.38, 13.45) | Moderate ^g^ | 4.04 (-5.38, 13.45) | Low ^n^ |
| SJC vs Wuling + SSRI | — | — | 3.16 (0.03, 6.29) | Low ^j^ | 3.16 (0.03, 6.29) | Low |
| SJC + Flupentixol/Melitracen vs SJC + SNRI | — | — | 6.64 (-2.78, 16.06) | Low ^h^ | 6.64 (-2.78, 16.06) | Very low ^n^ |
| SJC + Flupentixol/Melitracen vs SJC + SSRI | — | — | 2.81 (-2.47, 8.09) | Very low ^i^ | 2.81 (-2.47, 8.09) | Very low ^n^ |
| SJC + Flupentixol/Melitracen vs SNRI | — | — | 0.76 (-7.66, 9.17) | Low ^h^ | 0.76 (-7.66, 9.17) | Very low ^n^ |
| SJC + Flupentixol/Melitracen vs SSRI | — | — | -1.27 (-6.17, 3.64) | Low ^h^ | -1.27 (-6.17, 3.64) | Very low ^n^ |
| SJC + Flupentixol/Melitracen vs Usual care | — | — | -6.67 (-11.84, -1.5) | Moderate ^g^ | -6.67 (-11.84, -1.5) | Moderate |
| SJC + Flupentixol/Melitracen vs Wuling | — | — | -4.69 (-9.82, 0.44) | Moderate ^g^ | -4.69 (-9.82, 0.44) | Low ^n^ |
| SJC + Flupentixol/Melitracen vs Wuling + Flupentixol/Melitracen | — | — | -0.39 (-5.48, 4.71) | Moderate ^g^ | -0.39 (-5.48, 4.71) | Low ^n^ |
| SJC + Flupentixol/Melitracen vs Wuling + Hypericum | — | — | 8.86 (0.44, 17.27) | Low ^h^ | 8.86 (0.44, 17.27) | Low |
| SJC + Flupentixol/Melitracen vs Wuling + SNRI | — | — | 3.42 (-6.95, 13.78) | Moderate ^g^ | 3.42 (-6.95, 13.78) | Low ^n^ |
| SJC + Flupentixol/Melitracen vs Wuling + SSRI | — | — | 2.54 (-2.83, 7.91) | Low ^h^ | 2.54 (-2.83, 7.91) | Very low ^n^ |
| SJC + SNRI vs SJC + SSRI | — | — | -3.82 (-12.36, 4.71) | Low ^h^ | -3.82 (-12.36, 4.71) | Very low ^n^ |
| SJC + SNRI vs SNRI | -5.88 (-10.12, -1.64) | Low ^b^ | — | — | -5.88 (-10.12, -1.64) | Low |
| SJC + SNRI vs SSRI | — | — | -7.91 (-16.2, 0.39) | Low ^h^ | -7.91 (-16.2, 0.39) | Very low ^n^ |
| SJC + SNRI vs Usual care | — | — | -13.31 (-21.42, -5.21) | Low ^h^ | -13.31 (-21.42, -5.21) | Low |
| SJC + SNRI vs Wuling | — | — | -11.33 (-19.9, -2.76) | Low ^h^ | -11.33 (-19.9, -2.76) | Low |
| SJC + SNRI vs Wuling + Flupentixol/Melitracen | — | — | -7.03 (-15.76, 1.71) | Low ^h^ | -7.03 (-15.76, 1.71) | Very low ^n^ |
| SJC + SNRI vs Wuling + Hypericum | — | — | 2.22 (-7.14, 11.58) | Low ^h^ | 2.22 (-7.14, 11.58) | Very low ^n^ |
| SJC + SNRI vs Wuling + SNRI | — | — | -3.22 (-10.61, 4.16) | Low ^h^ | -3.22 (-10.61, 4.16) | Very low ^n^ |
| SJC + SNRI vs Wuling + SSRI | — | — | -4.1 (-12.7, 4.5) | Low ^h^ | -4.1 (-12.7, 4.5) | Very low ^n^ |
| SJC + SSRI vs SNRI | — | — | -2.06 (-9.46, 5.35) | Very low ^i^ | -2.06 (-9.46, 5.35) | Very low ^n^ |
| SJC + SSRI vs SSRI | -4.15 (-6.27, -2.03) | Very low ^b, p^ | -2.13 (-13.34, 9.08) | Low ^h^ | -4.08 (-6.17, -1.99) | Very low ^m^ |
| SJC + SSRI vs Usual care | — | — | -9.49 (-13.04, -5.94) | Very low ^i^ | -9.49 (-13.04, -5.94) | Very low |
| SJC + SSRI vs Wuling | — | — | -7.5 (-10.87, -4.14) | Very low ^k^ | -7.5 (-10.87, -4.14) | Very low |
| SJC + SSRI vs Wuling + Flupentixol/Melitracen | — | — | -3.2 (-7.29, 0.89) | Very low ^k^ | -3.2 (-7.29, 0.89) | Very low ^n^ |
| SJC + SSRI vs Wuling + Hypericum | — | — | 6.04 (-1.37, 13.45) | Very low ^i^ | 6.04 (-1.37, 13.45) | Very low ^n^ |
| SJC + SSRI vs Wuling + SNRI | — | — | 0.6 (-8.96, 10.16) | Moderate ^g^ | 0.6 (-8.96, 10.16) | Low ^n^ |
| SJC + SSRI vs Wuling + SSRI | — | — | -0.28 (-3.44, 2.89) | Very low ^k^ | -0.28 (-3.44, 2.89) | Very low ^n^ |
| SNRI vs SSRI | — | — | -2.02 (-9.15, 5.1) | Low ^h^ | -2.02 (-9.15, 5.1) | Very low ^n^ |
| SNRI vs Usual care | — | — | -7.43 (-14.34, -0.52) | Low ^h^ | -7.43 (-14.34, -0.52) | Low |
| SNRI vs Wuling | — | — | -5.44 (-12.89, 2.01) | Low ^h^ | -5.44 (-12.89, 2.01) | Very low ^n^ |
| SNRI vs Wuling + Flupentixol/Melitracen | — | — | -1.14 (-8.78, 6.5) | Low ^h^ | -1.14 (-8.78, 6.5) | Very low ^n^ |
| SNRI vs Wuling + Hypericum | — | — | 8.1 (-0.25, 16.45) | Moderate ^g^ | 8.1 (-0.25, 16.45) | Low ^n^ |
| SNRI vs Wuling + SNRI | 2.66 (-3.39, 8.71) | Moderate ^a^ | — | — | 2.66 (-3.39, 8.71) | Low ^n^ |
| SNRI vs Wuling + SSRI | — | — | 1.78 (-5.7, 9.27) | Low ^h^ | 1.78 (-5.7, 9.27) | Very low ^n^ |
| Usual care vs SSRI | 5.4 (-0.44, 11.24) | High | 5.41 (2.01, 8.81) | Low ^h^ | 5.41 (2.47, 8.34) | Moderate ^m^ |
| Wuling vs SSRI | 2.44 (-1.04, 5.92) | Low ^a, p^ | 4.84 (0.66, 9.02) | Very low ^k^ | 3.42 (0.75, 6.09) | Very low ^m^ |
| Wuling + Flupentixol/Melitracen vs SSRI | — | — | -0.88 (-4.44, 2.68) | Low ^j^ | -0.88 (-4.44, 2.68) | Very low ^n^ |
| Wuling + Hypericum vs SSRI | — | — | -10.12 (-17.25, -2.99) | Moderate ^g^ | -10.12 (-17.25, -2.99) | Moderate |
| Wuling + SNRI vs SSRI | — | — | -4.68 (-14.03, 4.66) | Moderate ^g^ | -4.68 (-14.03, 4.66) | Low ^n^ |
| Wuling + SSRI vs SSRI | -3.01 (-5.46, -0.56) | Moderate ^a^ | -18.64 (-29.21, -8.06) | Very low ^k^ | -3.81 (-6.19, -1.42) | Moderate |
| Usual care vs Wuling | — | — | 1.98 (-1.51, 5.48) | Very low ^k^ | 1.98 (-1.51, 5.48) | Very low ^n^ |
| Usual care vs Wuling + Flupentixol/Melitracen | 4.89 (-0.97, 10.75) | Moderate ^a^ | 7.22 (2.43, 12) | Very low ^k^ | 6.29 (2.58, 9.99) | Low ^m^ |
| Usual care vs Wuling + Hypericum | — | — | 15.53 (8.61, 22.44) | Low ^h^ | 15.53 (8.61, 22.44) | Low |
| Usual care vs Wuling + SNRI | — | — | 10.09 (0.91, 19.27) | Moderate ^g^ | 10.09 (0.91, 19.27) | Moderate |
| Usual care vs Wuling + SSRI | — | — | 9.21 (5.52, 12.9) | Very low ^k^ | 9.21 (5.52, 12.9) | Very Low |
| Wuling vs Wuling + Flupentixol/Melitracen | 5.44 (1.16, 9.72) | Low ^a, p^ | 2.56 (-2.73, 7.85) | Very low ^i^ | 4.3 (0.98, 7.63) | Very low ^m^ |
| Wuling vs Wuling + Hypericum | — | — | 13.54 (6.09, 21) | Low ^h^ | 13.54 (6.09, 21) | Low |
| Wuling vs Wuling + SNRI | — | — | 8.1 (-1.49, 17.7) | Moderate ^g^ | 8.1 (-1.49, 17.7) | Low ^n^ |
| Wuling vs Wuling + SSRI | 11.35 (7.03, 15.67) | Very low ^b, p^ | 2.39 (-2.29, 7.07) | Very low ^k^ | 7.23 (4.05, 10.4) | Very low ^m^ |
| Wuling + Flupentixol/Melitracen vs Wuling + Hypericum | — | — | 9.24 (1.6, 16.88) | Low ^h^ | 9.24 (1.6, 16.88) | Low |
| Wuling + Flupentixol/Melitracen vs Wuling + SNRI | — | — | 3.8 (-5.94, 13.54) | Moderate ^g^ | 3.8 (-5.94, 13.54) | Low ^n^ |
| Wuling + Flupentixol/Melitracen vs Wuling + SSRI | — | — | 2.93 (-1.16, 7.01) | Very low ^k^ | 2.93 (-1.16, 7.01) | Very low ^n^ |
| Wuling + Hypericum vs Wuling + SNRI | — | — | -5.44 (-15.75, 4.87) | Moderate ^g^ | -5.44 (-15.75, 4.87) | Low ^n^ |
| Wuling + Hypericum vs Wuling + SSRI | — | — | -6.32 (-13.81, 1.17) | Low ^h^ | -6.32 (-13.81, 1.17) | Very low ^n^ |
| Wuling + SNRI vs Wuling + SSRI | — | — | -0.88 (-10.5, 8.74) | Moderate ^g^ | -0.88 (-10.5, 8.74) | Low ^n^ |

**Outcome: All-cause drop out**

| Comparison | Direct estimate | Certainty | Indirect estimate | Certainty | Network estimate | Certainty |
| --- | --- | --- | --- | --- | --- | --- |
| FEW vs Hypericum | — | — | 0.04 (0, 2.81) | High | 0.04 (0, 2.81) | Moderate ^n^ |
| FEW vs SJC | — | — | 0.4 (0.01, 18.43) | High | 0.4 (0.01, 18.43) | Moderate ^n^ |
| FEW vs SJC + SSRI | — | — | 0.11 (0, 4.84) | Moderate ^d^ | 0.11 (0, 4.84) | Low ^n^ |
| FEW vs SSRI | 0.2 (0.01, 4.08) | High | — | — | 0.2 (0.01, 4.08) | Moderate ^n^ |
| FEW vs Usual care | 0.1 (0, 2.04) | High | — | — | 0.1 (0, 2.04) | Moderate ^n^ |
| FEW vs Wuling capsule | — | — | 1 (0.01, 70.43) | Moderate ^d^ | 1 (0.01, 70.43) | Low ^n^ |
| FEW vs Wuling capsule + SSRI | — | — | 0.2 (0.01, 6.35) | Moderate ^d^ | 0.2 (0.01, 6.35) | Low ^n^ |
| Hypericum vs SJC | — | — | 11.19 (0.15, 822.89) | High | 11.19 (0.15, 822.89) | Low ^o^ |
| Hypericum vs SJC + SSRI | — | — | 2.94 (0.04, 216.04) | Moderate ^d^ | 2.94 (0.04, 216.04) | Very Low ^o^ |
| Hypericum vs SSRI | — | — | 5.6 (0.15, 202.83) | High | 5.6 (0.15, 202.83) | Low ^o^ |
| Hypericum vs Usual care | 2.82 (0.12, 66.72) | High | — | — | 2.82 (0.12, 66.72) | Moderate ^n^ |
| Hypericum vs Wuling capsule | — | — | 27.98 (0.26, 3014.39) | Moderate ^d^ | 27.98 (0.26, 3014.39) | Very Low ^o^ |
| Hypericum vs Wuling capsule + SSRI | — | — | 5.6 (0.11, 296.21) | High | 5.6 (0.11, 296.21) | Low ^o^ |
| SJC vs SJC + SSRI | — | — | 0.26 (0.01, 7.41) | Moderate ^d^ | 0.26 (0.01, 7.41) | Low ^n^ |
| SJC vs SSRI | 0.5 (0.05, 5.31) | High | — | — | 0.5 (0.05, 5.31) | Moderate ^n^ |
| SJC vs Usual care | — | — | 0.25 (0.01, 4.62) | High | 0.25 (0.01, 4.62) | Moderate ^n^ |
| SJC vs Wuling capsule | — | — | 2.5 (0.05, 113.95) | Moderate ^d^ | 2.5 (0.05, 113.95) | Very Low ^o^ |
| SJC vs Wuling capsule + SSRI | — | — | 0.5 (0.03, 9.14) | Moderate ^d^ | 0.5 (0.03, 9.14) | Low ^n^ |
| SJC + SSRI vs SSRI | 1.9 (0.18, 20.15) | Moderate ^a^ | — | — | 1.9 (0.18, 20.15) | Low ^n^ |
| SJC + SSRI vs Usual care | — | — | 0.96 (0.05, 17.56) | Moderate ^d^ | 0.96 (0.05, 17.56) | Low ^n^ |
| SJC + SSRI vs Wuling capsule | — | — | 9.51 (0.21, 433.04) | Moderate ^d^ | 9.51 (0.21, 433.04) | Very Low ^o^ |
| SJC + SSRI vs Wuling capsule + SSRI | — | — | 1.9 (0.1, 34.71) | Moderate ^d^ | 1.9 (0.1, 34.71) | Low ^n^ |
| Usual care vs SSRI | 1.98 (0.36, 10.84) | High | — | — | 1.98 (0.36, 10.84) | Moderate ^n^ |
| Wuling capsule vs SSRI | 0.2 (0.01, 4.02) | Moderate ^a^ | — | — | 0.2 (0.01, 4.02) | Low ^n^ |
| Wuling capsule + SSRI vs SSRI | 1 (0.18, 5.43) | Moderate ^a^ | — | — | 1 (0.18, 5.43) | Low ^n^ |
| Usual care vs Wuling capsule | — | — | 9.92 (0.32, 311.98) | Moderate ^d^ | 9.92 (0.32, 311.98) | Very Low ^o^ |
| Usual care vs Wuling capsule + SSRI | — | — | 1.98 (0.18, 21.8) | Moderate ^d^ | 1.98 (0.18, 21.8) | Low ^n^ |
| Wuling capsule vs Wuling capsule + SSRI | 0.2 (0.01, 4.02) | Moderate ^a^ | — | — | 0.2 (0.01, 4.02) | Low ^n^ |

**Outcome: Any gastrointestinal event**

| Comparison | Direct Estimate | Certainty | Indirect Estimate | Certainty | Network Estimate | Certainty |
| --- | --- | --- | --- | --- | --- | --- |
| FEW vs FEW + SSRI | 1 (0.52, 1.92) | Moderate ^a^ | — | — | 1 (0.52, 1.92) | Low ^n^ |
| FEW vs Ginkgo biloba extract + SNRI | — | — | 0.25 (0, 13.01) | Moderate ^d^ | 0.25 (0, 13.01) | Low ^n^ |
| FEW vs Hypericum | — | — | 0.65 (0.03, 12) | Moderate ^d^ | 0.65 (0.03, 12) | Low ^n^ |
| FEW vs Jie-Yu + SSRI | — | — | 0.4 (0.07, 2.27) | Low ^e^ | 0.4 (0.07, 2.27) | Very low ^n^ |
| FEW vs Morinda Oligosaccharides | — | — | 0.93 (0.01, 127.11) | Moderate ^d^ | 0.93 (0.01, 127.11) | Very low ^o^ |
| FEW vs Morinda Oligosaccharides + SNRI | — | — | 0.19 (0, 13) | Moderate ^d^ | 0.19 (0, 13) | Low ^n^ |
| FEW vs SJC | — | — | 0.99 (0.19, 5.28) | Moderate ^d^ | 0.99 (0.19, 5.28) | Low ^n^ |
| FEW vs SJC + SNRI | — | — | 0.21 (0, 10.71) | Moderate ^d^ | 0.21 (0, 10.71) | Low ^n^ |
| FEW vs SJC + SSRI | — | — | 0.37 (0.07, 1.87) | Low ^e^ | 0.37 (0.07, 1.87) | Very low ^n^ |
| FEW vs SNRI | — | — | 0.31 (0.01, 13.33) | Moderate ^d^ | 0.31 (0.01, 13.33) | Low ^n^ |
| FEW vs SSRI | 0.33 (0.07, 1.59) | High | — | — | 0.33 (0.07, 1.59) | Moderate ^n^ |
| FEW vs Usual care | 0.33 (0.06, 1.89) | High | — | — | 0.33 (0.06, 1.89) | Moderate ^n^ |
| FEW vs Wuling | — | — | 0.93 (0.14, 6.12) | Moderate ^d^ | 0.93 (0.14, 6.12) | Low ^n^ |
| FEW vs Wuling + Hypericum | — | — | 0.44 (0.01, 13.22) | Moderate ^d^ | 0.44 (0.01, 13.22) | Low ^n^ |
| FEW vs Wuling + SNRI | — | — | 0.93 (0.01, 126.87) | Moderate ^d^ | 0.93 (0.01, 126.87) | Very low ^o^ |
| FEW vs Wuling + SSRI | — | — | 0.37 (0.07, 2.03) | Moderate ^d^ | 0.37 (0.07, 2.03) | Low ^n^ |
| FEW + SSRI vs Ginkgo biloba extract + SNRI | — | — | 0.25 (0, 13.73) | Moderate ^d^ | 0.25 (0, 13.73) | Low ^n^ |
| FEW + SSRI vs Hypericum | — | — | 0.65 (0.03, 12.9) | Moderate ^d^ | 0.65 (0.03, 12.9) | Low ^n^ |
| FEW + SSRI vs Jie-Yu + SSRI | — | — | 0.4 (0.06, 2.56) | Low ^h^ | 0.4 (0.06, 2.56) | Very low ^n^ |
| FEW + SSRI vs Morinda Oligosaccharides | — | — | 0.93 (0.01, 132.73) | Moderate ^d^ | 0.93 (0.01, 132.73) | Very low ^o^ |
| FEW + SSRI vs Morinda Oligosaccharides + SNRI | — | — | 0.19 (0, 13.66) | Moderate ^d^ | 0.19 (0, 13.66) | Low ^n^ |
| FEW + SSRI vs SJC | — | — | 0.99 (0.16, 5.97) | Moderate ^d^ | 0.99 (0.16, 5.97) | Low ^n^ |
| FEW + SSRI vs SJC + SNRI | — | — | 0.21 (0, 11.31) | Moderate ^d^ | 0.21 (0, 11.31) | Low ^n^ |
| FEW + SSRI vs SJC + SSRI | — | — | 0.37 (0.06, 2.12) | Low ^h^ | 0.37 (0.06, 2.12) | Very low ^n^ |
| FEW + SSRI vs SNRI | — | — | 0.31 (0.01, 14.1) | Moderate ^d^ | 0.31 (0.01, 14.1) | Low ^n^ |
| FEW + SSRI vs SSRI | — | — | 0.33 (0.06, 1.81) | Moderate ^d^ | 0.33 (0.06, 1.81) | Low ^n^ |
| FEW + SSRI vs Usual care | — | — | 0.33 (0.05, 2.13) | Moderate ^d^ | 0.33 (0.05, 2.13) | Low ^n^ |
| FEW + SSRI vs Wuling | — | — | 0.93 (0.13, 6.84) | Moderate ^d^ | 0.93 (0.13, 6.84) | Low ^n^ |
| FEW + SSRI vs Wuling + Hypericum | — | — | 0.44 (0.01, 14.06) | Moderate ^d^ | 0.44 (0.01, 14.06) | Low ^n^ |
| FEW + SSRI vs Wuling + SNRI | — | — | 0.93 (0.01, 132.47) | Moderate ^d^ | 0.93 (0.01, 132.47) | Very low ^o^ |
| FEW + SSRI vs Wuling + SSRI | — | — | 0.37 (0.06, 2.3) | Moderate ^d^ | 0.37 (0.06, 2.3) | Low ^n^ |
| Ginkgo biloba extract + SNRI vs Hypericum | — | — | 2.6 (0.18, 37.7) | Moderate ^d^ | 2.6 (0.18, 37.7) | Low ^n^ |
| Ginkgo biloba extract + SNRI vs Jie-Yu + SSRI | — | — | 1.62 (0.03, 77.43) | Moderate ^d^ | 1.62 (0.03, 77.43) | Low ^n^ |
| Ginkgo biloba extract + SNRI vs Morinda Oligosaccharides | — | — | 3.75 (0.12, 112.53) | Moderate ^d^ | 3.75 (0.12, 112.53) | Very low ^o^ |
| Ginkgo biloba extract + SNRI vs Morinda Oligosaccharides + SNRI | — | — | 0.75 (0.07, 7.7) | Moderate ^d^ | 0.75 (0.07, 7.7) | Low ^n^ |
| Ginkgo biloba extract + SNRI vs SJC | — | — | 3.99 (0.09, 185.95) | Moderate ^d^ | 3.99 (0.09, 185.95) | Very low ^o^ |
| Ginkgo biloba extract + SNRI vs SJC + SNRI | — | — | 0.83 (0.15, 4.68) | Moderate ^d^ | 0.83 (0.15, 4.68) | Low ^n^ |
| Ginkgo biloba extract + SNRI vs SJC + SSRI | — | — | 1.47 (0.03, 67.51) | Low ^h^ | 1.47 (0.03, 67.51) | Very low ^n^ |
| Ginkgo biloba extract + SNRI vs SNRI | 1.25 (0.36, 4.32) | High | — | — | 1.25 (0.36, 4.32) | Moderate ^n^ |
| Ginkgo biloba extract + SNRI vs SSRI | — | — | 1.34 (0.03, 59.68) | Moderate ^d^ | 1.34 (0.03, 59.68) | Low ^n^ |
| Ginkgo biloba extract + SNRI vs Usual care | — | — | 1.34 (0.04, 47.18) | Moderate ^d^ | 1.34 (0.04, 47.18) | Low ^n^ |
| Ginkgo biloba extract + SNRI vs Wuling | — | — | 3.76 (0.07, 192.76) | Moderate ^d^ | 3.76 (0.07, 192.76) | Very low ^o^ |
| Ginkgo biloba extract + SNRI vs Wuling + Hypericum | — | — | 1.77 (0.07, 43.1) | Moderate ^d^ | 1.77 (0.07, 43.1) | Low ^n^ |
| Ginkgo biloba extract + SNRI vs Wuling + SNRI | — | — | 3.75 (0.13, 112.22) | Moderate ^d^ | 3.75 (0.13, 112.22) | Very low ^o^ |
| Ginkgo biloba extract + SNRI vs Wuling + SSRI | — | — | 1.51 (0.03, 70.94) | Moderate ^d^ | 1.51 (0.03, 70.94) | Low ^n^ |
| Hypericum vs Jie-Yu + SSRI | — | — | 0.62 (0.04, 10.18) | Moderate ^d^ | 0.62 (0.04, 10.18) | Low ^n^ |
| Hypericum vs Morinda Oligosaccharides | — | — | 1.44 (0.03, 75.13) | Moderate ^g^ | 1.44 (0.03, 75.13) | Low ^n^ |
| Hypericum vs Morinda Oligosaccharides + SNRI | — | — | 0.29 (0.01, 6.27) | Moderate ^g^ | 0.29 (0.01, 6.27) | Low ^n^ |
| Hypericum vs SJC | — | — | 1.53 (0.1, 24.21) | Moderate ^d^ | 1.53 (0.1, 24.21) | Low ^n^ |
| Hypericum vs SJC + SNRI | — | — | 0.32 (0.02, 4.55) | Moderate ^g^ | 0.32 (0.02, 4.55) | Low ^n^ |
| Hypericum vs SJC + SSRI | — | — | 0.57 (0.04, 8.73) | Low ^h^ | 0.57 (0.04, 8.73) | Very low ^n^ |
| Hypericum vs SNRI | 0.48 (0.04, 5.12) | Moderate ^a^ | — | — | 0.48 (0.04, 5.12) | Low ^n^ |
| Hypericum vs SSRI | — | — | 0.52 (0.03, 7.62) | Moderate ^d^ | 0.52 (0.03, 7.62) | Low ^n^ |
| Hypericum vs Usual care | 0.52 (0.05, 5.41) | Moderate ^a^ | — | — | 0.52 (0.05, 5.41) | Low ^n^ |
| Hypericum vs Wuling | — | — | 1.44 (0.08, 26.01) | Moderate ^d^ | 1.44 (0.08, 26.01) | Low ^n^ |
| Hypericum vs Wuling + Hypericum | 0.68 (0.12, 3.89) | Moderate ^a^ | — | — | 0.68 (0.12, 3.89) | Low ^n^ |
| Hypericum vs Wuling + SNRI | — | — | 1.44 (0.03, 74.95) | Moderate ^g^ | 1.44 (0.03, 74.95) | Low ^n^ |
| Hypericum vs Wuling + SSRI | — | — | 0.58 (0.04, 9.26) | Moderate ^d^ | 0.58 (0.04, 9.26) | Low ^n^ |
| Jie-Yu + SSRI vs Morinda Oligosaccharides | — | — | 2.32 (0.02, 294.51) | Low ^h^ | 2.32 (0.02, 294.51) | Very low ^o^ |
| Jie-Yu + SSRI vs Morinda Oligosaccharides + SNRI | — | — | 0.46 (0.01, 29.77) | Low ^h^ | 0.46 (0.01, 29.77) | Very low ^n^ |
| Jie-Yu + SSRI vs SJC | — | — | 2.47 (0.94, 6.5) | Low ^h^ | 2.47 (0.94, 6.5) | Very low ^n^ |
| Jie-Yu + SSRI vs SJC + SNRI | — | — | 0.52 (0.01, 24.38) | Low ^h^ | 0.52 (0.01, 24.38) | Very low ^n^ |
| Jie-Yu + SSRI vs SJC + SSRI | — | — | 0.91 (0.37, 2.23) | Low ^j^ | 0.91 (0.37, 2.23) | Very low ^n^ |
| Jie-Yu + SSRI vs SNRI | — | — | 0.77 (0.02, 30.19) | Low ^h^ | 0.77 (0.02, 30.19) | Very low ^n^ |
| Jie-Yu + SSRI vs SSRI | 0.83 (0.39, 1.77) | Moderate ^b^ | — | — | 0.83 (0.39, 1.77) | Low ^n^ |
| Jie-Yu + SSRI vs Usual care | — | — | 0.83 (0.18, 3.78) | Low ^e^ | 0.83 (0.18, 3.78) | Very low ^n^ |
| Jie-Yu + SSRI vs Wuling | — | — | 2.33 (0.64, 8.49) | Low ^h^ | 2.33 (0.64, 8.49) | Very low ^n^ |
| Jie-Yu + SSRI vs Wuling + Hypericum | — | — | 1.1 (0.04, 29.62) | Low ^h^ | 1.1 (0.04, 29.62) | Very low ^n^ |
| Jie-Yu + SSRI vs Wuling + SNRI | — | — | 2.32 (0.02, 293.93) | Low ^h^ | 2.32 (0.02, 293.93) | Very low ^o^ |
| Jie-Yu + SSRI vs Wuling + SSRI | — | — | 0.93 (0.34, 2.54) | Low ^h^ | 0.93 (0.34, 2.54) | Very low ^n^ |
| Morinda Oligosaccharides vs Morinda Oligosaccharides + SNRI | 0.2 (0.01, 4.02) | Moderate ^a^ | — | — | 0.2 (0.01, 4.02) | Low ^n^ |
| Morinda Oligosaccharides vs SJC | — | — | 1.06 (0.01, 132.2) | Moderate ^d^ | 1.06 (0.01, 132.2) | Very low ^o^ |
| Morinda Oligosaccharides vs SJC + SNRI | — | — | 0.22 (0.01, 6.57) | Moderate ^g^ | 0.22 (0.01, 6.57) | Low ^n^ |
| Morinda Oligosaccharides vs SJC + SSRI | — | — | 0.39 (0, 48.17) | Moderate ^d^ | 0.39 (0, 48.17) | Low ^n^ |
| Morinda Oligosaccharides vs SNRI | 0.33 (0.01, 7.92) | Moderate ^a^ | — | — | 0.33 (0.01, 7.92) | Low ^n^ |
| Morinda Oligosaccharides vs SSRI | — | — | 0.36 (0, 42.85) | Moderate ^d^ | 0.36 (0, 42.85) | Low ^n^ |
| Morinda Oligosaccharides vs Usual care | — | — | 0.36 (0, 35.64) | Moderate ^g^ | 0.36 (0, 35.64) | Low ^n^ |
| Morinda Oligosaccharides vs Wuling | — | — | 1 (0.01, 134.47) | Moderate ^d^ | 1 (0.01, 134.47) | Very low ^o^ |
| Morinda Oligosaccharides vs Wuling + Hypericum | — | — | 0.47 (0.01, 35.62) | Moderate ^g^ | 0.47 (0.01, 35.62) | Low ^n^ |
| Morinda Oligosaccharides vs Wuling + SNRI | — | — | 1 (0.01, 88.01) | Moderate ^g^ | 1 (0.01, 88.01) | Low ^n^ |
| Morinda Oligosaccharides vs Wuling + SSRI | — | — | 0.4 (0, 50.36) | Moderate ^d^ | 0.4 (0, 50.36) | Low ^n^ |
| Morinda Oligosaccharides + SNRI vs SJC | — | — | 5.32 (0.08, 332.87) | Moderate ^d^ | 5.32 (0.08, 332.87) | Very low ^o^ |
| Morinda Oligosaccharides + SNRI vs SJC + SNRI | — | — | 1.11 (0.11, 11.17) | Moderate ^g^ | 1.11 (0.11, 11.17) | Low ^n^ |
| Morinda Oligosaccharides + SNRI vs SJC + SSRI | — | — | 1.96 (0.03, 121) | Low ^h^ | 1.96 (0.03, 121) | Very low ^o^ |
| Morinda Oligosaccharides + SNRI vs SNRI | 1.67 (0.23, 11.97) | Moderate ^a^ | — | — | 1.67 (0.23, 11.97) | Low ^n^ |
| Morinda Oligosaccharides + SNRI vs SSRI | — | — | 1.79 (0.03, 107.2) | Moderate ^d^ | 1.79 (0.03, 107.2) | Very low ^o^ |
| Morinda Oligosaccharides + SNRI vs Usual care | — | — | 1.79 (0.04, 86.29) | Moderate ^g^ | 1.79 (0.04, 86.29) | Low ^n^ |
| Morinda Oligosaccharides + SNRI vs Wuling | — | — | 5.01 (0.07, 342.78) | Moderate ^d^ | 5.01 (0.07, 342.78) | Very low ^o^ |
| Morinda Oligosaccharides + SNRI vs Wuling + Hypericum | — | — | 2.37 (0.07, 81.48) | Moderate ^g^ | 2.37 (0.07, 81.48) | Low ^n^ |
| Morinda Oligosaccharides + SNRI vs Wuling + SNRI | — | — | 5 (0.12, 208.08) | Moderate ^g^ | 5 (0.12, 208.08) | Low ^n^ |
| Morinda Oligosaccharides + SNRI vs Wuling + SSRI | — | — | 2.01 (0.03, 126.92) | Moderate ^d^ | 2.01 (0.03, 126.92) | Very low ^o^ |
| SJC vs SJC + SNRI | — | — | 0.21 (0, 9.62) | Moderate ^d^ | 0.21 (0, 9.62) | Low ^n^ |
| SJC vs SJC + SSRI | — | — | 0.37 (0.17, 0.8) | Low ^h^ | 0.37 (0.17, 0.8) | Low |
| SJC vs SNRI | — | — | 0.31 (0.01, 11.9) | Moderate ^d^ | 0.31 (0.01, 11.9) | Low ^n^ |
| SJC vs SSRI | 0.34 (0.18, 0.62) | Moderate ^a^ | — | — | 0.34 (0.18, 0.62) | Moderate |
| SJC vs Usual care | — | — | 0.34 (0.08, 1.43) | Moderate ^d^ | 0.34 (0.08, 1.43) | Low ^n^ |
| SJC vs Wuling | — | — | 0.94 (0.28, 3.17) | Moderate ^g^ | 0.94 (0.28, 3.17) | Low ^n^ |
| SJC vs Wuling + Hypericum | — | — | 0.44 (0.02, 11.64) | Moderate ^d^ | 0.44 (0.02, 11.64) | Low ^n^ |
| SJC vs Wuling + SNRI | — | — | 0.94 (0.01, 116.66) | Moderate ^g^ | 0.94 (0.01, 116.66) | Very low ^o^ |
| SJC vs Wuling + SSRI | — | — | 0.38 (0.16, 0.92) | Moderate ^d^ | 0.38 (0.16, 0.92) | Moderate |
| SJC + SNRI vs SJC + SSRI | — | — | 1.77 (0.04, 80) | Low ^h^ | 1.77 (0.04, 80) | Very low ^n^ |
| SJC + SNRI vs SNRI | 1.5 (0.45, 4.98) | Moderate ^a^ | — | — | 1.5 (0.45, 4.98) | Low ^n^ |
| SJC + SNRI vs SSRI | — | — | 1.61 (0.04, 70.71) | Moderate ^d^ | 1.61 (0.04, 70.71) | Low ^n^ |
| SJC + SNRI vs Usual care | — | — | 1.61 (0.05, 55.85) | Moderate ^g^ | 1.61 (0.05, 55.85) | Low ^n^ |
| SJC + SNRI vs Wuling | — | — | 4.51 (0.09, 228.5) | Moderate ^d^ | 4.51 (0.09, 228.5) | Very low ^o^ |
| SJC + SNRI vs Wuling + Hypericum | — | — | 2.13 (0.09, 50.94) | Moderate ^g^ | 2.13 (0.09, 50.94) | Low ^n^ |
| SJC + SNRI vs Wuling + SNRI | — | — | 4.5 (0.15, 132.77) | Moderate ^g^ | 4.5 (0.15, 132.77) | Very low ^o^ |
| SJC + SNRI vs Wuling + SSRI | — | — | 1.81 (0.04, 84.08) | Moderate ^d^ | 1.81 (0.04, 84.08) | Low ^n^ |
| SJC + SSRI vs SNRI | — | — | 0.85 (0.02, 31.63) | Low ^h^ | 0.85 (0.02, 31.63) | Very low ^n^ |
| SJC + SSRI vs SSRI | 0.91 (0.56, 1.48) | Moderate ^b^ | — | — | 0.91 (0.56, 1.48) | Very low ^n^ |
| SJC + SSRI vs Usual care | — | — | 0.91 (0.22, 3.7) | Low ^e^ | 0.91 (0.22, 3.7) | Very low ^n^ |
| SJC + SSRI vs Wuling | — | — | 2.55 (0.8, 8.12) | Low ^h^ | 2.55 (0.8, 8.12) | Very low ^n^ |
| SJC + SSRI vs Wuling + Hypericum | — | — | 1.2 (0.05, 30.87) | Low ^h^ | 1.2 (0.05, 30.87) | Very low ^n^ |
| SJC + SSRI vs Wuling + SNRI | — | — | 2.55 (0.02, 311.5) | Low ^h^ | 2.55 (0.02, 311.5) | Very low ^o^ |
| SJC + SSRI vs Wuling + SSRI | — | — | 1.02 (0.45, 2.31) | Low ^h^ | 1.02 (0.45, 2.31) | Very low ^n^ |
| SNRI vs SSRI | — | — | 1.07 (0.03, 38.77) | Moderate ^d^ | 1.07 (0.03, 38.77) | Low ^n^ |
| SNRI vs Usual care | — | — | 1.07 (0.04, 30.2) | Moderate ^g^ | 1.07 (0.04, 30.2) | Low ^n^ |
| SNRI vs Wuling | — | — | 3.01 (0.07, 126.23) | Moderate ^d^ | 3.01 (0.07, 126.23) | Very low ^o^ |
| SNRI vs Wuling + Hypericum | — | — | 1.42 (0.08, 26.83) | Moderate ^g^ | 1.42 (0.08, 26.83) | Low ^n^ |
| SNRI vs Wuling + SNRI | 3 (0.13, 71.04) | Moderate ^a^ | — | — | 3 (0.13, 71.04) | Low ^n^ |
| SNRI vs Wuling + SSRI | — | — | 1.21 (0.03, 46.24) | Moderate ^d^ | 1.21 (0.03, 46.24) | Low ^n^ |
| Usual care vs SSRI | 1 (0.27, 3.72) | High | — | — | 1 (0.27, 3.72) | Moderate ^n^ |
| Wuling vs SSRI | 0.4 (0.14, 1.16) | Moderate ^a^ | 0 (0, 3.02) | Low ^h^ | 0.36 (0.12, 1.02) | Low ^n^ |
| Wuling + Hypericum vs SSRI | — | — | 0.76 (0.03, 18.72) | Moderate ^d^ | 0.76 (0.03, 18.72) | Low ^n^ |
| Wuling + SNRI vs SSRI | — | — | 0.36 (0, 42.76) | Moderate ^d^ | 0.36 (0, 42.76) | Low ^n^ |
| Wuling + SSRI vs SSRI | 0.89 (0.46, 1.71) | Moderate ^b^ | — | — | 0.89 (0.46, 1.71) | Low ^n^ |
| Usual care vs Wuling | — | — | 2.8 (0.52, 15.07) | Moderate ^d^ | 2.8 (0.52, 15.07) | Low ^n^ |
| Usual care vs Wuling + Hypericum | — | — | 1.32 (0.07, 24.64) | Moderate ^g^ | 1.32 (0.07, 24.64) | Low ^n^ |
| Usual care vs Wuling + SNRI | — | — | 2.79 (0.03, 277.4) | Moderate ^d^ | 2.79 (0.03, 277.4) | Very low ^o^ |
| Usual care vs Wuling + SSRI | — | — | 1.12 (0.26, 4.88) | Moderate ^d^ | 1.12 (0.26, 4.88) | Low ^n^ |
| Wuling vs Wuling + Hypericum | — | — | 0.47 (0.02, 13.8) | Moderate ^d^ | 0.47 (0.02, 13.8) | Low ^n^ |
| Wuling vs Wuling + SNRI | — | — | 1 (0.01, 133.53) | Moderate ^d^ | 1 (0.01, 133.53) | Very low ^o^ |
| Wuling vs Wuling + SSRI | 0.36 (0.13, 1.04) | Moderate ^a^ | 80.23 (0.04, 169894.61) | Low ^h^ | 0.4 (0.14, 1.13) | Low ^n^ |
| Wuling + Hypericum vs Wuling + SNRI | — | — | 2.11 (0.03, 158.83) | Moderate ^g^ | 2.11 (0.03, 158.83) | Very low ^o^ |
| Wuling + Hypericum vs Wuling + SSRI | — | — | 0.85 (0.03, 22.47) | Moderate ^d^ | 0.85 (0.03, 22.47) | Low ^n^ |
| Wuling + SNRI vs Wuling + SSRI | — | — | 0.4 (0, 50.26) | Moderate ^d^ | 0.4 (0, 50.26) | Low ^n^ |

**Outcome: Any nervous system event**

| Comparison | Direct Estimate | Certainty | Indirect Estimate | Certainty | Network Estimate | Certainty |
| --- | --- | --- | --- | --- | --- | --- |
| FEW vs FEW + SSRI | 1 (0.22, 4.63) | Moderate ^a^ | — | — | 1 (0.22, 4.63) | Low ^n^ |
| FEW vs Ginkgo biloba extract + SNRI | — | — | 0.24 (0, 35.68) | Moderate ^d^ | 0.24 (0, 35.68) | Low ^n^ |
| FEW vs Hypericum | — | — | 0.2 (0, 8.87) | Moderate ^d^ | 0.2 (0, 8.87) | Low ^n^ |
| FEW vs Jie-Yu + SSRI | — | — | 0.09 (0, 2.23) | Low ^e^ | 0.09 (0, 2.23) | Very low ^n^ |
| FEW vs Morinda Oligosaccharides | — | — | 0.56 (0, 162.9) | Moderate ^d^ | 0.56 (0, 162.9) | Very low ^o^ |
| FEW vs Morinda Oligosaccharides + SNRI | — | — | 0.28 (0, 68.57) | Moderate ^d^ | 0.28 (0, 68.57) | Low ^n^ |
| FEW vs SJC | — | — | 1.03 (0.05, 23.48) | Moderate ^d^ | 1.03 (0.05, 23.48) | Low ^n^ |
| FEW vs SJC + SNRI | — | — | 0.68 (0, 109.62) | Moderate ^d^ | 0.68 (0, 109.62) | Very low ^o^ |
| FEW vs SJC + SSRI | — | — | 0.1 (0, 2.17) | Low ^e^ | 0.1 (0, 2.17) | Very low ^n^ |
| FEW vs SNRI | — | — | 0.56 (0, 80.68) | Moderate ^d^ | 0.56 (0, 80.68) | Low ^n^ |
| FEW vs SSRI | 0.11 (0.01, 2.02) | High | — | — | 0.11 (0.01, 2.02) | Moderate ^n^ |
| FEW vs Usual care | 0.1 (0, 2.04) | High | — | — | 0.1 (0, 2.04) | Moderate ^n^ |
| FEW vs Wuling | — | — | 0.33 (0.01, 12.68) | Moderate ^d^ | 0.33 (0.01, 12.68) | Low ^n^ |
| FEW vs Wuling + Hypericum | — | — | 0.2 (0, 21.93) | Moderate ^d^ | 0.2 (0, 21.93) | Low ^n^ |
| FEW vs Wuling + SSRI | — | — | 0.24 (0.01, 5.48) | Low ^e^ | 0.24 (0.01, 5.48) | Very low ^n^ |
| FEW + SSRI vs Ginkgo biloba extract + SNRI | — | — | 0.24 (0, 44.86) | Moderate ^d^ | 0.24 (0, 44.86) | Low ^n^ |
| FEW + SSRI vs Hypericum | — | — | 0.2 (0, 11.93) | Moderate ^d^ | 0.2 (0, 11.93) | Low ^n^ |
| FEW + SSRI vs Jie-Yu + SSRI | — | — | 0.09 (0, 3.17) | Low ^h^ | 0.09 (0, 3.17) | Very low ^n^ |
| FEW + SSRI vs Morinda Oligosaccharides | — | — | 0.56 (0, 199.66) | Moderate ^d^ | 0.56 (0, 199.66) | Very low ^o^ |
| FEW + SSRI vs Morinda Oligosaccharides + SNRI | — | — | 0.28 (0, 84.57) | Moderate ^d^ | 0.28 (0, 84.57) | Low ^n^ |
| FEW + SSRI vs SJC | — | — | 1.03 (0.03, 33.49) | Moderate ^d^ | 1.03 (0.03, 33.49) | Low ^n^ |
| FEW + SSRI vs SJC + SNRI | — | — | 0.68 (0, 137.38) | Moderate ^d^ | 0.68 (0, 137.38) | Very low ^o^ |
| FEW + SSRI vs SJC + SSRI | — | — | 0.1 (0, 3.1) | Low ^h^ | 0.1 (0, 3.1) | Very low ^n^ |
| FEW + SSRI vs SNRI | — | — | 0.56 (0, 101.66) | Moderate ^d^ | 0.56 (0, 101.66) | Very low ^o^ |
| FEW + SSRI vs SSRI | — | — | 0.11 (0, 2.95) | Moderate ^d^ | 0.11 (0, 2.95) | Low ^n^ |
| FEW + SSRI vs Usual care | — | — | 0.1 (0, 2.94) | Moderate ^d^ | 0.1 (0, 2.94) | Low ^n^ |
| FEW + SSRI vs Wuling | — | — | 0.33 (0.01, 17.24) | Moderate ^d^ | 0.33 (0.01, 17.24) | Low ^n^ |
| FEW + SSRI vs Wuling + Hypericum | — | — | 0.2 (0, 27.97) | Moderate ^d^ | 0.2 (0, 27.97) | Low ^n^ |
| FEW + SSRI vs Wuling + SSRI | — | — | 0.24 (0.01, 7.82) | Moderate ^d^ | 0.24 (0.01, 7.82) | Low ^n^ |
| Ginkgo biloba extract + SNRI vs Hypericum | — | — | 0.82 (0.03, 21.31) | Moderate ^d^ | 0.82 (0.03, 21.31) | Low ^n^ |
| Ginkgo biloba extract + SNRI vs Jie-Yu + SSRI | — | — | 0.4 (0, 34.51) | Low ^h^ | 0.4 (0, 34.51) | Very low ^n^ |
| Ginkgo biloba extract + SNRI vs Morinda Oligosaccharides | — | — | 2.37 (0.14, 39.9) | Moderate ^d^ | 2.37 (0.14, 39.9) | Low ^n^ |
| Ginkgo biloba extract + SNRI vs Morinda Oligosaccharides + SNRI | — | — | 1.19 (0.1, 13.86) | Moderate ^d^ | 1.19 (0.1, 13.86) | Low ^n^ |
| Ginkgo biloba extract + SNRI vs SJC | — | — | 4.34 (0.05, 366.21) | Moderate ^d^ | 4.34 (0.05, 366.21) | Very low ^o^ |
| Ginkgo biloba extract + SNRI vs SJC + SNRI | — | — | 2.85 (0.76, 10.65) | Moderate ^d^ | 2.85 (0.76, 10.65) | Low ^n^ |
| Ginkgo biloba extract + SNRI vs SJC + SSRI | — | — | 0.41 (0, 33.98) | Low ^h^ | 0.41 (0, 33.98) | Very low ^n^ |
| Ginkgo biloba extract + SNRI vs SNRI | 2.37 (1.18, 4.78) | High | — | — | 2.37 (1.18, 4.78) | High |
| Ginkgo biloba extract + SNRI vs SSRI | — | — | 0.47 (0.01, 33.79) | Moderate ^d^ | 0.47 (0.01, 33.79) | Low ^n^ |
| Ginkgo biloba extract + SNRI vs Usual care | — | — | 0.43 (0.01, 23.51) | Moderate ^d^ | 0.43 (0.01, 23.51) | Low ^n^ |
| Ginkgo biloba extract + SNRI vs Wuling | — | — | 1.37 (0.01, 171.45) | Moderate ^d^ | 1.37 (0.01, 171.45) | Very low ^o^ |
| Ginkgo biloba extract + SNRI vs Wuling + Hypericum | — | — | 0.84 (0.01, 59.28) | Moderate ^d^ | 0.84 (0.01, 59.28) | Low ^n^ |
| Ginkgo biloba extract + SNRI vs Wuling + SSRI | — | — | 1.01 (0.01, 85.38) | Moderate ^d^ | 1.01 (0.01, 85.38) | Low ^n^ |
| Hypericum vs Jie-Yu + SSRI | — | — | 0.49 (0.02, 10.22) | Low ^h^ | 0.49 (0.02, 10.22) | Very low ^n^ |
| Hypericum vs Morinda Oligosaccharides | — | — | 2.88 (0.04, 190.27) | Moderate ^g^ | 2.88 (0.04, 190.27) | Very low ^o^ |
| Hypericum vs Morinda Oligosaccharides + SNRI | — | — | 1.44 (0.03, 75.15) | Moderate ^g^ | 1.44 (0.03, 75.15) | Low ^n^ |
| Hypericum vs SJC | — | — | 5.26 (0.26, 107.45) | Moderate ^d^ | 5.26 (0.26, 107.45) | Very low ^o^ |
| Hypericum vs SJC + SNRI | — | — | 3.46 (0.12, 100.24) | Moderate ^g^ | 3.46 (0.12, 100.24) | Very low ^o^ |
| Hypericum vs SJC + SSRI | — | — | 0.49 (0.02, 9.94) | Low ^h^ | 0.49 (0.02, 9.94) | Very low ^n^ |
| Hypericum vs SNRI | 2.88 (0.12, 69.02) | Moderate ^a^ | — | — | 2.88 (0.12, 69.02) | Low ^n^ |
| Hypericum vs SSRI | — | — | 0.57 (0.04, 9.16) | Moderate ^d^ | 0.57 (0.04, 9.16) | Low ^n^ |
| Hypericum vs Usual care | 0.52 (0.05, 5.41) | Moderate ^a^ | — | — | 0.52 (0.05, 5.41) | Low ^n^ |
| Hypericum vs Wuling | — | — | 1.67 (0.05, 58.98) | Moderate ^d^ | 1.67 (0.05, 58.98) | Low ^n^ |
| Hypericum vs Wuling + Hypericum | 1.02 (0.07, 15.85) | Moderate ^a^ | — | — | 1.02 (0.07, 15.85) | Low ^n^ |
| Hypericum vs Wuling + SSRI | — | — | 1.22 (0.06, 25.1) | Moderate ^d^ | 1.22 (0.06, 25.1) | Low ^n^ |
| Jie-Yu + SSRI vs Morinda Oligosaccharides | — | — | 5.93 (0.03, 1054.9) | Low ^h^ | 5.93 (0.03, 1054.9) | Very low ^o^ |
| Jie-Yu + SSRI vs Morinda Oligosaccharides + SNRI | — | — | 2.97 (0.02, 436.7) | Low ^h^ | 2.97 (0.02, 436.7) | Very low ^o^ |
| Jie-Yu + SSRI vs SJC | — | — | 10.83 (1.95, 60) | Low ^h^ | 10.83 (1.95, 60) | Low |
| Jie-Yu + SSRI vs SJC + SNRI | — | — | 7.12 (0.08, 667.55) | Low ^h^ | 7.12 (0.08, 667.55) | Very low ^o^ |
| Jie-Yu + SSRI vs SJC + SSRI | — | — | 1.01 (0.19, 5.5) | Low ^j^ | 1.01 (0.19, 5.5) | Very low ^n^ |
| Jie-Yu + SSRI vs SNRI | — | — | 5.93 (0.07, 483.8) | Low ^h^ | 5.93 (0.07, 483.8) | Very low ^o^ |
| Jie-Yu + SSRI vs SSRI | 1.17 (0.34, 4.07) | Low ^b^ | — | — | 1.17 (0.34, 4.07) | Very low ^n^ |
| Jie-Yu + SSRI vs Usual care | — | — | 1.06 (0.15, 7.37) | Low ^e^ | 1.06 (0.15, 7.37) | Very low ^n^ |
| Jie-Yu + SSRI vs Wuling | — | — | 3.43 (0.27, 44.35) | Low ^h^ | 3.43 (0.27, 44.35) | Very low ^n^ |
| Jie-Yu + SSRI vs Wuling + Hypericum | — | — | 2.1 (0.03, 126.79) | Low ^h^ | 2.1 (0.03, 126.79) | Very low ^o^ |
| Jie-Yu + SSRI vs Wuling + SSRI | — | — | 2.51 (0.45, 14.08) | Low ^j^ | 2.51 (0.45, 14.08) | Very low ^n^ |
| Morinda Oligosaccharides vs Morinda Oligosaccharides + SNRI | 0.5 (0.05, 5.27) | Moderate ^a^ | — | — | 0.5 (0.05, 5.27) | Low ^n^ |
| Morinda Oligosaccharides vs SJC | — | — | 1.83 (0.01, 319.02) | Moderate ^d^ | 1.83 (0.01, 319.02) | Very low ^o^ |
| Morinda Oligosaccharides vs SJC + SNRI | — | — | 1.2 (0.06, 22.99) | Moderate ^g^ | 1.2 (0.06, 22.99) | Low ^n^ |
| Morinda Oligosaccharides vs SJC + SSRI | — | — | 0.17 (0, 29.64) | Low ^h^ | 0.17 (0, 29.64) | Very low ^n^ |
| Morinda Oligosaccharides vs SNRI | 1 (0.07, 15.38) | Moderate ^a^ | — | — | 1 (0.07, 15.38) | Low ^n^ |
| Morinda Oligosaccharides vs SSRI | — | — | 0.2 (0, 30.11) | Moderate ^d^ | 0.2 (0, 30.11) | Low ^n^ |
| Morinda Oligosaccharides vs Usual care | — | — | 0.18 (0, 21.85) | Moderate ^g^ | 0.18 (0, 21.85) | Low ^n^ |
| Morinda Oligosaccharides vs Wuling | — | — | 0.58 (0, 141.87) | Moderate ^d^ | 0.58 (0, 141.87) | Very low ^o^ |
| Morinda Oligosaccharides vs Wuling + Hypericum | — | — | 0.35 (0, 53.03) | Moderate ^g^ | 0.35 (0, 53.03) | Low ^n^ |
| Morinda Oligosaccharides vs Wuling + SSRI | — | — | 0.42 (0, 74.33) | Moderate ^d^ | 0.42 (0, 74.33) | Low ^n^ |
| Morinda Oligosaccharides + SNRI vs SJC | — | — | 3.65 (0.03, 527.91) | Moderate ^d^ | 3.65 (0.03, 527.91) | Very low ^o^ |
| Morinda Oligosaccharides + SNRI vs SJC + SNRI | — | — | 2.4 (0.18, 32.54) | Moderate ^g^ | 2.4 (0.18, 32.54) | Low ^n^ |
| Morinda Oligosaccharides + SNRI vs SJC + SSRI | — | — | 0.34 (0, 49.03) | Low ^h^ | 0.34 (0, 49.03) | Very low ^n^ |
| Morinda Oligosaccharides + SNRI vs SNRI | 2 (0.19, 21.09) | Moderate ^a^ | — | — | 2 (0.19, 21.09) | Low ^n^ |
| Morinda Oligosaccharides + SNRI vs SSRI | — | — | 0.39 (0, 49.57) | Moderate ^d^ | 0.39 (0, 49.57) | Low ^n^ |
| Morinda Oligosaccharides + SNRI vs Usual care | — | — | 0.36 (0, 35.62) | Moderate ^g^ | 0.36 (0, 35.62) | Low ^n^ |
| Morinda Oligosaccharides + SNRI vs Wuling | — | — | 1.16 (0.01, 237.62) | Moderate ^d^ | 1.16 (0.01, 237.62) | Very low ^o^ |
| Morinda Oligosaccharides + SNRI vs Wuling + Hypericum | — | — | 0.71 (0.01, 87.22) | Moderate ^g^ | 0.71 (0.01, 87.22) | Low ^n^ |
| Morinda Oligosaccharides + SNRI vs Wuling + SSRI | — | — | 0.85 (0.01, 123.02) | Moderate ^d^ | 0.85 (0.01, 123.02) | Very low ^o^ |
| SJC vs SJC + SNRI | — | — | 0.66 (0.01, 60.42) | Moderate ^d^ | 0.66 (0.01, 60.42) | Low ^n^ |
| SJC vs SJC + SSRI | — | — | 0.09 (0.02, 0.48) | Low ^h^ | 0.09 (0.02, 0.48) | Very low ^n^ |
| SJC vs SNRI | — | — | 0.55 (0.01, 43.76) | Moderate ^d^ | 0.55 (0.01, 43.76) | Low ^n^ |
| SJC vs SSRI | 0.11 (0.03, 0.35) | Moderate ^a^ | — | — | 0.11 (0.03, 0.35) | Moderate |
| SJC vs Usual care | — | — | 0.1 (0.01, 0.65) | Moderate ^d^ | 0.1 (0.01, 0.65) | Moderate |
| SJC vs Wuling | — | — | 0.32 (0.03, 3.95) | Moderate ^g^ | 0.32 (0.03, 3.95) | Low ^n^ |
| SJC vs Wuling + Hypericum | — | — | 0.19 (0, 11.45) | Moderate ^d^ | 0.19 (0, 11.45) | Low ^n^ |
| SJC vs Wuling + SSRI | — | — | 0.23 (0.04, 1.23) | Low ^h^ | 0.23 (0.04, 1.23) | Very low ^n^ |
| SJC + SNRI vs SJC + SSRI | — | — | 0.14 (0, 12.98) | Low ^h^ | 0.14 (0, 12.98) | Very low ^n^ |
| SJC + SNRI vs SNRI | 0.83 (0.27, 2.55) | Moderate ^a^ | — | — | 0.83 (0.27, 2.55) | Low ^n^ |
| SJC + SNRI vs SSRI | — | — | 0.16 (0, 12.94) | Moderate ^d^ | 0.16 (0, 12.94) | Low ^n^ |
| SJC + SNRI vs Usual care | — | — | 0.15 (0, 9.06) | Moderate ^g^ | 0.15 (0, 9.06) | Low ^n^ |
| SJC + SNRI vs Wuling | — | — | 0.48 (0, 65.03) | Moderate ^d^ | 0.48 (0, 65.03) | Low ^n^ |
| SJC + SNRI vs Wuling + Hypericum | — | — | 0.3 (0, 22.72) | Moderate ^g^ | 0.3 (0, 22.72) | Low ^n^ |
| SJC + SNRI vs Wuling + SSRI | — | — | 0.35 (0, 32.6) | Moderate ^d^ | 0.35 (0, 32.6) | Low ^n^ |
| SJC + SSRI vs SNRI | — | — | 5.86 (0.07, 464.33) | Low ^h^ | 5.86 (0.07, 464.33) | Very low ^o^ |
| SJC + SSRI vs SSRI | 1.16 (0.37, 3.62) | Low ^b^ | — | — | 1.16 (0.37, 3.62) | Very low ^n^ |
| SJC + SSRI vs Usual care | — | — | 1.05 (0.16, 6.82) | Low ^e^ | 1.05 (0.16, 6.82) | Very low ^n^ |
| SJC + SSRI vs Wuling | — | — | 3.39 (0.28, 41.69) | Low ^h^ | 3.39 (0.28, 41.69) | Very low ^n^ |
| SJC + SSRI vs Wuling + Hypericum | — | — | 2.08 (0.04, 121.43) | Low ^h^ | 2.08 (0.04, 121.43) | Very low ^o^ |
| SJC + SSRI vs Wuling + SSRI | — | — | 2.48 (0.48, 12.91) | Low ^j^ | 2.48 (0.48, 12.91) | Very low ^n^ |
| SNRI vs SSRI | — | — | 0.2 (0, 13.43) | Moderate ^d^ | 0.2 (0, 13.43) | Low ^n^ |
| SNRI vs Usual care | — | — | 0.18 (0, 9.31) | Moderate ^g^ | 0.18 (0, 9.31) | Low ^n^ |
| SNRI vs Wuling | — | — | 0.58 (0, 68.59) | Moderate ^d^ | 0.58 (0, 68.59) | Low ^n^ |
| SNRI vs Wuling + Hypericum | — | — | 0.35 (0.01, 23.55) | Moderate ^g^ | 0.35 (0.01, 23.55) | Low ^n^ |
| SNRI vs Wuling + SSRI | — | — | 0.42 (0.01, 34.01) | Low ^h^ | 0.42 (0.01, 34.01) | Very low ^n^ |
| Usual care vs SSRI | 1.1 (0.25, 4.86) | High | — | — | 1.1 (0.25, 4.86) | Moderate ^n^ |
| Wuling vs SSRI | 0.5 (0.05, 5.27) | Moderate ^a^ | 0.01 (0, 12.79) | Low ^h^ | 0.34 (0.04, 3.19) | Low ^n^ |
| Wuling + Hypericum vs SSRI | — | — | 0.56 (0.01, 27.59) | Moderate ^d^ | 0.56 (0.01, 27.59) | Low ^n^ |
| Wuling + SSRI vs SSRI | 0.47 (0.14, 1.53) | Low ^b^ | — | — | 0.47 (0.14, 1.53) | Very low ^n^ |
| Usual care vs Wuling | — | — | 3.23 (0.22, 47.24) | Moderate ^d^ | 3.23 (0.22, 47.24) | Low ^n^ |
| Usual care vs Wuling + Hypericum | — | — | 1.98 (0.05, 73.37) | Moderate ^g^ | 1.98 (0.05, 73.37) | Low ^n^ |
| Usual care vs Wuling + SSRI | — | — | 2.37 (0.35, 15.85) | Low ^e^ | 2.37 (0.35, 15.85) | Very low ^n^ |
| Wuling vs Wuling + Hypericum | — | — | 0.61 (0.01, 55.14) | Moderate ^d^ | 0.61 (0.01, 55.14) | Low ^n^ |
| Wuling vs Wuling + SSRI | 0.5 (0.05, 5.27) | Moderate ^a^ | 23.08 (0.02, 27238.89) | Low ^h^ | 0.73 (0.08, 6.85) | Low ^n^ |
| Wuling + Hypericum vs Wuling + SSRI | — | — | 1.2 (0.02, 70.78) | Moderate ^d^ | 1.2 (0.02, 70.78) | Low ^n^ |

## 8.2 Absolute contribution matrices

**Outcome: Response rate**


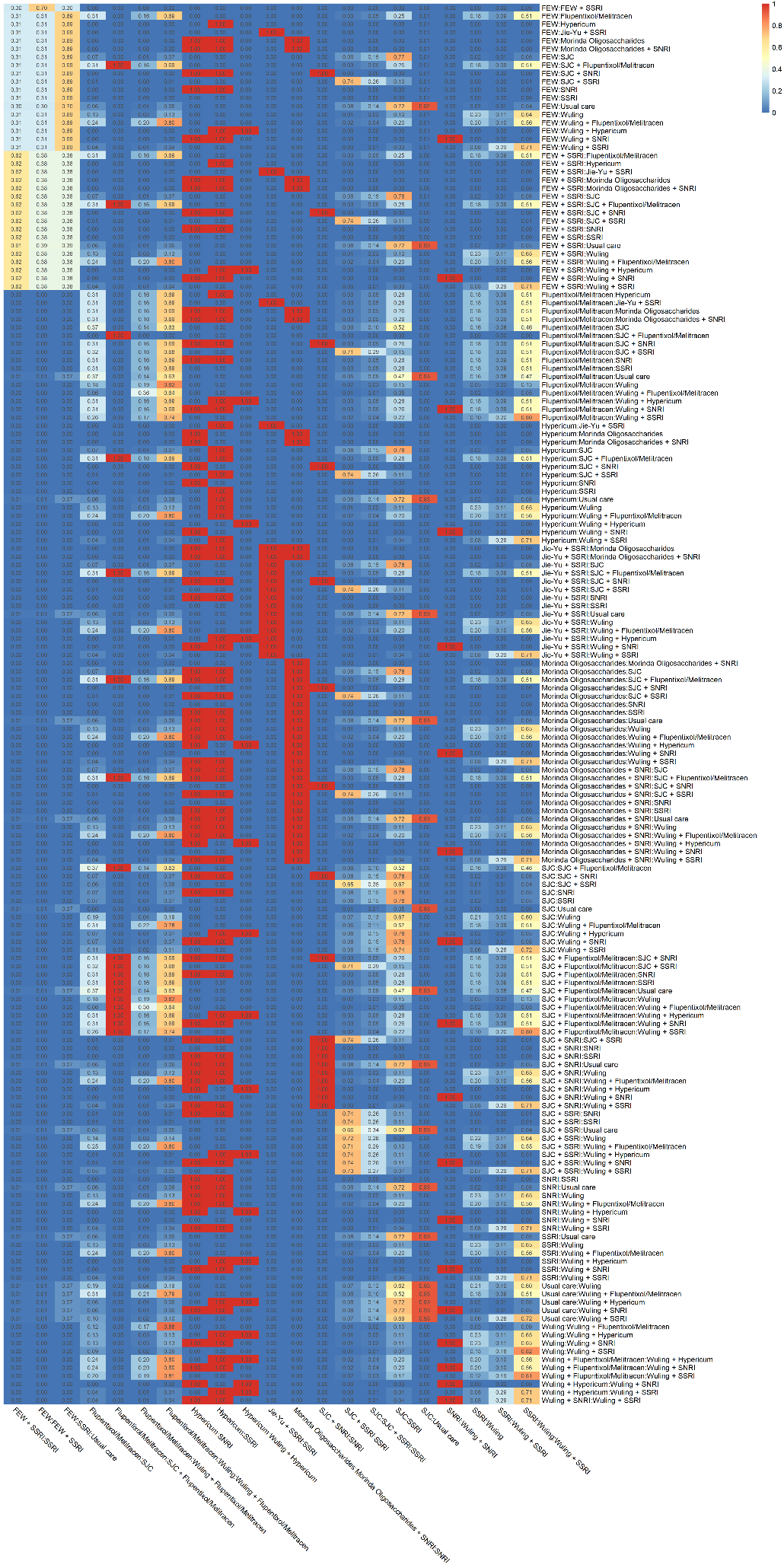


**Outcome: Mean changes in HAMD score from baseline**


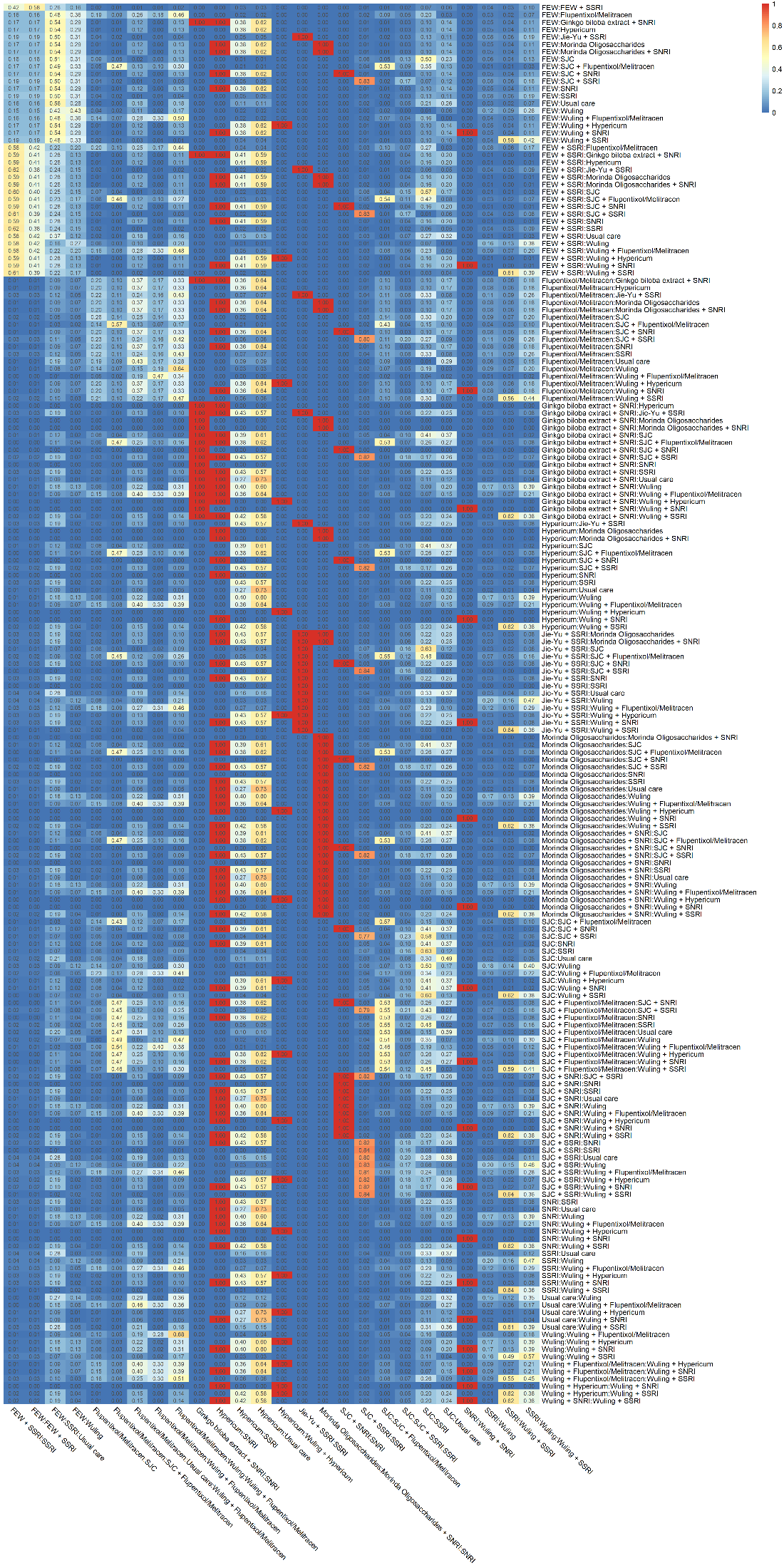


**Outcome: All-cause drop out**


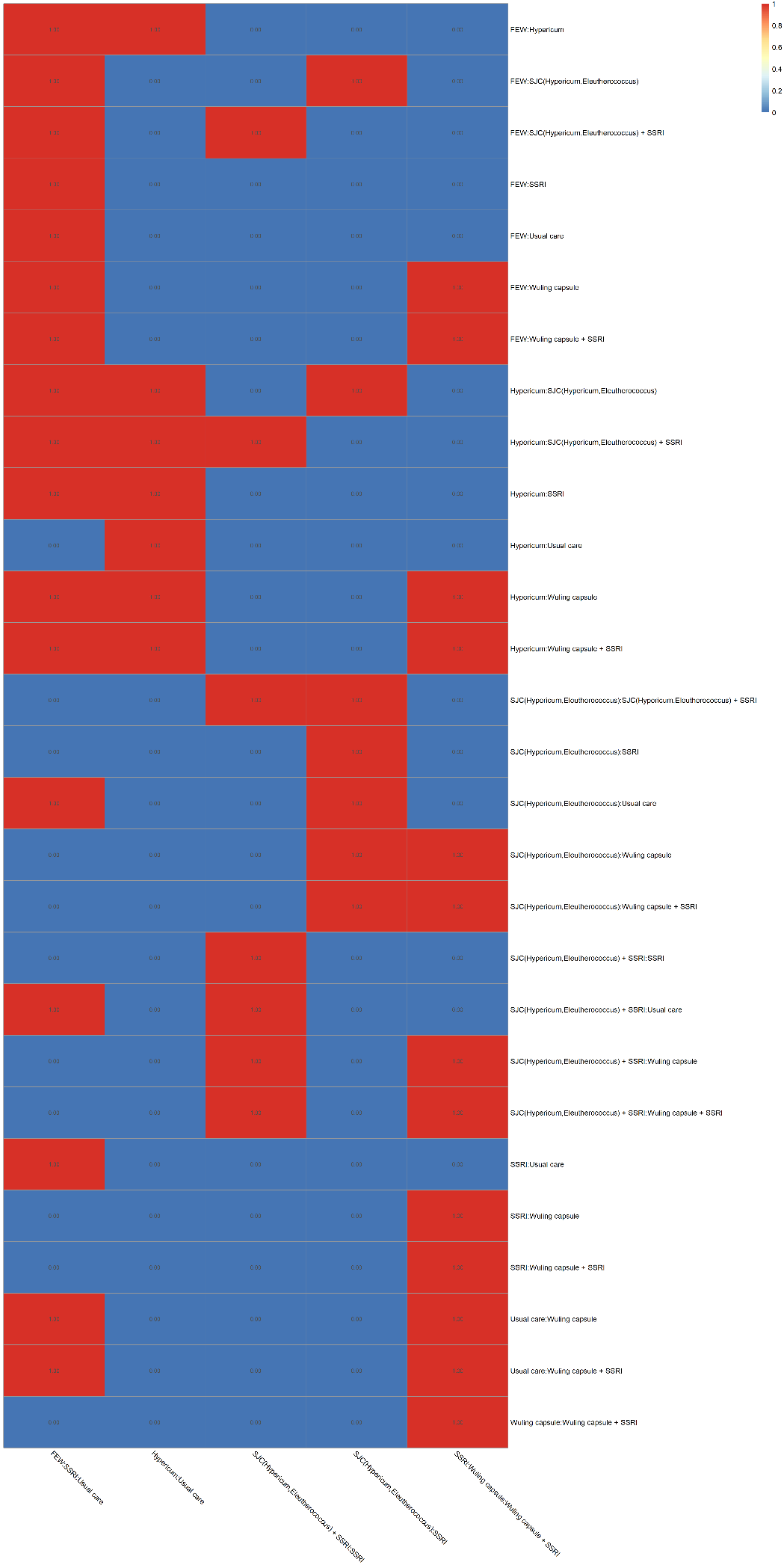


**Outcome: Any gastrointestinal event**


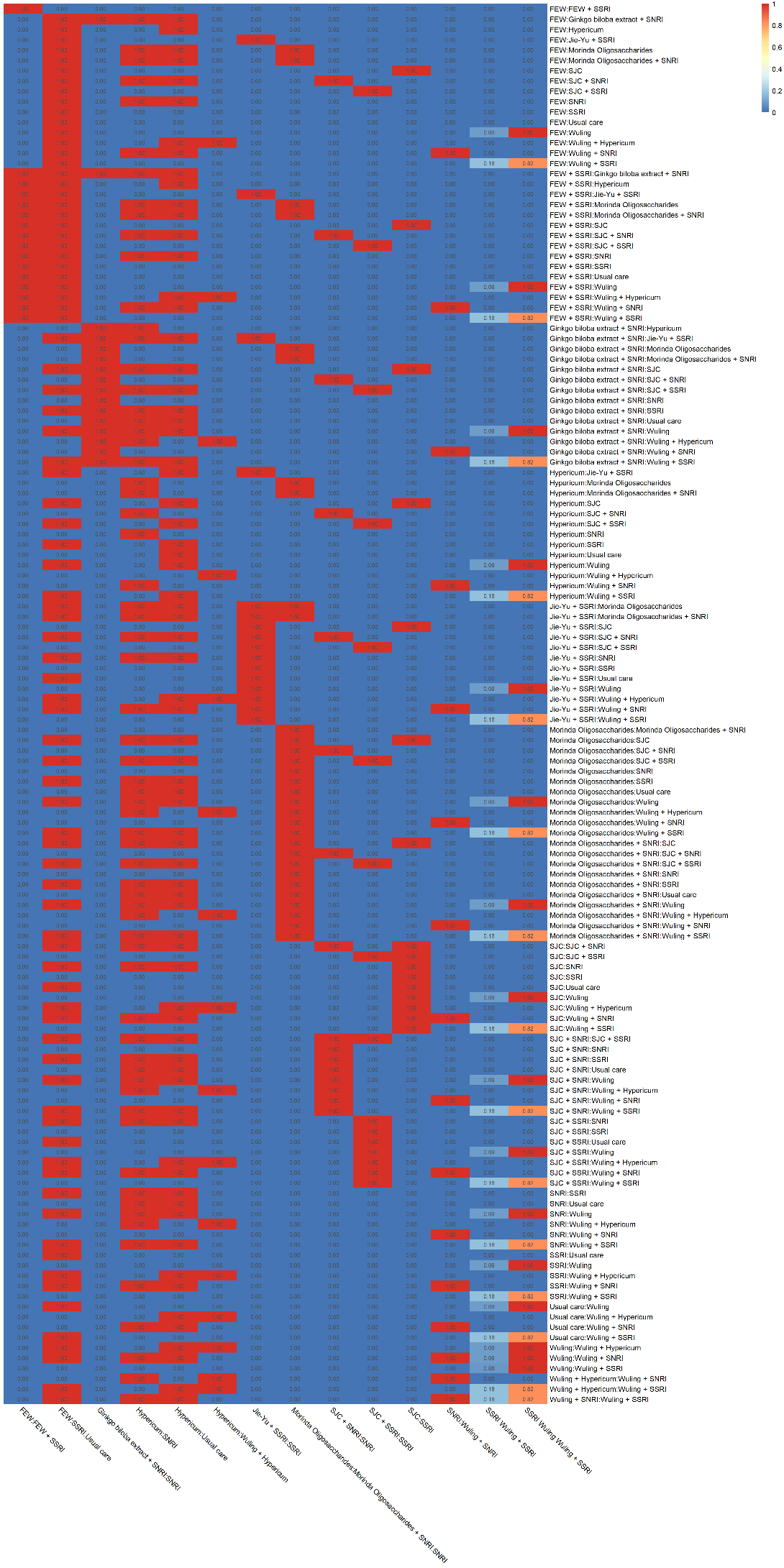


**Outcome: Any nervous system event**


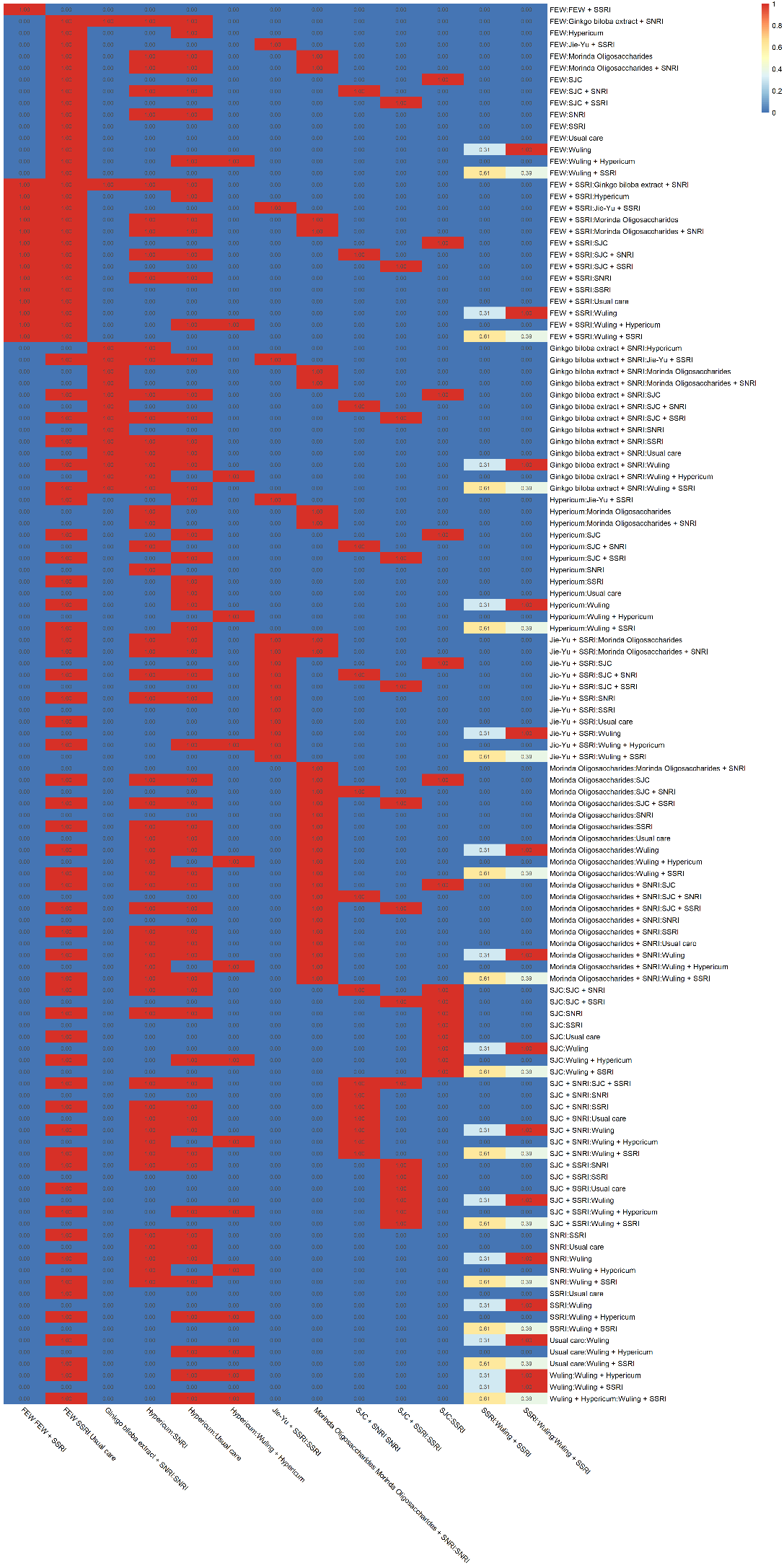


## 8.3 Percentage contribution matrices

Notes: The study calculated the percentage contribution matrix of each direct comparison to each network comparison using a random walk approach. A random walk on a graph is a stochastic process that describes a succession of random walk between vertices which are connected by an edge. The weight of an edge relates to the probability that the walker moves along that edge. The approach uses the graph representation of the network meta-analysis to construct the transition matrix for a random walk on the network structure. The approach shows that the net number of times a walker crosses each edge of the network is related to the evidence flow network. See details in the paper by Davies and colleagues.

Reference: Davies, A. L., Papakonstantinou, T., Nikolakopoulou, A., Rücker, G., & Galla, T. (2022). Network meta‐analysis and random walks. Statistics in Medicine, 41(12), 2091-2114.

**Outcome: Response rate**


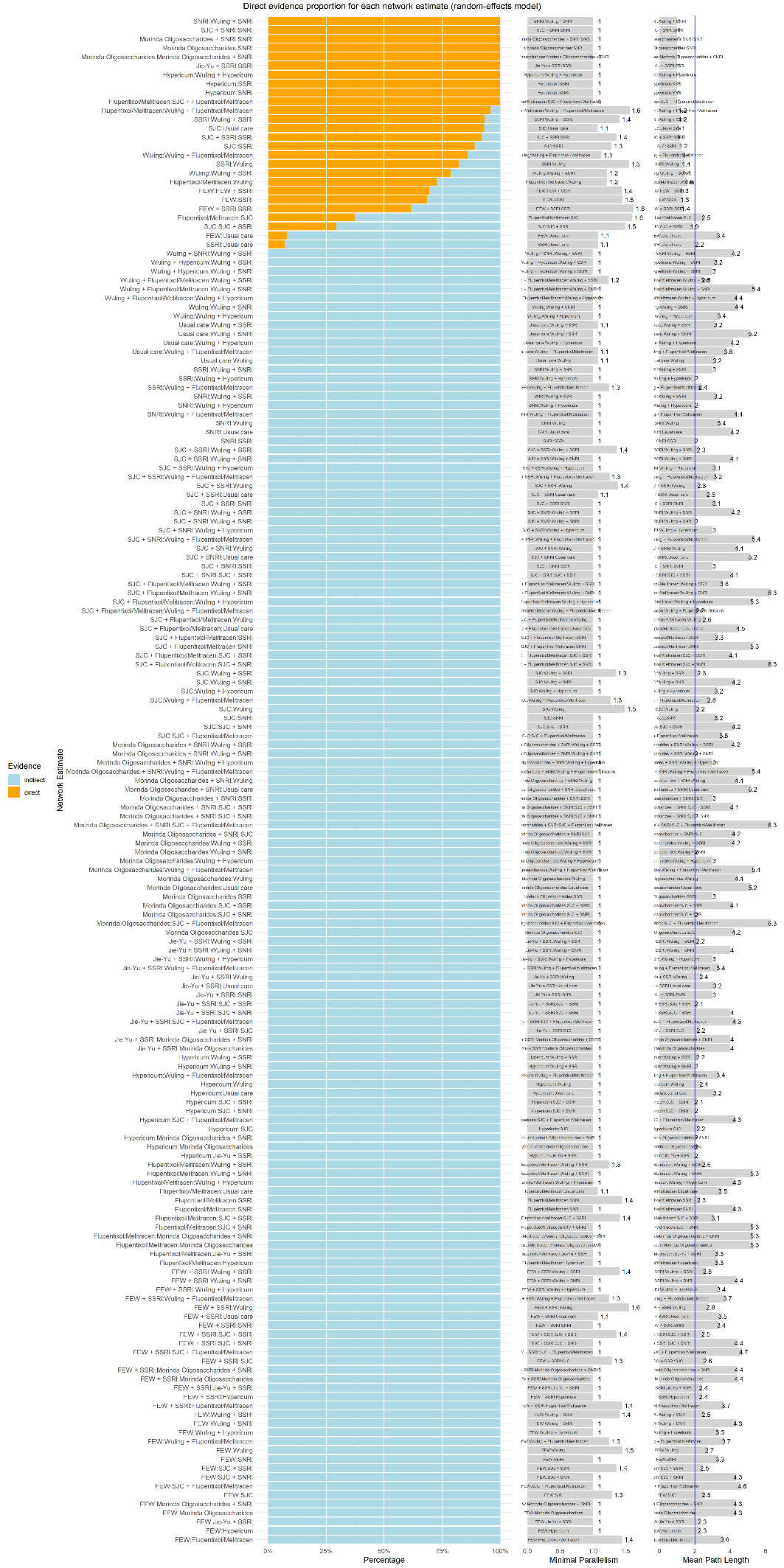


**Outcome: Mean changes in HAMD score from baseline**


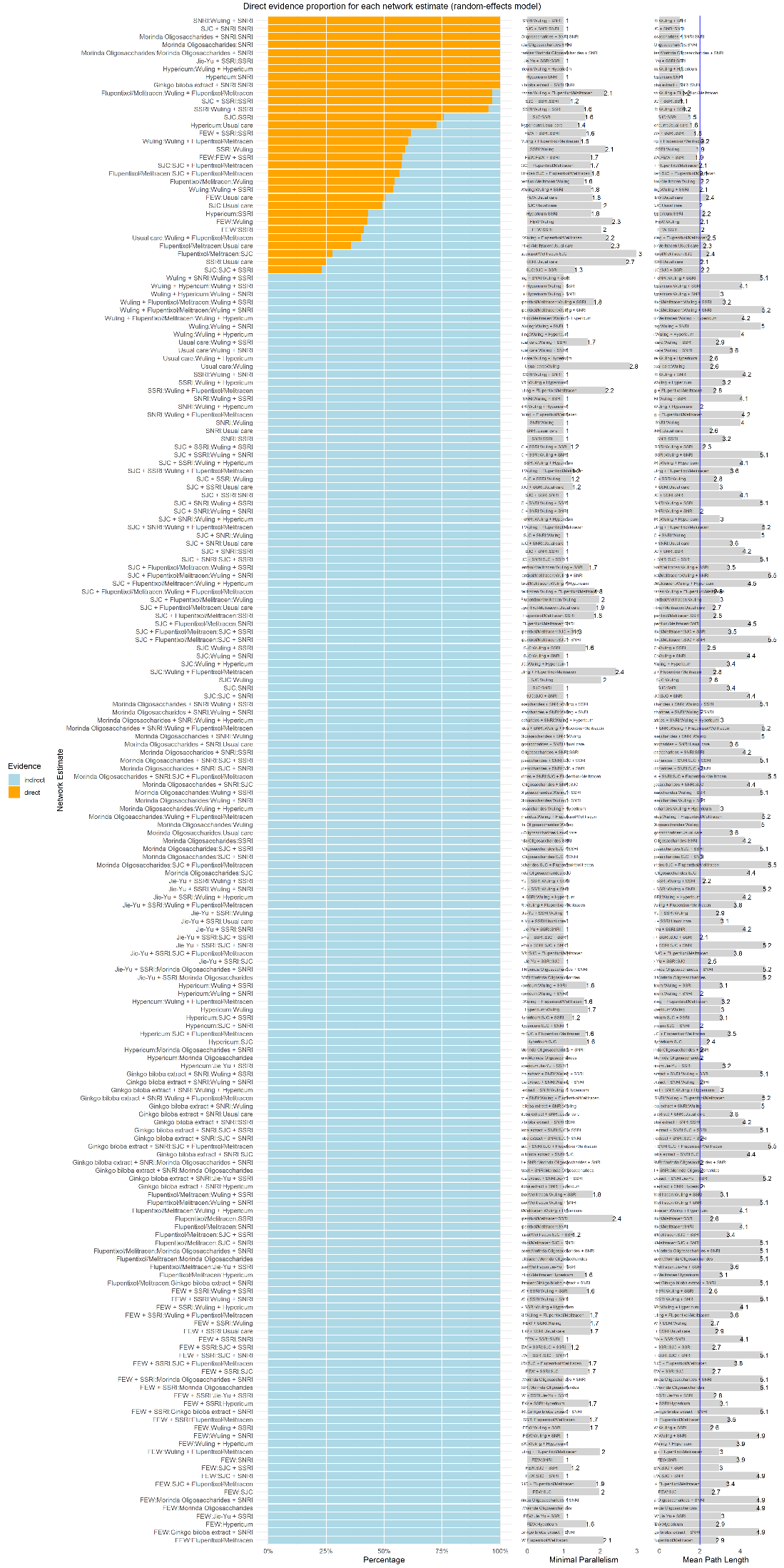


**Outcome:** **All-cause drop out**


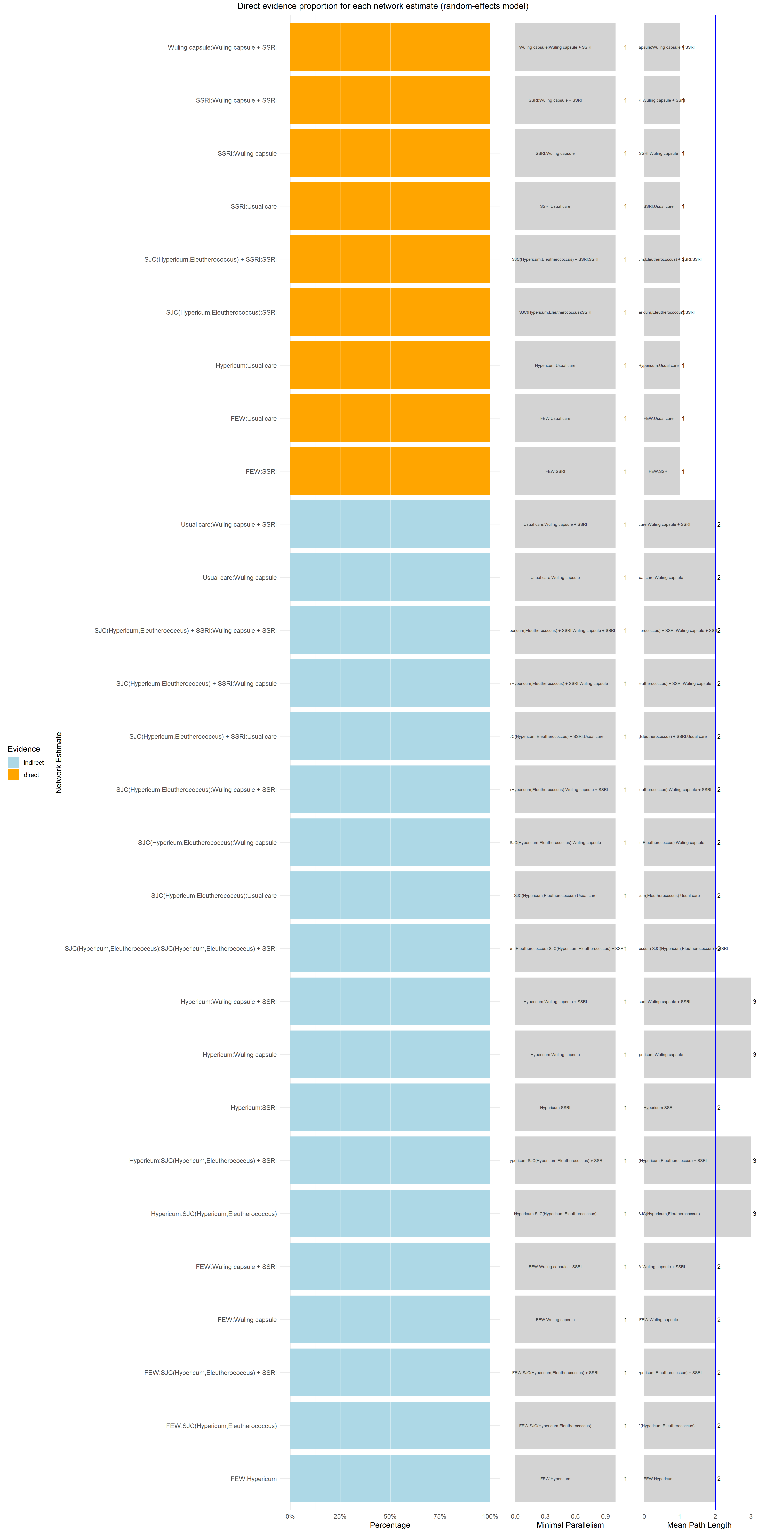


**Outcome:** **Any gastrointestinal event**


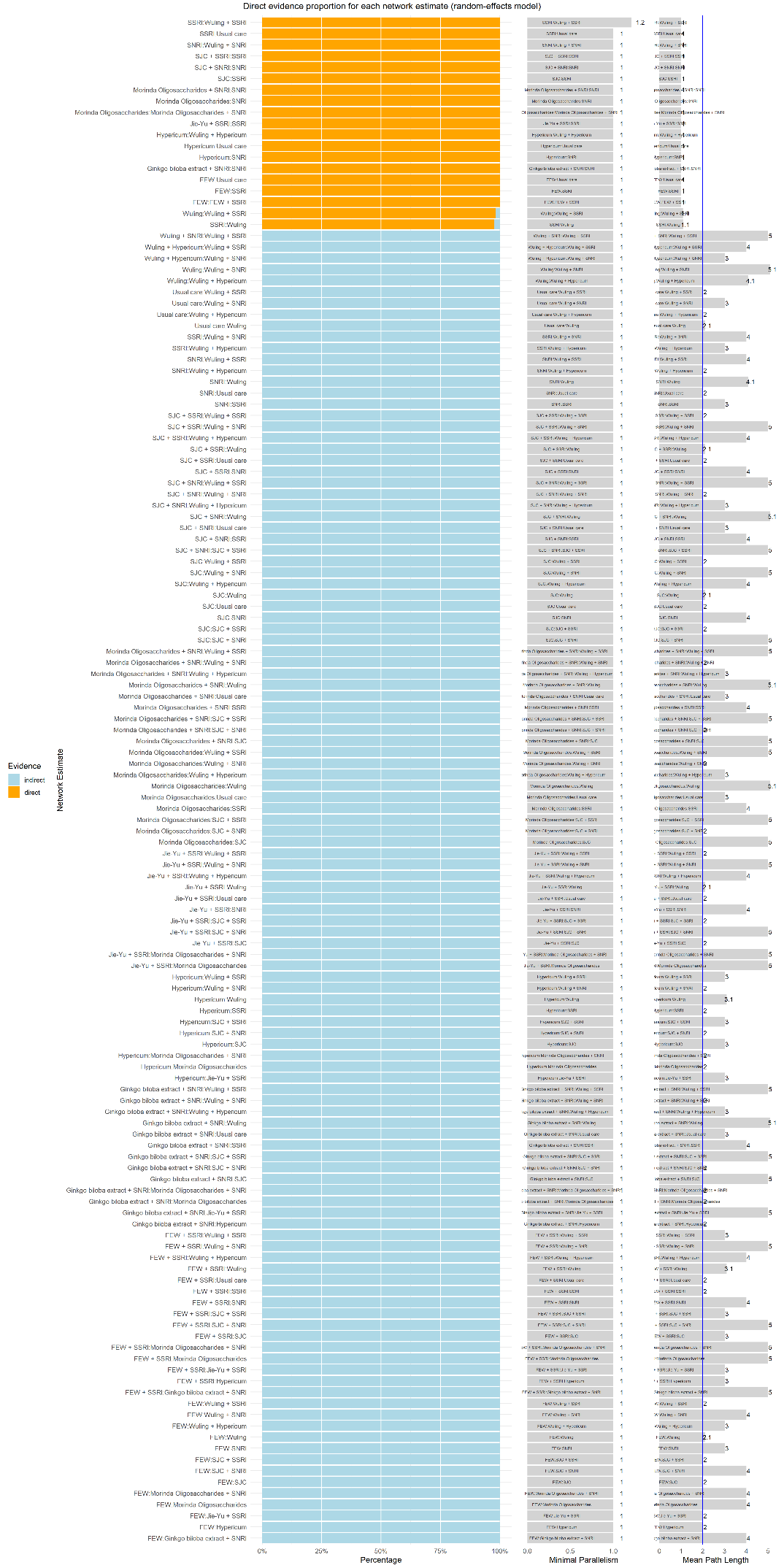


**Outcome:** **Any nervous system event**


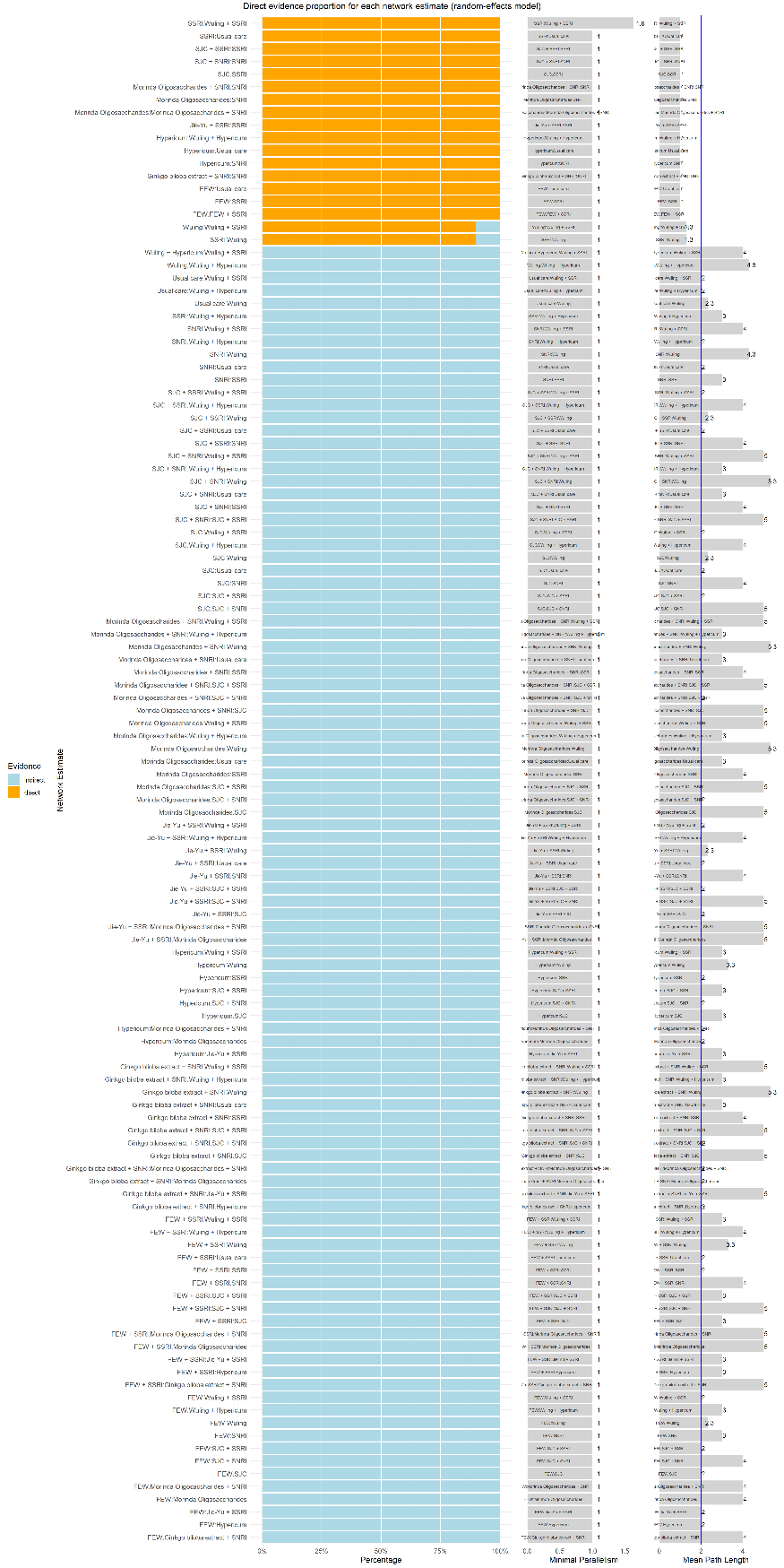


# Appendix 9: Results of regression analyses

**9.1 Length of follow-up**

| Response rate | |
| --- | --- |
| Treatments | **Coefficients** |
| FEW | -0.19  (-9.06, 8.51) |
| FEW + SSRI | -0.18  (-4.62, 4.20) |
| Flupentixol/Melitracen | 0.26  (-0.46, 0.97) |
| Hypericum | -0.10  (-5.72, 5.49) |
| Jie-Yu + SSRI | -0.07  (-0.39, 0.23) |
| Morinda Oligosaccharides | -0.13  (-18.51, 18.64) |
| Morinda Oligosaccharides + SNRI | 0.12  (-18.36, 18.68) |
| SJC | -0.16  (-0.39, 0.07) |
| SJC + Flupentixol/Melitracen | 0.01  (-5.28, 5.27) |
| SJC + SNRI | -0.37  (-13.36, 12.50) |
| SJC + SSRI | 0.15  (-0.34, 0.64) |
| SNRI | -0.05  (-7.74, 7.47) |
| Usual care | -5.09  (-12.81, -1.90) |
| Wuling | 0.12  (-0.08, 0.32) |
| Wuling + Flupentixol/Melitracen | -0.21  (-1.06, 0.61) |
| Wuling + Hypericum | 0.32  (-18.38, 19.22) |
| Wuling + SNRI | -0.22  (-13.07, 12.57) |
| Wuling + SSRI | -0.03  (-0.25, 0.19) |

| Mean changes in HAMD score from baseline | |
| --- | --- |
| Treatments | **Coefficients** |
| FEW | -0.26  (-9.75, 8.95) |
| FEW + SSRI | 0.84  (-3.89, 0.56) |
| Flupentixol/Melitracen | -0.84  (-3.3, 1.62) |
| Ginkgo biloba extract + SNRI | -1.21  (-18.17, 15.99) |
| Hypericum | 0.31  (-2.62, 3.21) |
| Jie-Yu + SSRI | -0.50  (-2.16, 1.14) |
| Morinda Oligosaccharides | 3.54  (-13.75, 20.71) |
| Morinda Oligosaccharides + SNRI | -1.72  (-19.00, 15.16) |
| SJC | 0.10  (-1.05, 1.24) |
| SJC + Flupentixol/Melitracen | -0.87  (-5.60, 3.91) |
| SJC + SNRI | 2.15  (-11.35, 15.79) |
| SJC + SSRI | 0.81  (-1.65, 3.19) |
| SNRI | -0.12  (-4.41, 4.24) |
| Usual care | -0.97  (-3.32, 1.38) |
| Wuling | -0.32  (-1.32, 0.68) |
| Wuling + Flupentixol/Melitracen | -1.24  (-3.70, 1.22) |
| Wuling + Hypericum | -3.75  (-20.94, 13.19) |
| Wuling + SNRI | 0.55  (-12.93, 14.20) |
| Wuling + SSRI | -0.77  (-1.70, 0.16) |

# Appendix 10: Results of subgroup analyses

**10.1 Severity of depression (Subgroups: Mile to moderate, Severe)**

| Outcome: Response rate | |
| --- | --- |
| Treatments | **Coefficients** |
| FEW | 0.02  (-19.11, 19.39) |
| FEW + SSRI | 0.03  (-19.79, 19.70) |
| Flupentixol/Melitracen | 1.05  (-10.31, 12.19) |
| Hypericum | 0.29  (-10.79, 11.60) |
| Jie-Yu + SSRI | -0.09  (-19.9, 19.61) |
| Morinda Oligosaccharides | -0.07  (-13.71, 13.75) |
| Morinda Oligosaccharides + SNRI | 0.27  (-13.37, 14.28) |
| SJC | 0.27  (-0.94, 1.51) |
| SJC + Flupentixol/Melitracen | 0.29  (-14.81, 15.03) |
| SJC + SNRI | 0.46  (-8.52, 9.37) |
| SJC + SSRI | -0.06  (-1.32, 1.18) |
| SNRI | 0.22  (-8.73, 9.09) |
| Usual care | 0.02  (-19.56, 19.38) |
| Wuling | -1.13  (-12.49, 9.91) |
| Wuling + Flupentixol/Melitracen | 0.28  (-14.48, 15.04) |
| Wuling + Hypericum | 0.02  (-19.81, 19.37) |
| Wuling + SNRI | -0.03  (-19.62, 19.61) |
| Wuling + SSRI | 0.02  (-19.26, 19.24) |

| Outcome: Mean changes in HAMD score from baseline | |
| --- | --- |
| Treatments | **Coefficients** |
| FEW | 0.12  (-19.56, 19.67) |
| FEW + SSRI | 0.17  (-19.83, 20.29) |
| Flupentixol/Melitracen | 1.74  (-8.65, 11.92) |
| Ginkgo biloba extract + SNRI | -0.08  (-19.78, 19.56) |
| Hypericum | 1.25  (-6.43, 8.70) |
| Jie-Yu + SSRI | 0.09  (-19.58, 19.68) |
| Morinda Oligosaccharides | 2.97  (-11.61, 17.34) |
| Morinda Oligosaccharides + SNRI | -2.96  (-17.54, 11.44) |
| SJC | -0.11  (-4.98, 4.62) |
| SJC + Flupentixol/Melitracen | 0.86  (-11.06, 12.55) |
| SJC + SNRI | -3.43  (-14.32, 7.50) |
| SJC + SSRI | 1.92  (-2.80, 6.46) |
| SNRI | -2.36  (-11.96, 7.22) |
| Usual care | -2.29  (-10.49, 5.86) |
| Wuling | -1.24  (--12.15, 9.54) |
| Wuling + Flupentixol/Melitracen | -1.61  (-12.29, 9.01) |
| Wuling + Hypericum | -0.03  (-19.67, 19.54) |
| Wuling + SNRI | -0.09  (-19.92, 19.72) |
| Wuling + SSRI | 0.02  (-20.07, 20.09) |

# Appendix 11: Results of sensitivity analyses

**11.1 Exclusion of studies that were followed up less than eight weeks**

**Outcome: Response rate**

| FEW |  |  |  |  |  |  |  |  |  |  |
| --- | --- | --- | --- | --- | --- | --- | --- | --- | --- | --- |
| 0.93  (0.67,1.29) | FEW + SSRI |  |  |  |  |  |  |  |  |  |
| 0.78  (0.46,1.31) | 0.84  (0.45,1.55) | Flupentixol/Melitracen |  |  |  |  |  |  |  |  |
| 0.69  (0.47,1.01) | 0.74  (0.45,1.23) | 0.88  (0.56,1.38) | Jie Yu Pill + SSRI |  |  |  |  |  |  |  |
| 0.94  (0.63,1.39) | 1.01  (0.60,1.69) | 1.20  (0.76,1.89) | 1.36  (1.01,1.83) | SJC |  |  |  |  |  |  |
| 0.66  (0.45,0.96) | 0.71  (0.43,1.17) | 0.84  (0.54,1.31) | 0.96  (0.73,1.25) | 0.70  (0.55,0.90) | SJC + SSRI |  |  |  |  |  |
| 0.92  (0.66,1.29) | 1.00  (0.62,1.59) | 1.18  (0.79,1.77) | 1.35  (1.10,1.65) | 0.99  (0.80,1.22) | 1.41  (1.18,1.68) | SSRI |  |  |  |  |
| 36.80 (2.32,583.17) | 39.63 (2.45,640.22) | 47.16 (2.90,768.12) | 53.57 (3.36,853.91) | 39.30 (2.46,627.1) | 56.00 (3.52,891.09) | 39.83 (2.52,630.19) | Usual care |  |  |  |
| 0.78  (0.52,1.18) | 0.84  (0.50,1.42) | 1.00  (0.73,1.37) | 1.14  (0.83,1.56) | 0.83  (0.60,1.15) | 1.19  (0.88,1.60) | 0.84  (0.66,1.08) | 0.02  (0.00,0.34) | Wuling capsule |  |  |
| 0.68  (0.41,1.13) | 0.74  (0.40,1.34) | 0.87  (0.65,1.18) | 0.99  (0.64,1.53) | 0.73  (0.47,1.13) | 1.04  (0.68,1.58) | 0.74  (0.50,1.08) | 0.02  (0.00,0.30) | 0.87  (0.65,1.18) | Wuling capsule + Flupentixol/Melitracen |  |
| 0.68  (0.45,1.01) | 0.73  (0.44,1.22) | 0.87  (0.57,1.31) | 0.99  (0.73,1.33) | 0.72  (0.53,0.99) | 1.03  (0.78,1.37) | 0.73  (0.59,0.92) | 0.02  (0.00,0.29) | 0.87  (0.67,1.13) | 0.99  (0.67,1.47) | Wuling capsule + SSRI |


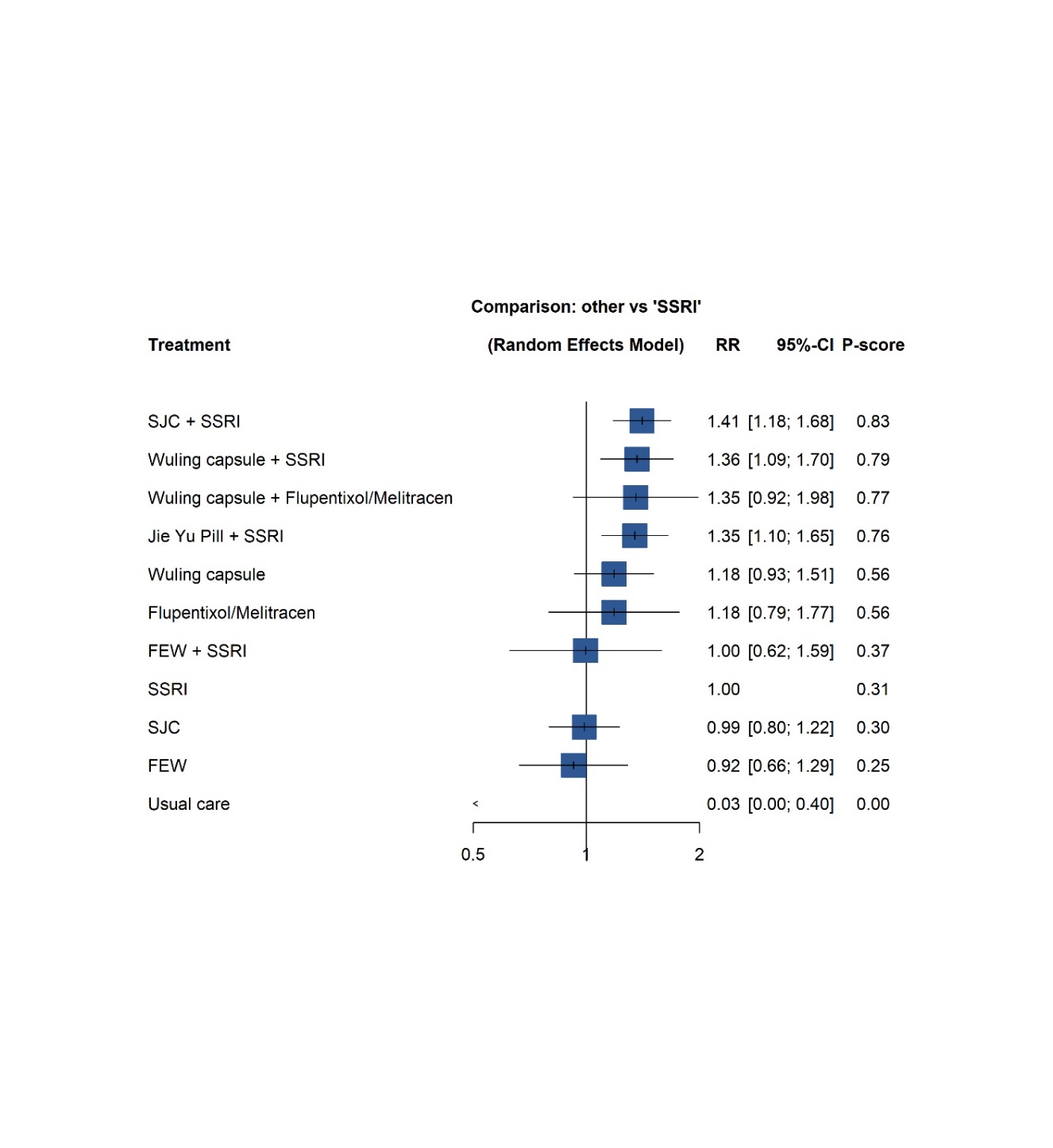


**Outcome: Mean changes in HAMD score from baseline**

| FEW |  |  |  |  |  |  |  |  |  |  |  |  |  |  |  |  |
| --- | --- | --- | --- | --- | --- | --- | --- | --- | --- | --- | --- | --- | --- | --- | --- | --- |
| -4.98 (-12.10, 2.14) | FEW + SSRI |  |  |  |  |  |  |  |  |  |  |  |  |  |  |  |
| -2.21 (-10.56, 6.14) | 2.77 ( -8.21,13.75) | Flupentixol/Melitracen |  |  |  |  |  |  |  |  |  |  |  |  |  |  |
| 2.97 (-10.77,16.71) | 7.95 ( -7.52,23.42) | 5.18 (-10.28,20.64) | Ginkgo biloba extract + SNRI |  |  |  |  |  |  |  |  |  |  |  |  |  |
| -2.04 (-11.65, 7.57) | 2.94 ( -9.02,14.90) | 0.17 (-11.77,12.11) | -5.01 (-14.83, 4.81) | Hypericum |  |  |  |  |  |  |  |  |  |  |  |  |
| 4.34 ( -1.84,10.52) | 9.32 ( -0.11,18.74) | 6.55 ( -1.77,14.87) | 1.37 (-12.72,15.46) | 6.38 ( -3.72,16.48) | Jie Yu Pill + SSRI |  |  |  |  |  |  |  |  |  |  |  |
| -7.98 (-21.77, 5.81) | -3.00 (-18.52,12.52) | -5.77 (-21.28, 9.74) | -10.95 (-20.93, -0.97) | -5.94 (-15.84, 3.96) | -12.32 (-26.46, 1.82) | Morinda Oligosaccharides |  |  |  |  |  |  |  |  |  |  |
| 4.28 ( -9.52,18.08) | 9.26 ( -6.27,24.79) | 6.49 ( -9.02,22.01) | 1.31 ( -8.67,11.29) | 6.32 ( -3.58,16.22) | -0.06 (-14.20,14.09) | 12.26 (5.16,19.36) | Morinda Oligosaccharides + SNRI |  |  |  |  |  |  |  |  |  |
| -0.97 ( -7.27, 5.32) | 4.01 ( -5.50,13.51) | 1.24 ( -7.17, 9.65) | -3.94 (-18.09,10.20) | 1.07 ( -9.11,11.24) | -5.31 ( -9.90, -0.72) | 7.01 ( -7.19,21.20) | -5.25 (-19.45, 8.94) | SJC |  |  |  |  |  |  |  |  |
| 2.75 ( -3.26, 8.76) | 7.73 ( -1.59,17.05) | 4.96 ( -3.24,13.15) | -0.22 (-14.24,13.79) | 4.79 ( -5.21,14.79) | -1.59 ( -5.78, 2.60) | 10.73 ( -3.34,24.80) | -1.53 (-15.60,12.54) | 3.72 ( -0.36, 7.80) | SJC + SSRI |  |  |  |  |  |  |  |
| -0.86 (-12.68,10.96) | 4.12 ( -9.68,17.92) | 1.35 (-12.44,15.14) | -3.83 (-10.83, 3.17) | 1.18 ( -5.71, 8.07) | -5.20 (-17.42, 7.03) | 7.12 (0.01,14.23) | -5.14 (-12.26, 1.98) | 0.11 (-12.17,12.40) | -3.61 (-15.75, 8.53) | SNRI |  |  |  |  |  |  |
| -0.88 ( -6.20, 4.45) | 4.10 ( -4.79,13.00) | 1.33 ( -6.37, 9.04) | -3.85 (-17.58, 9.89) | 1.16 ( -8.44,10.77) | -5.21 ( -8.34, -2.08) | 7.10 ( -6.69,20.89) | -5.16 (-18.95, 8.64) | 0.10 ( -3.26, 3.46) | -3.63 ( -6.41, -0.84) | -0.02 (-11.83,11.80) | SSRI |  |  |  |  |  |
| -5.64 (-12.14, 0.86) | -0.66 (-10.30, 8.98) | -3.43 (-13.05, 6.19) | -8.61 (-20.72, 3.50) | -3.60 (-10.68, 3.48) | -9.98 (-17.18, -2.77) | 2.34 ( -9.83,14.51) | -9.92 (-22.09, 2.25) | -4.67 (-11.98, 2.64) | -8.39 (-15.45, -1.33) | -4.78 (-14.65, 5.09) | -4.76 (-11.26, 1.73) | Usual care |  |  |  |  |
| -3.91 ( -9.27, 1.45) | 1.07 ( -7.84, 9.98) | -1.70 ( -8.11, 4.70) | -6.88 (-20.96, 7.19) | -1.87 (-11.95, 8.21) | -8.25 (-13.56, -2.94) | 4.07 (-10.06,18.20) | -8.19 (-22.32, 5.94) | -2.94 ( -8.39, 2.51) | -6.66 (-11.77, -1.55) | -3.05 (-15.26, 9.16) | -3.04 ( -7.32, 1.25) | 1.73 ( -5.45, 8.91) | Wuling capsule |  |  |  |
| 2.09 ( -6.27,10.45) | 7.07 ( -3.91,18.05) | 4.30 (0.30, 8.30) | -0.88 (-16.35,14.58) | 4.13 ( -7.82,16.07) | -2.25 (-10.57, 6.07) | 10.07 ( -5.44,25.58) | -2.19 (-17.71,13.33) | 3.06 ( -5.35,11.48) | -0.66 ( -8.86, 7.54) | 2.95 (-10.84,16.74) | 2.97 ( -4.75,10.68) | 7.73 ( -1.90,17.35) | 6.00 ( -0.41,12.41) | Wuling capsule + Flupentixol/Melitracen |  |  |
| 7.24 ( -4.58,19.06) | 12.22 ( -1.58,26.02) | 9.45 ( -4.34,23.24) | 4.27 ( -7.73,16.27) | 9.28 (2.39,16.17) | 2.90 ( -9.32,15.13) | 15.22 (3.16,27.28) | 2.96 ( -9.11,15.03) | 8.21 ( -4.07,20.50) | 4.49 ( -7.65,16.63) | 8.10 ( -1.64,17.84) | 8.12 ( -3.70,19.94) | 12.88 (3.00,22.76) | 11.15 ( -1.06,23.36) | 5.15 ( -8.64,18.94) | Wuling capsule + Hypericum |  |
| 4.78 ( -1.99,11.55) | 9.76 ( -0.06,19.59) | 6.99 ( -1.47,15.46) | 1.81 (-12.63,16.26) | 6.82 ( -3.77,17.41) | 0.45 ( -5.23, 6.12) | 12.76 ( -1.73,27.26) | 0.50 (-14.00,15.00) | 5.76 ( -0.05,11.56) | 2.03 ( -3.46, 7.52) | 5.64 ( -6.99,18.27) | 5.66 (0.92,10.39) | 10.42 (2.54,18.30) | 8.69 (3.16,14.23) | 2.69 ( -5.77,11.16) | -2.46 (-15.09,10.18) | Wuling capsule + SSRI |


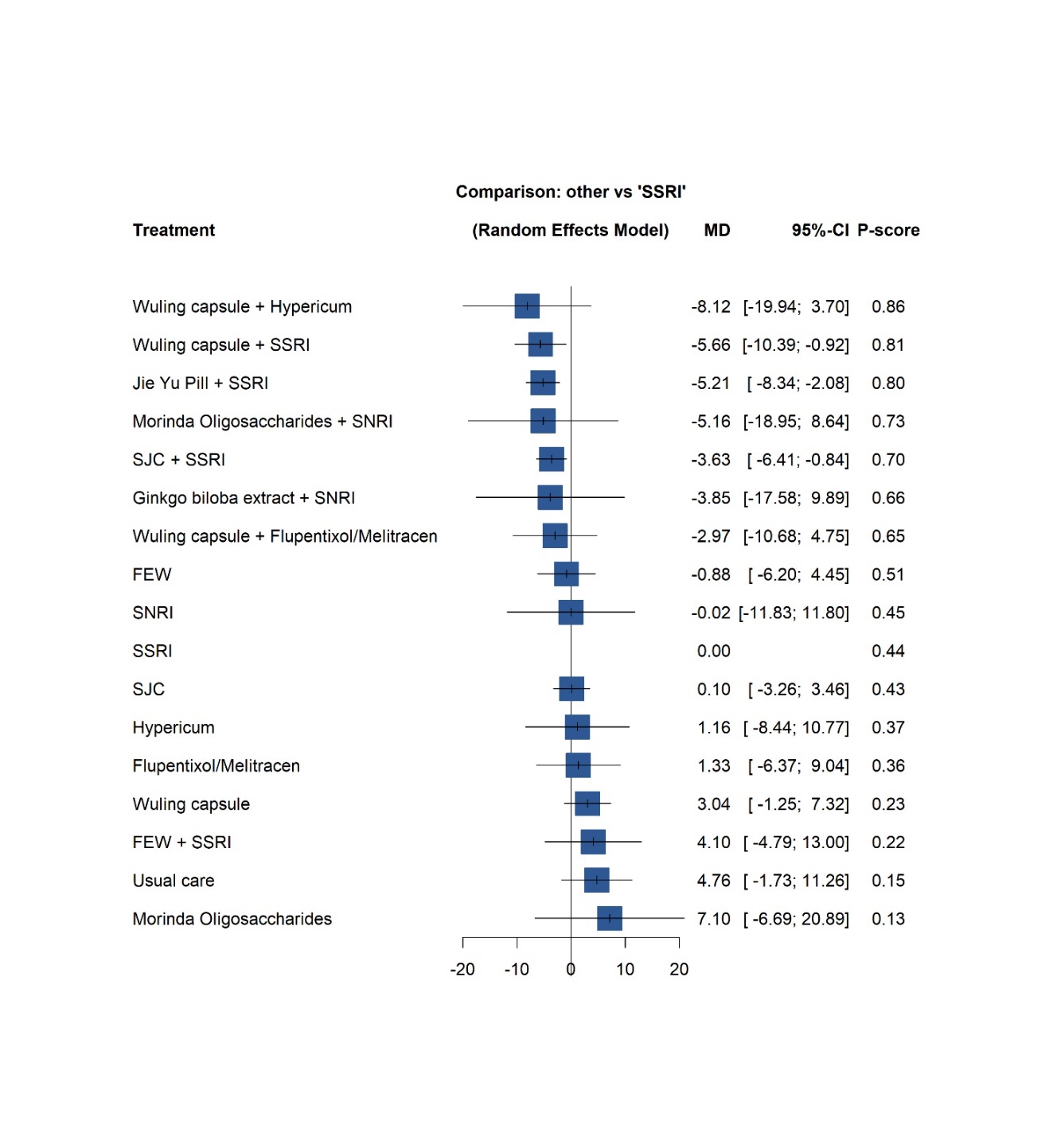


**Outcome: All-cause drop out**

| FEW |  |  |  |  |  |  |
| --- | --- | --- | --- | --- | --- | --- |
| 0.04  (0.00,2.81) | Hypericum |  |  |  |  |  |
| 0.11  (0.00,4.84) | 2.94  (0.04,216.04) | SJC + SSRI |  |  |  |  |
| 0.20  (0.01,4.08) | 5.60  (0.15,202.83) | 1.90  (0.18,20.15) | SSRI |  |  |  |
| 0.10  (0.00,2.04) | 2.82  (0.12,66.72) | 0.96  (0.05,17.56) | 0.50  (0.09,2.75) | Usual care |  |  |
| 1.00  (0.01,70.43) | 27.98 (0.26,3014.39) | 9.51  (0.21,433.04) | 5.00  (0.25,100.58) | 9.92  (0.32,311.98) | Wuling capsule |  |
| 0.20  (0.01,6.35) | 5.60  (0.11,296.21) | 1.90  (0.10,34.71) | 1.00  (0.18,5.43) | 1.98  (0.18,21.80) | 0.20  (0.01,4.02) | Wuling capsule + SSRI |


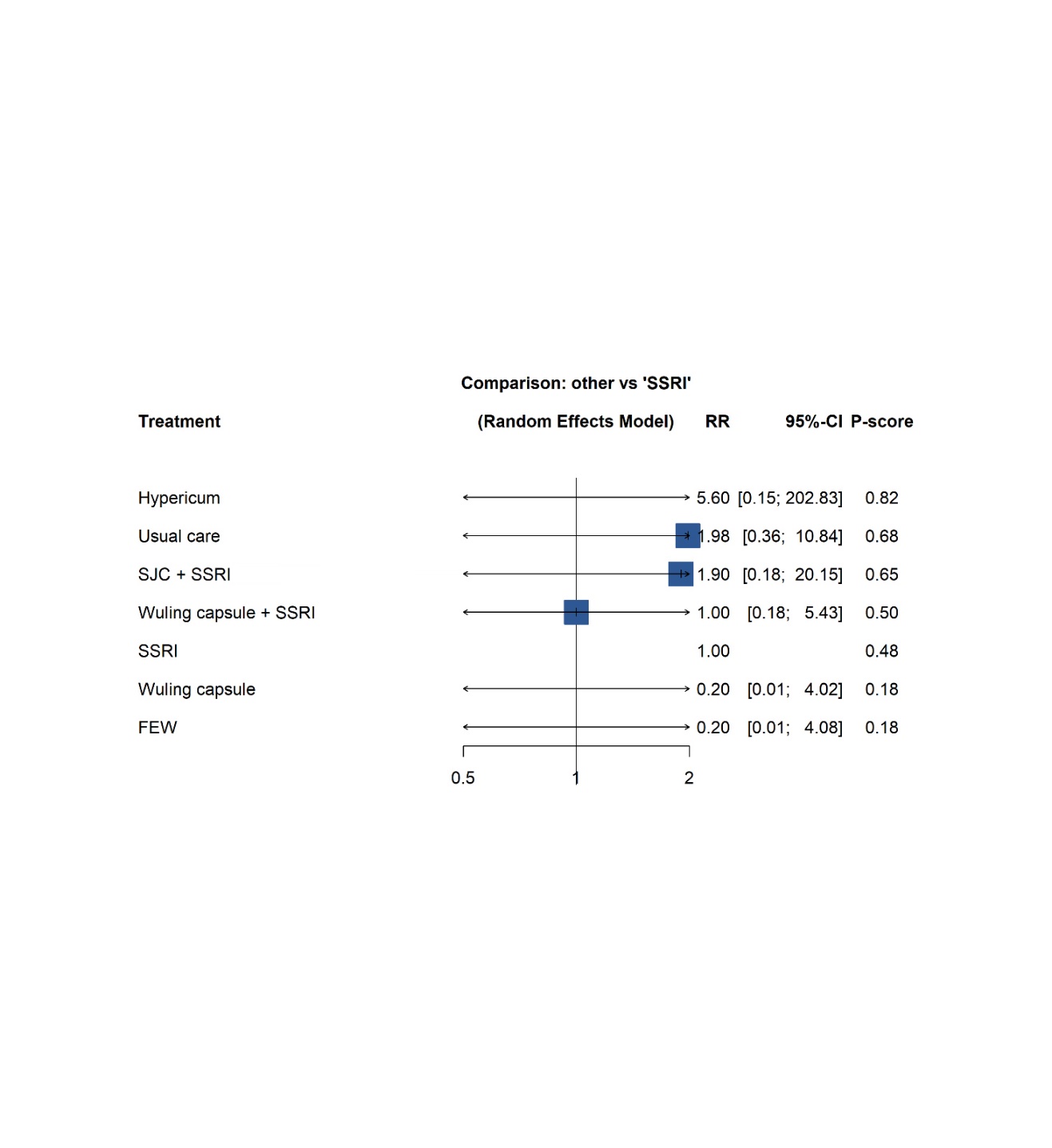


**Outcome: Any gastrointestinal event**

| FEW |  |  |  |  |  |  |  |  |
| --- | --- | --- | --- | --- | --- | --- | --- | --- |
| 1.00  (0.52,1.92) | FEW + SSRI |  |  |  |  |  |  |  |
| 0.40  (0.07,2.27) | 0.40  (0.06,2.56) | Jie Yu Pill + SSRI |  |  |  |  |  |  |
| 0.80  (0.14,4.59) | 0.80  (0.12,5.16) | 1.99  (0.67,5.94) | SJC |  |  |  |  |  |
| 0.39  (0.07,2.03) | 0.39  (0.07,2.30) | 0.97  (0.38,2.46) | 0.49  (0.19,1.27) | SJC + SSRI |  |  |  |  |
| 0.33  (0.07,1.59) | 0.33  (0.06,1.81) | 0.83  (0.39,1.77) | 0.42  (0.19,0.92) | 0.85  (0.50,1.46) | SSRI |  |  |  |
| 0.33  (0.06,1.89) | 0.33  (0.05,2.13) | 0.83  (0.18,3.78) | 0.42  (0.09,1.94) | 0.85  (0.21,3.53) | 1.00  (0.27,3.72) | Usual care |  |  |
| 0.93  (0.14,6.12) | 0.93  (0.13,6.84) | 2.33  (0.64,8.49) | 1.17  (0.31,4.36) | 2.39  (0.73,7.78) | 2.80  (0.98,8.01) | 2.80 (0.52,15.07) | Wuling capsule |  |
| 0.37  (0.07,2.03) | 0.37  (0.06,2.30) | 0.93  (0.34,2.54) | 0.47  (0.17,1.31) | 0.96  (0.41,2.24) | 1.12  (0.58,2.16) | 1.12  (0.26,4.88) | 0.40  (0.14,1.13) | Wuling capsule + SSRI |

**
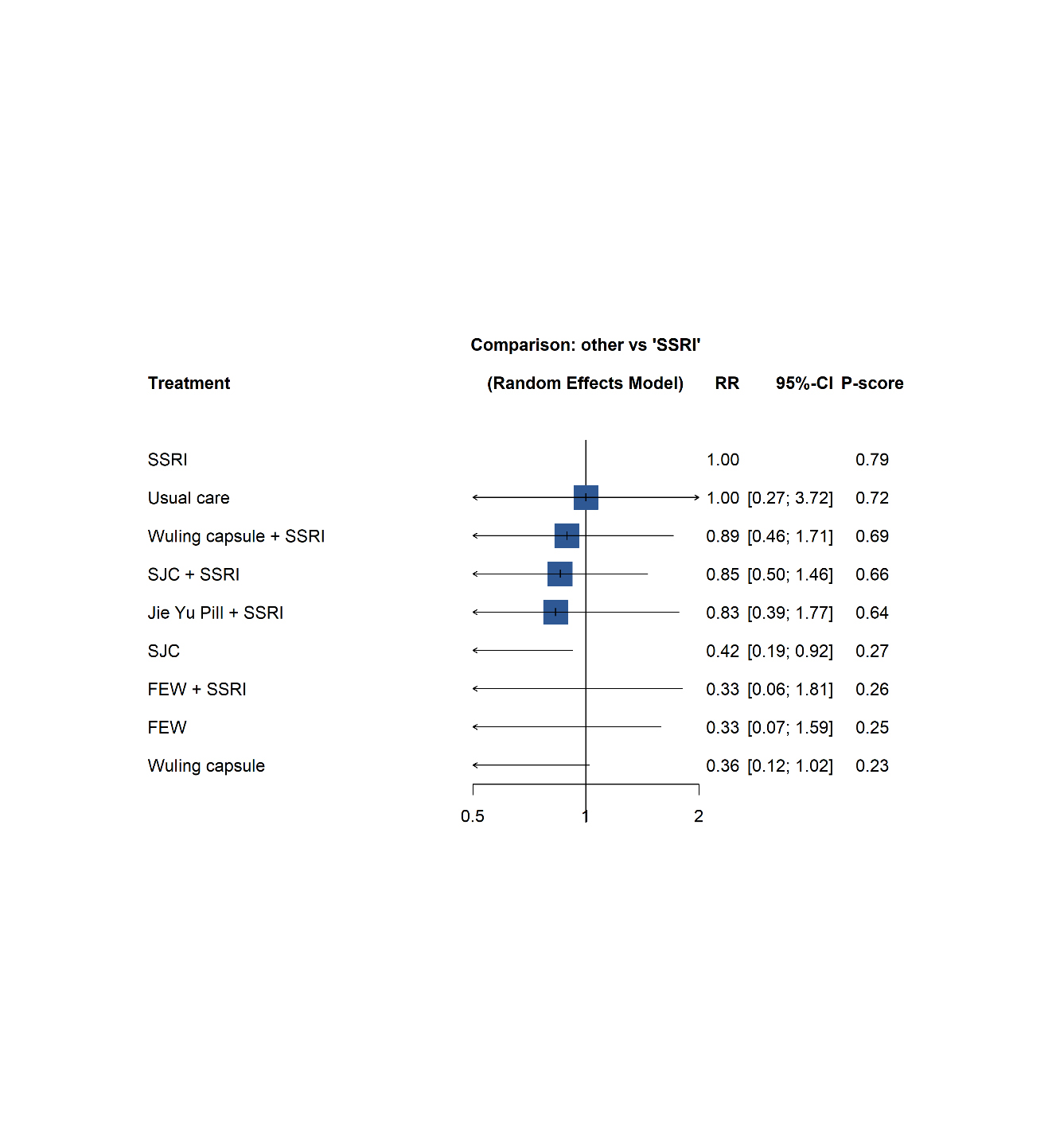
**

**Outcome: Any nervous system event**

| FEW |  |  |  |  |  |  |  |  |
| --- | --- | --- | --- | --- | --- | --- | --- | --- |
| 1.00 (0.22,4.63) | FEW + SSRI |  |  |  |  |  |  |  |
| 0.09 (0.00,2.23) | 0.09  (0.00,3.17) | Jie Yu Pill + SSRI |  |  |  |  |  |  |
| 0.92  (0.03,26.17) | 0.92  (0.02,36.54) | 9.69  (1.20, 78.06) | SJC |  |  |  |  |  |
| 0.06  (0.00,1.67) | 0.06  (0.00,2.34) | 0.66  (0.09,4.74) | 0.07  (0.01,0.66) | SJC + SSRI |  |  |  |  |
| 0.11  (0.01,2.02) | 0.11  (0.00,2.95) | 1.17  (0.34,4.07) | 0.12  (0.02,0.64) | 1.76  (0.39,8.06) | SSRI |  |  |  |
| 0.10  (0.00,2.04) | 0.10  (0.00,2.94) | 1.06  (0.15,7.37) | 0.11  (0.01,1.02) | 1.60  (0.19, 13.37) | 0.91  (0.21,4.00) | Usual care |  |  |
| 0.33  (0.01,12.68) | 0.33  (0.01,17.24) | 3.43  (0.27,44.35) | 0.35  (0.02,5.77) | 5.17  (0.35, 77.09) | 2.93  (0.31,27.40) | 3.23  (0.22,47.24) | Wuling capsule |  |
| 0.24  (0.01,5.48) | 0.24  (0.01,7.82) | 2.51  (0.45,14.08) | 0.26  (0.03,2.02) | 3.79  (0.55, 26.07) | 2.15  (0.65,7.05) | 2.37  (0.35,15.85) | 0.73  (0.08,6.85) | Wuling capsule + SSRI |

**
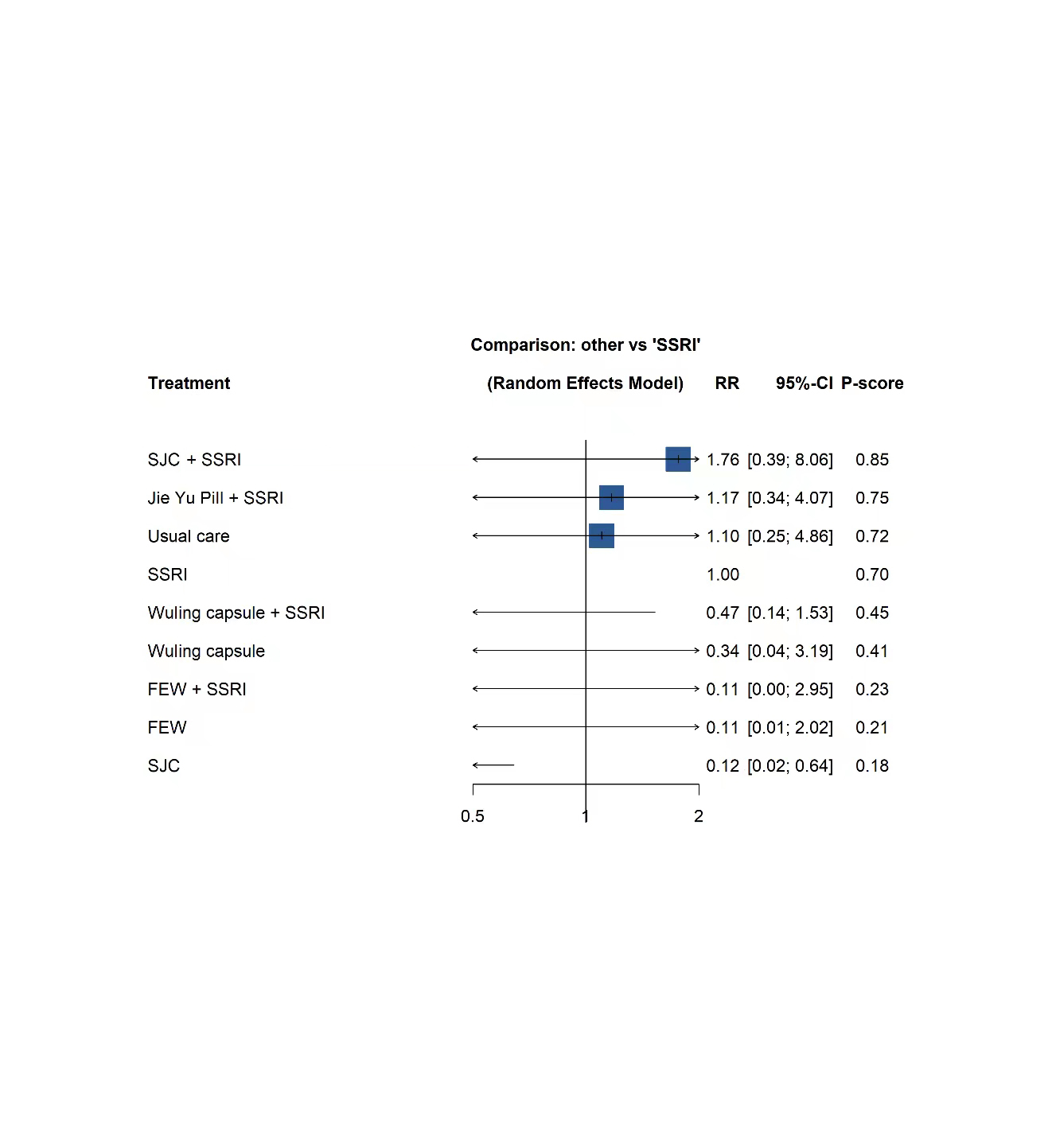
**

# Appendix 12: Reference list of included studies

1. **An, L 2019**

- **An Liang. Clinical observation on Shugan Jieyu Capsule on depression after cerebral infarction. CJGMCM. 2019, August;34(15):2289-2291.**

1. **Chai, D 2015**

- **Chai D H, Du J, Zhao X T. Wuling capture combined with the treatment of senile post-stroke depression for 36 cases. Chinese medicine modern distance education of China.2015, July;13(13):49-50.**

1. **Chen, D 2007**

- **Chen D, Liu Y, Dou W B.** **Effects of neurostan on post- stroke depression and its nervous function defect.** **China Journal of Modern Medicine.2007, September;17(18):2276-2278.**

1. **Chen, J 2015**

- **Chen J, Ruan S Q.** **Efficacy and safety of Wuling capsule on patients with post-stroke depression.** **Chinese Journal of Biochemical Pharmaceutics.2015;35(10):29-31.**

1. **Chen, JB 2015**

- **Chen J B.** **Comparative analysis of Shugan Jieyu capsule and paroxetine in the treatment of post-stroke depression.** **Research of Integrated Traditional Chinese and Western Medicine.2015, June;7(3):138-139,141.**

1. **Chen, L 2015**

- **Chen L, Man X R.** **Clinical efficacy and safety analysis of Wuling capsule combined with Deanxit in the treatment of post-stroke depression.** **Journal of Clinical Medical.2015, November;2(32):6625-6626.**

1. **Deng, X 2016**

- **Deng X L.** **Effects of Shugan jieyu capsule on post-stroke depressive symptoms and recovery.** **Journal of Basic Chinese Medicine 2016, May;22(5):660-662.**

1. **Fang, J 2015**

- **Fang J, Li X H, Chen W W.** **Effect of Shugan Jieyu capsules on serum brain-derived neurotrophic factor and 5-HT levels with post stroke depression patients.** **WORLD CLINICAL DRUGS.2015;36(5):331-334.**

1. **Fu, J 2008**

- **Fu J L, Zhao Y W, Sun X J.** **Efficacy and safety of Deanxit combined with Wuling Capsule in treating post-stroke depression：A randomized controlled trial.** **Journal of Chinese Integrative Medicine.2008, March;6(3):258-261.**

1. **Gao, G 2016**

- **Gao G L.** **Shugan Jieyu Capsule combined with short-term Deanxit in treatment of post - stroke depression in elderly patients.** **J Medical Forum.2016 October;37(10):155-156.**

1. **Guo, H 2016**

- **Guo H.** **Clinical effect of Shugan Jieyu capsule on post-stroke depression.** **Chin J of Clinical Rational Drug Use.2016, March;9(3c):127-128.**

1. **Hou, J 2015**

- **Hou J H.** **Effect of paroxetine combined with Shugan Jieyu capsule on post-stroke depression.** **JOURNAL OF NEW CHINESE MEDICINE.2015, November;47(11):38-39.**

1. **Huang, R 2016**

- **Huang R E, Zhang X C.** **Effect of Wuling Capsule combined with Deanxit on neurological function and daily living activities in patients with post-stroke depression.2016, April;44(2):80-82.**

1. **Jia, K 2013**

- **Jia K.** **Clinical observation of citalopram hydrobromide combined with Shugan Jieyu capsule in the treatment of post-stroke depression. Medical Journal of Chinese People's Health .2017, June;29(12):34-35.**

1. **Jia, K 2017**

- **Jia K, Zhang Z X.** **Jieyu pill combined with paroxetine hydrochloride tablets in the treatment of 40 cases of post-stroke depression.** **Herald of Medicine.2013,April;32(4)：491-493.**

1. **Lei, X 2016**

- **Lei X W, Zhang X M.** **A control study of Shugan Jieyu capsule plus escitalopram in the treatment of post-stroke depression.** **J Clin Psychosom Dis .2016, May;22(3):21-25.**

1. **Li, F 2019**

- **Li F, Zhang L, Wang L X.** **Clinical study on Wuling Capsules combined with extract of St.** **John’s Wort tablets in the treatment of cerebral infarction with depression at the convalescent stage.** **China Pharmaceuticals.2019, December;28(24):52-55.**

1. **Li, W 2018**

- **Li W, Lai X Z.** **A case control study of Shuganjieyu capsule with sertraline tablets in the treatment of post-stroke depression.** **J Clin Psychosom Dis.2018, July;24(24):110-112.**

1. **Li, X 2014**

- **Li X, Li S Q.** **50 Cases of patiets with depression due to brain stroke treated with paroxetine hydrochloride combined with depression-release pill.** **HENAN TRADITIONAL CHINESE MEDICINE.2014, December;34(12):2317-2319.**

1. **Li, Z 2010**

- **Li Z.** **Treatment of 30 cases of depression after ischemic stroke with modified Danzhi Xiaoyao Powder. HENAN TRADITIONAL CHINESE MEDICINE.2010,April;30(4)：376-377.**

1. **Li, L 2008**

- **Li L T, Wang S H, Ge H Y, Chen J, Yue S W, Y M.** **The beneficial effects of the herbal medicine Free and Easy Wanderer Plus (FEWP) and fluoxetine on post-stroke depression.** **THE JOURNAL OF ALTERNATIVE AND COMPLEMENTARY MEDICINE.2008;14(7):841-846.**

1. **Liang, Z 2019**

- **Liang Z H, Jia Y B, Wang M L, Li Z R, Li M, Yun Y L, Zhu R X.** **Efficacy of ginkgo biloba extract as augmentation of venlafaxine in treating post-stroke depression.** **Neuropsychiatric Disease and Treatment.** **2019; 15:2551-2557.**

1. **Liu, Y 2014**

- **Liu Y, Luo S Y.** **Wuling capsule combined with fluoxetine in the treatment of 41 cases of post-stroke depression.** **SHAANXI JOURNAL OF TRADITIONAL CHINESE MEDICINE.2014;35(6):656-657.**

1. **Luo, C 2014**

- **Luo C, Qiao Z H.** **Clinical observation on 34 cases of post-stroke depression treated with Jieyu Pill and fluoxetine.** **Chinese journal of ethnomedicine and ethnopharmacy.2014;** **23(13):75-76.**

1. **Mao, S 2016**

- **Mao S L, Luo S.** **Efficacy of Shuganjieyu capsule for the treatment of post-stroke depression.** **Clinical Medication Journal.2016 January;14(1):54-56.**

1. **Mo, W 2004**

- **Mo W Y, Yu H Y, Yang Y. Comparison of San John’s Wort extract with fluoxetine in the treatment of post-stroke depression. Chinese Journal of New Drugs.2014;13(10):942-944.**

1. **Na, W 2012**

- **Na W Q, Li J H, Chen K, Xia Q C.** **Comparative observation on efficacy of Shuganjieyu Capsules combined with sertraline in treatment of aged poststroke depression.** **Zhejiang JITCWM.2012;22(4):245-247.**

1. **Shen, G 2019**

- **Shen G Q.** **Clinical efficacy of the Wuling capsule plus western medicine on post-stroke depression.** **Clinical Journal of Chinese Medicine.2019;11(19):88-90.**

1. **Shi, Z 2017**

- **Shi Z Z, Cheng J W, Bai Y.** **The efficacy of Shugan Jieyu capsule combined with fluoxetine in the treatment of post-stroke depression.** **YI YAO QIAN YAN.2017, January;7(2):349-351.**

1. **Su, W 2012**

- **Su W, Na W Q, Li J H, Yang J H, Xia Q C, Guan T F.** **Efficacy comparation of Shuganjieyu Capsule and citalopram on treatment of ischemic poststroke depression in aged patients.2012, March;31(3):322-325.**

1. **Sun, X 2010**

- **Sun X J, Fu S X.** **A control study of St. John’s wort extract vs venlafaxine in poststroke depression.** **J Clin Psychosom Dis，2010, January;16(1):13-14,21.**

1. **Tao, Z 2020**

- **Tao Z, Guo W H, Gao L, Zhao L, Cheng M, Song J G.** **Observation on efficacy of Wuling Capsule combined with venlafaxine and acupuncture in the treatment of post-stroke depression.** **Evaluation and analysis of drug-use in hospitals of China.2020;20(6):678-681.**

1. **Tian, J 2020**

- **Tian J, Bai Y J, You A M, Yao J P.** **Effect of Jieyu Wan combined with escitalopram on neurological function and serum NF- κB,5-HT, mir-146 and mir-221-3p in patients with post-stroke depression.** **Information on Traditional Chinese Medicine.2020, November;37(6):96-100.**

1. **Tian, X 2021**

- **Tian X S, Gong Y T, Ji C S, Zhang C.** **Clinical efficacy of Deanxit combined with Wuling Capsule in treating post-stroke depression.** **Information on Traditional Chinese Medicine.2021, January;38(1):63-66.**

1. **Wan, A 2006**

- **Wan A L, Yuan Y F.** **Curative effects on post-stroke depression with Wuling capsule versus sailuote capsule.** **Chinese Journal of Clinical Rehabilitation.2006, August;10(31):4-6.**

1. **Wang, T 2021**

- **Wang T, Dong H H, Ji S, Jin X G.** **Clinical effect of venlafaxine combined with ShuganJieyu capsule on depression after cerebral infarction.2021, May;27(3):101-104.**

1. **Wang, Z 2008**

- **Wang Z Y.** **Effect of fluoxetine combined with Free and Easy Wanderer Plus (FEWP) on post-stroke depression.** **Journal of Medical Forum.2008, March;29(5)：75-76.**

1. **Wu, H 2015**

- **Wu H Y, Lin P, He X S, Lu Z Y.** **Clinical effect of Shugan Jieyu capsule combined with Deanxit on post-stroke depression.** **China Prac Med.2015, April;10(10):134-136.**

1. **Wu, W 2017**

- **Wu W B.** **Clinical comparison of Shugan Jieyu capsule and Deanxit in the treatment of post-stroke depression. Psychological Doctor.2017,December;23(35):75.**

1. **Wu, Y 2016**

- **Wu Y G, Shi X M, Jiang Q, Chu Z H.** **Clinical research of duloxetine combined with morinda officinalis oligose capsule on the treatment of post-stroke depression.** **Chin J Clin Pharmacol Ther.2016 July;21(7):816-820.**

1. **Xie, Y 2018**

- **Xie Y, Gao Z Y, Wang X H, Li X J, Wang X Q. Clinical efficacy of Wuling Capsule combined with western medicine in the treatment of stroke depression and its effects on serum inflammatory factors and NPY.** **WORLD CHINESE MEDICINE.2018, March;13(3):679-682.**

1. **Xu, B 2007**

- **Xu B, Zhou M Y, Zhang S J. Observation on effect of** **Wuling Capsule in treating poststroke depression.** **CJITWM.2007, July;27(7):640-642.**

1. **Yang, H 2018**

- **Yang H J, Cui J L, Zhang Z Y.** **Clinical curative effect of Jieyu pill combined with paroxetine in treatment of post-stroke depression. SHAANXI JOURNAL OF TRADITIONAL CHINESE MEDICINE.2018,November;39(11):1530-1532.**

1. **Ye, Y 2021**

- **Ye Y, Zhao W, Zheng Q X.** **Clinical study on Shugan Jieyu Capsules combined with fluoxetine for post- stroke depression.** **JOURNAL OF NEW CHINESE MEDICINE.2021, March ;53(5):75-78.**

1. **Yu, W 2014**

- **Yu W Q, Liu T J, Huang K X. Clinical observation on Wuling Capsule combined with Deanxit in stroke depression treatment.** **Journal of Qiqihar Medical College.2014;35(10):1411-1412.**

1. **Zeng, M 2018**

- **Zeng M L, Chen L, Ni B, Chen K L, Zhi H Y, Zhang Y T, Hu W H. Effects of Xiaoyao Pill Combined with Fluoxetine in Treating Post-stroke Depression and its Influence on the Serum 5-Serotonin Level Zhejiang JITCWM.2018;28(12):997-999.**

1. **Zhang, M 2019**

- **Zhang M S, Zhang M, Li Q, Liu S Y.** **A control study on the effect of sertraline and Wuling capsule on serum neurotransmitters in depression after cerebral infarction.** **Chinese Journal of Integrative Medicine on Cardio-/Cerebrovascuiar Disease.2019, December;17(24):4066-4068.**

1. **Zhang, Y 2013**

- **Zhang Y, Xu B.** **Effect of venlafaxine sustained-release tablet combined with Shuganjieyu capsule in the treatment of post- stroke depression.** **Chin J Prim Med Pharm.2013, October;20(20):3074-3076.**

1. **Zhao, B 2005**

- **Zhao B R, Fu K F. Clinical observation on 64 cases of post-stroke depression treated with Luyoutai. Chin J Medic Guide.2005, April;3(4):420-421.**

1. **Zhao, G 2015**

- **Zhao G J, Tao F. Efficacy observation of shugan-jieyu capsule and escitalopram in treating post-stroke depression. Chin J Prim Med Pharm.2015, December;22(22):3470-3472.**

1. **Zhao, Z 2013**

- **Zhao Z, Pan M, Zhang S Q, He Y.** **Shugan Jieyu capsule combined with paroxetine tablets in the treatment of 40 cases of post-stroke depression.** **TCM Res.2013,July;26(7)：22-24.**
